# Chromium photocatalysis: accessing structural complements to Diels–Alder adducts with electron-deficient dienophiles†
†Electronic supplementary information (ESI) available: Experimental procedures, characterization data, electrochemistry data, UV/visible data, NMR spectra. See DOI: 10.1039/c6sc03303b


**DOI:** 10.1039/c6sc03303b

**Published:** 2016-09-12

**Authors:** Susan M. Stevenson, Robert F. Higgins, Matthew P. Shores, Eric M. Ferreira

**Affiliations:** a Department of Chemistry , University of Georgia , Athens , GA 30602 , USA . Email: emferr@uga.edu; b Department of Chemistry , Colorado State University , Fort Collins , CO 80523 , USA

## Abstract

A chromium-catalyzed, visible light-activated net [4 + 2] cycloaddition between dienes and electron-deficient alkenes is described. Gathered evidence, *via* control experiments, isolated intermediates, and measured redox potentials, points to several converging reaction pathways that afford the cyclohexene adducts, including a photochemical [2 + 2] cycloaddition/vinylcyclobutane rearrangement cascade and a substrate excitation/oxidation sequence to a radical cation intermediate. Notably, the accompanying mechanistic stipulations result in a process that yields regioisomeric compounds from those generated by traditional Diels–Alder cycloadditions.

## Abstract 2

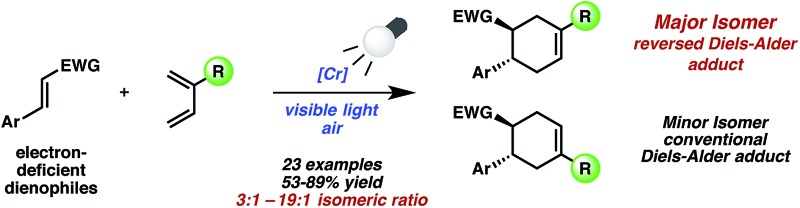
A Cr-photocatalyzed [4 + 2] cycloaddition between dienes and electron-deficient alkenes is reported, accessed by up to three converging pathways to yield the “*meta*” adducts.

## Introduction

In the past decade, the renaissance of photoredox catalysis has generated renewed interest in radical cation accelerated reactions initiated through photoinduced electron transfer (PET).^1^ Notable examples in the area of radical cation [2 + 2] and [4 + 2] cycloadditions^2^ have utilized metal photoredox catalysts containing Ru^3^ and Ir^4^ ions, as well as triarylpyrylium salts,^5^ as light-activated single-electron oxidants. In the interest of advancing sustainable chemical transformations,^6^ there has recently been a shift toward developing photocatalysts based on more earth-abundant metals, with particular achievements reported using Cu-^7^ and Fe-containing^8^ systems. Our groups have investigated strongly oxidizing poly-pyridyl and -phenanthrolinyl Cr photocatalysts.^9^ We reported that these light-activated Cr(iii) complexes (*E*_1/2_ = +1.33–1.84 V *vs.* SCE^10^) are capable of promoting radical cation Diels–Alder cycloadditions of electron-rich alkenes,^9*b*^ akin to the Ru-initiated examples from Yoon in 2011.^11^ Mechanistically, however, the Cr-photocatalyzed cycloaddition differs from the Ru version^12^ in that the reaction catalyzed by [Cr(Ph_2_phen)_3_](BF_4_)_3_ favored an oxygen-mediated catalytic cycle over radical chain propagation.^9*c*^ This unique behavior encouraged us to investigate the synthetic utility of the Cr photocatalysts further.

**Fig. 1:**
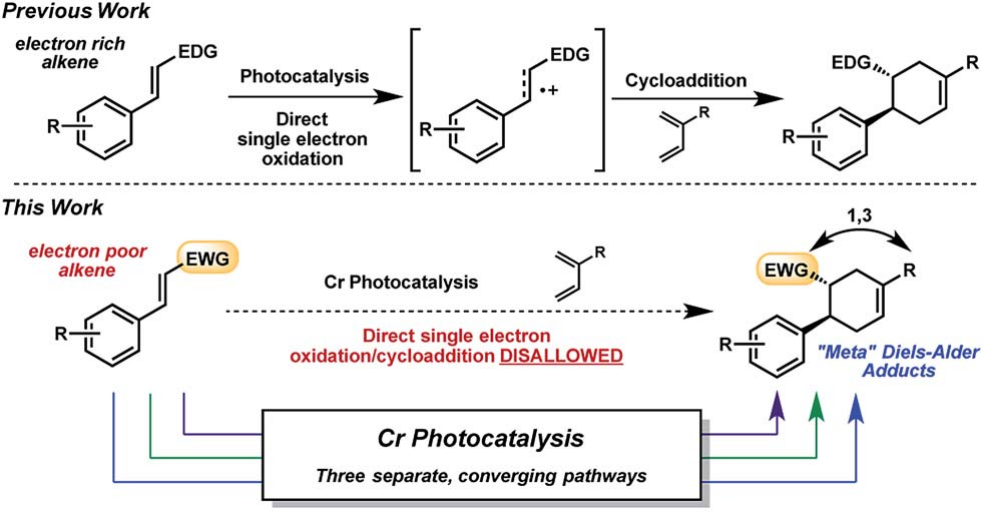
Photocatalyzed [4 + 2] cycloadditions with electron deficient alkenes.

The radical cation [4 + 2] cycloaddition using Ru or Cr with light was relatively constrained by the requirement of sufficiently oxidizable alkenes, a common stipulation in these reactions.^13^ Being mindful that photocatalysis has often provided an entry point for the construction of nonintuitive bonds *via* novel reaction manifolds,^14^ we wondered whether electron-poor dienophiles *outside of the oxidizable realm* could also yield cycloaddition products. Though electron-poor olefins would typically be expected to participate in [4 + 2] cycloadditions through more conventional, LUMO-lowering activation modes (*e.g.*, thermal, Lewis acid), a photochemical strategy could broaden the range of Diels–Alder cycloadducts attainable, and perhaps offer orthogonal selectivity profiles (*e.g.*, regio-, diastereo-). Herein, we report the Cr-photocatalyzed cycloaddition of electron-poor olefins with dienes (Fig. 1). Experiments implicate multiple operative mechanistic pathways that converge to form the same cycloadducts. Importantly, the described transformation results in Diels–Alder products of *reversed* regioselectivity, yielding “*meta*” adducts as opposed to the “*ortho*” and “*para*” adducts generated under conventional activation.

## Results and discussion

### Reaction development

**Fig. 2:**
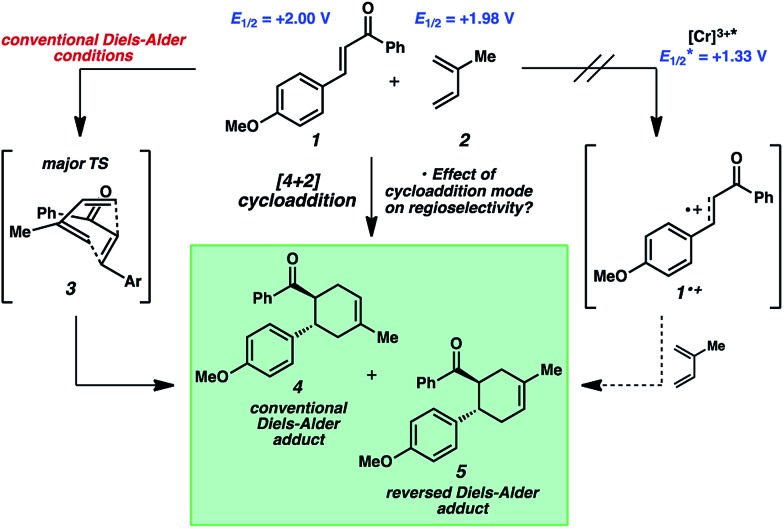
Cycloaddition reactivity of 4-methoxychalcone.

The reactivity of 4-methoxychalcone is illustrative (**1**, Fig. 2). In intermolecular Diels–Alder cycloadditions using conventional activation, enones of this type routinely require rather forcing conditions and yield predominantly adduct **4**.^15^ Based on our previous efforts, we reasoned that the cycloaddition of 4-methoxychalcone through a photocatalytic oxidation pathway would be out of the question, as the oxidation potential of enone **1** (+2.00 V, in CH_3_NO_2_) is too positive to be oxidized by the [Cr(Ph_2_phen)_3_]^3+^ catalyst (
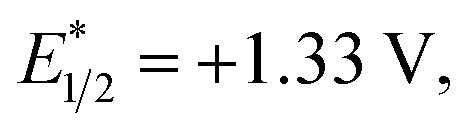
 in CH_3_NO_2_) in the pathway as described for the electron-rich dienophiles.^9b,16^ Thus, under Cr-photocatalysis conditions, no cycloaddition between 4-methoxychalcone (**1**) and isoprene (**2**) should occur.

**Scheme 1:**
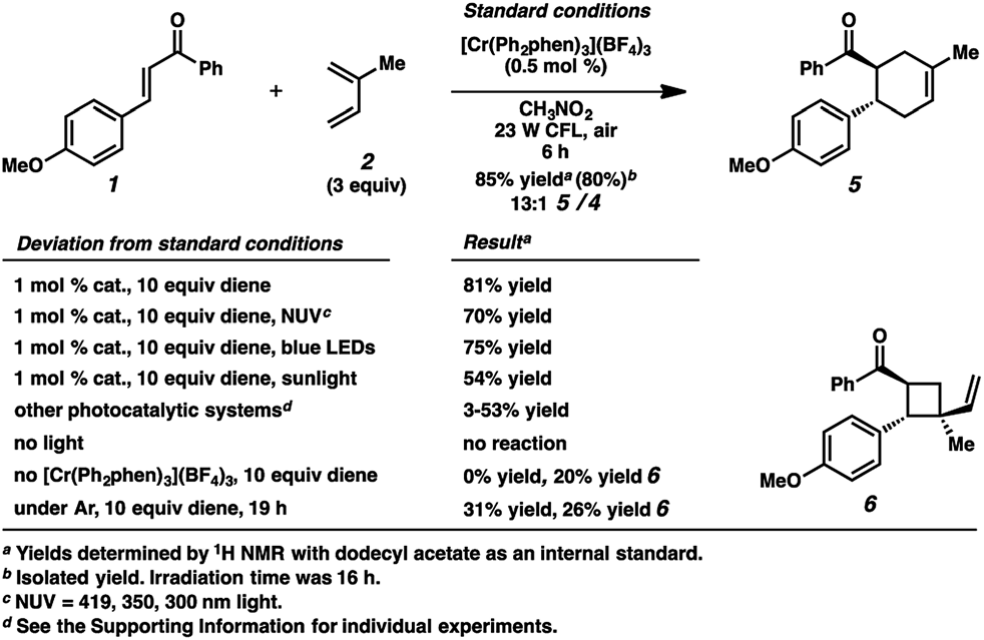
Selected optimization experiments.

Surprisingly, however, when enone **1** was exposed to [Cr(Ph_2_phen)_3_](BF_4_)_3_ in the presence of isoprene (**2**) and irradiation with a 23 W compact fluorescent light bulb, cycloadduct **5** was formed in 85% yield (80% isolated yield, Scheme 1). Remarkably, the regioselectivity of this cycloaddition was 13 : 1 favoring the reversed Diels–Alder adduct (**5**). We found that increased catalyst loading and equivalents of diene did not increase the yield. Near-UV (NUV), blue LEDs, and sunlight also effected this transformation, but optimal results were achieved with the 23 W CFL source. Other photoredox catalysts were less effective (*vide infra*). A control experiment confirmed that no reaction occurred without light. Notably, with light but in the absence of catalyst, vinylcyclobutane **6** was formed in 20% yield, presumably *via* photochemical [2 + 2] cycloaddition between enone **1** and isoprene,^17^ but no [4 + 2] products were detected. Lastly, performing the reaction in the absence of air significantly decelerated the formation of cyclohexene **5**, while vinylcyclobutane **6** also formed. We had previously found oxygen to be important for Cr-mediated photocatalysis with electron-rich substrates (*vide infra*).^9b,c,18^

### Substrate scope

**Scheme 2:**
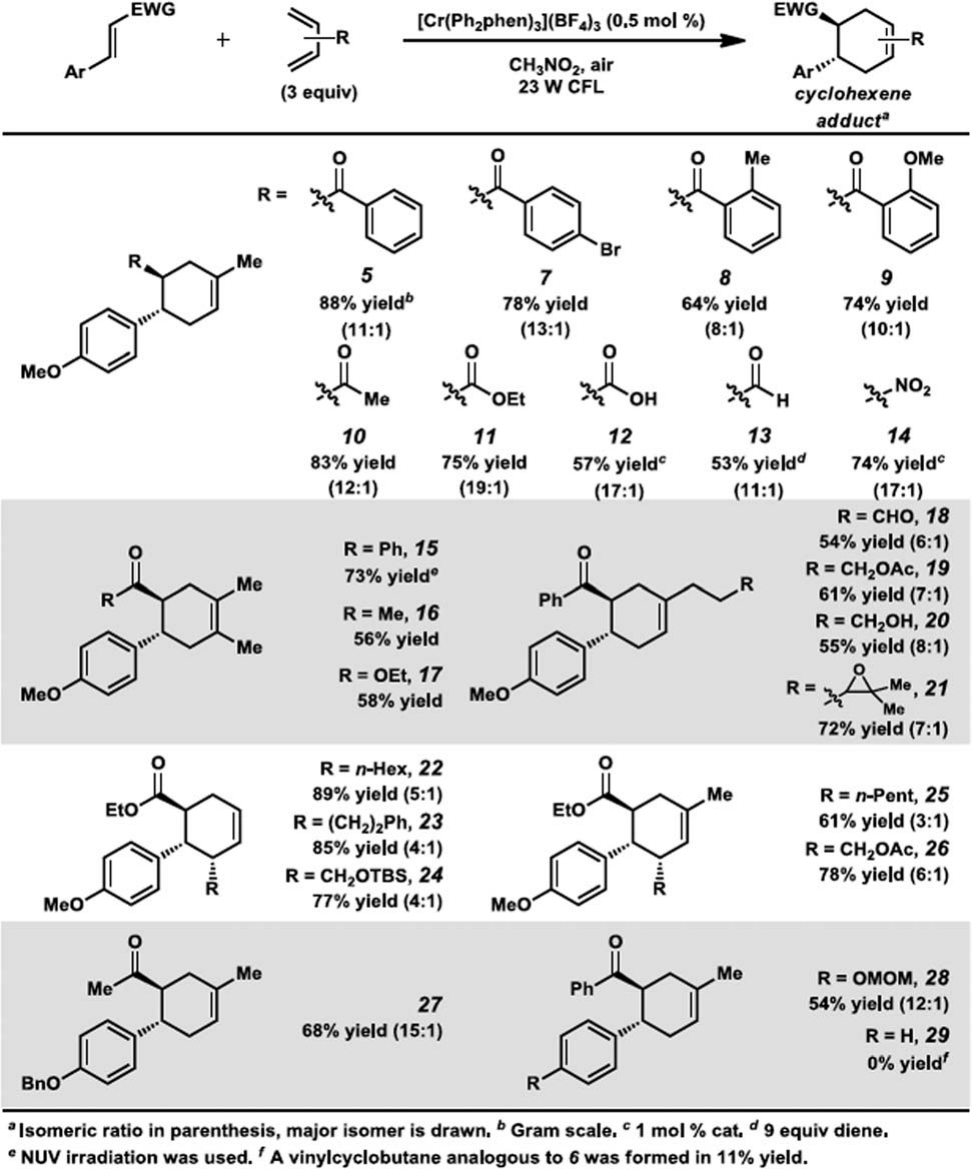
The Cr-photocatalyzed [4 + 2] cycloaddition between dienes and electron-deficient alkenes-scope.

Encouraged by the success of this reaction, we set out to test the limits of these cycloadditions by examining other electron-poor olefins (Scheme 2). We were delighted to find this process was successful for a range of cycloaddition partners. Several differentially substituted chalcone derivatives were found to be viable substrates for this transformation (**5**, **7–9**). Other α,β-unsaturated carbonyl compounds reacted in moderate to high yields (**10–13**). A nitroolefin was also a competent substrate, forming cyclohexene **14** in 74% yield. Consistent with the 4-methoxychalcone case, the transformations with isoprene all proceeded with high levels of regioselectivity (8 : 1 to 19 : 1). Symmetrical 2,3-dimethyl-1,3-butadiene could be used with several of these dienophiles, affording cyclohexenes **15–17** in good yields. Regioselectivity considerations are not applicable for this diene, but it is still remarkable that this method circumvents the forcing conditions generally required to obtain these adducts.^19^ Differentially substituted dienes were also proficient in this reaction. The cycloaddition conditions were tolerant of a variety of functional groups on the diene (**18–21**, **24**, **26**). In addition, terminally substituted dienes gave cyclohexenes **22–26** in high yields. Somewhat lower regioselectivity ratios were observed, but stereoselectivity was excellent in these processes, as the major constitutional isomer was observed as a single diastereomer.^20^ Lastly, enones containing different electron-rich aryl groups at the β-position also participated in the reaction (**27**, **28**), although we note here the scope was more limited.^21^ For example, an enone with an electron-neutral arene was not productive in this transformation (**29**).

### Mechanistic pathways

We have found in our initial mechanistic studies the potentially operative pathways deviate significantly from the direct oxidation route. As mentioned previously, accounting for reduction potentials it is unlikely that the photoexcited Cr catalyst (Cr^3+^*^/2+^ *E*_1/2_ = +1.33 V in CH_3_NO_2_) could be directly oxidizing 4-methoxychalcone (**1** ^+/0^, +2.00 V) or isoprene (**2** ^+/0^, +1.98 V (ref. 22)) to initiate the cycloaddition.^23^ Furthermore, all attempts to catalyze the cycloaddition using photocatalysts with more positive excited state reduction potentials, such as [Ru(bpz)_3_]^2+^, triphenylpyrilium, Mes-Acr, or a variety of cyanoarenes and chloranil,^24^ provided lower yields than [Cr(Ph_2_phen)_3_](BF_4_)_3_ (see the ESI†), indicating that this transformation is likely not proceeding *via* simple direct oxidation of the electron deficient alkene. An energy transfer pathway could also be ruled out since the long-lived excited state of 
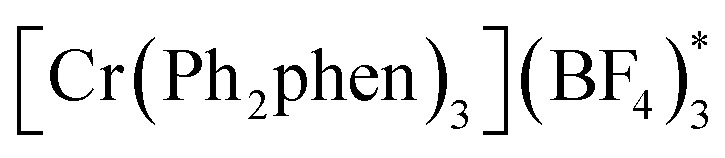
 (38 kcal mol^–1^) is considerably lower than the transfer energies of either the 4-methoxychalcone (**1**) or isoprene (the triplet excited state energies are both ∼60 kcal mol^–1^).^25^

**Fig. 3:**
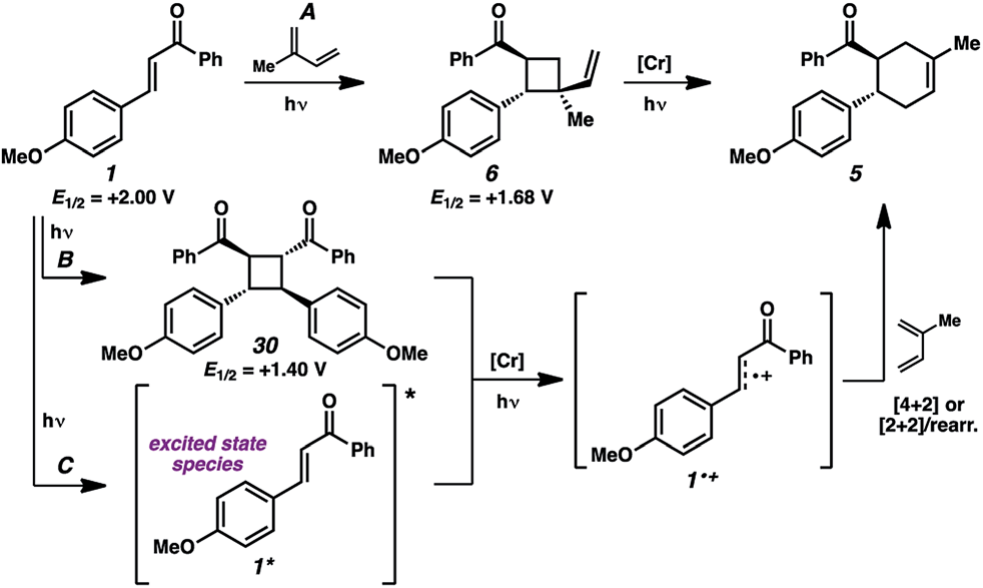
Possible mechanistic pathways.

**Scheme 3:**
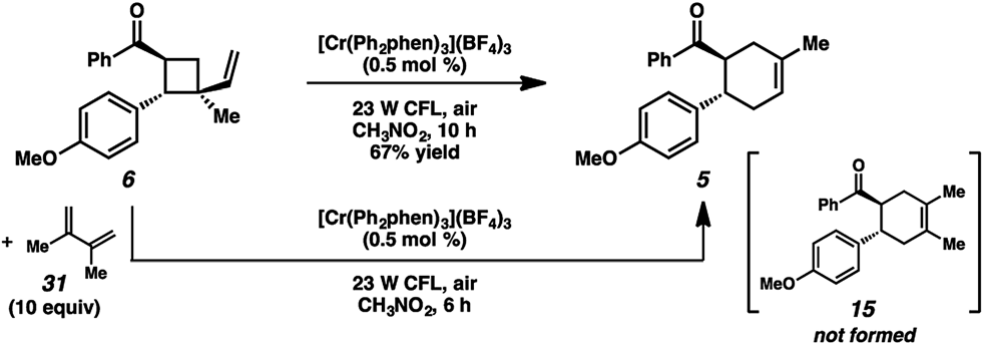
Rearrangement of vinylcyclobutane **6**.

We thus considered other mechanistic pathways (Fig. 3). One possibility is that the reaction is proceeding through intermediate vinylcyclobutane **6** (*Pathway A*), which we observed when the reaction was performed without catalyst.^26^ Indeed, when vinylcyclobutane **6** (*E*_1/2_ = +1.68 V in CH_3_NO_2_) was exposed to the Cr conditions, product **5** was formed in 67% yield as a single isomer (Scheme 3).^27^ When this rearrangement was performed in the presence of excess diene **31**, only the rearrangement product was observed and not the cross-adduct between 4-methoxychalcone and the added diene, confirming that cyclohexene **5** is forming through direct rearrangement of vinylcyclobutane **6** and not through cycloreversion/recombination.^28,29^ Thus, we believe that a cascade pathway involving photochemical [2 + 2] cycloaddition (**1**, λ_max_ = 340 nm) followed by single-electron oxidative vinylcyclobutane rearrangement^30^ is viable and occurring. We note, however, that cyclobutane **6** was formed in only 20% yield without catalyst, while 85% yield of cyclohexene **5** was formed with catalyst in the same duration, suggesting that this [2 + 2]/rearrangement may not be the only mechanistic pathway.

**Scheme 4:**
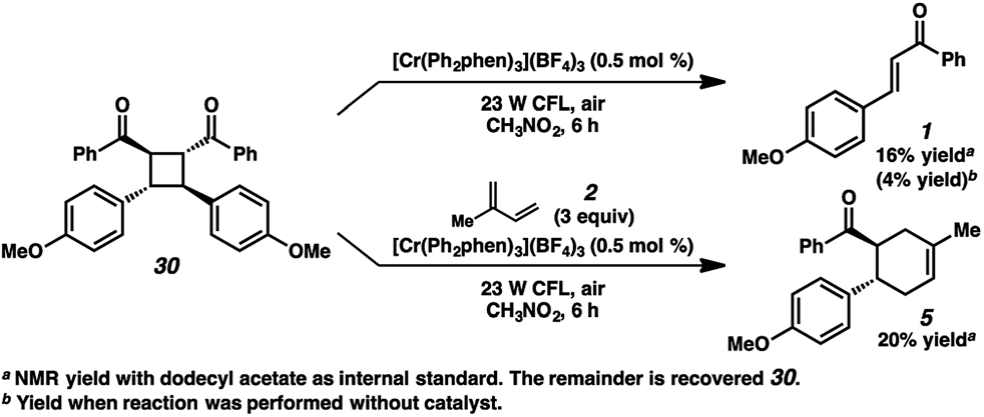
Cycloreversion of enone dimer **30**.

A different route, *Pathway B* offers a means of accessing a reactive enone radical cation (**1˙^+^**, Fig. 3). If *in situ* dimerization of enone **1** occurs,^31^ the resulting dimer (**30**) has a significantly lower reduction potential (*E*_1/2_ = +1.40 V, in CH_3_NO_2_) than the starting enone. Oxidation could then induce cycloreversion to the radical cation, and interception of this putative radical cation with the diene could afford the cycloaddition product (*via* direct [4 + 2] and/or [2 + 2]/rearrangement).^32^ Control experiments suggest this pathway is a minor contributor at most. First, enone dimer **30** was found to form in only trace amounts under the standard Cr conditions in the absence of diene. Furthermore, independently synthesized enone dimer **30** subjected to irradiation affords retrocyclobutanation with and without catalyst, but in low yields (Scheme 4). When dimer **30** was exposed to the cycloaddition conditions in place of enone **1**, cyclohexene **5** formed in 20% yield, at least implicating the feasibility of this pathway. Notwithstanding, the low production of dimer **30** and the relatively sluggish cycloreversion suggests a lesser role in the formation of cyclohexene **5**.

An alternative pathway for the generation of **1˙^+^** recognizes that the photoexcited enone (1*) will be easier to oxidize than its ground state form (Fig. 3, *Pathway C*). Select reports indicate that the excitation of chalcone derivatives and related α,β-unsaturated carbonyl species lead to excimers^33^ and/or charge-transfer complexes,^34^ which enable single-electron oxidation.^35^ The oxidation of enone 1* in this manner would generate radical cation **1˙^+^**, which would proceed to product **5** as described earlier. Here, we note that enone **1** shows weak emission at 443 nm when excited at 340 nm in acetonitrile, from which we estimate an excited state reduction potential (**1˙^+^** ^/0*^) of –0.80 V, which suggests thermodynamic competency for oxidation of 1* by the photoexcited Cr catalyst. The triplet excited state lifetime of enone **1** was reported to be 23 and 29 ns in heptane and methanol, respectively,^25*a*^ suggesting this is well within the range of feasibility for oxidation by the long-lived excited state of the Cr catalyst.

**Scheme 5:**
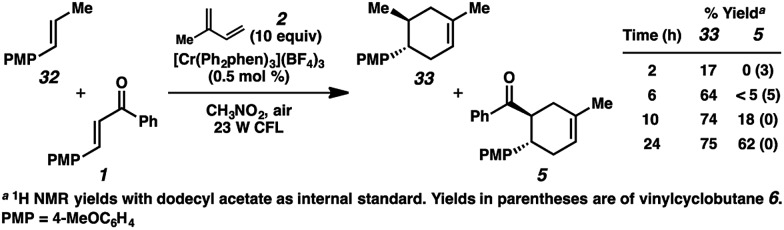
Competition experiment with *trans*-anethole.

Bauld and coworkers have discussed the inhibitory effect of *trans*-anethole on competing radical cation cycloadditions and vinylcyclobutane rearrangements due to its highly oxidizable nature (*E*_1/2_ = +1.35 V (ref. 9*c*)).^22,36^ Thus, if the cycloaddition of enone **1** and isoprene (**2**) is proceeding through a radical cation (*e.g.*, **1˙^+^**), we would expect the addition of *trans*-anethole to considerably impede the formation of cycloadduct **5**. In accordance with this hypothesis, in a competition experiment using a 1 : 1 mixture of *trans*-anethole (**32**) and enone **1** with excess isoprene, the cycloaddition with enone **1** proceeded only after the majority of **32** was consumed (Scheme 5). The inhibitory effect of alkene **32** may implicate the intermediacy of the enone radical cation (**1˙^+^**) in the overall process.

In an effort to probe the termination step(s) concerning all possible pathways, quantum yield experiments were performed. Since compounds **1**, **6**, and **30** can all be invoked as starting materials or intermediates in the mechanism, each was tested for their photochemical efficiency. At 350 nm excitation, maximum quantum yield values for species **1**, **6**, and **30** were *Φ* = 0.013, 0.45, and 0.013, respectively. Compounds **1**, **6**, and **30** do absorb light at 350 nm (as does nitromethane), and their equilibria in solution complicate the calculation of an accurate quenching factor. Therefore compound **6**, which showed the largest quantum yield, was excited at 400 nm in the presence of [Cr(Ph_2_phen)_3_]^3+^ in CD_3_NO_2_ where the maximum quantum yield was *Φ* = 0.93. Since only [Cr(Ph_2_phen)_3_]^3+^ absorbs light at this wavelength, the quenching factor is assumed to be ∼1, giving a chain length value of <1. These data corroborate the lack of a predominant radical chain mechanism^12^ in the reaction manifold. Moreover, when a deoxygenated sample containing **6** and [Cr(Ph_2_phen)_3_]^3+^ was irradiated at 400 nm in nitromethane, the quantum yield decreased to a maximum value of *Φ* = 0.21, further implicating this reactivity as photocatalytic instead of photoinitiated, akin to our previous report.^9*c*^

The presence of oxygen in this transformation also deserves mention. In our earlier study, we had discussed the roles of O_2_ in the cycloaddition using electron-rich alkenes. O_2_ was essential, and the absence of it shut down catalysis altogether. Singlet oxygen was formed in these cycloadditions *via* a quench of the long-lived Cr(iii) excited state. The singlet oxygen is then reduced to superoxide by Cr(ii), and the superoxide then reduces the cycloadduct radical cation. In this specific reaction with electron-deficient alkenes, the effect of O_2_ is still beneficial to the overall reaction progress, but not nearly to the same extent as the earlier transformation (*i.e.*, reaction rates are slower, but the cyclohexene product is still formed *via* catalytic turnover). We believe singlet oxygen is formed in these cycloadditions as well. In the analysis of the reaction and the substrate scope, in several cases we noted the formation of a minor byproduct (average <5% yield). We determined this byproduct to be an allylic hydroperoxide, presumably arising from the oxidation of the cyclohexene product by ^1^O_2_.^37^ It is possible that we may be amplifying or diminishing specific reaction pathways in the presence of O_2_; future studies may elucidate its multifaceted effects.

From the evidence amassed thus far we conclude that this Cr-photocatalyzed cycloaddition using an electron deficient dienophile can occur through several reaction pathways involving photochemical and radical cation processes, all outside of the direct electron transfer or energy transfer pathways. Coincidentally, the operative pathways all converge to the same cyclohexene adducts. Further mechanistic studies are underway.^38^

### Diels–Alder regioselectivity analysis

A hallmark characteristic of the Diels–Alder reaction is its highly predictive regioselective outcomes. An electron-withdrawing group on the dienophile and an electron-donating group on the diene will impact the coefficients of the FMOs so as to dictate the overall regioselectivity of the cycloaddition (*i.e.*, the “*ortho*–*para* rule”). Efforts to reverse this natural regioselectivity of Diels–Alder cycloadditions have been reported, but only a handful of strategies have been successful.^39,40^ These include the incorporation of electronically steering substituents that can be subsequently removed,^41^ and catalyzed *vs.* thermal/noncatalyzed cycloadditions that adjust the molecular orbital coefficients of the reactants (*e.g.*, selective coordination of a sterically unhindered carbonyl).^42^ Table 1 depicts an alternative strategy to achieve this unnatural regioselectivity. Example dienes and β-(*para*-methoxyphenyl)nitroethylene were combined using both previously described LiClO_4_/CH_3_NO_2_ conditions^43^ and our chromium photocatalyzed conditions. As can be seen, these transformations each proceed to afford complementary regiochemistries, where the photocatalyzed reactions occur with high selectivity for the unconventional Diels–Alder adducts. This unconventional regioselectivity tracks consistently with all of the selectivities observed in the cases in Scheme 2. These transformations represent a novel, conditions-based approach to invert the Diels–Alder regioselectivity with reactive diene/dienophile partners.

**Table 1:** Reversed regioselectivity under Cr-photocatalysis conditions

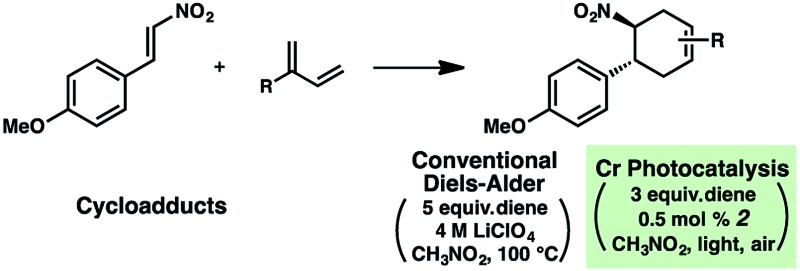
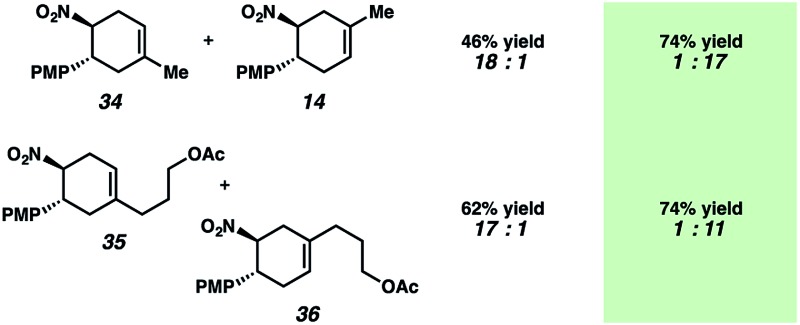

**Fig. 4:**
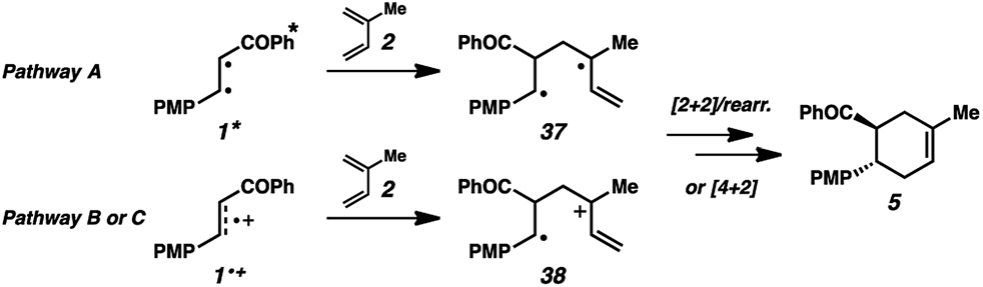
General regiochemical explanation.

Our hypothesis for the observed regioselectivity is illustrated in Fig. 4, using chalcone **1** and isoprene as an example. Notably, whether the cycloaddition is proceeding through a photochemical “head-to-tail” [2 + 2] cycloaddition^44^ followed by a radical cation vinylcyclobutane rearrangement, or an enone radical cation pathway, the net reversed Diels–Alder regioselectivity should be favored. With isoprene (**2**), the methyl substituent allows selective generation of the more stable diradical or radical cation intermediate. Presumably, the aryl group also stabilizes this intermediate species to some extent.

## Conclusions

The deliberate investigations of photocatalytic manifolds with various components have unlocked new transformations that possess tremendous potential for the chemistry community. We have shown that that the Cr-catalyzed [4 + 2] cycloaddition of dienes with electron-poor alkenes (*i.e.*, outside of the oxidizable realm) can yield cyclohexene adducts, and, perhaps most pertinently, with opposite regioselectivities to those provided *via* the traditional Diels–Alder cycloaddition. In as much as the previous Cr-catalyzed [4 + 2] cycloaddition is a chemoselective complement to the Diels–Alder reaction, this example represents a *regioselective* complement to the transformation. This process is optimally effective using Cr photocatalysis. Mechanistic investigations elucidating the unique impact of Cr, and efforts to expand this chemistry, will be reported in due course.
