# Supplementary material for: Chromium photocatalysis: accessing structural complements to Diels–Alder adducts with electron-deficient dienophiles
Source: Chem Sci. 2016 Sep 12;8(1):654–60. doi: 10.1039/c6sc03303b (PMC5297334; doi:10.1039/c6sc03303b)

Electronic Supplementary Material (ESI) for Chemical Science.
This journal is © The Royal Society of Chemistry 2016

Supporting Information

Chromium Photocatalysis: Accessing Structural Complements to Diels-Alder Adducts with Electron-Deficient Dienophiles

Susan M. Stevenson,[a] Robert F. Higgins,[b] Matthew P. Shores,[b] and Eric M. Ferreira*[a]

[a]*Department of Chemistry, University of Georgia, Athens, GA 30602, United States*

[b]*Department of Chemistry, Colorado State University, Fort Collins, CO 80523, United States*

emferr@uga.edu

Table of Contents

Additional References........S2

Materials and Methods........S3

Cr-Photocatalyzed Cycloaddition........S5

Optimization Table........S23

Catalyst Evaluation........S25

Electrochemical Data........S28

Cr-Photocatalyzed Vinylcyclobutane Rearrangement........S30

Vinylcyclobutane Trapping Experiments........S31

UV/Vis Spectra........S33

Enone Dimer Experiments........S34

Static Emission Spectrum of 4-Methoxychalcone........S36

Competition Experiment........S37

Reactivity of Unsubstituted Chalcone........S38

Normal Electron-Demand Diels-Alder Reactions........S39

Thermal Diels-Alder Reactions........S41

Synthesis of Electron-Poor Alkenes........S43

Synthesis of Dienes........S51

Photochemical [2+2] Cycloaddition........S56

Structural and Stereochemical Determination of 1-Substituted Diene Products........S59

Identification of Minor Isomer........S64

Oxidative Cleavage of PMP Group........S66

Supporting Information References........S68

Spectra Compilation........S70

Additional References

Select Intramolecular Diels-Alder Reactions of Enones with Conventional Activation (ref 15):

High heat: a) T. Nomura, T. Pukai, T. Narita, S. Terada, J. Uzawa, Y. Iitaka, *Tetrahedron Lett*. **1981**, *22*, 2195-2198; b) C. M. Brito, D. C. G. A. Pinto, A. M. S. Silva, A. M. G. Silva, A. C. Tomé, J. A. S. Cavaleiro, *Eur. J. Org. Chem.* **2006**, 2558-2569; c) C. F. Chee, I. Abdullah, M. J. C. Buckle, N. A. Rahman, *Tetrahedron Lett*. **2010**, *51*, 495-498.

High pressure: a) A. C. Kinsman, M. A. Kerr, *Org. Lett*. **2000**, *2*, 3517-3520; b) E. Ballerini, L. Minuti, O. Piermatti, *J. Org. Chem*. **2010**, *75*, 4251-4260.

Microwaves: a) M. Karthikeyan, R. Kamakshi, V. Sridar, B. S. R. Reddy, *Synth. Commun*. **2003**, *33*, 4199-4204; b) R. Kamakshi, B. S. R. Reddy, *J. Polym. Sci. A Polym. Chem*. **2008**, *46*, 1521-1531.

Uncommon reagents: a) M. F. Reetz, N. Jiao, *Angew. Chem., Int. Ed*. **2006**, *45*, 2416-2419; b) J. L. Corbett, R. T. Weaver, *Synth. Commun*. **2008**, *38*, 489-498; c) H. Cong, D. Ledbetter, G. T. Rowe, J. P. Caradonna, J. A. Porco, *J. Am. Chem. Soc.* **2008**, *130*, 9214-9215; d) H. Cong, C. F. Becker, S. J. Elliott, M. W. Grinstaff, J. A. Porco, *J. Am. Chem. Soc*. **2010**, *132*, 7514-7518; e) J. Bah, J. Franzén, *Chem. Eur. J*. **2014**, *20*, 1066-1072.

Select Investigations into [4+2] vs. [2+2]/Vinylcyclobutane Rearrangements in Electron Transfer Processes (ref 26):

a) R. A. Neunteufel, D. R. Arnold, *J. Am. Chem. Soc.* **1973**, *95*, 4080-4081; b) T. Asanuma, M. Yamamoto, Y. Nishijima, *J. Chem. Soc., Chem. Commun*. **1975**, 608-609; c) T. Asanuma, T. Gotoh, A. Tsuchida, M. Yamamoto, Y. Nishijima, *J. Chem. Soc., Chem. Commun*. **1977**, 485-486; d) A. J. Maroulis, D. R. Arnold, *J. Chem. Soc., Chem. Commun*. **1979**, 351-352; e) S. L. Mattes, S. Farid, *J. Am. Chem. Soc*. **1983**, *105*, 1386-1387; f) M. Yamamoto, H. Yoshikawa, T. Gotoh, Y. Nishijima, *Bull. Chem. Soc. Jpn*. **1983**, *56*, 2531-2532; g) S. Kadota, K. Tsubono, K. Makino, M. Takeshita, T. Kikuchi, *Tetrahedron Lett*. **1987**, *28*, 2857-2860; h) N. P. Schepp, L. J. Johnston, *J. Am. Chem. Soc*. **1994**, *116*, 6895-6903; i) M. Schmittel, H. von Seggern, *J. Am. Chem. Soc*. **1993**, *115*, 2165-2177; j) N. P. Schepp, D. Shukla, H. Sarker, N. L. Bauld, L. J. Johnston, *J. Am. Chem. Soc*. **1997**, *119*, 10325-10334; k) L. L. O'Neil, O. Wiest, *J. Org. Chem*. **2006**, *71*, 8926-8933; l) X. Wang, C. Chen, *Tetrahedron* **2015**, *71*, 3690-3693.

Materials and Methods

Chromium catalysts were synthesized as previously described.[1,2] $Ru(bpz)_3(PF_6)_2$, 9-mesityl-10-methylacridinium tetrafluoroborate, and 2,4,6-triphenylpyrilium tetrafluoroborate were purchased from Sigma-Aldrich. All solvents, excluding acetonitrile and nitromethane, were purified by passing through activated alumina columns; acetonitrile was distilled over CaH_2, and nitromethane was purchased from Alfa Aesar (98+%, A11806) and used without further purification. All reagents were used as received unless otherwise noted. Commercially available chemicals were purchased from Alfa Aesar (Ward Hill, MA), Sigma-Aldrich (St. Louis, MO), Oakwood Products, (West Columbia, SC), Strem (Newburyport, MA) and TCI America (Portland, OR). Qualitative TLC analysis was performed on 250 mm thick, 60 Å, glass backed, F254 silica (Silicycle, Quebec City, Canada). Visualization was accomplished with UV light and exposure to *p*-anisaldehyde or $KMnO_4$ solution followed by heating. Flash chromatography was performed using Silicycle silica gel (230-400 mesh). Reactions under NUV irradiation were performed in a Luzchem chamber reactor equipped with 10 lamps of wavelengths 419, 350, and 300 nm. Irradiation with visible light was performed in a sealed box using a 23 W compact fluorescent light bulb (EcoSmart 23 W bright white CFL spiral light bulb, 1600 lumens). NMR spectra were acquired at the University of Georgia Chemical Sciences Magnetic Resonance Facility on a Varian Mercury Plus 400 MHz NMR. ^1H NMR spectra were acquired at 400 MHz and are reported relative to $SiMe_4$ (δ 0.00). ^{13}C NMR spectra were acquired at 100 MHz and are reported relative to $SiMe_4$ (δ 0.00). IR spectra were obtained on a Shimadzu IRPrestige-21 FT-IR. High resolution mass spectrometry data were acquired by the Proteomics and Mass Spectrometry Facility at the University of Georgia on a Thermo Orbitrap Elite. Absorption spectra were obtained with a Hewlett-Packard 8453 spectrometer in quartz cuvettes with a 1 cm path length. Emission spectra were obtained with an Aviv automated titrating differential/ratio spectrofluorometer (model ATF105). Quantum yields and chain lengths were determined in a similar manner reported by Cismesia and Yoon.[3] Actinometry experiments were performed using a Newport TLS-300XU tunable light source, which includes a 200 watt Xe arc lamp, a Cornerstone 130 monochromator, and a motorized filter wheel; this instrument provides a wavelength range of 250-2400 nm and a wavelength selectivity of ±0.7 nm. Electrochemical experiments were performed in 0.1 M solutions of Bu_4NPF_6 in CH_3CN and CH_3NO_2. Cyclic voltammograms (CVs) and square-wave voltammograms

(SWVs) were recorded with a CH Instruments potentiostat (Model 1230A or 660C) using a 0.25 mm Pt disk or 0.25 mm glassy carbon disk working electrode, Ag^{+}/Ag reference electrode and a Pt wire auxiliary electrode. Scans were collected at a rate of 100 mV/s. Reported potentials are referenced to the $[Cp_2Fe]^{+}/[Cp_2Fe]$ redox couple (abbreviated Fc^{+}/Fc, where Cp = cyclopentadienyl), and were determined by adding ferrocene (which was sublimed before use) as an internal standard at the conclusion of each electrochemical experiment. Oxygenated experiments were performed by bubbling O_2 into the experimentation vessel for 10 seconds prior to data collection. Dark experiments were performed by removing the vessel from all light for 20 minutes before and during scans. To minimize electrode interactions with possible superoxide species present, the working electrode was polished before each set of experiments were performed. The surface of the working electrode was also cleaned with a Kimwipe before each scan to help provide a clean surface of the electrode.

Cr-Photocatalyzed Cycloaddition

General Notes: Irradiation was performed in a sealed box using a 23 W compact fluorescent light bulb; the temperature inside the box was recorded at ~45 °C. Reported NMR data are of the major isomer. Ratios of "opposite"/"normal" Diels-Alder isomers were determined through analysis of crude ^{1}H NMR spectra. Reported yields are for the mixture of isomers.

General Procedure for Cr-Photocatalyzed Cycloaddition: A flame-dried 2-dram borosilicate vial open to air was charged with the alkene (1 equiv), diene (3 equiv), $[Cr(Ph_2phen)_3](BF_4)_3$ (0.5 mol %), and nitromethane (0.10 M). The vial was then capped and placed in front of a bright white 23 W compact fluorescent light bulb in a closed box lined with aluminum foil. The solution was irradiated with stirring until consumption of the alkene was complete, as determined by TLC, and then it was passed through a short plug of silica (2.0-2.5 cm high x 1 cm wide, Et_2O eluent). The volatile materials were removed by rotary evaporation, and the resulting residue was purified by flash chromatography.

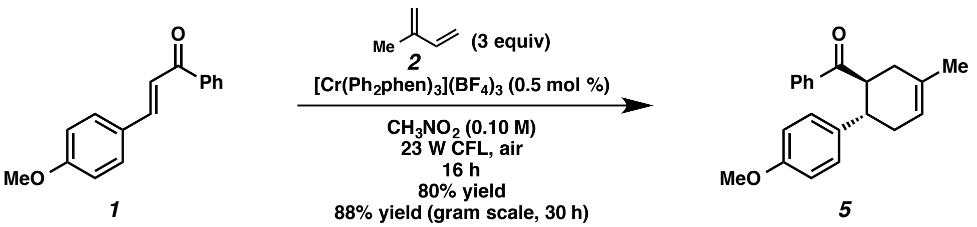

Cyclohexene 5. Prepared according to the *General Procedure* using alkene **1** (23.8 mg, 0.100 mmol), diene **2** (30.1 µL, 0.300 mmol), $[Cr(Ph_2phen)_3](BF_4)_3$ (0.7 mg, 0.000500 mmol), and nitromethane (1.00 mL). The reaction mixture was irradiated for 16 h. The crude product was purified by flash chromatography (100% hexanes → 9:1 hexanes/EtOAc eluent) to afford cyclohexene **5** (24.5 mg, 80% yield, 13:1 isomeric ratio) as a white solid.

Gram-scale procedure: Prepared according to a modified *General Procedure* (modification: the reaction was run in a 250-mL round-bottom flask and stoppered with a plastic cap) using alkene **1** (1.00 g, 4.20 mmol), diene **2** (1.26 mL,

12.6 mmol), $[Cr(Ph_2phen)_3](BF_4)_3$ (27.5 mg, 0.0210 mmol), and nitromethane (42.0 mL). The reaction mixture was irradiated for 30 h. The crude product was purified by flash chromatography (100% hexanes → 9:1 hexanes/EtOAc eluent) to afford cyclohexene **5** (1.13 g, 88% yield, 11:1 isomeric ratio) as a white solid.

TLC: R_f = 0.68 in 3:1 hexanes/EtOAc, visualized by UV.

^{1}H NMR (400 MHz; $CDCl_3$): δ 7.79 (d, *J* = 7.2 Hz, 2H), 7.47 (t, *J* = 7.2 Hz, 1H), 7.36 (t, *J* = 7.6 Hz, 2H), 7.09 (d, *J* = 8.7 Hz, 2H), 6.68 (d, *J* = 8.7 Hz, 2H), 5.54 (br s, 1H), 3.97 (td, *J* = 10.7, 5.5 Hz, 1H), 3.68 (s, 3H), 3.18 (td, *J* = 10.7, 5.5 Hz, 1H), 2.35-2.24 (comp. m, 4H), 1.72 (s, 3H).

^{13}C NMR (100 MHz; $CDCl_3$): δ 203.8, 157.8, 137.4, 135.7, 132.7, 128.39, 128.36, 128.0, 122.0, 120.9, 113.7, 55.1, 47.3, 41.5, 35.2, 34.2, 23.2.

IR (ATR, neat): 3055, 2909, 2839, 1682, 1512, 1242, 1034, 734, 702 cm^{-1}.

HRMS (ESI+): *m/z* calc'd for $(M + H)^+$ $[C_{21}H_{22}O_2 + H]^+$: 307.1693, found 307.1699.

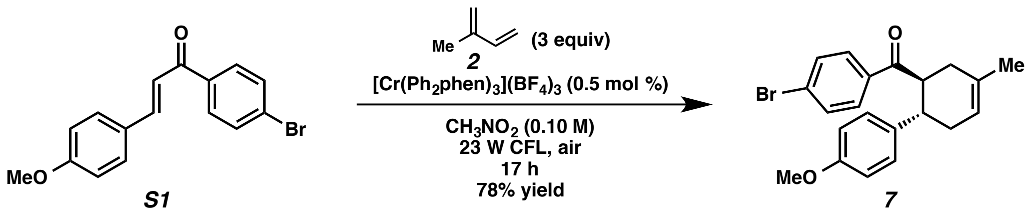

Cyclohexene 7. Prepared according to the *General Procedure* using alkene **S1** (31.7 mg, 0.100 mmol), diene **2** (30.1 µL, 0.300 mmol), $[Cr(Ph_2phen)_3](BF_4)_3$ (0.7 mg, 0.000500 mmol), and nitromethane (1.00 mL). The reaction mixture was irradiated for 17 h. The crude product was purified by flash chromatography (100% hexanes → 6:1 hexanes/EtOAc eluent) to afford cyclohexene **7** (29.9 mg, 78% yield, 13:1 isomeric ratio) as a colorless oil.

TLC: R_f = 0.71 in 3:1 hexanes/EtOAc, stained blue with *p*-anisaldehyde.

^{1}H NMR (400 MHz; $CDCl_3$): δ 7.64 (d, *J* = 8.4 Hz, 2H), 7.49 (d, *J* = 8.4 Hz, 2H), 7.06 (d, *J* = 8.6 Hz, 2H), 6.68 (d, *J* = 8.6 Hz, 2H), 5.53 (br s, 1H), 3.89 (td, *J* = 10.7, 5.3 Hz, 1H), 3.68 (s, 3H), 3.14 (td, *J* = 10.7, 5.5 Hz, 1H), 2.32-2.15 (comp. m, 4H), 1.71 (s, 3H).

^{13}C NMR (100 MHz; $CDCl_3$): δ 202.9, 157.9, 136.2, 136.1, 132.3, 131.7, 129.5, 128.3, 127.8, 121.0, 113.7, 55.1, 47.4, 41.7, 35.1, 34.1, 23.2.

IR (ATR, neat): 2963, 2901, 2832, 1682, 1582, 1512, 1242, 1003, 826, 733 cm^{-1}.

HRMS (ESI+): *m/z* calc'd for $(M + H)^+$ $[C_{21}H_{21}BrO_2 + H]^+$: 385.0798, found 385.0805.

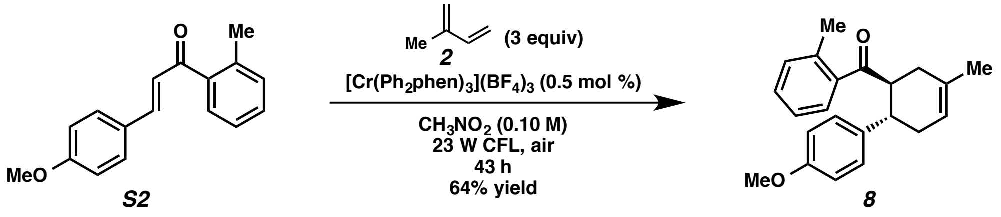

Cyclohexene 8. Prepared according to the *General Procedure* using alkene **S2** (25.2 mg, 0.100 mmol), diene **2** (30.1 μL, 0.300 mmol), $[Cr(Ph_2phen)_3](BF_4)_3$ (0.7 mg, 0.000500 mmol), and nitromethane (1.00 mL). The reaction mixture was irradiated for 43 h. The crude product was purified by flash chromatography (100% hexanes → 6:1 hexanes/EtOAc eluent) to afford cyclohexene **8** (20.6 mg, 64% yield, 8:1 isomeric ratio) as a colorless oil.

TLC: R_f = 0.82 in 3:1 hexanes/EtOAc, visualized by UV.

^{1}H NMR (400 MHz; $CDCl_3$): δ 7.41 (d, *J* = 7.7 Hz, 1H), 7.26 (t, *J* = 7.5 Hz, 1H), 7.16 (t, *J* = 7.5 Hz, 1H), 7.08 (comp. m, 3H), 6.70 (d, *J* = 8.4 Hz, 2H), 5.51 (br s, 1H), 3.78-3.73 (m, 1H), 3.72 (s, 3H), 3.11 (td, *J* = 10.8, 5.7 Hz, 1H), 2.42-2.29 (comp. m, 2H), 2.25-2.18 (comp. m, 2H), 2.05 (s, 3H), 1.73 (s, 3H).

^{13}C NMR (100 MHz; $CDCl_3$): δ 208.2, 158.0, 139.6, 137.6, 136.3, 132.5, 131.4, 130.7, 128.6, 127.4, 125.3, 120.9, 113.7, 55.2, 50.9, 42.2, 34.8, 34.4, 23.3, 20.2.

IR (ATR, neat): 2963, 2909, 2839, 1682, 1512, 1242, 1034, 734 cm^{-1}.

HRMS (ESI+): *m/z* calc'd for $(M + H)^+$ $[C_{22}H_{24}O_2 + H]^+$: 321.1849, found 321.1849.

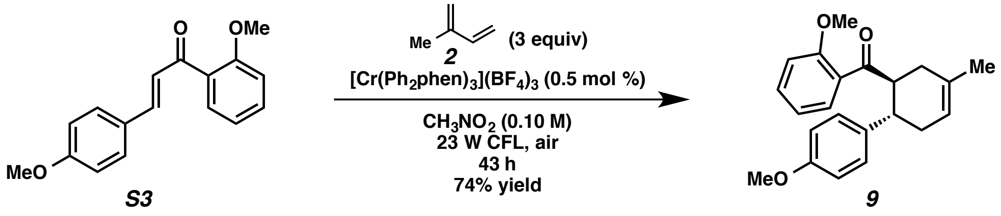

Cyclohexene 9. Prepared according to the *General Procedure* using alkene **S3** (26.8 mg, 0.100 mmol), diene **2** (30.1 μL, 0.300 mmol), [Cr(Ph_2phen)$_3$](BF_4)$_3$ (0.7 mg, 0.000500 mmol), and nitromethane (1.00 mL). The reaction mixture was irradiated for 43 h. The crude product was purified by flash chromatography (100% hexanes → 6:1 hexanes/EtOAc eluent) to afford cyclohexene **9** (24.8 mg, 74% yield, 10:1 isomeric ratio) as a colorless oil.

TLC: R_f = 0.52 in 3:1 hexanes/EtOAc, visualized by UV.

^{1}H NMR (400 MHz; $CDCl_3$): δ 7.29 (td, *J* = 7.8, 0.8 Hz, 1H), 7.03 (d, *J* = 8.6 Hz, 2H), 6.82 (t, *J* = 7.5 Hz, 2H), 6.74 (t, *J* = 7.5 Hz, 1H), 6.67 (d, *J* = 8.6 Hz, 2H), 5.48 (br s, 1H), 3.84 (s, 3H), 3.84-3.80 (m, 1H), 3.71 (s, 3H), 3.04 (td, *J* = 10.5, 5.7 Hz, 1H), 2.39-2.16 (comp. m, 4H), 1.73 (s, 3H).

^{13}C NMR (100 MHz; $CDCl_3$): δ 208.0, 157.8, 157.2, 136.5, 133.1, 132.2, 130.7, 129.3, 128.7, 120.43, 120.40, 113.4, 110.8, 55.5, 55.2, 52.4, 42.5, 34.0, 33.7, 23.4.

IR (ATR, neat): 2963, 2901, 2832, 1682, 1598, 1512, 1242, 1018, 756 cm^{-1}.

HRMS (ESI+): *m/z* calc'd for $(M + H)^+$ $[C_{22}H_{24}O_3 + H]^+$: 337.1798, found 337.1801.

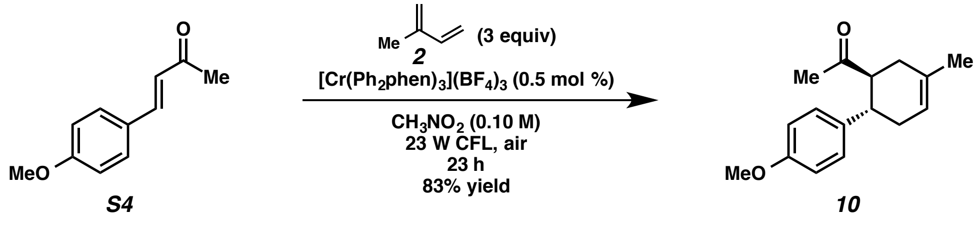

Cyclohexene 10. Prepared according to the *General Procedure* using alkene **S4** (17.6 mg, 0.100 mmol), diene **2** (30.1 μL, 0.300 mmol), [Cr(Ph_2phen)$_3$](BF_4)$_3$ (0.7 mg, 0.000500 mmol), and nitromethane (1.00 mL). The reaction mixture

was irradiated for 23 h. The crude product was purified by flash chromatography (100% hexanes → 9:1 hexanes/EtOAc eluent) to afford cyclohexene **10** (20.3 mg, 83% yield, 12:1 isomeric ratio) as a colorless oil.

TLC: R_f = 0.61 in 3:1 hexanes/EtOAc, stained yellow with *p*-anisaldehyde.

^{1}H NMR (400 MHz; $CDCl_3$): δ 7.09 (d, *J* = 8.6 Hz, 2H), 6.81 (d, *J* = 8.6 Hz, 2H), 5.46 (br s, 1H), 3.76 (s, 3H), 3.00 (td, *J* = 10.9, 5.2 Hz, 1H), 2.87 (td, *J* = 10.9, 5.4 Hz, 1H), 2.29-2.04 (comp. m, 4H), 1.82 (s, 3H), 1.71 (s, 3H).

^{13}C NMR (100 MHz; $CDCl_3$): δ 212.4, 158.1, 136.0, 132.1, 128.4, 120.7, 113.9, 55.2, 53.8, 41.9, 34.1, 33.3, 29.8, 23.2.

IR (ATR, neat): 2963, 2909, 2832, 1705, 1612, 1512, 1435, 1242, 1173, 1034, 826 cm^{-1}.

HRMS (ESI+): *m/z* calc'd for $(M + H)^+$ $[C_{16}H_{20}O_2 + H]^+$: 245.1536, found 245.1538.

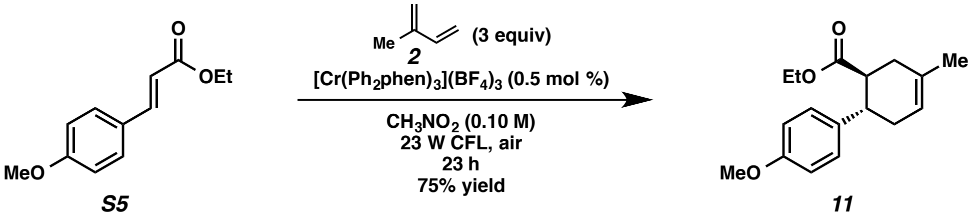

Cyclohexene 11. Prepared according to the *General Procedure* using alkene **S5** (20.6 mg, 0.100 mmol), diene **2** (30.1 μL, 0.300 mmol), $[Cr(Ph_2phen)_3](BF_4)_3$ (0.7 mg, 0.000500 mmol), and nitromethane (1.00 mL). The reaction mixture was irradiated for 23 h. The crude product was purified by flash chromatography (100% hexanes → 6:1 hexanes/EtOAc eluent) to afford cyclohexene **11** (20.6 mg, 75% yield, 19:1 isomeric ratio) as a colorless oil.

TLC: R_f = 0.67 in 3:1 hexanes/EtOAc, visualized by UV.

^{1}H NMR (400 MHz; $CDCl_3$): δ 7.12 (d, *J* = 8.5 Hz, 2H), 6.80 (d, *J* = 8.5 Hz, 2H), 5.46 (br s, 1H), 3.85 (qd, *J* = 7.1, 1.5 Hz, 2H), 3.76 (s, 3H), 2.90 (td, *J* = 10.9, 5.4 Hz, 1H), 2.80 (td, *J* = 10.9, 5.1 Hz, 1H), 2.42-2.16 (comp. m, 4H), 1.71 (s, 3H), 0.93 (t, *J* = 7.1 Hz, 3H).

^{13}C NMR (100 MHz; $CDCl_3$): δ 175.2, 158.1, 136.0, 132.1, 128.5, 120.6, 113.6, 59.9, 55.21, 55.19, 46.9, 42.1, 33.9, 23.2, 13.9.

IR (ATR, neat): 2963, 2901, 2839, 1782, 1512, 1242, 1172, 1034, 826 cm^{-1}.

HRMS (ESI+): *m/z* calc'd for $(M + H)^+$ $[C_{17}H_{22}O_3 + H]^+$: 275.1642, found 275.1640.

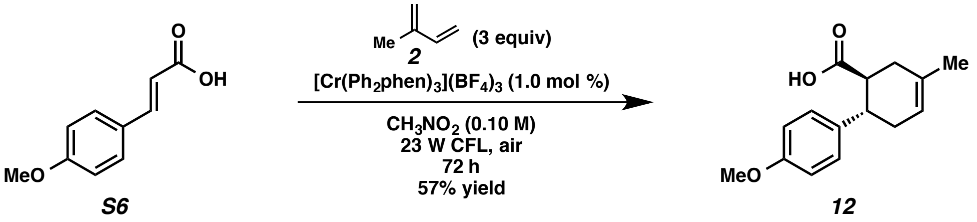

Cyclohexene 12. Prepared according to a modified *General Procedure* (modification: 1 mol % catalyst was used) using alkene **S6** (17.8 mg, 0.100 mmol), diene **2** (30.1 μL, 0.300 mmol), $[Cr(Ph_2phen)_3](BF_4)_3$ (1.3 mg, 0.00100 mmol), and nitromethane (1.00 mL). The reaction mixture was irradiated for 72 h. The crude product was purified by flash chromatography (100% hexanes → 2:1 hexanes/EtOAc eluent) to afford cyclohexene **12** (14.0 mg, 57% yield, 17:1 isomeric ratio) as a white solid.

TLC: R_f = 0.64 in 1:1 hexanes/EtOAc, stained with $KMnO_4$.

^{1}**H NMR** (400 MHz; $CDCl_3$): δ 7.11 (d, *J* = 8.6 Hz, 2H), 6.80 (d, *J* = 8.6 Hz, 2H), 5.45 (br s, 1H), 3.77 (s, 3H), 2.91 (td, *J* = 10.6, 5.4 Hz, 1H), 2.81 (td, *J* = 10.6, 5.4 Hz, 1H), 2.37-2.08 (comp. m, 4H), 1.70 (s, 3H).

^{13}**C NMR** (100 MHz; $CDCl_3$): δ 180.0, 158.1, 135.8, 131.8, 128.3, 120.6, 113.8, 55.2, 46.3, 41.3, 33.64, 33.61, 23.1.

IR (ATR, neat): 3364, 2970, 2909, 2839, 1713, 1512, 1242, 949, 826 cm^{-1}.

HRMS (ESI+): *m/z* calc'd for $(M + H)^+$ $[C_{15}H_{18}O_3 + H]^+$: 247.1329, found 247.1326.

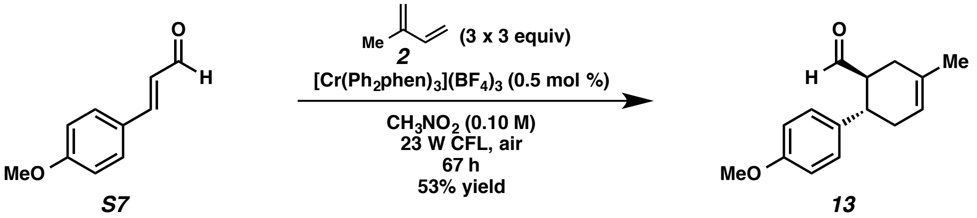

Cyclohexene 13. Prepared according to a modified *General Procedure* (modification: 9 equivalents of diene were used, and it was added in 3 portions) using alkene **S7** (8.1 mg, 50.0 μmol), diene **2** (15.0 μL, 150 μmol),

$[Cr(Ph_2phen)_3](BF_4)_3$ (0.3 mg, 0.250 μmol), and nitromethane (0.500 mL). After 24 and 48 h, additional portions of diene **2** (15.0 μL each) were added. The reaction mixture was irradiated for 67 h total. The crude product was purified by flash chromatography (100% hexanes → 9:1 hexanes/EtOAc eluent) to afford cyclohexene **13** (6.1 mg, 53% yield, 11:1 isomeric ratio) as a colorless oil.

TLC: R_f = 0.63 in 3:1 hexanes/EtOAc, stained blue with *p*-anisaldehyde.

^{1}H NMR (400 MHz; $CDCl_3$): δ 9.45 (s, 1H), 7.13 (d, *J* = 8.6 Hz, 2H), 6.84 (d, *J* = 8.6 Hz, 2H), 5.49 (br s, 1H), 3.78 (s, 3H), 2.98 (td, *J* = 9.8, 5.6 Hz, 1H), 2.82-2.75 (m, 1H), 2.34-2.02 (comp. m, 4H), 1.73 (s, 3H).

^{13}C NMR (100 MHz; $CDCl_3$): δ 204.5, 158.3, 135.3, 131.8, 128.4, 120.9, 114.1, 55.2, 51.9, 39.9, 33.4, 29.5, 23.3.

IR (ATR, neat): 2970, 2909, 2839, 1721, 1512, 1250, 826 cm^{-1}.

HRMS (ESI+): *m/z* calc'd for $(M + H)^+$ $[C_{15}H_{18}O_2 + H]^+$: 231.1380, found 231.1379.

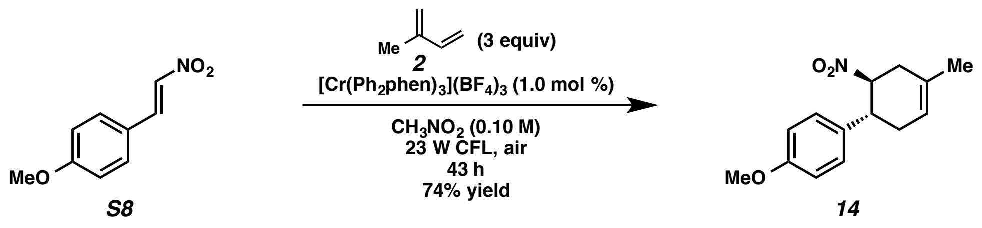

Cyclohexene 14. Prepared according to a modified *General Procedure* (modification: 1 mol % catalyst was used) using alkene **S8** (17.9 mg, 0.100 mmol), diene **2** (30.1 μL, 0.300 mmol), $[Cr(Ph_2phen)_3](BF_4)_3$ (1.3 mg, 0.00100 mmol), and nitromethane (1.00 mL). The reaction mixture was irradiated for 43 h. The crude product was purified by flash chromatography (100% hexanes → 6:1 hexanes/EtOAc eluent) to afford cyclohexene **14** (18.2 mg, 74% yield, 17:1 isomeric ratio) as a pale yellow oil.

TLC: R_f = 0.65 in 3:1 hexanes/EtOAc, visualized by UV.

^{1}H NMR (400 MHz; $CDCl_3$): δ 7.13 (d, *J* = 8.4 Hz, 2H), 6.83 (d, *J* = 8.4 Hz, 2H), 5.49 (br s, 1H), 4.91 (td, *J* = 10.8, 5.6 Hz, 1H), 3.76 (s, 3H), 3.29 (td, *J* = 10.8, 5.8 Hz, 1H), 2.79-2.72 (m, 1H), 2.59-2.52 (m, 1H), 2.44-2.37 (m, 1H), 2.34-2.24 (m, 1H), 1.75 (s, 3H).

^{13}C NMR (100 MHz; $CDCl_3$): δ 158.8, 131.9, 130.3, 128.3, 120.7, 114.2, 88.1, 55.2, 43.4, 35.6, 33.1, 22.8.

IR (ATR, neat): 2963, 2909, 2839, 1551, 1512, 1373, 1242, 1033, 826, 733 cm^{-1}.

HRMS (ESI+): *m/z* calc'd for $(M + H)^+$ $[C_{14}H_{17}NO_3 + H]^+$: 248.1281, found 248.1286.

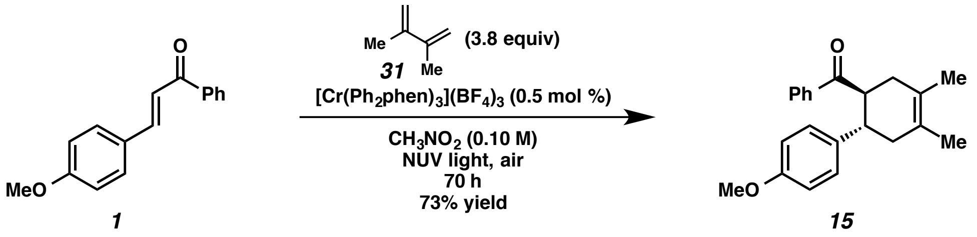

Cyclohexene 15. Prepared according to a modified *General Procedure* (modification: 300-420 nm irradiation was used) using alkene **1** (23.8 mg, 0.100 mmol), diene **31** (43.0 μL, 0.380 mmol), $[Cr(Ph_2phen)_3](BF_4)_3$ (0.7 mg, 0.000500 mmol), and nitromethane (1.00 mL). The reaction mixture was irradiated with 300-420 nm light for 70 h. The crude product was purified by flash chromatography (100% hexanes → 9:1 hexanes/EtOAc eluent) to afford cyclohexene **15** (23.4 mg, 73% yield) as a colorless oil.

TLC: R_f = 0.63 in 3:1 hexanes/EtOAc, stained with $KMnO_4$.

^{1}H NMR (400 MHz; $CDCl_3$): δ 7.81 (d, *J* = 7.4 Hz, 2H), 7.48 (t, *J* = 7.4 Hz, 1H), 7.37 (t, *J* = 7.7 Hz, 2H), 7.10 (d, *J* = 8.6 Hz, 2H), 6.69 (d, *J* = 8.6 Hz, 2H), 3.95 (td, *J* = 10.8, 5.4 Hz, 1H), 3.68 (s, 3H), 3.25-3.22 (m, 1H), 2.31-2.21 (comp. m, 4H), 1.67 (app. s, 6H).

^{13}C NMR (100 MHz; $CDCl_3$): δ 203.7, 157.8, 137.4, 136.7, 132.6, 128.4, 128.3, 128.0, 125.7, 124.1, 113.7, 55.1, 47.7, 42.2, 40.7, 37.0, 18.7, 18.6.

IR (ATR, neat): 2901, 2832, 1674, 1512, 1242, 1034, 825, 733, 694 cm^{-1}.

HRMS (ESI+): *m/z* calc'd for $(M + H)^+$ $[C_{22}H_{24}O_2 + H]^+$: 321.1849, found 321.1855

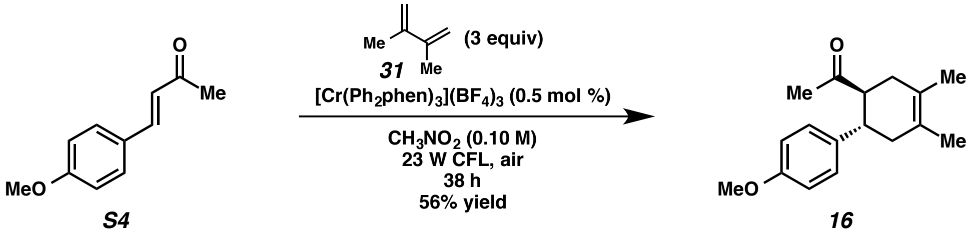

Cyclohexene 16. Prepared according the *General Procedure* using alkene **S4** (15.4 mg, 0.0873 mmol), diene **31** (29.0 μL, 0.262 mmol), $[Cr(Ph_2phen)_3](BF_4)_3$ (0.6 mg, 0.000437 mmol), and nitromethane (0.870 mL). The reaction mixture was irradiated for 38 h. The crude product was purified by flash chromatography (100% hexanes → 10:1 hexanes/EtOAc eluent) to afford cyclohexene **16** (12.6 mg, 56% yield) as a colorless oil.

TLC: R_f = 0.65 in 3:1 hexanes/EtOAc, stained with $KMnO_4$.

^{1}H NMR (400 MHz; $CDCl_3$): δ 7.10 (d, *J* = 8.7 Hz, 2H), 6.82 (d, *J* = 8.7 Hz, 2H), 3.77 (s, 3H), 3.01-2.89 (comp. m, 2H), 2.32-2.03 (comp. m, 4H), 1.84 (s, 3H), 1.67 (s, 3H), 1.63 (s, 3H).

^{13}C NMR (100 MHz; $CDCl_3$): δ 212.3, 158.1, 136.1, 128.3, 125.4, 123.7, 113.9, 55.2, 54.2, 42.7, 40.6, 35.0, 29.6, 18.7, 18.6.

IR (ATR, neat): 2991, 2911, 1705, 1512, 1244, 1176, 1033, 827 cm^{-1}.

HRMS (ESI+): *m/z* calc'd for $(M + H)^+$ $[C_{17}H_{22}O_2 + H]^+$: 259.1693, found 259.1689.

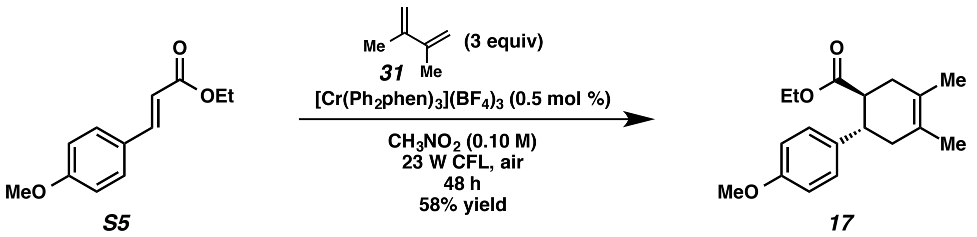

Cyclohexene 17. Prepared according the *General Procedure* using alkene **S5** (20.6 mg, 0.100 mmol), diene **31** (33.8 μL, 0.300 mmol), $[Cr(Ph_2phen)_3](BF_4)_3$ (0.7 mg, 0.000500 mmol), and nitromethane (1.00 mL). The reaction mixture was irradiated for 48 h. The crude product was purified by flash chromatography (100% hexanes → 9:1 hexanes/EtOAc eluent) to afford cyclohexene **17** (16.6 mg, 58% yield) as a colorless oil.

TLC: R_f = 0.73 in 3:1 hexanes/EtOAc, stained with $KMnO_4$.

^{1}H NMR (400 MHz; $CDCl_3$): δ 7.13 (d, *J* = 8.7 Hz, 2H), 6.81 (d, *J* = 8.7 Hz, 2H), 3.86 (qd, *J* = 7.1, 2.5 Hz, 2H), 3.77 (s, 3H), 2.99-2.92 (m, 1H), 2.78 (td, *J* = 11.2, 5.3 Hz, 1H), 2.45-2.38 (m, 1H), 2.23-2.15 (comp. m, 3H), 1.66 (s, 3H), 1.63 (s, 3H), 0.94 (t, *J* = 7.1 Hz, 3H).

^{13}C NMR (100 MHz; $CDCl_3$): δ 175.2, 158.1, 136.1, 128.4, 125.3, 123.7, 113.6, 59.9, 55.2, 47.2, 42.9, 40.3, 35.5, 18.7, 18.6, 13.9.

IR (ATR, neat): 2980, 2904, 1728, 1512, 1244, 1176, 1153, 1033, 829 cm^{-1}.

HRMS (ESI+): *m/z* calc'd for $(M + H)^+$ $[C_{18}H_{24}O_3 + H]^+$: 289.1798, found 289.1799.

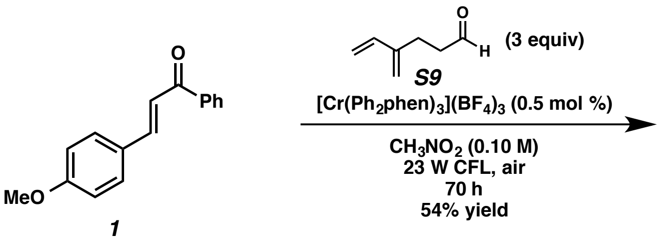

Cyclohexene 18. Prepared according to the *General Procedure* using alkene **1** (17.9 mg, 75.0 μmol), diene **S9** (24.8 mg, 225 μmol), $[Cr(Ph_2phen)_3](BF_4)_3$ (0.5 mg, 0.375 μmol), and nitromethane (0.750 mL). The reaction mixture was irradiated for 70 h. The crude product was purified by flash chromatography (100% hexanes → 3:1 hexanes/EtOAc eluent) to afford cyclohexene **18** (14.1 mg, 54% yield, 6:1 isomeric ratio) as a colorless oil.

TLC: R_f = 0.35 in 3:1 hexanes/EtOAc, stained blue with *p*-anisaldehyde.

^{1}H NMR (400 MHz; $CDCl_3$): δ 9.79 (s, 1H), 7.79 (d, J = 7.3 Hz, 2H), 7.48 (t, J = 7.3 Hz, 1H), 7.37 (t, J = 7.7 Hz, 2H), 7.09 (d, J = 8.6 Hz, 2H), 6.68 (d, J = 8.6 Hz, 2H), 5.57 (br s, 1H), 3.95 (td, J = 10.5, 5.2 Hz, 1H), 3.68 (s, 3H), 3.21-3.15 (m, 1H), 2.60-2.56 (comp. m, 2H), 2.37-2.19 (comp. m, 6H).

^{13}C NMR (100 MHz; $CDCl_3$): δ 203.6, 202.1, 157.9, 137.2, 136.1, 134.3, 132.8, 128.5, 128.4, 128.0, 121.5, 113.7, 55.1, 47.0, 41.7, 41.6, 33.9, 33.5, 29.4.

IR (ATR, neat): 2901, 2832, 2723, 1721, 1674, 1512, 1242, 1034, 826, 733 cm^{-1}.

HRMS (ESI+): *m/z* calc'd for $(M + H)^+$ $[C_{23}H_{24}O_3 + H]^+$: 349.1798, found 349.1796.

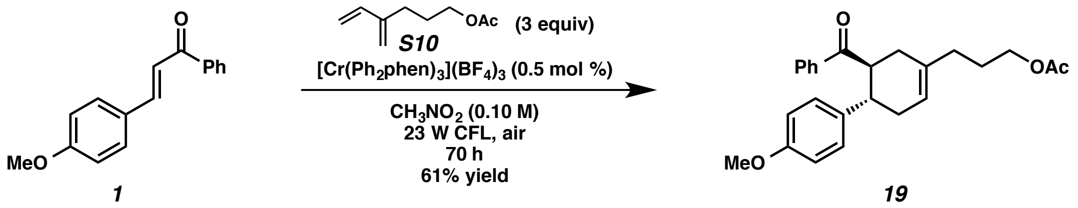

Cyclohexene 19. Prepared according to the *General Procedure* using alkene **1** (17.9 mg, 75.0 μmol), diene **S10** (34.7 mg, 225 μmol), $[Cr(Ph_2phen)_3](BF_4)_3$ (0.5 mg, 0.375 μmol), and nitromethane (0.750 mL). The reaction mixture was irradiated for 70 h. The crude product was purified by flash chromatography (100% hexanes → 3:1 hexanes/EtOAc eluent) to afford cyclohexene **19** (18.0 mg, 61% yield, 7:1 isomeric ratio) as a colorless oil.

TLC: R_f = 0.50 in 3:1 hexanes/EtOAc, visualized by UV.

^{1}H NMR (400 MHz; $CDCl_3$): δ 7.79 (d, J = 7.3 Hz, 2H), 7.48 (t, J = 7.3 Hz, 1H), 7.37 (t, J = 7.7 Hz, 2H), 7.09 (d, J = 8.6 Hz, 2H), 6.69 (d, J = 8.6 Hz, 2H), 5.57 (br s, 1H), 4.07 (t, 2H, J = 6.4 Hz), 3.98-3.92 (m, 1H), 3.68 (s, 3H), 3.18 (td, J = 10.8, 5.7 Hz, 1H), 2.41-2.20 (comp. m, 4H), 2.09-2.05 (comp. m, 2H), 2.05 (s, 3H), 1.82-1.74 (comp. m, 2H).

^{13}C NMR (100 MHz; $CDCl_3$): δ 203.7, 171.2, 157.8, 137.2, 136.3, 135.0, 132.8, 128.5, 128.34, 128.28, 128.0, 121.3, 113.7, 64.1, 55.1, 47.2, 41.6, 34.1, 33.5, 26.4, 21.0.

IR (ATR, neat): 2955, 2901, 2832, 1728, 1674, 1512, 1234, 1034, 826, 702 cm^{-1}.

HRMS (ESI+): *m/z* calc'd for $(M + H)^+$ $[C_{25}H_{28}O_4 + H]^+$: 393.2060, found 393.2065.

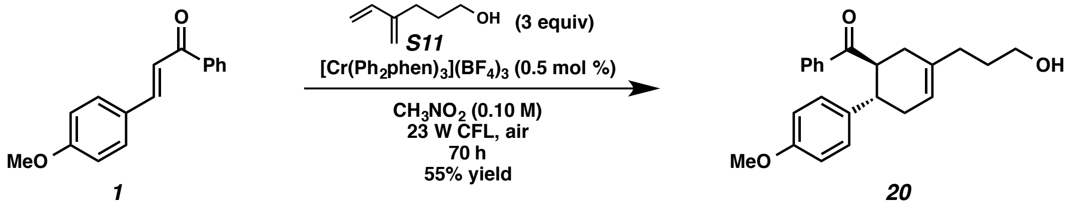

Cyclohexene 20. Prepared according to the *General Procedure* using alkene **1** (17.9 mg, 75.0 μmol), diene **S11** (25.2 mg, 225 μmol), $[Cr(Ph_2phen)_3](BF_4)_3$ (0.5 mg, 0.375 μmol), and nitromethane (0.750 mL). The reaction mixture was

irradiated for 70 h. The crude product was purified by flash chromatography (100% hexanes → 4:1 hexanes/EtOAc eluent) to afford cyclohexene **20** (14.5 mg, 55% yield, 8:1 isomeric ratio) as a colorless oil.

TLC: R_f = 0.62 in 1:1 hexanes/EtOAc, stained blue with *p*-anisaldehyde.

^{1}H NMR (400 MHz; $CDCl_3$): δ 7.79 (d, *J* = 8.0 Hz, 2H), 7.48 (t, *J* = 7.4 Hz, 1H), 7.37 (t, *J* = 7.6 Hz, 2H), 7.09 (d, *J* = 8.4 Hz, 2H), 6.69 (d, *J* = 8.4 Hz, 2H), 5.59 (br s, 1H), 3.95 (td, *J* = 10.4, 5.1 Hz, 1H), 3.68 (s, 3H), 3.22-3.15 (m, 1H), 2.41-2.21 (comp. m, 4H), 2.12-2.08 (comp. m, 2H), 1.75-1.65 (comp. m, 4H).

^{13}C NMR (100 MHz; $CDCl_3$): δ 203.8, 157.8, 136.4, 135.7, 132.8, 128.5, 128.3, 128.0, 121.0, 113.7, 62.6, 55.1, 47.2, 41.6, 34.1, 33.6, 33.5, 30.5.

IR (ATR, neat): 3387, 2931, 2909, 2839, 1674, 1512, 1265, 1242, 1034, 732, 702 cm^{-1}.

HRMS (ESI+): *m/z* calc'd for $(M + H)^+$ $[C_{23}H_{26}O_3 + H]^+$: 351.1955, found 351.1955.

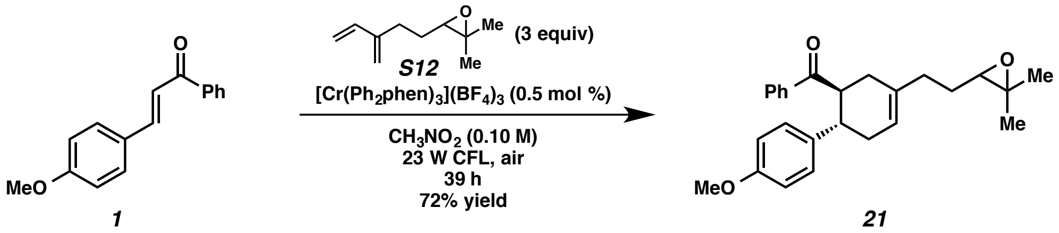

Cyclohexene 21. Prepared according to the *General Procedure* using alkene **1** (23.8 mg, 0.100 mmol), diene **S12** (45.7 mg, 0.300 mmol), $[Cr(Ph_2phen)_3](BF_4)_3$ (0.7 mg, 0.000500 mmol), and nitromethane (1.00 mL). The reaction mixture was irradiated for 39 h. The crude product was purified by flash chromatography (100% hexanes → 9:1 hexanes/EtOAc eluent) to afford cyclohexene **21** (28.2 mg, 72% yield, 7:1 isomeric ratio) as a colorless oil.

TLC: R_f = 0.52 in 3:1 hexanes/EtOAc, stained yellow with *p*-anisaldehyde.

^{1}H NMR (400 MHz; $CDCl_3$): δ 7.81-7.78 (comp. m, 2H), 7.48 (t, *J* = 7.3 Hz, 1H), 7.37 (t, *J* = 7.6 Hz, 2H), 7.09 (d, *J* = 8.5 Hz, 2H), 6.69 (d, *J* = 8.5 Hz, 2H), 5.61 (br s, 1H), 3.99-3.92 (m, 1H), 3.68 (s, 3H), 3.23-3.16 (m, 1H), 2.73 (q, *J* = 5.4 Hz, 1H), 2.44-2.10 (comp. m, 6H), 1.68-1.63 (comp. m, 2H), 1.32 (s, 3H), 1.27 (d, *J* = 1.4 Hz, 3H).

^{13}C NMR (100 MHz; $CDCl_3$): δ 203.7, 157.8, 137.2, 136.3, 135.3, 132.8, 128.4, 128.0, 121.0, 113.7, 64.0, 58.4, 55.1, 47.1, 41.6, 34.2, 34.0, 27.3, 24.9, 18.8.

IR (ATR, neat): 3055, 2963, 2909, 2839, 1682, 1512, 1250, 734, 702 cm^{-1}.

HRMS (ESI+): *m/z* calc'd for $(M + H)^+$ $[C_{26}H_{30}O_3 + H]^+$: 391.2268, found 391.2267.

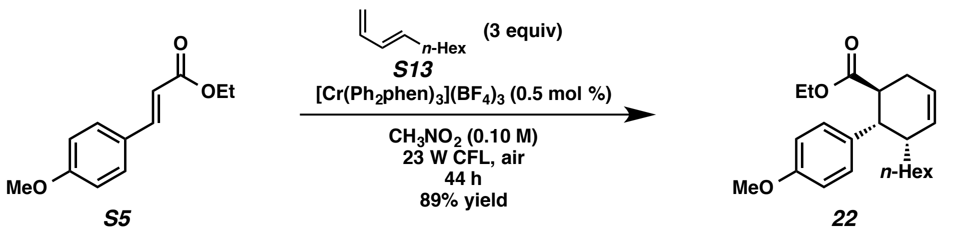

Cyclohexene 22. Prepared according to the *General Procedure* using alkene **S5** (15.5 mg, 75.0 μmol), diene **S13**[4] (31.1 mg, 225 μmol), $[Cr(Ph_2phen)_3](BF_4)_3$ (0.5 mg, 0.375 μmol), and nitromethane (0.750 mL). The reaction mixture was irradiated for 44 h. The crude product was purified by flash chromatography (100% hexanes → 9:1 hexanes/EtOAc eluent) to afford cyclohexene **22** (22.9 mg, 89% yield, 5:1 isomeric ratio) as a colorless oil.

TLC: R_f = 0.76 in 3:1 hexanes/EtOAc, stained blue with *p*-anisaldehyde.

^{1}H NMR (400 MHz; $CDCl_3$): δ 7.11 (d, *J* = 8.5 Hz, 2H), 6.80 (d, *J* = 8.5 Hz, 2H), 5.89-5.86 (m, 1H), 5.72-5.69 (m, 1H), 3.96 (q, *J* = 7.1 Hz, 2H), 3.77 (s, 3H), 3.29 (dd, *J* = 10.4, 5.4 Hz, 1H), 3.00 (dt, *J* = 10.4, 7.6 Hz, 1H), 2.38-2.36 (comp. m, 2H), 2.24-2.21 (m, 1H), 1.27-1.09 (comp. m, 10H), 1.04 (t, *J* = 7.1 Hz, 3H), 0.81 (t, *J* = 7.1 Hz, 3H).

^{13}C NMR (100 MHz; $CDCl_3$): δ 175.5, 157.8, 134.2, 131.5, 129.4, 124.1, 113.3, 60.1, 55.1, 44.9, 41.2, 39.0, 31.7, 31.4, 29.4, 28.9, 27.3, 22.5, 14.04, 14.01.

IR (ATR, neat): 2955, 2924, 2855, 1728, 1512, 1242, 1242, 1173, 1034, 833 cm^{-1}.

HRMS (ESI+): *m/z* calc'd for $(M + H)^+$ $[C_{22}H_{32}O_3 + H]^+$: 345.2424, found 345.2426.

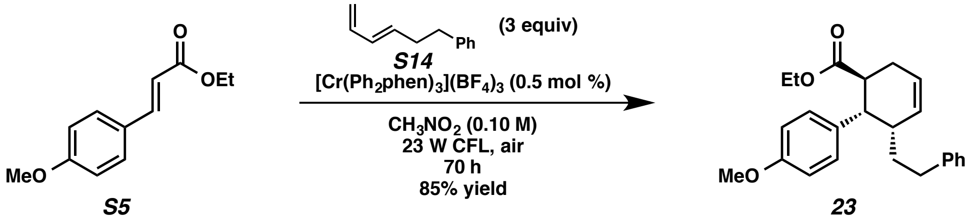

Cyclohexene 23. Prepared according to the *General Procedure* using alkene **S5** (15.5 mg, 75.0 µmol), diene **S14**[3] (35.6 mg, 225 µmol), $[Cr(Ph_2phen)_3](BF_4)_3$ (0.5 mg, 0.375 µmol), and nitromethane (0.750 mL). The reaction mixture was irradiated for 70 h. The crude product was purified by flash chromatography (100% hexanes → 15:1 hexanes/EtOAc eluent) to afford cyclohexene **23** (23.1 mg, 85% yield, 4:1 isomeric ratio) as a colorless oil.

TLC: R_f = 0.74 in 3:1 hexanes/EtOAc, stained red with *p*-anisaldehyde.

^{1}H NMR (400 MHz; $CDCl_3$): δ 7.20 (t, *J* = 7.2 Hz, 3H), 7.13 (d, *J* = 8.4 Hz, 2H), 6.99 (d, *J* = 7.6 Hz, 2H), 6.81 (d, *J* = 8.4 Hz, 2H), 5.94-5.90 (m, 1H), 5.81-5.75 (m, 1H), 3.97 (q, *J* = 7.1 Hz, 2H), 3.78 (s, 3H), 3.35 (dd, *J* = 10.2, 5.4 Hz, 1H), 3.01 (dt, *J* = 10.2, 7.5 Hz, 1H), 2.42-2.39 (comp. m, 3H), 2.35-2.27 (comp. m, 2H), 1.54-1.41 (comp. m, 2H), 1.04 (t, *J* = 7.1 Hz, 3H).

^{13}C NMR (100 MHz; $CDCl_3$): δ 175.4, 157.9, 142.3, 133.9, 131.0, 129.4, 128.24, 128.19, 125.6, 124.7, 113.5, 60.2, 55.2, 44.7, 41.3, 38.5, 33.6, 33.2, 28.6, 14.0.

IR (ATR, neat): 3024, 2932, 2839, 1728, 1512, 1242, 1157, 1034, 833, 733, 702 cm^{-1}.

HRMS (ESI+): *m/z* calc'd for $(M + H)^+$ $[C_{24}H_{28}O_3 + H]^+$: 365.2111, found 365.2109.

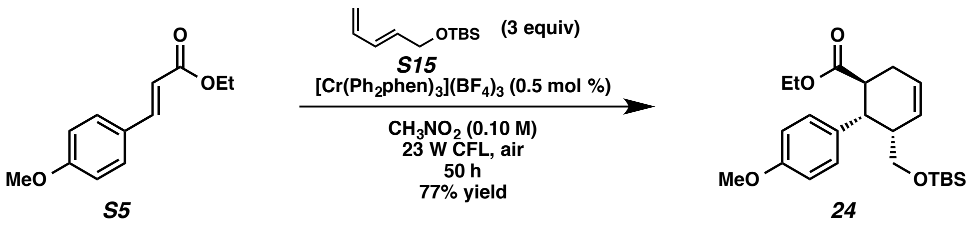

Cyclohexene 24. Prepared according to the *General Procedure* using alkene **S5** (24.7 mg, 0.120 mmol), diene **S15** (71.4 mg, 0.360 mmol), $[Cr(Ph_2phen)_3](BF_4)_3$ (0.8 mg, 0.000600 mmol), and nitromethane (1.20 mL). The reaction mixture was irradiated for 50 h. The crude product was purified by flash chromatography (100% hexanes → 15:1 hexanes/EtOAc eluent) to afford cyclohexene **24** (37.6 mg, 77% yield, 4:1 isomeric ratio) as a colorless oil.

TLC: R_f = 0.76 in 3:1 hexanes/EtOAc, stained red with *p*-anisaldehyde.

^{1}H NMR (400 MHz; $CDCl_3$): δ 7.18 (d, *J* = 8.5 Hz, 2H), 6.80 (d, *J* = 8.5 Hz, 2H), 5.86-5.75 (comp. m, 2H), 3.93 (q, *J* = 7.1 Hz, 2H), 3.78 (s, 3H), 3.35 (d, *J* = 4.4 Hz, 2H), 3.32-3.26 (m, 1H), 2.36-2.32 (comp. m, 3H), 1.01 (t, *J* = 7.1 Hz, 3H), 0.86 (s, 9H), -0.05 (s, 3H), -0.08 (s, 3H).

^{13}C NMR (100 MHz; $CDCl_3$): δ 175.8, 158.0, 133.7, 129.4, 129.0, 126.0, 113.3, 63.3, 60.0, 55.2, 44.0, 41.82, 41.77, 28.9, 25.8, 18.2, 14.0, -5.6.

IR (ATR, neat): 2932, 2901, 2855, 1728, 1512, 1250, 1042, 833, 772 cm^{-1}.

HRMS (ESI+): *m/z* calc'd for $(M + H)^+$ $[C_{23}H_{36}O_4Si + H]^+$: 405.2456, found 405.2455.

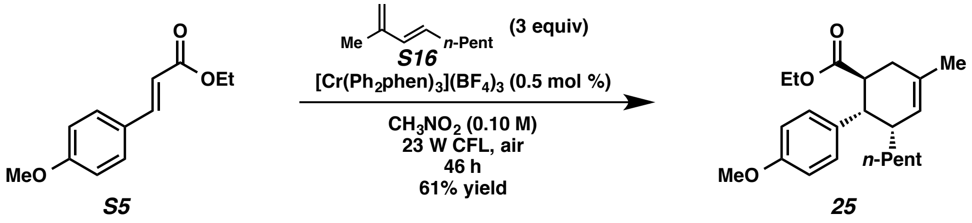

Cyclohexene 25. Prepared according to the *General Procedure* using alkene **S5** (15.5 mg, 75.0 μmol), diene **S16**[3] (31.1 mg, 225 μmol), $[Cr(Ph_2phen)_3](BF_4)_3$ (0.5 mg, 0.375 μmol), and nitromethane (0.750 mL). The reaction mixture was irradiated for 46 h. The crude product was purified by flash chromatography (100% hexanes → 9:1 hexanes/EtOAc eluent) to afford cyclohexene **25** (15.8 mg, 61% yield, 3:1 isomeric ratio) as a colorless oil.

TLC: R_f = 0.78 in 3:1 hexanes/EtOAc, stained blue with *p*-anisaldehyde.

^{1}H NMR (400 MHz; $CDCl_3$): δ 7.09 (d, *J* = 8.5 Hz, 2H), 6.79 (d, *J* = 8.5 Hz, 2H), 5.57 (br s, 1H), 3.97 (q, *J* = 7.1 Hz, 2H), 3.77 (s, 3H), 3.24 (dd, *J* = 10.3, 5.4 Hz, 1H), 3.04-2.98 (m, 1H), 2.29-2.16 (comp. m, 3H), 1.72 (s, 3H), 1.36-1.14 (comp. m, 8H), 1.05 (t, *J* = 7.1 Hz, 3H), 0.79 (t, *J* = 7.0 Hz, 3H).

^{13}C NMR (100 MHz; $CDCl_3$): δ 175.6, 157.8, 134.2, 131.3, 129.4, 125.7, 113.3, 60.1, 55.1, 47.7, 44.9, 41.7, 39.1, 33.3, 32.0, 31.4, 27.1, 23.4, 22.6, 14.0.

IR (ATR, neat): 2955, 2924, 2855, 1736, 1512, 1466, 1250, 1180, 1034, 833 cm^{-1}.

HRMS (ESI+): *m/z* calc'd for $(M + H)^+$ $[C_{22}H_{32}O_3 + H]^+$: 345.2424, found 345.2423.

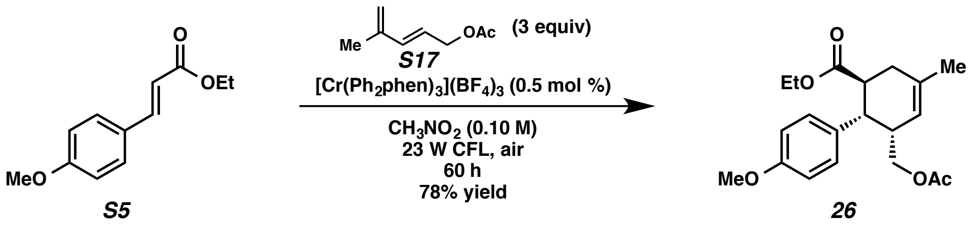

Cyclohexene 26. Prepared according to the *General Procedure* using alkene **S5** (15.5 mg, 75.0 μmol), diene **S17** (31.5 mg, 225 μmol), $[Cr(Ph_2phen)_3](BF_4)_3$ (0.5 mg, 0.375 μmol), and nitromethane (0.750 mL). The reaction mixture was irradiated for 60 h. The crude product was purified by flash chromatography (100% hexanes → 10:1 hexanes/EtOAc eluent) to afford cyclohexene **26** (20.2 mg, 78% yield, 6:1 isomeric ratio) as a colorless oil.

TLC: R_f = 0.55 in 3:1 hexanes/EtOAc, visualized by UV.

^{1}H NMR (400 MHz; $CDCl_3$): δ 7.10 (d, *J* = 8.6 Hz, 2H), 6.80 (d, *J* = 8.6 Hz, 2H), 5.45 (br s, 1H), 3.97 (q, *J* = 7.1 Hz, 2H), 3.90 (dd, *J* = 11.0, 5.3 Hz, 1H), 3.76 (s, 3H), 3.67 (dd, *J* = 11.0, 6.4 Hz, 1H), 3.31 (dd, *J* = 10.8, 6.0 Hz, 1H), 3.12 (ddd, *J* = 10.8, 8.8, 6.3 Hz, 1H), 2.63-2.57 (m, 1H), 2.32-2.28 (comp. m, 2H), 1.91 (s, 3H), 1.74 (s, 3H), 1.04 (t, *J* = 7.1 Hz, 3H).

^{13}C NMR (100 MHz; $CDCl_3$): δ 175.2, 169.6, 158.2, 134.2, 132.9, 129.0, 121.6, 113.7, 65.1, 60.3, 55.1, 43.1, 41.8, 39.0, 33.3, 23.3, 20.9, 14.0.

IR (ATR, neat): 2963, 2901, 2839, 1728, 1512, 1247, 1172, 1034, 833 cm^{-1}.

HRMS (ESI+): *m/z* calc'd for $(M + H)^+$ $[C_{20}H_{26}O_5 + H]^+$: 347.1853, found 347.1853.

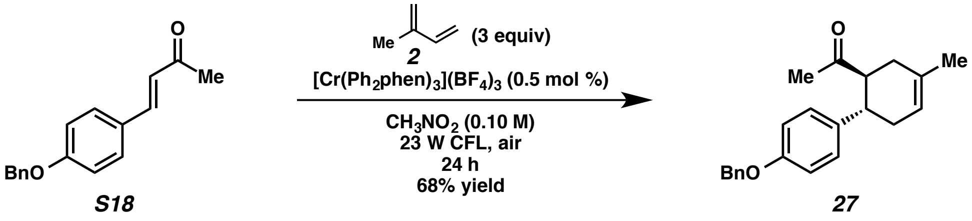

Cyclohexene 27. Prepared according to the *General Procedure* using alkene **S18** (25.2 mg, 0.100 mmol), diene **2** (30.1 μL, 0.300 mmol), $[Cr(Ph_2phen)_3](BF_4)_3$ (0.7 mg, 0.000500 mmol), and nitromethane (1.00 mL). The reaction

mixture was irradiated for 24 h. The crude product was purified by flash chromatography (100% hexanes → 6:1 hexanes/EtOAc eluent) to afford cyclohexene **27** (21.9 mg, 68% yield, 15:1 isomeric ratio) as a white solid.

TLC: R_f = 0.68 in 3:1 hexanes/EtOAc, stained yellow with *p*-anisaldehyde.

^{1}H NMR (400 MHz; $CDCl_3$): δ 7.42-7.29 (comp. m, 5H), 7.10 (d, *J* = 8.6 Hz, 2H), 6.89 (d, *J* = 8.6 Hz, 2H), 5.46 (br s, 1H), 5.01 (s, 2H), 3.00 (td, *J* = 10.9, 5.3 Hz, 1H), 2.88 (td, *J* = 10.9, 5.4 Hz, 1H), 2.29-2.05 (comp. m, 4H), 1.83 (s, 3H), 1.71 (s, 3H).

^{13}C NMR (100 MHz; $CDCl_3$): δ 212.4, 157.4, 137.0, 136.3, 132.2, 128.6, 128.4, 127.9, 127.5, 120.71, 120.70, 114.9, 70.0, 53.8, 42.0, 34.1, 33.3, 23.2.

IR (ATR, neat): 2970, 2909, 2839, 1705, 1512, 1242, 903, 725 cm^{-1}.

HRMS (ESI+): *m/z* calc'd for $(M + H)^+$ $[C_{22}H_{24}O_2 + H]^+$: 321.1849, found 321.1853.

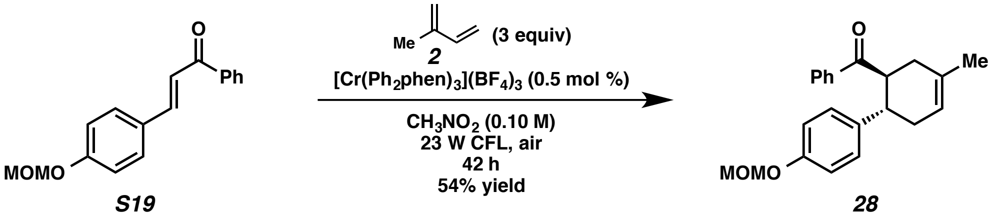

Cyclohexene 28. Prepared according to the *General Procedure* using alkene **S19** (26.8 mg, 0.100 mmol), diene **2** (30.1 µL, 0.300 mmol), $[Cr(Ph_2phen)_3](BF_4)_3$ (0.7 mg, 0.000500 mmol), and nitromethane (1.00 mL). The reaction mixture was irradiated for 42 h. The crude product was purified by flash chromatography (100% hexanes → 6:1 hexanes/EtOAc eluent) to afford cyclohexene **28** (18.1 mg, 54% yield, 12:1 isomeric ratio) as a colorless oil.

TLC: R_f = 0.63 in 3:1 hexanes/EtOAc, stained blue with *p*-anisaldehyde.

^{1}H NMR (400 MHz; $CDCl_3$): δ 7.80 (d, *J* = 7.6 Hz, 2H), 7.47 (t, *J* = 7.6 Hz, 1H), 7.37 (t, *J* = 7.6 Hz, 2H), 7.09 (d, *J* = 8.5 Hz, 2H), 6.81 (d, *J* = 8.5 Hz, 2H), 5.52 (br s, 1H), 5.07-5.02 (comp. m, 2H), 3.97 (td, *J* = 10.5, 5.4 Hz, 1H), 3.39 (s, 3H), 3.18 (td, *J* = 10.5, 5.7 Hz, 1H), 2.38-2.19 (comp. m, 4H), 1.71 (s, 3H).

^{13}C NMR (100 MHz; $CDCl_3$): δ 203.7, 155.5, 137.9, 137.3, 132.7, 132.4, 128.42, 128.38, 128.0, 120.9, 116.1, 94.4, 55.9, 47.2, 41.5, 35.2, 34.2, 23.2.

IR (ATR, neat): 2970, 2901, 2839, 1682, 1512, 1234, 1150, 1003, 903, 725 cm^{-1}.

HRMS (ESI+): *m/z* calc'd for $(M + H)^+$ $[C_{22}H_{24}O_3 + H]^+$: 337.1798, found 337.1799.

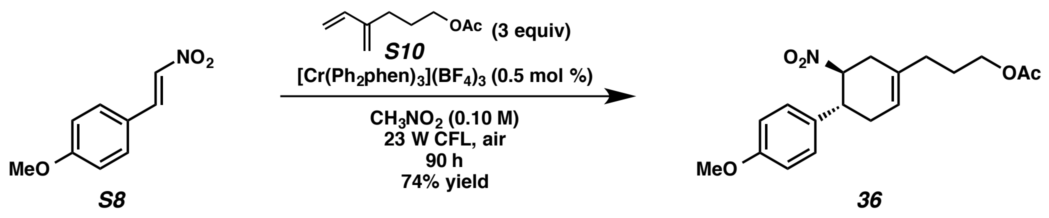

Cyclohexene 36. Prepared according to the *General Procedure* using alkene **S8** (9.0 mg, 50.0 µmol), diene **S10** (23.1 mg, 150 µmol), $[Cr(Ph_2phen)_3](BF_4)_3$ (0.5 mg, 0.250 µmol), and nitromethane (0.500 mL). The reaction mixture was irradiated for 90 h. The crude product was purified by flash chromatography (100% hexanes → 4:1 hexanes/EtOAc eluent) to afford cyclohexene **36** (12.4 mg, 74% yield, 11:1 isomeric ratio) as a yellow oil.

TLC: $R_f = 0.57$ in 3:1 hexanes/EtOAc, stained with $KMnO_4$.

^{1}H NMR (500 MHz; $CDCl_3$): δ 7.16 (d, *J* = 8.6 Hz, 2H), 6.87 (d, *J* = 8.6 Hz, 2H), 5.56 (br s, 1H), 4.93 (td, *J* = 10.6, 5.7 Hz, 1H), 4.11 (t, *J* = 13.2, 6.6 Hz, 2H), 3.80 (s, 3H), 3.34 (td, *J* = 10.6, 5.9 Hz, 1H), 2.83-2.78 (comp. m, 2H), 2.61 (dd, *J* = 16.6, 5.2 Hz, 1H), 2.52-2.46 (m, 1H), 2.37-2.31 (m, 1H), 2.15 (t, *J* = 7.5 Hz, 2H), 2.10 (s, 3H), 2.08-2.07 (m, 1H), 1.84-1.78 (comp. m, 2H).

^{13}C NMR (100 MHz; $CDCl_3$): δ 171.2, 158.9, 132.9, 128.4, 127.9, 121.0, 114.2, 88.0, 63.81, 63.79, 55.2, 43.5, 34.0, 33.1, 32.9, 26.3, 21.0.

IR (ATR, neat): 2918, 2839, 1732, 1548, 1514, 1375, 1244, 1032, 829, 733 cm^{-1}.

HRMS (ESI+): *m/z* calc'd for $(M + Na)^+$ $[C_{18}H_{23}NO_5 + Na]^+$: 356.1468, found 356.1468.

Optimization Table

Table S1. Selected optimization experiments.

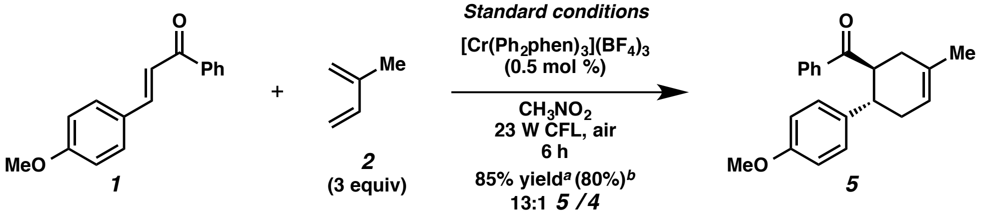

Deviation from standard conditions	*Result*[a]
1 mol % cat., 10 equiv diene	81% yield
1 mol % cat., 10 equiv diene, NUV	70% yield
1 mol % cat., 10 equiv diene, blue LEDs	75% yield
1 mol % cat., 10 equiv diene, sunlight	54% yield
no light	no reaction
no $[Cr(Ph_2phen)_3](BF_4)_3$, 10 equiv diene	0% yield, 20% yield *6*
under Ar, 10 equiv diene, 19 h	31% yield, 26% yield *6*

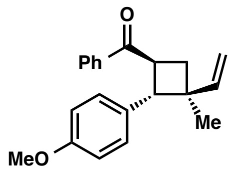

6

[a] Yields determined by ^1H NMR with dodecyl acetate as an internal standard.
[b] Isolated yield. Irradiation time was 16 h.
NUV = 419, 350, 300 nm light

General Procedure for Deviations from Standard Conditions: A flame-dried 2-dram borosilicate vial open to air was charged with alkene **1** (11.9 mg, 0.0500 mmol), diene **2** (50.1 μL, 0.500 mmol), catalyst, nitromethane (0.500 mL), and dodecyl acetate (internal standard, 11.4 mg, 0.0500 mmol). The vial was then capped and placed in front of the indicated light source. The solution was irradiated with stirring for 6 h, then passed through a short plug of silica (2.0-2.5 x 1 cm, Et_2O eluent). The volatile materials were removed by rotary evaporation, and the resulting crude product mixture was analyzed by ^1H NMR.

Irradiation with a 23 W compact fluorescent light bulb was performed in a sealed box lined with aluminum foil (EcoSmart 23 W bright white CFL spiral light bulb, 1600 lumens). Reactions under NUV irradiation were performed in a Luzchem chamber reactor equipped with 10 lamps of wavelengths 419, 350, and 300 nm. Irradiation with blue LEDs was performed in a covered 1-L beaker lined with aluminum foil with the blue LED strand coiled around the inside of the beaker. Irradiation with sunlight was performed by placing the reaction vessel on a stir plate in front of a window on a sunny day. The reaction with no light was performed by wrapping the reaction vessel in aluminum foil.

The reaction under Ar was performed in a 50-mL Schlenk flask; the reaction components were combined open to air, then the entire reaction mixture was degassed by three freeze-pump-thaw cycles.

Catalyst Evaluation

Table S2. Catalyst comparison.

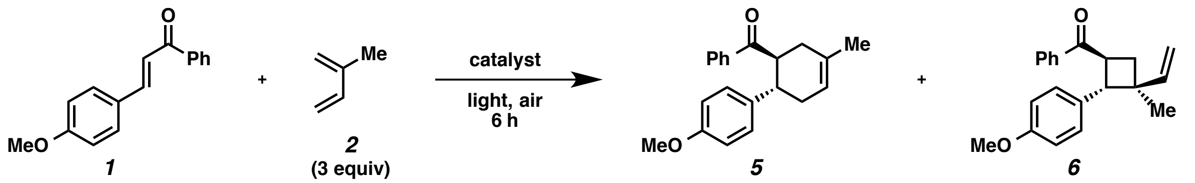

Entry	Catalyst (mol %)	$E_{1/2}$* (V vs. SCE)	Solvent	Light source	Yield (%)[b] 5	6	Yield (%)[b] 6 → 5
1	$[Cr(Ph_2phen)_3](BF_4)_3$ (0.5)	+1.33	CH_3NO_2	23 W CFL	85	0	67[c]
2	$[Cr(phen)_3](OTf)_3$ (0.5)	+1.45	CH_3NO_2	23 W CFL	52[d]	6	79
3	$[(phen)_2Cr(dmcbpy)](OTf)_3$ (0.5)	+1.68	CH_3NO_2	23 W CFL	26[d]	12	82
4	$[Cr(dmcbpy)_3](BF_4)_3$ (0.5)	+1.84	CH_3NO_2	23 W CFL	14[d]	9	75
5	$Ru(bpz)_3(PF_6)_2$ (1.0)	+1.45	CH_3NO_2	23 W CFL	55[d]	8	77
6	$Mes\text{-}Acr(BF_4)$ (5)	+2.06	DCE	blue LEDs	53[d]	0	23[d]
7	$TPT(BF_4)$ (3)	+2.28	CH_3CN	blue LEDs	3[d]	0	84
8	chloranil (10)	+3.33	CH_3CN	NUV	36[d,e]	7	
9	DCB (10)	+2.67	CH_3CN	NUV	10[d,e]	42	
10	TCB (10)	+3.15	CH_3CN	NUV	50[d,e]	8	41
11	DCN (10)	+2.19	CH_3CN	NUV	39[d,e]	14	
12	DCA (10)	+1.97	CH_3CN	NUV	43[d,e]	4	41

[a] All $E_{1/2}$* values were reported in CH_3CN, except for $[Cr(Ph_2phen)_3](BF_4)_3$, which was taken in CH_3NO_2.
[b] ^1H NMR yields with dodecyl acetate as internal standard
[c] Yield diminished because of oxidation to ***S20***.
[d] Majority of mass balance is recovered starting material.
[e] Reaction time was 20 h.

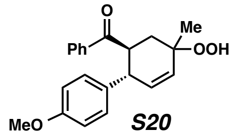

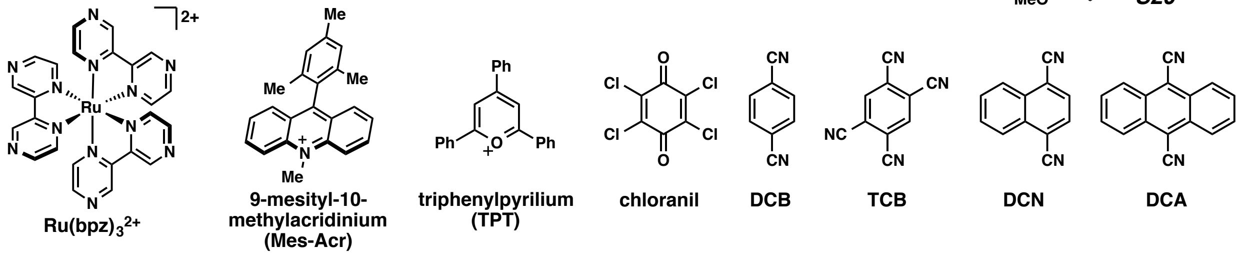

Excited state reduction potentials were found in the literature:

$Ru(bpz)_3^{2+}$: Prier, C. K.; Rankic, D. A.; MacMillan, D. W. C. *Chem. Rev.* **2013**, *113*, 5322-5363.

$Mes\text{-}Acr^+$: Ohkubo, K.; Mizushime, K.; Iwata, R.; Souma, K.; Suzuki, S.; Fukuzumi, S. *Chem. Commun.* **2010**, *46*, 601-603.

TPT^+: Gesmundo, N. J.; Nicewicz, D. A. *Beilstein J. Org. Chem.* **2014**, *10*, 1272-1281.

Chloranil, DCB, TCB, DCN, DCA: Fagnoni, M.; Dondi, D.; Ravelli, D.; Albini, A. *Chem. Rev.* **2007**, *107*, 2725-2756.

General Procedure for Full Cycloaddition: A flame-dried 2-dram borosilicate vial open to air was charged with alkene **1** (11.9 mg, 0.0500 mmol), diene **2** (15.0 µL, 0.150 mmol), catalyst, nitromethane (0.500 mL), and dodecyl acetate (internal standard, 11.4 mg, 0.0500 mmol). The vial was then capped and placed in front of the indicated light source. The solution was irradiated with stirring for 6 h, then passed through a short plug of silica (2.0-2.5 x 1 cm, Et_2O eluent). The volatile materials were removed by rotary evaporation, and the resulting crude product mixture was analyzed by ^1H NMR.

General Procedure for Vinylcyclobutane Rearrangement: A flame-dried 2-dram borosilicate vial open to air was charged with vinylcyclobutane **6** (9.2 mg, 0.0300 mmol), catalyst, nitromethane (0.300 mL), and dodecyl acetate (internal standard, 6.9 mg, 0.0300 mmol). The vial was then capped and placed in front of the indicated light source. The solution was irradiated with stirring for 6 h, then passed through a short plug of silica (2.0-2.5 x 1 cm, Et_2O eluent). The solvent was removed by rotary evaporation, and the resulting crude product mixture was analyzed by ^1H NMR.

Cycloaddition Catalyzed by Aminium Radical Cation Salt

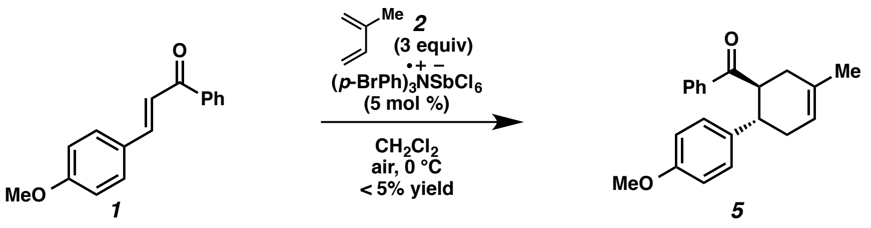

A flame-dried 2-dram borosilicate vial open to air was charged with alkene **1** (9.5 mg, 0.0400 mmol), diene **2** (12.0 µL, 0.120 mmol), CH_2Cl_2 (0.400 mL), and dodecyl acetate (internal standard, 9.1 mg, 0.0400 mmol). The vial was

then capped and cooled to 0 °C, then (p-BrPh)$_3$NSbCl$_6$ (1.6 mg, 0.00200 mmol) was added. The reaction mixture was stirred at 0 °C for 1 h, then was passed through a short plug of silica (2.0-2.5 x 1 cm, CH_2Cl_2 eluent). The volatile materials were removed by rotary evaporation, and the resulting crude product mixture was analyzed by ^{1}H NMR. <5% yield of cyclohexene **5** had formed. An analogous experiment was performed where the vial was irradiated with a 23 W CFL (~45 °C) for 6 h. Again, <5% yield of cyclohexene **5** was formed.

Vinylcyclobutane Rearrangement Catalyzed by Aminium Radical Cation Salt

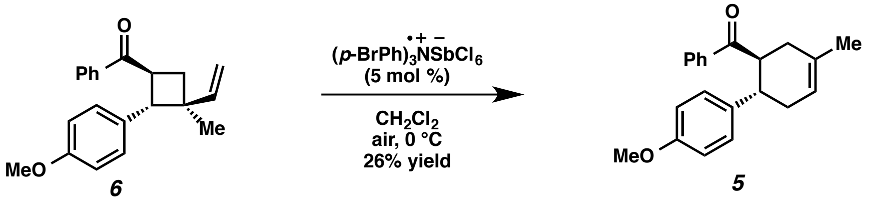

A flame-dried 2-dram borosilicate vial open to air was charged with vinylcyclobutane **6** (12.3 mg, 0.0400 mmol), CH_2Cl_2 (0.400 mL), and dodecyl acetate (internal standard, 9.1 mg, 0.0400 mmol). The vial was then capped and cooled to 0 °C, then (p-BrPh)$_3$NSbCl$_6$ (1.6 mg, 0.00200 mmol) was added. The reaction mixture was stirred at 0 °C for 1 h, then was passed through a short plug of silica (2.0-2.5 x 1 cm, CH_2Cl_2 eluent). The volatile materials were removed by rotary evaporation, and the resulting crude product mixture was analyzed by ^{1}H NMR. 26% yield of cyclohexene **5** had formed.

Electrochemical Data

Table S3. Compilation of measured electrochemical data.[a]

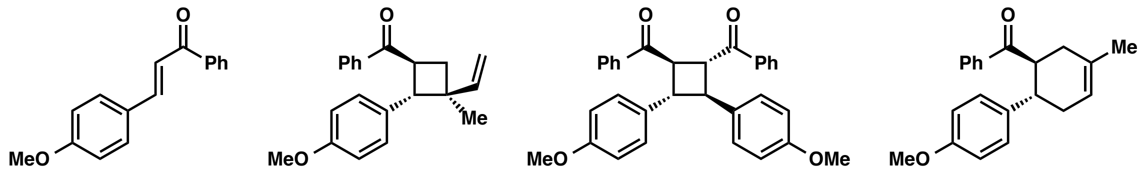

1 6 30 5

Compound	$E_{1/2}$ $M^{\bullet+/0}$ (V) (CH_3NO_2)[b]	E^o_{ox} (V) (CH_3NO_2)[c]	$E_{1/2}$ $M^{\bullet+/0}$ (V) (CH_3CN)[b]	E^o_{ox} (V) (CH_3CN)[c]
**1**	+1.51	+1.30	+1.29	+1.14
**1 + O_2 bubbling**	+1.65	+1.42	+1.22	+1.07
**1 + isoprene + air**[d]	+1.31			
**6**	+1.38	+1.15	+1.33	+1.10
**6 + O_2 bubbling**	+1.33	+1.08	+1.33	+1.04
**6 + isoprene + air**[e]	+1.14	+0.77	+1.24	+0.86
**30**[d]	+1.10	+0.96	+1.14	+1.00
**30 + O_2 bubbling**	+1.05	+0.91	+1.10	+0.98
**30 + isoprene**[f]	+1.03	+0.88	+1.11	+0.97
**30 + isoprene + air**[f]	+1.04	+0.89	+1.14	+0.98
**5**	+1.30	+1.13	+1.19	+1.02
**5 + O_2 bubbling**	+1.33	+1.07	+1.21	+1.03

[a] All potentials referenced to Fc^+/Fc. Scans were performed at 100 mV/s scan rates in 0.1 M Bu_4NPF_6 solutions.
[b] $E_{1/2}$ and E^o_{ox} values were gathered from square wave voltammograms gathered at 4 mV increments.
[c] "E^o_{ox}" defined here as the onset potential for the oxidation wave occurring when scanning the potential in the positive direction.
[d] Data acquired with 3 equiv isoprene added.
[e] Data acquired with 2 equiv isoprene added.
[f] Data acquired with 6 equiv isoprene added.

To convert the potentials from V vs. Fc^+/Fc to V vs. SCE, 0.35 was added to the potentials taken in CH_3NO_2, and 0.40 was added to the potentials taken in CH_3CN.[5]

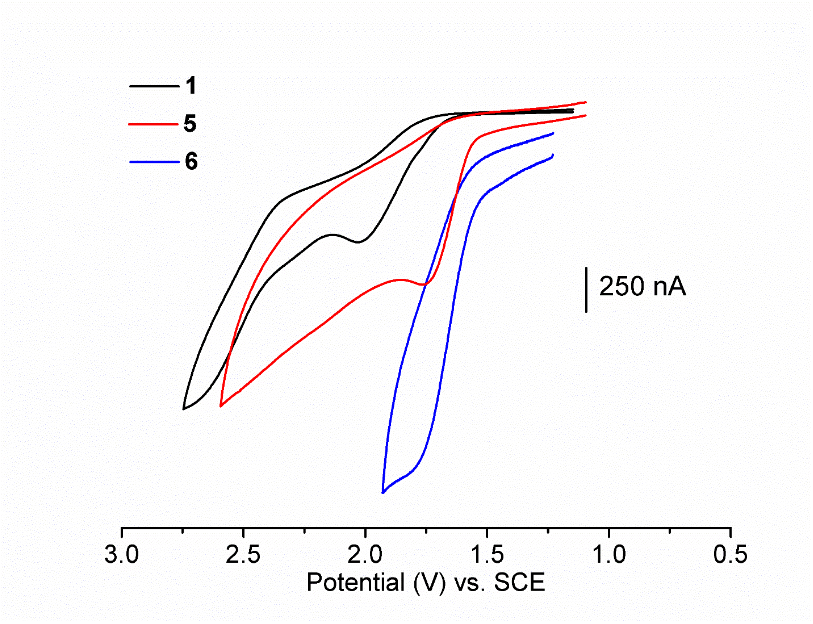

Figure S1. Cyclic voltammograms (CVs) of **1** (black trace), **6** (blue trace) and **5** (red trace) in a 0.1 M Bu_4NPF_6 solution in CH_3NO_2 measured at a scan rate of 100 mV/s.

Cr-Photocatalyzed Vinylcyclobutane Rearrangement

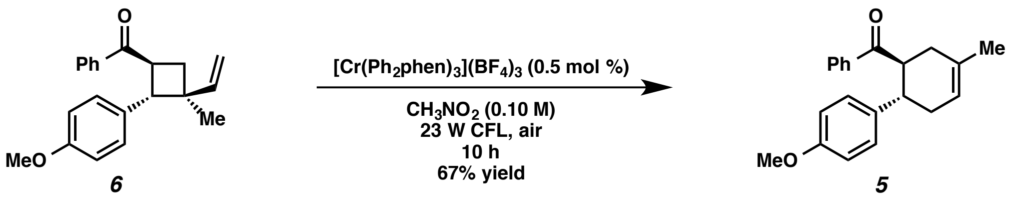

A 1/2-dram borosilicate vial open to air was charged with vinylcyclobutane **6** (15.3 mg, 0.0500 mmol), $[Cr(Ph_2phen)_3](BF_4)_3$ (0.3 mg, 0.000250 mmol) and nitromethane (0.500 mL). The vial was capped and irradiated with a 23 W CFL in a closed box lined with aluminum foil for 10 h with stirring. The reaction mixture was then passed through a short plug of silica (2.0-2.5 x 1 cm, Et_2O eluent). The solvent was removed by rotary evaporation, and the crude product was purified by flash chromatography (100% hexanes → 9:1 hexanes/EtOAc eluent) to afford cyclohexene **5** (10.2 mg, 67% yield) as a colorless oil. Only trace cyclohexene **5** was observed in the absence of catalyst (6 h) or in the absence of light (60 h). When this same reaction was performed under Ar (degassed by three freeze-pump-thaw cycles) in a 50-mL Schlenk flask with dodecyl acetate (11.4 mg, 0.0500 mmol) as an internal standard, the product (**5**) was formed in 64% NMR yield after 6 h.

Vinylcyclobutane Trapping Experiments

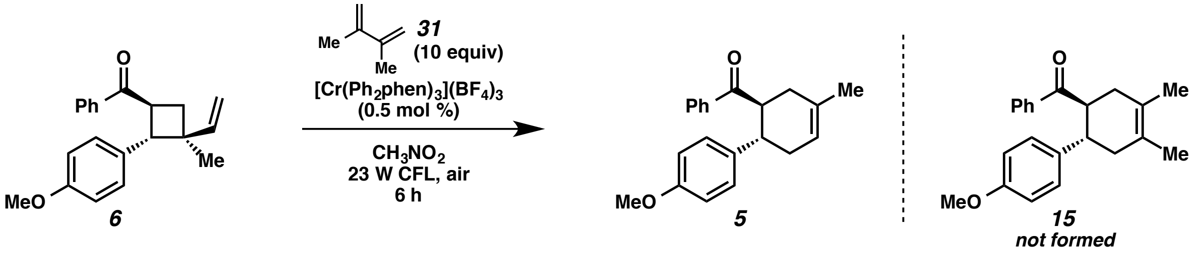

Performed according to the *General Procedure* using vinylcyclobutane **6** (9.5 mg, 0.0310 mmol), diene **31** (34.0 µL, 0.310 mmol), $[Cr(Ph_2phen)_3](BF_4)_3$ (0.2 mg, 0.000155 mmol), and nitromethane (0.310 mL). The reaction mixture was irradiated for 6 h, then was passed through a short plug of silica (2.0-2.5 x 1 cm, Et_2O eluent). The volatile materials were removed by rotary evaporation and the crude reaction mixture was analyzed by ^1H NMR to reveal only the formation of cyclohexene **5**. Cyclohexene **15** was not formed.

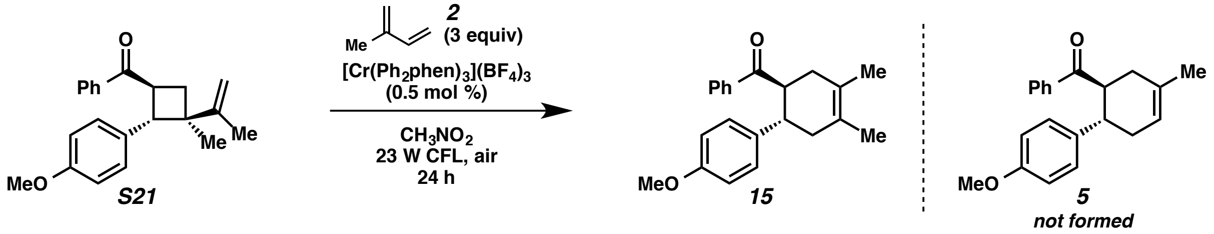

Performed according to the *General Procedure* using vinylcyclobutane **S21**[6] (16.0 mg, 0.0500 mmol), diene **2** (15.0 µL, 0.150 mmol), $[Cr(Ph_2phen)_3](BF_4)_3$ (0.3 mg, 0.000250 mmol), nitromethane (0.500 mL), and dodecyl acetate (internal standard, 11.4 mg, 0.0500 mmol). The reaction mixture was irradiated for 24 h, then was passed through a short plug of silica (2.0-2.5 x 1 cm, Et_2O eluent). The volatile materials were removed by rotary evaporation and the crude reaction mixture was analyzed by ^1H NMR to reveal only the formation of cyclohexene **15**. Cyclohexene **5** was not formed.

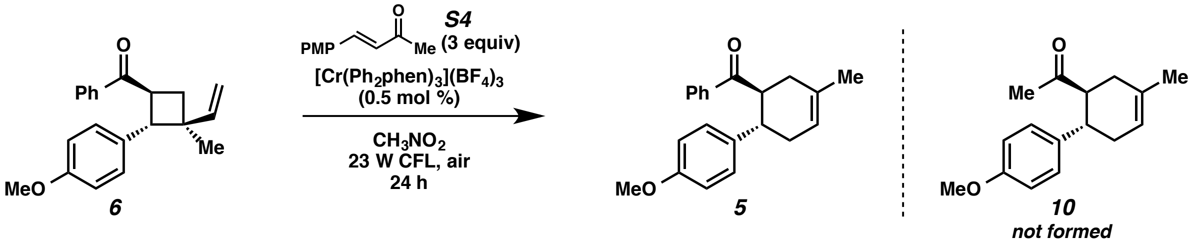

Performed according to the *General Procedure* using vinylcyclobutane **6** (15.3 mg, 0.0500 mmol), alkene **S4** (26.4 mg, 0.150 mmol), $[Cr(Ph_2phen)_3](BF_4)_3$ (0.3 mg, 0.000250 mmol), nitromethane (0.500 mL), and dodecyl acetate (internal standard, 11.4 mg, 0.0500 mmol). The reaction mixture was irradiated for 24 h, then was passed through a short plug of silica (2.0-2.5 x 1 cm, Et_2O eluent). The volatile materials were removed by rotary evaporation and the crude reaction mixture was analyzed by ^{1}H NMR to reveal only the formation of cyclohexene **5**. Cyclohexene **10** was not formed.

UV/Vis Spectra

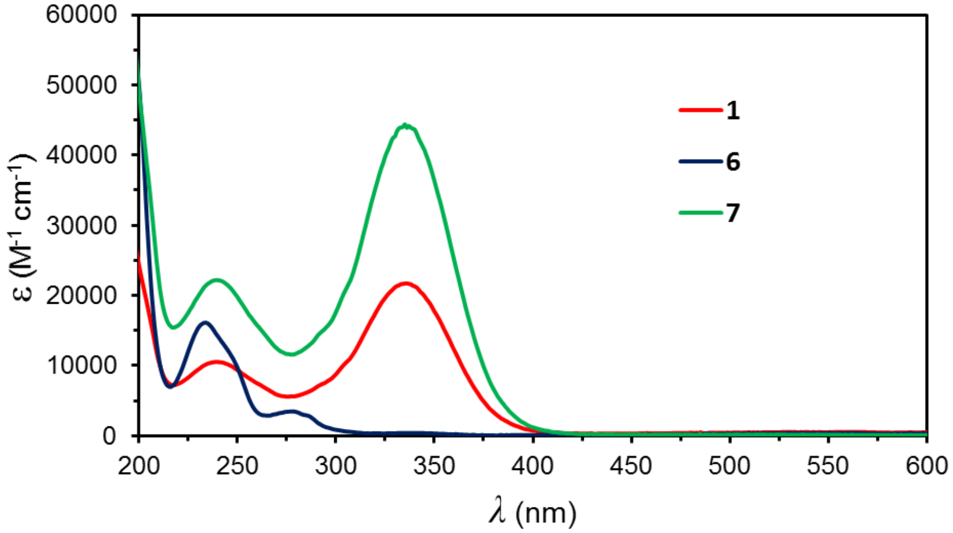

Figure S2. UV-vis spectra of compounds **1** (red trace), **6** (blue trace) and **7** (green trace) in acetonitrile.

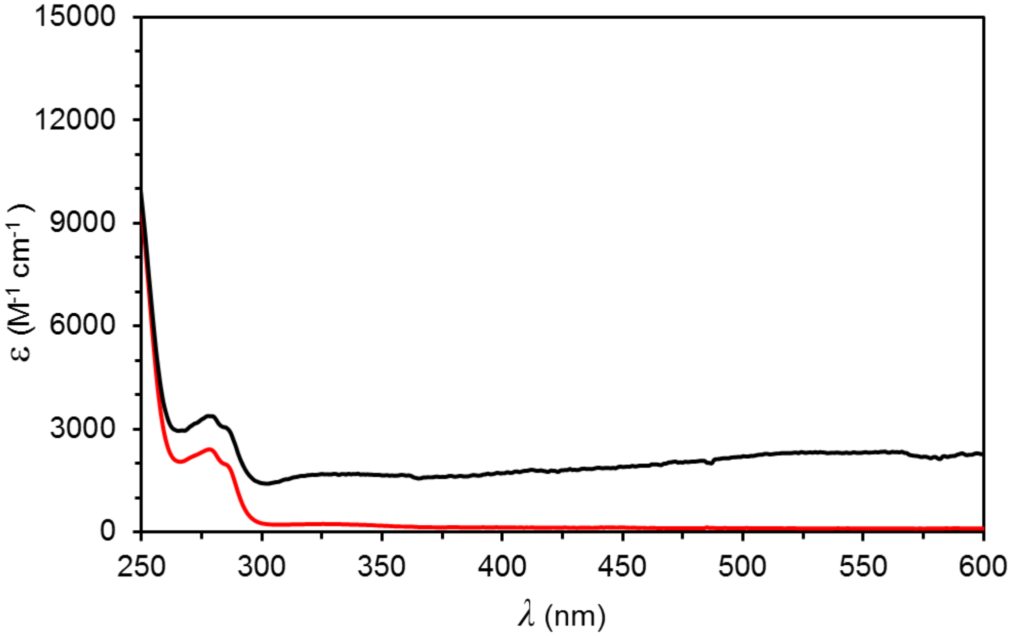

Figure S3. UV-vis spectra of **6** before and after 18 hour irradiation under 420 nm light. The increased absorption is due to evaporation of solvent and/or instrument drift.

Enone Dimer Experiments

Enone dimer **30** was synthesized through irradiation (350 nm) of a concentrated solution of 4-methoxychalcone (**1**) in nitromethane (1.0 M) in the presence of benzophenone (0.1 equiv). All spectroscopic data were consistent with previously reported values.[7]

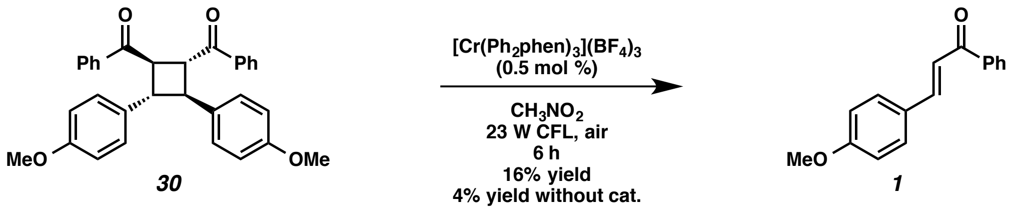

Cycloreversion of Enone Dimer. To a 1/2-dram vial upon to air was added dimer **30** (8.0 mg, 0.0168 mmol), $[Cr(Ph_2phen)_3](BF_4)_3$ (0.1 mg, 0.0000840 mmol), nitromethane (0.170 mL), and dodecyl acetate (internal standard, 3.8 mg, 0.0168 mmol). The vial was capped and stirred in front of a 23 W CFL in a closed box lined with aluminum foil. The reaction mixture was irradiated for 6 h, then was passed through a short plug of silica (2.0-2.5 x 1 cm, Et_2O eluent). The volatile materials were removed by rotary evaporation and the crude reaction mixture was analyzed by ^1H NMR. In the presence of catalyst, enone **1** was obtained in 16% NMR yield. In the absence of catalyst, 4% NMR yield of enone **1** was observed.

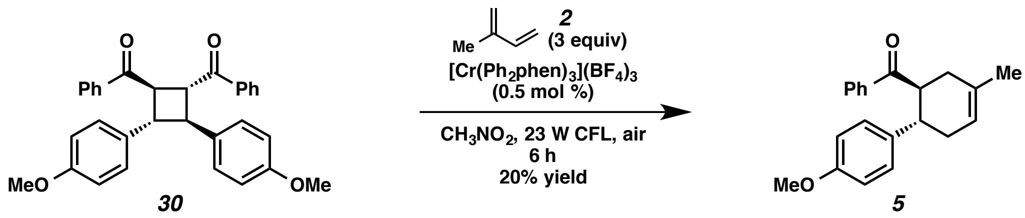

Trapping Experiment with Enone Dimer. To a 1/2-dram vial upon to air was added dimer **30** (8.0 mg, 0.0168 mmol), diene **2** (5.00 μL, 0.0504 mmol), $[Cr(Ph_2phen)_3](BF_4)_3$ (0.1 mg, 0.0000840 mmol), nitromethane (0.170 mL),

and dodecyl acetate (internal standard, 3.8 mg, 0.0168 mmol). The vial was capped and stirred in front of a 23 W CFL in a closed box lined with aluminum foil. The reaction mixture was irradiated for 6 h, then was passed through a short plug of silica (2.0-2.5 x 1 cm, Et_2O eluent). The volatile materials were removed by rotary evaporation and the crude reaction mixture was analyzed by ^1H NMR. Cyclohexene **5** was obtained in 20% NMR yield.

Static Emission Spectrum of 4-Methoxychalcone

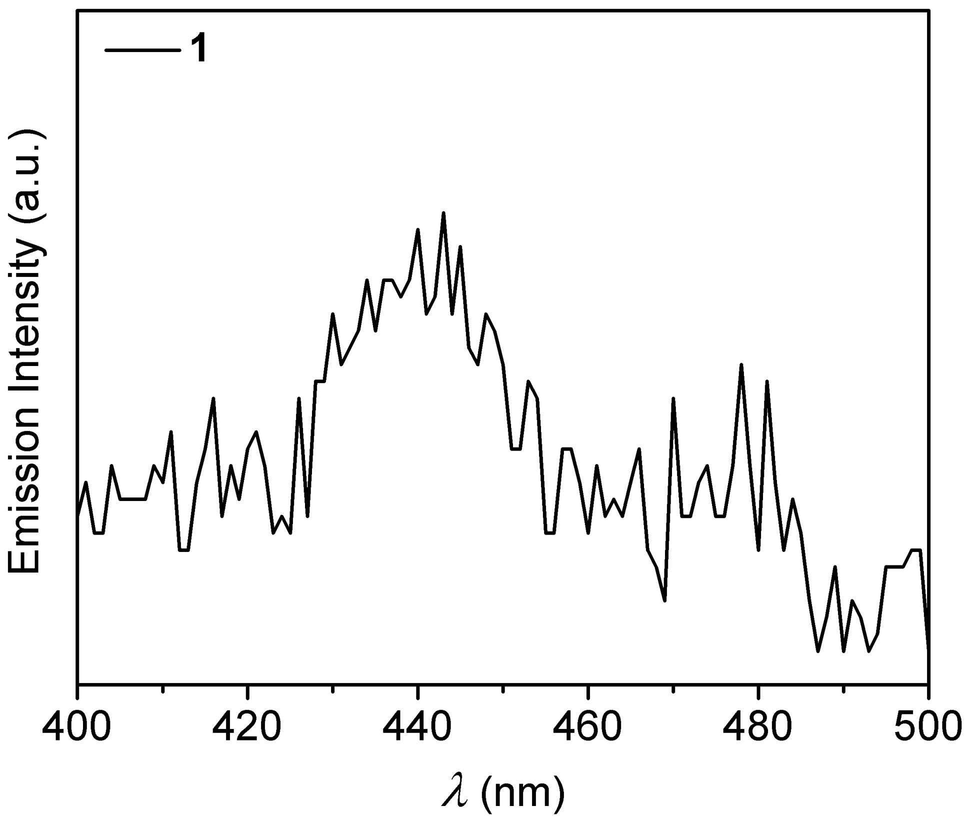

Figure S4. Static emission (λ_{ex} = 340 nm, bandwidth = 2 nm) of 4-methoxychalcone (**1**) in acetonitrile at 0.0016 M. The λ_{em} centered at 443 nm was reproduced on two separate samples that were degassed for ~45 min before the measurement was performed. A sample of acetonitrile revealed no increase in emission intensity over the wavelength range tested.

Competition Experiment

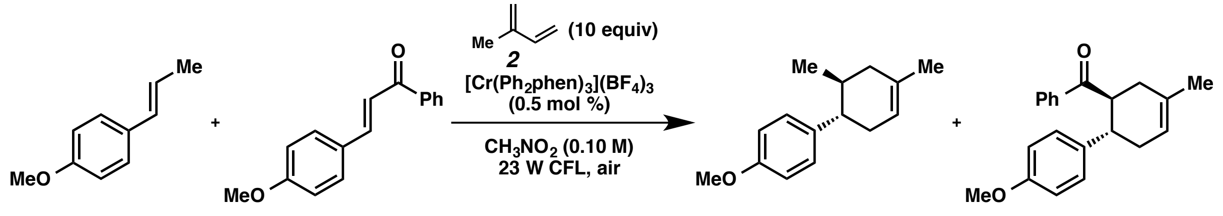

32 ***1*** ***33*** ***5***

Time (h)	% Remaining[a] *32*	*1*	% Formed[a,b] *33*	*5*
2	45	97	17	0 (3)
4	22	94	29	0 (4)
6	>5	94	64	<5 (5)
10	0	60	74	18 (0)
24	0	25	75	62 (0)

[a] NMR yields with dodecyl acetate as the internal standard
[b] Numbers in parentheses are yields of vinylcyclobutane *6*.

Procedure: A stock solution was prepared containing alkene **32** (36.8 mg, 0.248 mmol), alkene **1** (59.1 mg, 0.248 mmol), diene **2** (0.250 mL, 2.48 mmol), $[Cr(Ph_2phen)_3](BF_4)_3$ (1.6 mg, 0.00124 mmol), and dodecyl acetate (internal standard, 56.6 mg, 0.248 mmol) in nitromethane (2.48 mL). Into five 1-dr borosilicate vials was added 0.400 mL of the stock solution. The vials were capped and placed in front of a bright white 23 W compact fluorescent light bulb in a closed box lined with aluminum foil. At each indicated time, a vial was removed from the box, and the reaction mixture was passed through a short plug of silica (2.0-2.5 x 1 cm, Et_2O eluent). The volatile materials were removed by rotary evaporation, and the resulting crude product mixture was analyzed by ^1H NMR.

Reactivity of Unsubstituted Chalcone

For the reaction of chalcone (**S22**) with isoprene (**2**) under the Cr conditions, we observed the formation of vinylcyclobutane **S23**, as well as *cis*-**22**, but cyclohexene **29** did not form.

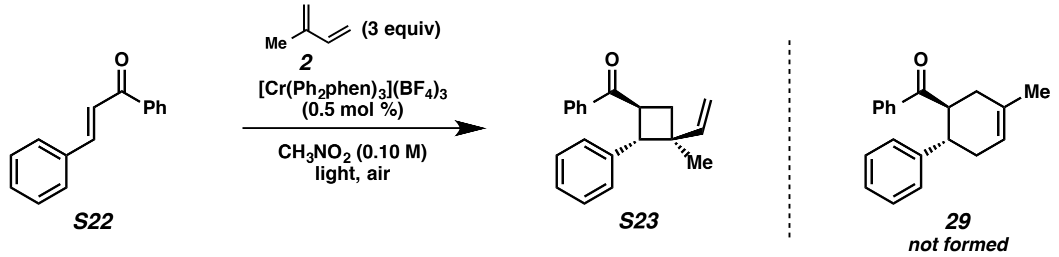

Time (h)	Light source	Catalyst?	Yield *S23* (%)[a]
72 h	23 W CFL	with cat.	11
72 h	23 W CFL	no cat.	15
64 h	NUV	with cat.	33
64 h	NUV	no cat.	40

[a] NMR yields with dodecyl acetate as the internal standard
[*cis*-chalcone was also observed in all cases.]

Procedure: A 2-dram borosilicate vial open to air was charged with alkene **S22** (15.6 mg, 0.0750 mmol), diene **2** (22.5 µL, 0.225 mmol), $[Cr(Ph_2phen)_3](BF_4)_3$ (0.5 mg, 0.000375 mmol) (if catalyst was being used), nitromethane (0.750 mL), and dodecyl acetate (internal standard, 17.1 mg, 0.0750 mmol). The vial was then capped and placed in front of the indicated light source. The solution was irradiated with stirring for the indicated time, then passed through a short plug of silica (2.0-2.5 x 1 cm, Et_2O eluent). The volatile materials were removed by rotary evaporation, and the resulting crude product mixture was analyzed by ^{1}H NMR.

Normal Electron-Demand Diels-Alder Reactions

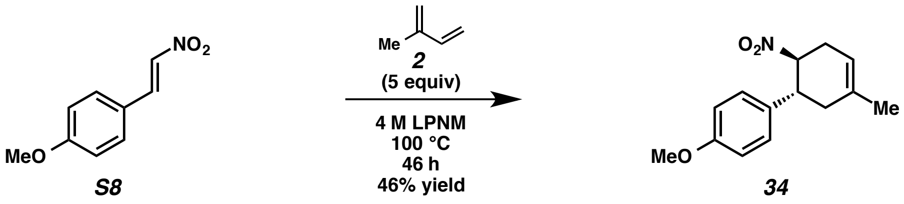

Cyclohexene 34. The 4 M LPNM solution was prepared by adding anhydrous $LiClO_4$ (0.145 g, 1.36 mmol) to nitromethane (0.340 mL, 0.50 M with respect to alkene **S8**) in a flame-dried 1-dram vial, open to air. The vial was capped and the mixture was stirred until complete dissolution of the $LiClO_4$ was achieved (ca. 2 h). To the solution was added alkene **S8** (30.0 mg, 0.167 mmol) and diene **2** (85.2 µL, 0.850 mmol). The vial was capped, and the reaction mixture was stirred at 100 °C. After 46 h, the reaction mixture was allowed to cool to ambient temperature, and then was diluted with H_2O (1 mL) and Et_2O (1 mL). The layers were separated, and the aqueous layer was extracted with Et_2O (2 x 1 mL). The combined organic layers were washed with brine, then dried over $MgSO_4$. The solvent was removed by rotary evaporation, and the crude residue was purified by flash chromatography (100% hexanes → 9:1 hexanes/EtOAc eluent) to afford cyclohexene **34** (19.4 mg, 46% yield, 18:1) as an off-white solid.

TLC: R_f = 0.74 in 3:1 hexanes/EtOAc, stained blue with *p*-anisaldehyde.

^{1}H NMR (400 MHz; $CDCl_3$): δ 7.14 (d, *J* = 8.4 Hz, 2H), 6.84 (d, *J* = 8.4 Hz, 2H), 5.40 (br s, 1H), 4.85 (td, *J* = 10.6, 5.7 Hz, 1H), 3.77 (s, 3H), 3.39 (td, *J* = 10.6, 6.7 Hz, 1H), 2.81-2.64 (comp. m, 2H), 2.35-2.23 (comp. m, 2H), 1.71 (s, 3H).

^{13}C NMR (100 MHz; $CDCl_3$): δ 158.8, 134.1, 132.0, 128.3, 116.7, 114.2, 87.7, 55.2, 43.8, 37.9, 31.3, 22.8.

IR (ATR, neat): 2963, 2909, 2839, 1551, 1512, 1250, 1034, 833, 733 cm^{-1}.

HRMS (ESI+): *m/z* calc'd for $(M + Na)^+$ $[C_{14}H_{17}NO_3 + Na]^+$: 270.1101, found 270.1105.

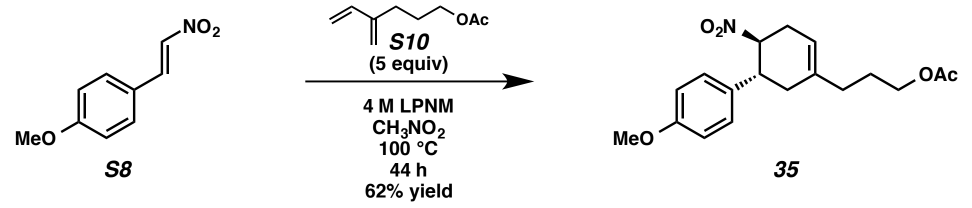

Cyclohexene 35. The 4 M LPNM solution was prepared by adding anhydrous $LiClO_4$ (85.1 mg, 0.800 mmol) to nitromethane (0.200 mL, 0.25 M with respect to alkene **S8**) in a flame-dried 1-dram vial, open to air. The vial was capped and the reaction mixture was stirred until complete dissolution of the $LiClO_4$ was achieved (ca. 2 h). To the solution was added alkene **S8** (9.0 mg, 0.0500 mmol) and diene **S10** (38.6 mg, 0.250 mmol). The vial was capped, and the reaction mixture was stirred at 100 °C. After 44 h, the reaction mixture was allowed to cool to ambient temperature, and then was diluted with H_2O (0.5 mL) and Et_2O (0.5 mL). The layers were separated, and the aqueous layer was extracted with Et_2O (2 x 1 mL). The combined organic layers were washed with brine, then dried over $MgSO_4$. The solvent was removed by rotary evaporation, and the crude residue was purified by flash chromatography (100% hexanes → 9:1 hexanes/EtOAc eluent) to afford cyclohexene **35** (10.3 mg, 62% yield, 17:1) as a colorless oil.

TLC: R_f = 0.36 in 3:1 hexanes/EtOAc, visualized by UV.

^{1}H NMR (500 MHz; $CDCl_3$): δ 7.17 (d, J = 8.7 Hz, 2H), 6.87 (d, J = 8.7 Hz, 2H), 5.47 (br s, 1H), 4.89 (ddd, J = 11.0, 10.1, 5.8 Hz, 1H), 4.08 (t, J = 13.0, 6.5 Hz, 3H), 3.80 (s, 3H), 3.41 (td, J = 11.0, 6.1 Hz, 1H), 2.82-2.72 (comp. m, 2H), 2.41-2.28 (comp. m, 2H), 2.12-2.05 (comp. m, 7H), 1.80-1.77 (comp. m, 2H).

^{13}C NMR (100 MHz; $CDCl_3$): δ 171.1, 158.9, 136.8, 131.9, 128.3, 117.0, 114.2, 87.7, 63.9, 55.3, 43.7, 36.3, 33.0, 31.2, 26.4, 21.0.

IR (ATR, neat): 2956, 2855, 1732, 1548, 1514, 1366, 1238, 1033, 831 cm^{-1}.

HRMS (ESI+): *m/z* calc'd for $(M + Na)^+$ $[C_{18}H_{23}NO_5 + Na]^+$: 356.1468, found 356.1467.

Thermal Diels-Alder Reactions

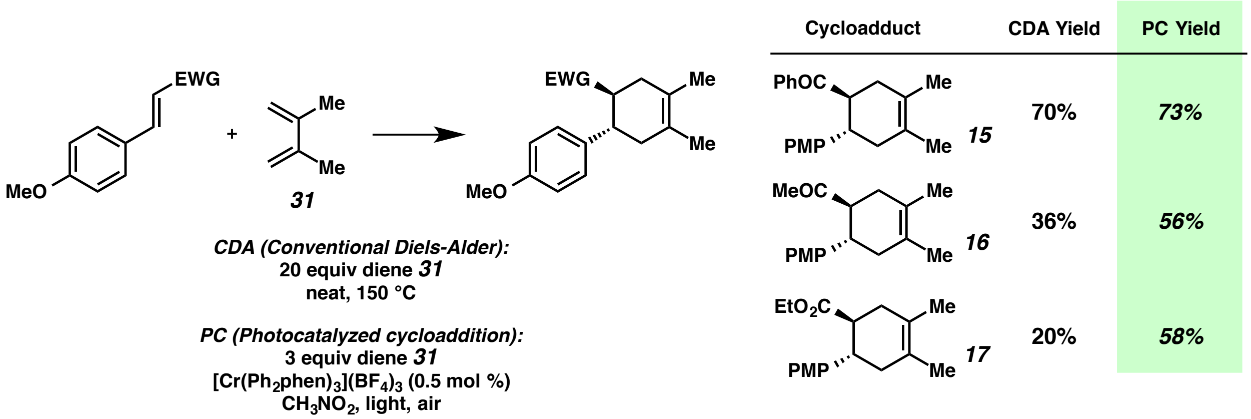

Scheme S1. Comparison of conventional Diels-Alder and Cr-photocatalyzed cycloaddition conditions.

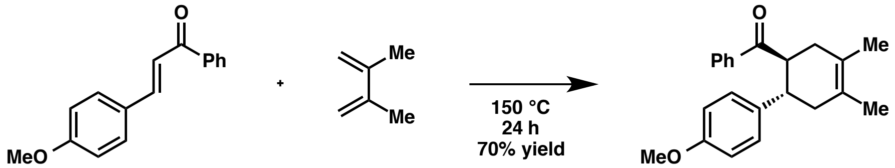

1 **31** (20 equiv) **15**

Cyclohexene 15. A 16 x 125 mm glass culture tube was charged with alkene **1** (95.3 mg, 0.400 mmol) and diene **31** (0.900 mL, 8.00 mmol). The tube was sealed with a Teflon cap and heated at 150 °C for 24 h. The reaction mixture was allowed to cool to ambient temperature, and the unreacted diene was removed by rotary evaporation. The resulting residue was purified by flash chromatography (100% hexanes → 20:1 hexanes/EtOAc eluent) to afford cyclohexene **15** (90.0 mg, 70% yield) as a colorless oil.

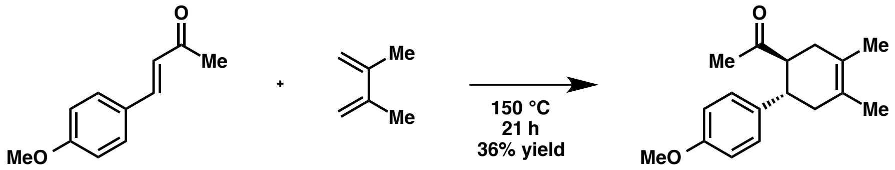

S4 **31** (20 equiv) **16**

Cyclohexene 16. A 2-dr vial was charged with alkene **S4** (15.4 mg, 0.0873 mmol) and diene **31** (0.200 mL, 1.75 mmol). The vial was sealed with a Teflon cap and heated at 150 °C for 21 h. The reaction mixture was allowed to cool to ambient temperature, and the unreacted diene was removed by rotary evaporation. The resulting residue was purified by flash chromatography (100% hexanes → 9:1 hexanes/EtOAc eluent) to afford cyclohexene **16** (8.1 mg, 36% yield) as a colorless oil.

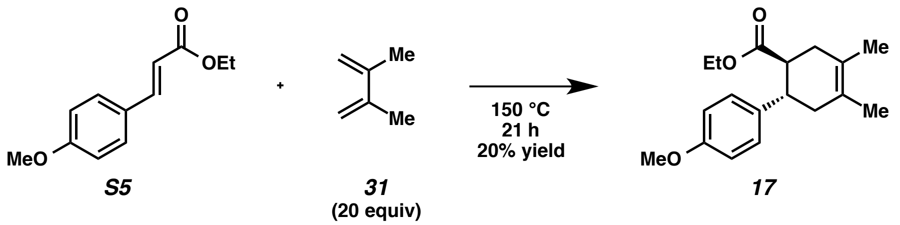

Cyclohexene 17. A 2-dr vial was charged with alkene **S5** (18.0 mg, 0.0873 mmol) and diene **31** (0.200 mL, 1.75 mmol). The vial was sealed with a Teflon cap and heated at 150 °C for 21 h. The reaction mixture was allowed to cool to ambient temperature, and the unreacted diene was removed by rotary evaporation. The resulting residue was purified by flash chromatography (100% hexanes → 9:1 hexanes/EtOAc eluent) to afford cyclohexene **17** (5.0 mg, 20% yield) as a colorless oil.

Synthesis of Electron-Poor Alkenes

General Notes: Reactions were performed in flame-dried glassware under Ar, unless otherwise noted. Aldol condensation reactions did not require flame-dried glassware and could be run in a stoppered flask.

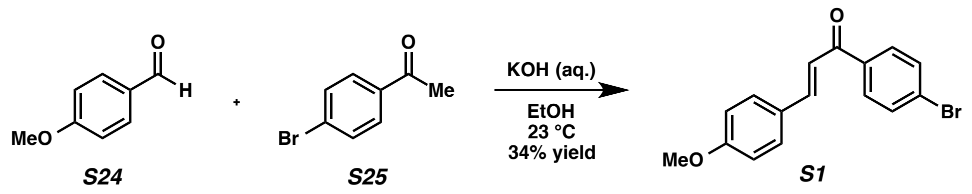

Ketone S1. To a solution of aldehyde **S24** (0.120 mL, 1.00 mmol) and ketone **S25** (0.199 g, 1.00 mmol) in EtOH (10.0 mL) at 23 °C was added aq. KOH (0.500 g in 1.00 mL H_2O). The reaction mixture was capped and stirred at ambient temperature for 16 h. EtOH was removed by rotary evaporation, and the crude mixture was partitioned between H_2O (15 mL) and EtOAc (15 mL). The aqueous layer was extracted with EtOAc (3 x 15 mL). The organic layers were combined, washed with brine (40 mL), and dried over $MgSO_4$. The solvent was removed by rotary evaporation, and the crude residue was purified by flash chromatography (100% hexanes → 6:1 hexanes/EtOAc eluent) to afford ketone **S1** (0.108 g, 34% yield) as an off-white solid.

TLC: R_f = 0.46 in 4:1 hexanes/EtOAc, stained with $KMnO_4$.

All spectroscopic data were consistent with previously reported values.[8]

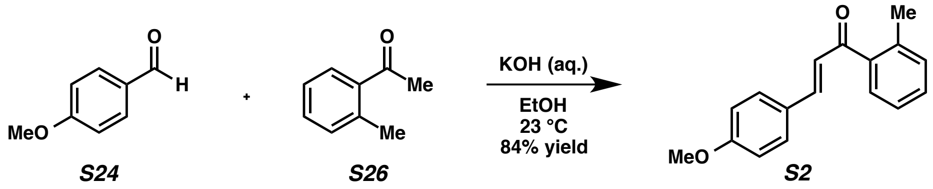

Ketone S2. To a solution of aldehyde **S24** (0.120 mL, 1.00 mmol) and ketone **S26** (0.130 mL, 1.00 mmol) in EtOH (10.0 mL) at 23 °C was added aq. KOH (0.500 g in 1.00 mL H_2O). The reaction mixture was capped and stirred at

ambient temperature for 16 h. EtOH was removed by rotary evaporation, and the crude mixture was partitioned between H_2O (15 mL) and EtOAc (15 mL). The aqueous layer was extracted with EtOAc (3 x 15 mL). The organic layers were combined, washed with brine (40 mL), and dried over $MgSO_4$. The solvent was removed by rotary evaporation, and the crude residue was purified by flash chromatography (100% hexanes → 6:1 hexanes/EtOAc eluent) to afford ketone **S2** (0.212 g, 84% yield) as a yellow oil.

TLC: R_f = 0.42 in 4:1 hexanes/EtOAc, stained with $KMnO_4$.

All spectroscopic data were consistent with previously reported values.[9]

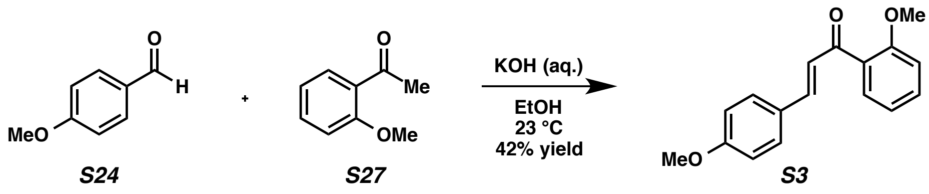

Ketone S3. To a solution of aldehyde **S24** (0.120 mL, 1.00 mmol) and ketone **S27** (0.14 mL, 1.00 mmol) in EtOH (10.0 mL) at 23 °C was added aq. KOH (0.500 g in 1.00 mL H_2O). The reaction mixture was capped and stirred at ambient temperature for 16 h. EtOH was removed by rotary evaporation, and the crude mixture was partitioned between H_2O (15 mL) and EtOAc (15 mL). The aqueous layer was extracted with EtOAc (3 x 15 mL). The organic layers were combined, washed with brine (40 mL), and dried over $MgSO_4$. The solvent was removed by rotary evaporation, and the crude residue was purified by flash chromatography (100% hexanes → 6:1 hexanes/EtOAc eluent) to afford ketone **S3** (0.114 g, 42% yield) as a yellow oil.

TLC: R_f = 0.22 in 4:1 hexanes/EtOAc, stained with $KMnO_4$.

^{1}H NMR (400 MHz; $CDCl_3$): δ 7.59-7.52 (comp. m, 3H), 7.46 (t, J = 7.9 Hz, 1H), 7.05-6.98 (comp. m, 2H), 6.91 (d, J = 8.4 Hz, 2H), 3.89 (s, 3H), 3.84 (s, 3H).

^{13}C NMR (100 MHz; $CDCl_3$): δ 193.2, 161.4, 157.9, 143.4, 132.5, 130.2, 130.1, 129.5, 127.8, 124.9, 120.7, 114.3, 111.6, 55.7, 55.4.

IR (ATR, neat): 2970, 2839, 1651, 1589, 1172, 1026, 825, 756 cm^{-1}.

HRMS (ESI+): *m/z* calc'd for $(M + H)^+$ $[C_{17}H_{16}O_3 + H]^+$: 269.1172, found 269.1176.

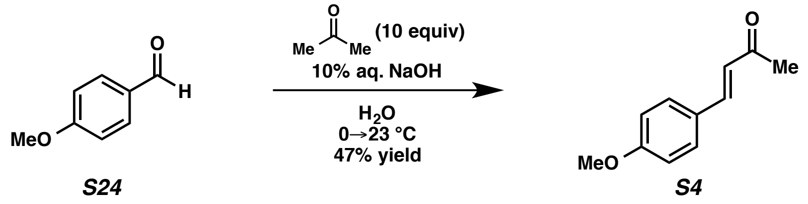

Ketone S4. To a solution of aldehyde **S24** (1.22 mL, 10.0 mmol) and acetone (7.35 mL, 100 mmol) in H_2O (2.00 mL) at 0 °C was added 10% aq. NaOH (5.00 mL) dropwise over 30 min. The mixture was allowed to warm to ambient temperature. After stirring for 20 h, 1 M aq. HCl was added slowly until a white precipitate formed (ca. 15 mL). After stirring for 30 min, the precipitate was collected by vacuum filtration through a Büchner funnel. The solid was washed sequentially with ice cold H_2O and EtOH, then dried under vacuum. The crude yellow solid was purified by flash chromatography (8:1 → 1:1 hexanes/EtOAc eluent) to afford ketone **S4** (0.834 g, 47% yield) as a yellow solid.

TLC: $R_f = 0.33$ in 4:1 hexanes/EtOAc, stained red with *p*-anisaldehyde.

All spectroscopic data were consistent with previously reported values.[10]

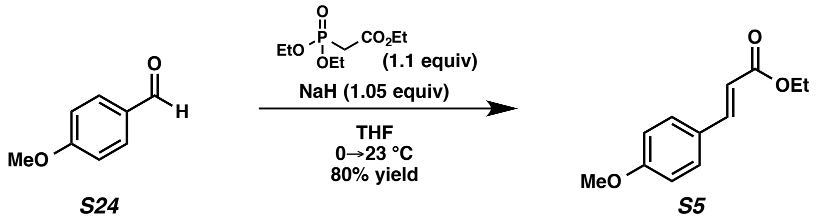

Ester S5. To a solution of triethyl phosphonoacetate (5.46 mL, 27.5 mmol) in THF (31.0 mL) at 0 °C was added NaH (1.06 g, 60% dispersion in mineral oil, 26.5 mmol). The reaction mixture was stirred at 0 °C for 45 min, then aldehyde **S24** (3.04 mL, 25.0 mmol) was added dropwise. The mixture was allowed to warm to ambient temperature and maintained for 15 h. The reaction mixture was then diluted with brine (30 mL), and the THF was removed by rotary evaporation. The mixture was extracted with CH_2Cl_2 (3 x 20 mL), and the combined organic layers were washed with

brine (50 mL), then dried over $MgSO_4$. The solvent was removed by rotary evaporation, and the crude residue was purified by flash chromatography (100% hexanes → 4:1 hexanes/EtOAc) to afford ester **S5** (4.14 g, 80% yield) as a white solid.

TLC: R_f = 0.48 in 4:1 hexanes/EtOAc, visualized by UV.

All spectroscopic data were consistent with previously reported values.[11]

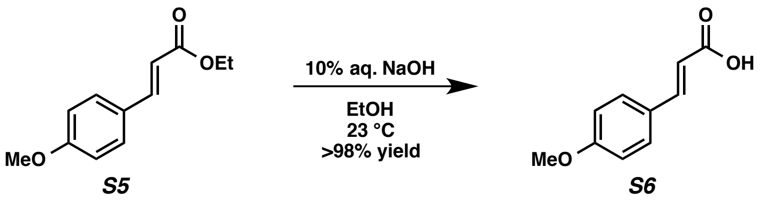

Carboxylic acid S6. To a solution of ester **S5** (1.00 g, 4.85 mmol) in EtOH (9.70 mL) at 23 °C was added 10% aq. NaOH (19.0 mL). The flask was stoppered, and the reaction mixture was stirred at ambient temperature for 45 h. The reaction mixture was diluted with 1 M aq. HCl (25 mL). EtOAc was added until the white solid was completely dissolved (ca. 75 mL) . The layers were separated, and the aqueous layer was extracted with EtOAc (3 x 25 mL). The combined organic layers were washed with brine (60 mL) and dried over $MgSO_4$. The solvent was removed by rotary evaporation to afford carboxylic acid **S6** (0.864 g, quantitative) as a white solid. The crude residue was sufficiently pure by ^{1}H NMR and therefore was used without further purification.

TLC: R_f = 0.60 in 1:1 hexanes/EtOAc, visualized by UV.

All spectroscopic data were consistent with previously reported values.[12]

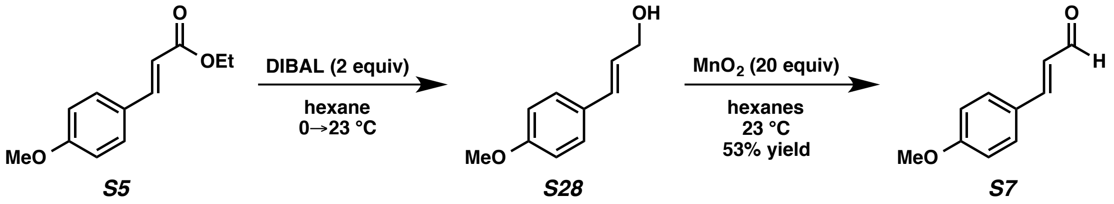

Alcohol S28. To a solution of ester **S5** (1.50 g, 7.27 mmol) in hexane (58.0 mL) at 0 °C was added DIBAL (14.6 mL, 1.0 M in hexane) dropwise with an addition funnel. The reaction mixture was stirred at 0 °C for 2 h, then was allowed to warm to ambient temperature and stirred an additional 20 min. The reaction mixture was then diluted with sat. aq. Rochelle salt (75.0 mL) and stirred overnight. The layers were separated, and the aqueous layer was extracted with CH_2Cl_2 (3 x 50 mL). The combined organic layers were washed with brine (50 mL), then dried over $MgSO_4$. The volatile materials were removed by rotary evaporation to afford alcohol **S28** as a white solid, which was used in subsequent reactions without further purification. All spectroscopic data were consistent with previously reported values.[13]

Aldehyde S7. To a solution of alcohol **S28** (0.739 g, 4.50 mmol) in hexanes (22.5 mL) was added MnO_2 (7.82 g, 90.0 mmol). The reaction mixture was stirred for 3 h, and then was filtered through a short plug of silica (5 cm high x 3 cm wide EtOAc eluent). The filtrate was concentrated by rotary evaporation, and the crude residue was purified by flash chromatography (100% hexanes → 3:1 hexanes/EtOAc eluent) to afford aldehyde **S7** (0.387 g, 53% yield) as a white solid.

TLC: R_f = 0.56 in 3:1 hexanes/EtOAc, stained with $KMnO_4$.

All spectroscopic data were consistent with previously reported values.[6]

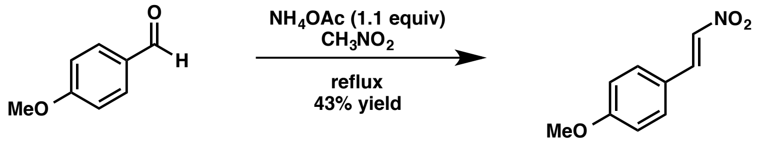

S24 *S8*

Nitroalkene S8. To a solution of NH_4OAc (0.848 g, 11.0 mmol) in nitromethane (14.0 mL, 0.71 M with respect to **S12**) at 23 °C was added aldehyde **S24** (1.22 mL, 10.0 mmol). The reaction flask was equipped with a reflux condenser (open to air) and the mixture was heated at 100 °C for 4 h. The reaction was allowed to cool to ambient temperature, and the nitromethane was removed by rotary evaporation. The resulting residue was dissolved in CH_2Cl_2 (20 mL), and the organic layer was washed sequentially with H_2O (20 mL) and brine (20 mL), then dried over

Na_2SO_4. The volatile materials were removed by rotary evaporation, and the crude residue was purified by flash chromatography (20:1 → 5:1 hexanes/EtOAc eluent) to afford nitroalkene **S8** (0.764 g, 43% yield) as a yellow solid.

TLC: R_f = 0.81 in 1:1 hexanes/EtOAc, stained with $KMnO_4$.

All spectroscopic data were consistent with previously reported values.[14]

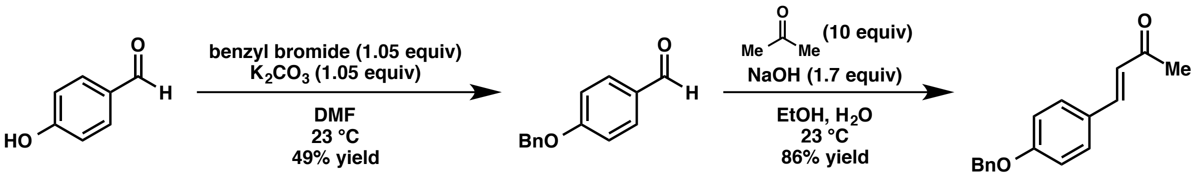

S29 *S30* *S18*

Aldehyde S30. To a solution of aldehyde **S29** (1.83 g, 15.0 mmol) and K_2CO_3 (2.18 g, 15.8 mmol) in DMF (15.0 mL) at 23 °C was added benzyl bromide (1.87 mL, 15.8 mmol). The reaction mixture was stirred for 24 h, and then was diluted with H_2O (20 mL) and EtOAc (20 mL). The layers were separated, and the aqueous layer was extracted with EtOAc (3 x 20 mL). The combined organic layers were washed with brine (50 mL), then dried over Na_2SO_4. The volatile materials were removed by rotary evaporation, and the crude residue was purified by flash chromatography (10:1 → 2:1 hexanes/EtOAc eluent) to afford aldehyde **S30** (1.572 g, 49% yield) as a white solid.

TLC: R_f = 0.42 in 4:1 hexanes/EtOAc, stained with $KMnO_4$.

^{1}H NMR (400 MHz; $CDCl_3$): δ 9.88 (s, 1H), 7.84 (d, J = 8.7 Hz, 2H), 7.44-7.35 (comp. m, 5H), 7.08 (d, J = 8.7 Hz, 2H), 5.15 (s, 2H).

Ketone S18. To a solution of aldehyde **S30** (0.424 g, 2.00 mmol) and acetone (1.47 mL, 20.0 mmol) in EtOH (3.30 mL) at 23 °C was added a solution of NaOH (0.136 g, 3.40 mmol) in H_2O (3.30 mL). The reaction mixture was stirred at ambient temperature for 20 h, and it was then diluted with H_2O (10 mL) and EtOAc (10 mL). The layers were separated, and the aqueous layer was extracted with EtOAc (3 x 10 mL). The combined organic layers were washed with brine (30 mL), then dried over $MgSO_4$. The volatile materials were removed by rotary evaporation, and the crude

residue was purified by flash chromatography (10:1 → 4:1 hexanes/EtOAc eluent) to afford ketone **S18** (0.433 g, 86% yield) as an off-white solid.

TLC: R_f = 0.24 in 4:1 hexanes/EtOAc, stained with $KMnO_4$.

^{1}H NMR (400 MHz; $CDCl_3$): δ 7.51-7.48 (comp. m, 2H), 7.45-7.34 (comp. m, 6H), 6.99 (d, J = 8.6 Hz, 2H), 6.60 (d, J = 16.1 Hz, 1H), 5.10 (s, 2H), 2.36 (s, 3H).

^{13}C NMR (100 MHz; $CDCl_3$): δ 198.4, 160.7, 143.2, 136.3, 130.0, 128.7, 128.2, 127.5, 127.3, 125.1, 115.3, 70.1, 27.4.

IR (ATR, neat): 2978, 2886, 1667, 1597, 1242, 1172, 910, 732 cm^{-1}.

HRMS (ESI+): *m/z* calc'd for $(M + H)^+$ $[C_{17}H_{16}O_2 + H]^+$: 253.1223, found 253.1224.

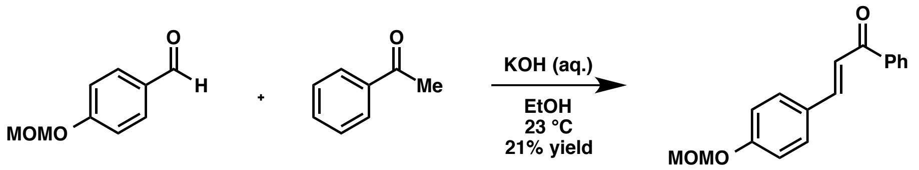

S31 *S32* *S19*

Ketone S19. To aldehyde **S31**[15] (0.341 g, 2.05 mmol) in EtOH (20.0 mL) and aq. KOH (1.00 g in 2.00 mL H_2O) at 23 °C was added ketone **S32** (0.230 mL, 2.00 mmol). The reaction mixture was capped and stirred at ambient temperature for 24 h. Then EtOH was removed by rotary evaporation, and the crude mixture was partitioned between H_2O (25 mL) and EtOAc (25 mL). The aqueous layer was extracted with EtOAc (3 x 20 mL). The organic layers were combined, washed with brine (50 mL), and dried over $MgSO_4$. The solvent was removed by rotary evaporation, and the crude residue was purified by flash chromatography (100% hexanes → 6:1 hexanes/EtOAc eluent) to afford ketone **S19** (0.172 g, 21% yield) as a yellow oil.

TLC: R_f = 0.35 in 4:1 hexanes/EtOAc, visualized by UV.

^{1}H NMR (400 MHz; $CDCl_3$): δ 8.01 (d, J = 8.6 Hz, 2H), 7.83 (d, J = 8.7 Hz, 1H), 7.61-7.56 (comp. m, 3H), 7.50 (t, J = 7.4 Hz, 2H), 7.43 (d, J = 15.7 Hz, 1H), 7.07 (d, J = 8.6 Hz, 2H), 5.22 (s, 2H), 3.49 (s, 3H).

^{13}C NMR (100 MHz; $CDCl_3$): δ 190.5, 159.2, 144.5, 138.4, 132.6, 131.9, 130.1, 128.6, 128.4, 120.2, 116.5, 94.1, 56.2.

IR (ATR, neat): 2978, 2901, 2824, 1659, 1589, 1504, 1149, 980, 833, 694 cm^{-1}.

HRMS (ESI+): *m/z* calc'd for $(M + H)^+$ $[C_{17}H_{16}O_3 + H]^+$: 269.1172, found 269.1172.

Synthesis of Dienes

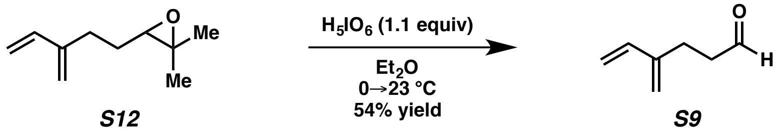

Diene S9. To a solution of epoxide **S12** (0.581 g, 3.81 mmol) in Et_2O (15.2 mL) at 0 °C was added periodic acid (0.955 g, 4.19 mmol). The reaction mixture was allowed to warm to ambient temperature, and was stirred for 20 h. The reaction mixture was passed through a short plug of celite (3 cm high x 2 cm wide, Et_2O eluent), and then the filtrate was washed sequentially with sat. aq. $NaHCO_3$ (20 mL) and 10% aq. $Na_2S_2O_3$ (20 mL). The combined aqueous washes were extracted with Et_2O (50 mL), and the combined organic layers were washed with brine (50 mL), then dried over $MgSO_4$. The solvent was removed by rotary evaporation, and the crude residue was purified by flash chromatography (100% hexanes → 15:1 hexanes/EtOAc eluent) to afford diene **S9** (0.228 g, 54% yield) as a colorless oil.

TLC: R_f = 0.67 in 3:1 hexanes/EtOAc, stained with $KMnO_4$.

All spectroscopic data were consistent with previously reported values.[16]

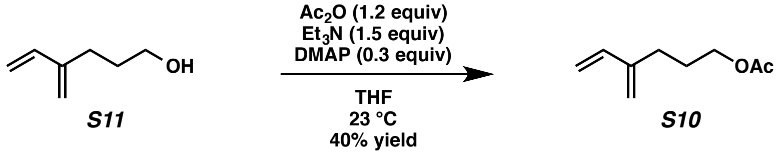

Diene S10. To a solution of alcohol **S11** (0.168 g, 1.50 mmol) and acetic anhydride (0.170 mL, 1.80 mmol) in THF (15.0 mL) at 23 °C were added triethylamine (0.310 mL, 2.25 mmol) and DMAP (55.0 mg, 0.450 mmol). The reaction mixture was stirred at ambient temperature for 22 h. The solvent was removed by rotary evaporation, and the crude residue was purified by flash chromatography (100% hexanes → 9:1 hexanes/EtOAc eluent) to afford diene **S10** (93.3 mg, 40% yield) as a colorless oil.

TLC: R_f = 0.76 in 3:1 hexanes/EtOAc, stained with $KMnO_4$.

All spectroscopic data were consistent with previously reported values.[17]

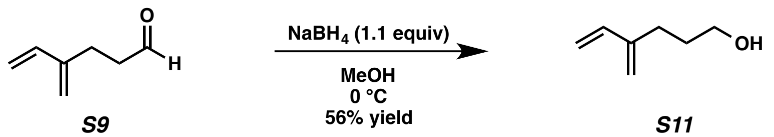

S9 *S11*

Diene S11. To a solution of aldehyde **S9** (2.64 g, 24.0 mmol) in MeOH (20.0 mL) at 0 °C was added $NaBH_4$ (0.999 g, 26.4 mmol). The reaction mixture was stirred at 0 °C for 20 min, then diluted with H_2O (20 mL) and Et_2O (20 mL), and was allowed to warm to ambient temperature. The layers were separated, and the aqueous layer was washed with Et_2O (3 x 30 mL). The combined organic layers were washed with brine (50 mL) and dried over $MgSO_4$. The solvent was removed by rotary evaporation, and the crude residue was purified by flash chromatography (100% hexanes → 3:1 hexanes/EtOAc eluent) to afford diene **S11** (1.57 g, 56% yield) as a colorless oil.

TLC: R_f = 0.27 in 3:1 hexanes/EtOAc, stained with $KMnO_4$.

All spectroscopic data were consistent with previously reported values.[18]

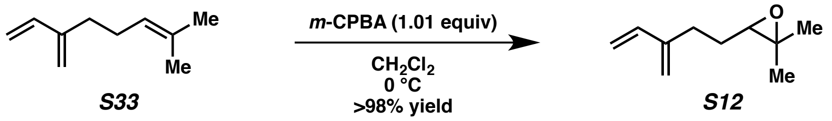

S33 *S12*

Diene S12. To a solution of myrcene (**S33**) (0.860 mL, 5.00 mmol) in CH_2Cl_2 (6.90 mL) at 0 °C was added *m*-CPBA (1.25 g, 5.05 mmol). The reaction mixture was stirred at 0 °C for 10 min, and then was diluted with 10% aq. NaOH (10 mL). The reaction mixture was allowed to warm to ambient temperature. The layers were separated, and the aqueous layer was extracted with CH_2Cl_2 (3 x 10 mL). The combined organic layers were washed sequentially with H_2O (20 mL) and brine (20 mL), then dried over $MgSO_4$. The solvent was removed by rotary evaporation to afford diene **S12** (0.767 g, quantitative) as a colorless oil which was used without further purification.

TLC: R_f = 0.76 in 3:1 hexanes/EtOAc, stained blue with *p*-anisaldehyde.

All spectroscopic data were consistent with previously reported values.[19]

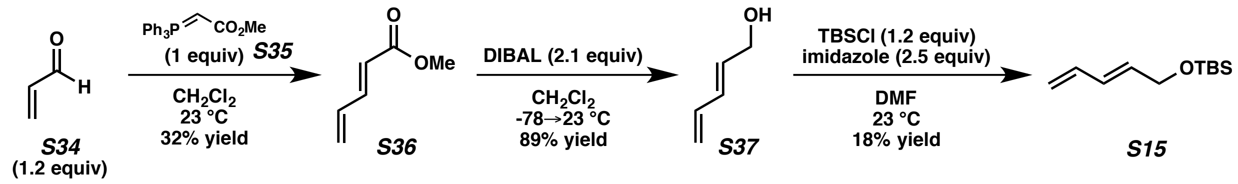

Ester S36. To a solution of ylide **S35** (8.36 g, 25.0 mmol) in CH_2Cl_2 (62.5 mL) at 23 °C was added aldehyde **S34** (2.00 mL, 30.0 mmol). The reaction mixture was stirred for 45 h, and then was filtered through a plug of silica (5 cm high x 3 cm wide, CH_2Cl_2 eluent). The filtrate was concentrated by rotary evaporation, and the crude residue was purified by flash chromatography (100% hexanes → 9:1 hexanes/EtOAc eluent) to afford ester **S36** (0.891 g, 32% yield) as a colorless oil.

TLC: R_f = 0.72 in 3:1 hexanes/EtOAc, stained with $KMnO_4$.

^{1}H NMR (400 MHz; $CDCl_3$): δ 7.27 (dd, *J* = 15.4, 10.7 Hz, 1H), 6.45 (dt, *J* = 16.9, 10.7 Hz, 1H), 5.91 (d, *J* = 15.4 Hz, 1H), 5.61 (d, *J* = 16.9 Hz, 1H), 5.50 (d, *J* = 10.1 Hz, 1H), 3.75 (s, 3H).

Alcohol S37. To a solution of ester **S36** (0.516 g, 4.60 mmol) in CH_2Cl_2 (7.70 mL) at -78 °C was added DIBAL (9.70 mL, 1.0 M in hexanes, 9.70 mmol). The reaction mixture was allowed to warm to 23 °C, and was stirred for 3.5 h. The reaction mixture was diluted with Et_2O (15 mL), then H_2O (0.330 mL) was added, followed by 1.0 M aq. NaOH (0.660 mL), then H_2O (0.330 mL) again, causing a white precipitate to formed. The mixture was stirred for 1 h, then $MgSO_4$ was added. After stirring for an additional 10 min, the mixture was filtered through celite (CH_2Cl_2 eluent). The solvent was removed by rotary evaporation to afford alcohol **S37** (0.345 g, 89% yield), which was used without further purification. All spectroscopic data were consistent with previously reported values.[20]

Diene S15. To a solution of alcohol **S37** (0.505 g, 6.00 mmol) in DMF (8.80 mL) at 23 °C was added TBSCl (1.09 g, 7.20 mmol) and imidazole (1.02 g, 15.0 mmol). The reaction mixture was stirred at ambient temperature for 16 h, and then was diluted with sat. aq. NH_4Cl (10 mL) and Et_2O (10 mL). The layers were separated, and the aqueous layer was extracted with Et_2O (3 x 15 mL). The combined organic layers were washed with brine (30 mL) and dried over $MgSO_4$. The solvent was removed by rotary evaporation, and the crude residue was purified by flash chromatography (100% hexanes → 20:1 hexanes/EtOAc eluent) to afford diene **S15** (0.220 g, 18% yield) as a colorless oil.

TLC: R_f = 0.86 in 3:1 hexanes/EtOAc, stained with $KMnO_4$.

All spectroscopic data were consistent with previously reported values.[21]

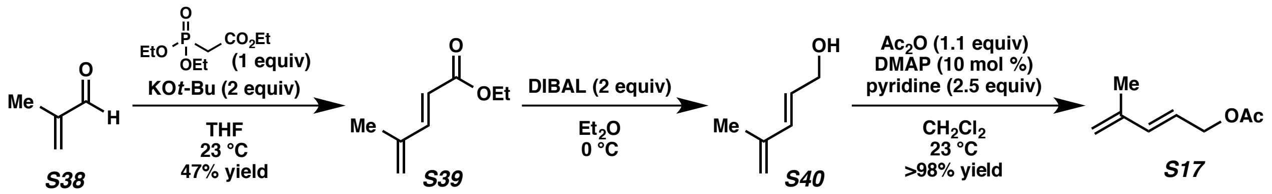

Ester S39. To a solution of triethyl phosphonoacetate (3.97 mL, 20.0 mmol) in THF (40.0 mL) at 23 °C was added KO*t*-Bu (4.49 g, 40.0 mmol). The reaction mixture was stirred for 35 min, then aldehyde **S38** (1.65 mL, 20.0 mmol) was added. The reaction mixture was stirred for 3 h, and then was diluted with H_2O (40 mL) and Et_2O (40 mL). The layers were separated, and the aqueous layer was extracted with Et_2O (3 x 30 mL). The combined organic layers were washed with brine (50 mL), then dried over $MgSO_4$. The solvent was removed by rotary evaporation, and the crude residue was purified by flash chromatography (100% hexanes → 4:1 hexanes/EtOAc) to afford ester **S39** (1.32 g, 47% yield) as a colorless oil.

TLC: R_f = 0.71 in 4:1 hexanes/EtOAc, stained with $KMnO_4$.

All spectroscopic data were consistent with previously reported values.[22]

Alcohol S40. To a solution of ester **S39** (1.32 g, 9.42 mmol) in Et_2O (23.6 mL) at 0 °C was added DIBAL (18.8 mL, 1.0 M in hexanes, 18.8 mmol). The reaction mixture stirred at 0 °C for 2 h, and then was diluted with MeOH (5.00

mL), then 1.0 M aq. HCl (5.00 mL) was added, followed by Et_2O (10.0 mL). The layers were separated, and the aqueous layer was extracted with Et_2O (3 x 15 mL). The combined organic layers were washed with brine (20 mL), then dried over $MgSO_4$. The solvent was removed by rotary evaporation to afford alcohol **S40**, which was used without further purification. All spectroscopic data were consistent with previously reported values.[23]

Diene S17. To a solution of alcohol **S40** (0.196 g, 2.00 mmol) in CH_2Cl_2 (8.00 mL) at 23 °C was added DMAP (24.4 mg, 0.200 mmol) and pyridine (0.400 mL, 5.00 mmol). The solution was stirred at ambient temperature for 15 min, and then acetic anhydride (0.210 mL, 2.20 mmol) was added. After 16 h, the reaction mixture was diluted with H_2O (10 mL). The layers were separated, and the organic layer was washed sequentially with 1 M aq. HCl (10 mL), H_2O (10 mL), sat. aq. $NaHCO_3$ (10 mL), and brine (10 mL). The organic layer was dried over $MgSO_4$, and the solvent was removed by rotary evaporation. The crude residue was purified by flash chromatography (100% pentane → 9:1 pentane/Et_2O) to afford diene **S17** (0.279 g, quantitative) as a colorless oil.

TLC: R_f = 0.69 in 4:1 hexanes/EtOAc, stained with $KMnO_4$.

^{1}H NMR (400 MHz; $CDCl_3$): δ 6.37 (d, *J* = 15.7 Hz, 1H), 5.75-5.68 (m, 1H), 5.02 (d, *J* = 4.4 Hz, 1H), 4.63 (s, 1H), 4.61 (s, 1H), 2.07 (s, 3H), 1.84 (s, 3H).

^{13}C NMR (100 MHz; $CDCl_3$): δ 170.8, 141.0, 137.0, 123.0, 117.9, 65.0, 21.0, 18.4.

IR (ATR, neat): 2978, 2886, 1736, 1227, 964, 733 cm^{-1}.

HRMS (ESI+): *m/z* calc'd for $(M + H)^+$ $[C_8H_{12}O_2 + H]^+$: 141.0910, found 141.0908.

Photochemical [2+2] Cycloaddition

An independent synthesis of vinylcyclobutane **6** was devised in order to determine its structure and perform additional experiments to confirm its role as a reaction intermediate.

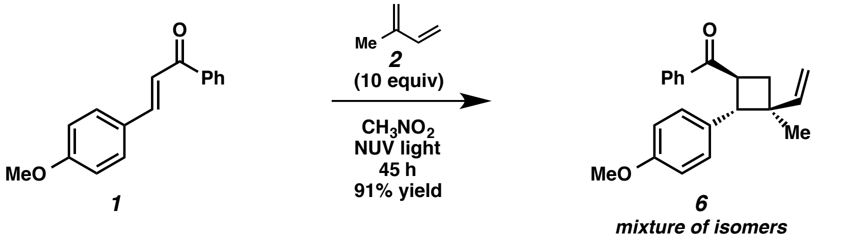

Vinylcyclobutane 6. A flame-dried 2-dram borosilicate vial open to air was charged with alkene **1** (0.238 g, 1.00 mmol), diene **2** (1.00 mL, 10.0 mmol), and nitromethane (2.50 mL). The vial was capped, and the reaction mixture was irradiated with 300, 350, and 419 nm light, with stirring, for 45 h. The volatile materials were then removed by rotary evaporation, and the residue was purified by flash chromatography (100% hexanes → 15:1 hexanes/EtOAc eluent) to afford cyclobutane **6** as the major product of an inseparable mixture of isomers (0.278 g, 91% combined yield) as a pale yellow oil.

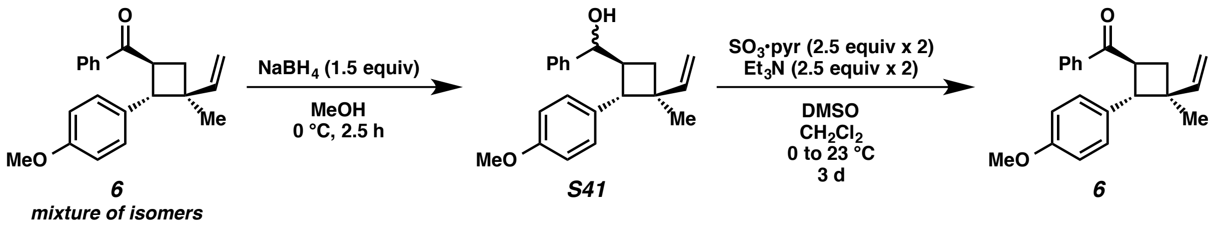

The vinylcyclobutane isomers were difficult to separate by flash chromatography. The phenyl ketone was reduced to the alcohol (**S41**) to make purification easier, and then was oxidized to the ketone again.

Alcohol S41. To the vinylcyclobutane mixture (148.0 mg, 0.483 mmol) in MeOH (3.22 mL) at 0 °C under argon was

added $NaBH_4$ (27.4 mg, 0.725 mmol). The reaction mixture was stirred at 0 °C for 2.5 h, then the volatile materials were removed by rotary evaporation. The crude residue was partitioned between Et_2O (10 mL) and sat. aq. NH_4Cl (10 mL). The aqueous layer was extracted with Et_2O (2 x 10 mL). The combined organic layers were washed with brine (30 mL) and dried over $MgSO_4$. The volatile materials were then removed by rotary evaporation, and the residue was purified by flash chromatography (100% hexanes → 10:1 hexanes/EtOAc eluent) to afford a pure sample of alcohol **S41** (21.3 mg, ~1:1 mixture of diastereomers at the alcohol carbon) as a colorless oil. The remaining desired alcohol product coeluted with the undesired isomers.

TLC: R_f = 0.52 in 3:1 hexanes/EtOAc, stained blue with *p*-anisaldehyde.

Pure vinylcyclobutane 6. To a mixture of DMSO (0.28 mL) and CH_2Cl_2 (0.07 mL) at 0 °C under argon was added sulfur trioxide pyridine complex (27.5 mg, 0.173 mmol). The mixture was stirred for 10 min at 0 °C, then vinylcyclobutane **S41** (21.3 mg, 0.0691 mmol) and Et_3N (0.024 mL, 0.173 mmol) were added as a solution in CH_2Cl_2. The reaction mixture was stirred at 0 °C for 1.5 h, then was allowed to warm to ambient temperature and stirred for 10 h. At this time, the vinylcyclobutane (**S41**) was not yet consumed, so additional sulfur trioxide pyridine complex (27.5 mg, 0.173 mmol) and Et_3N (0.024 mL, 0.173 mmol) were added. The reaction was stirred for 3 d total. The reaction mixture was then diluted with H_2O (2 mL) and CH_2Cl_2 (2 mL). The layers were separated, and the aqueous layer was extracted with CH_2Cl_2 (2 x 5 mL). The combined organic layers were washed with brine (10 mL) and dried over Na_2SO_4. The volatile materials were then removed by rotary evaporation, and the residue was purified by flash chromatography (100% hexanes → 9:1 hexanes/EtOAc eluent) to afford pure vinylcyclobutane **6** (16.4 mg, 77% yield) as a colorless oil.

TLC: R_f = 0.66 in 3:1 hexanes/EtOAc, visualized by UV.

^{1}H NMR (400 MHz; $CDCl_3$): δ 7.96 (d, *J* = 7.6 Hz, 2H), 7.56 (t, *J* = 7.6 Hz, 1H), 7.45 (*J* = 7.6 Hz, 1H), 7.05 (d, *J* = 8.7 Hz, 2H), 6.82 (d, *J* = 8.7 Hz, 2H), 6.09 (dd, *J* = 17.6, 10.4 Hz, 1H), 5.04 (d, *J* = 1.9 Hz, 1H), 5.00 (dd, *J* = 4.0, 1.9 Hz, 1H), 4.24 (q, *J* = 9.7 Hz, 1H), 3.95 (d, *J* = 9.7 Hz, 1H), 3.78 (s, 3H), 2.30 (t, *J* = 10.0 Hz, 1H), 2.17 (t, *J* = 10.0 Hz, 1H), 1.03 (s, 3H).

^{13}C NMR (100 MHz; $CDCl_3$): δ 200.4, 158.1, 147.1, 136.1, 133.0, 131.4, 128.6, 128.4, 128.3, 113.5, 111.4, 55.2, 48.3, 41.8, 40.9, 36.7, 20.5.

IR (ATR, neat): 2963, 2832, 1674, 1512, 1242, 1033, 732, 694 cm^{-1}.

HRMS (ESI+): *m/z* calc'd for $(M + H)^+$ $[C_{21}H_{22}O_2 + H]^+$: 307.1693, found 307.1693.

The stereochemistry of vinylcyclobutane **6** was confirmed through 1D NOESY NMR.

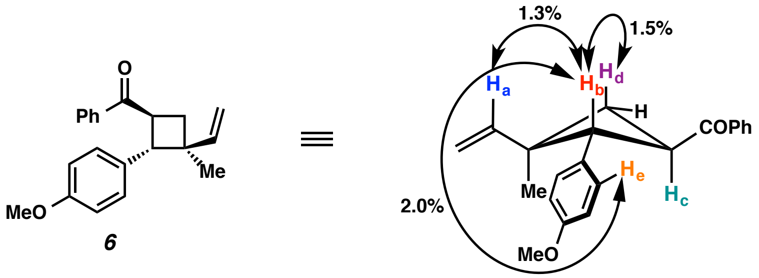

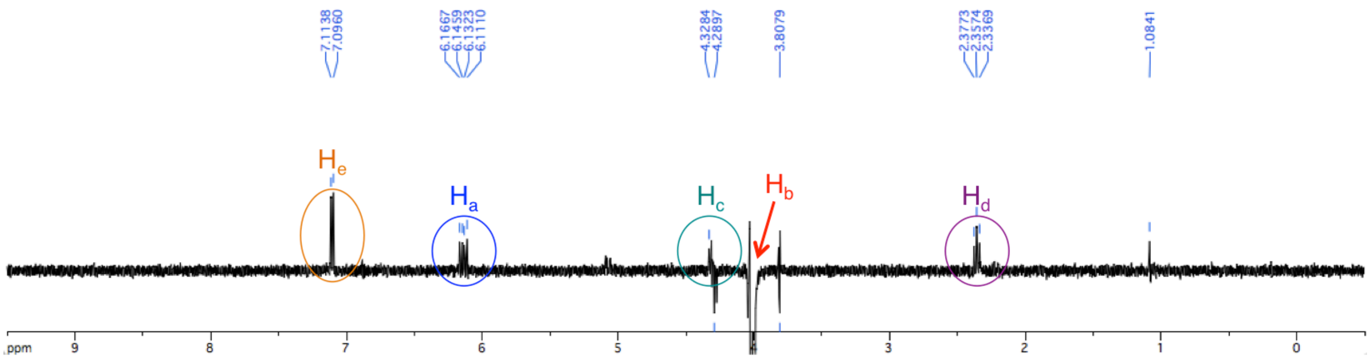

Figure S5. 1D NOESY of vinylcyclobutane **6**.

Structural and Stereochemical Determination of 1-Substituted Diene Products

The major constitutional isomer of cyclohexene **22** was determined through COSY and HMQC NMR. The COSY NMR (Figure S6) revealed that protons H_a and H_b are coupled to each other, as are H_c and H_d.

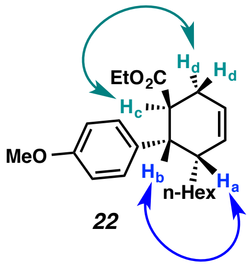

22

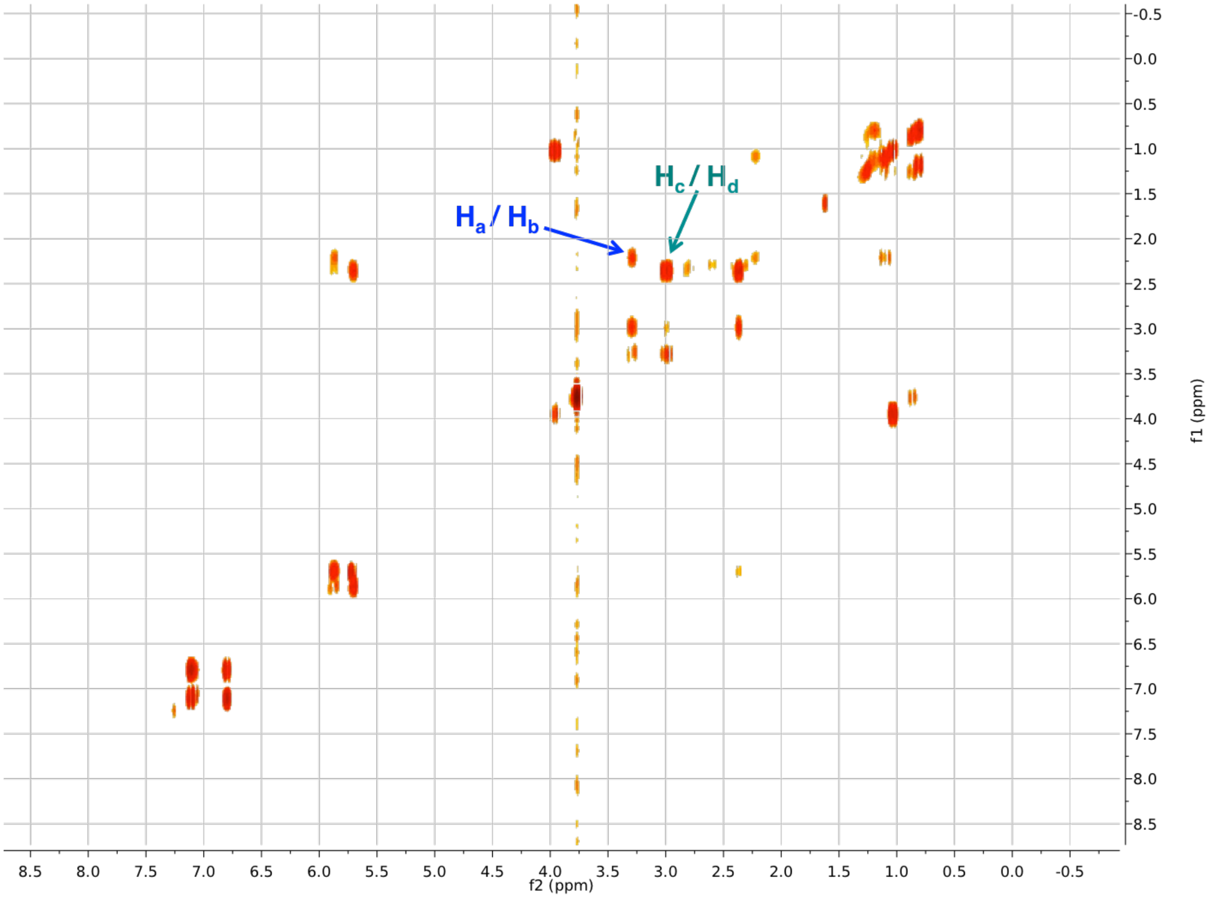

Figure S6. COSY NMR of cyclohexene **22**.

HMQC NMR was used to confirm that the proton peak at 2.37 ppm with an integration of 2 was a result of just the H_d protons and not a mix of H_d and H_c. The HMQC spectrum (Figure S7) indicated that the protons represented by that peak were both bonded to the same carbon atom, thus further solidifying our structural assignment of cyclohexene **22**.

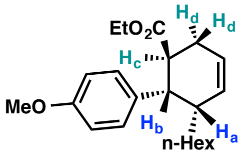

22

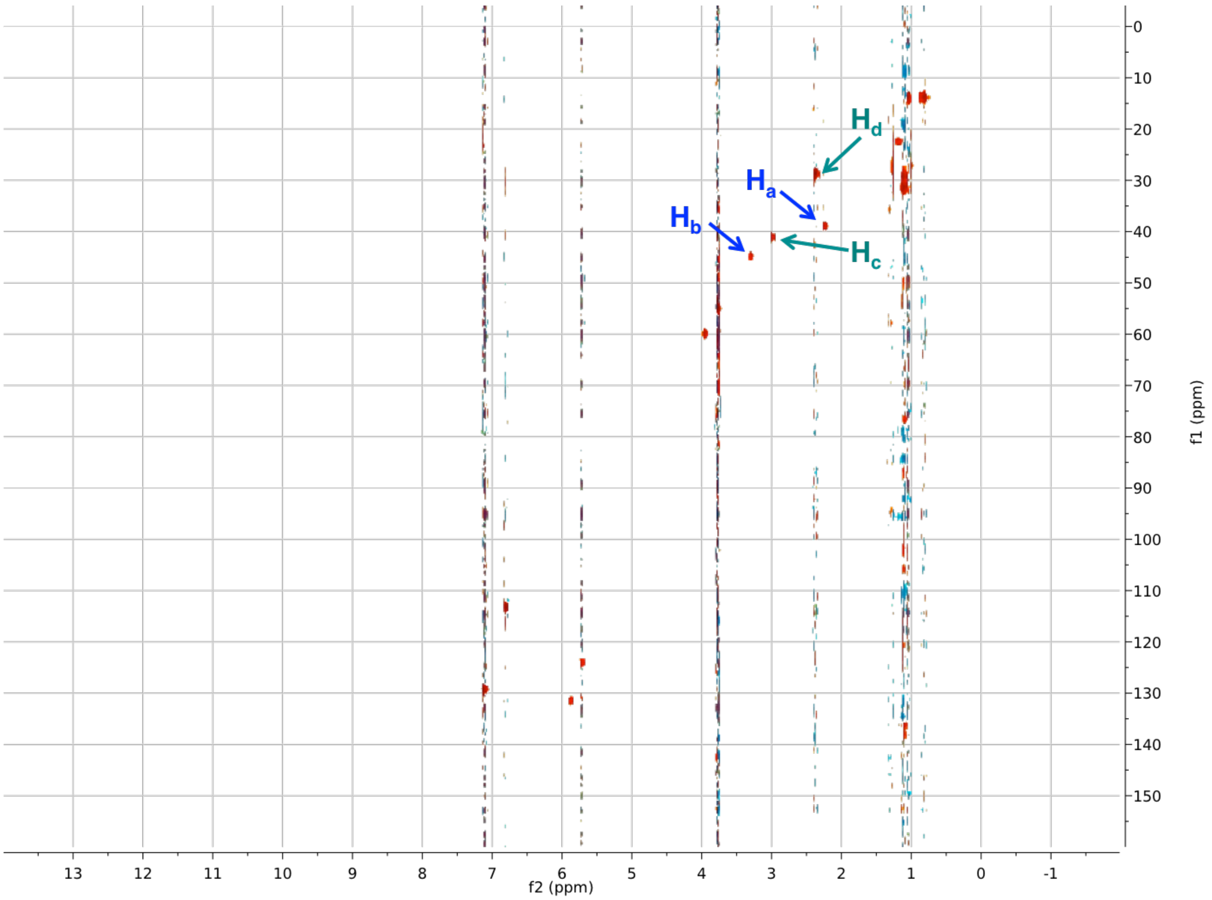

Figure S7. HMQC NMR of cyclohexene **22**.

Decoupling Experiment

The stereochemistry of the 1-substituted diene products was determined through structural modification of cyclohexene **24** and a ^{1}H NMR decoupling experiment.

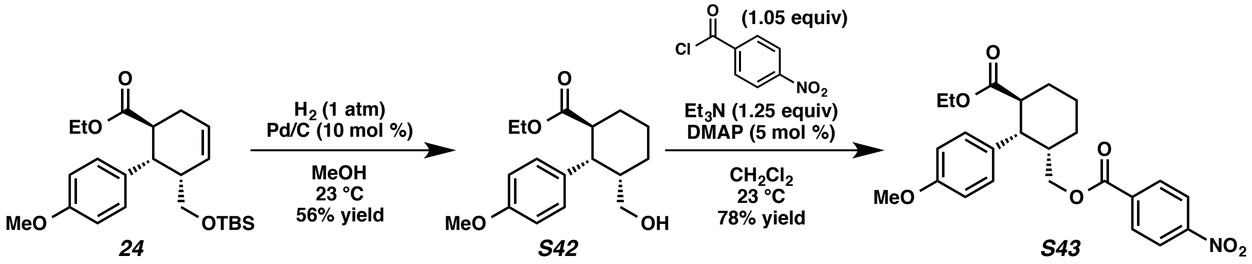

Cyclohexane S42. To a solution of cyclohexene **24** (37.6 mg, 93.0 µmol) in MeOH (0.900 mL) at 23 °C was added Pd/C (10% w/w, 9.9 mg, 9.30 µmol). The flask was sealed with a rubber septum, and H_2 was bubbled through the solution for 1 min using a balloon and needle outlet. The needle outlet was removed, and the reaction mixture was left under positive pressure of H_2 and stirred for 9 h. The crude reaction mixture was passed through a short plug of celite (Et_2O eluent), and the solvent was removed by rotary evaporation. The resulting residue was purified by flash chromatography (100% hexanes → 2:1 hexanes/EtOAc eluent) to afford cyclohexane **S42** (15.3 mg, 56% yield) as a colorless oil.

TLC: R_f = 0.16 in 3:1 hexanes/EtOAc, stained with $KMnO_4$.

^{1}H NMR (400 MHz; $CDCl_3$): δ 7.09 (d, *J* = 8.4 Hz, 2H), 6.81 (d, *J* = 8.4 Hz, 2H), 3.93 (q, *J* = 7.1 Hz, 2H), 3.76 (s, 3H), 3.54-3.47 (comp. m, 2H), 3.17 (dd, *J* = 11.7, 4.2 Hz, 1H), 2.90 (td, *J* = 11.7, 2.8 Hz, 1H), 2.07-1.99 (comp. m, 2H), 1.65-1.53 (comp. m, 4H), 1.01 (t, *J* = 7.1 Hz, 3H).

Nitrobenzoate S43. To a solution of cyclohexane **S42** (15.3 mg, 52.3 µmol) and 4-nitrobenzoyl chloride (10.2 mg, 55.0 µmol) in CH_2Cl_2 (0.500 mL) at 23 °C was added triethylamine (9.0 µL, 65.0 µmol), then DMAP (0.3 mg, 2.60 µmol). The reaction mixture was stirred at ambient temperature for 16 h, then was diluted with CH_2Cl_2 (3.00 mL), and

the solution was washed with H_2O (2 x 3.00 mL) and dried over Na_2SO_4. The solvent was removed by rotary evaporation, and the crude residue was purified by flash chromatography (100% hexanes → 9:1 hexanes/EtOAc eluent) to afford nitrobenzoate **S43** (16.6 mg, 78% yield) as a colorless oil.

TLC: R_f = 0.50 in 9:1 hexanes/EtOAc, visualized by UV.

^{1}H NMR (400 MHz; $CDCl_3$): δ 8.21 (d, *J* = 8.5 Hz, 2H), 7.97 (d, *J* = 8.5 Hz, 2H), 7.11 (d, *J* = 8.5 Hz, 2H), 6.78 (d, *J* = 8.5 Hz, 2H), 4.41 (t, *J* = 11.0 Hz, 1H), 4.12 (dd, *J* = 11.0, 5.3 Hz, 1H), 3.95 (q, *J* = 7.1 Hz, 2H), 3.71 (s, 3H), 3.27 (dd, *J* = 11.5, 4.6 Hz, 1H), 2.99 (td, *J* = 11.5, 3.3 Hz, 1H), 2.45-2.41 (m, 1H), 2.15-1.96 (comp. m, 2H), 1.75-1.49 (comp. m, 4H), 1.03 (t, *J* = 7.1 Hz, 3H).

Decoupling of proton H_c (2.45 ppm) of cyclohexane **S43** resulted in peak H_b changing from a doublet of doublets (*J* = 11.5, 4.6 Hz) to just a doublet (*J* = 11.5 Hz) (Figure S8). From this data, we can conclude that the coupling constant between H_a and H_b is 11.5 Hz, corresponding to an anti relationship, and the coupling constant between H_b and H_c is 4.6 Hz, corresponding to a syn relationship.

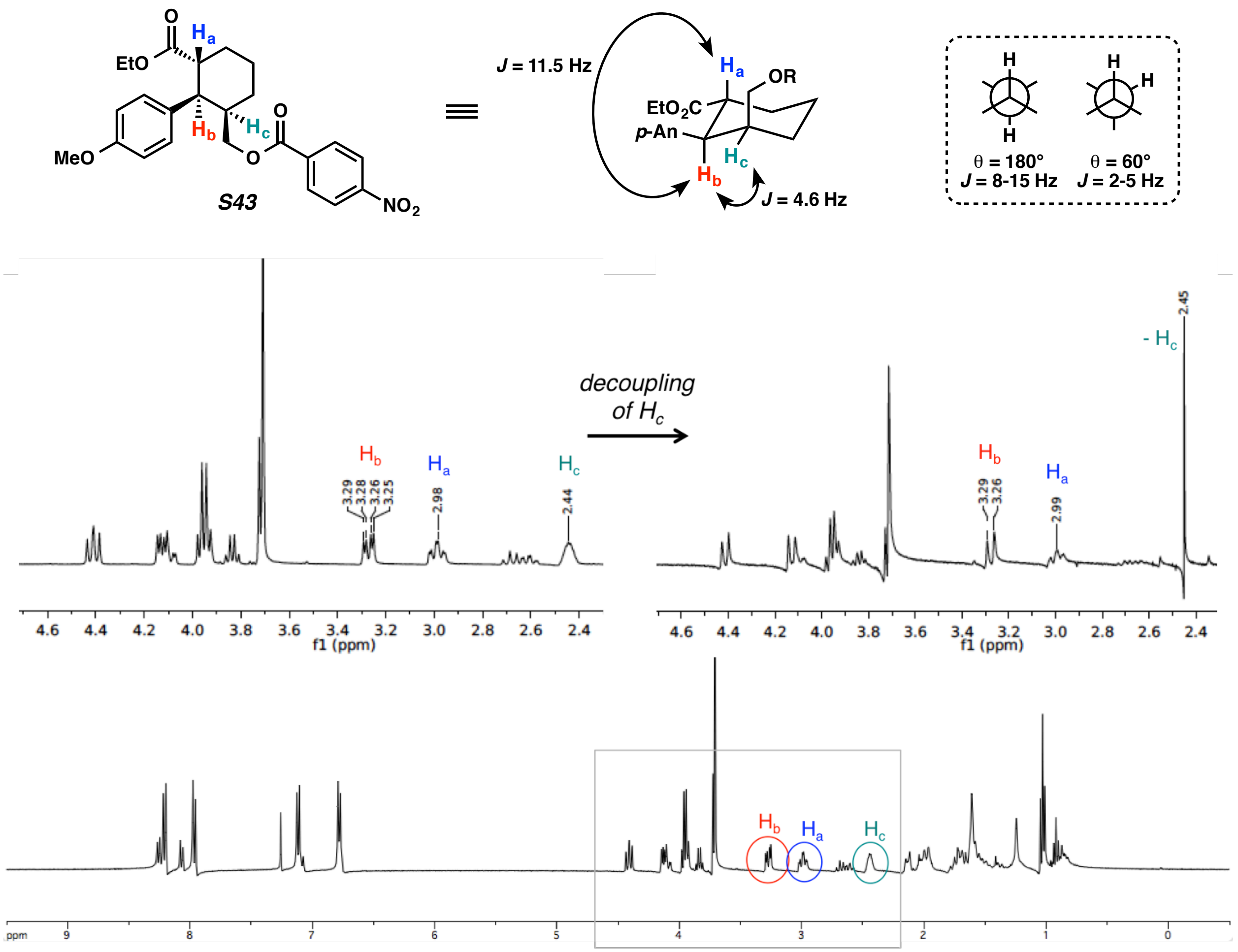

Figure S8. ^{1}H NMR decoupling experiment on cyclohexane **S43.**

Identification of Minor Isomer

In all cases, the minor isomer was assigned as the constitutional isomer. For the 2-substituted diene products, this was determined by comparing the ^{1}H NMR spectra of several of the products obtained with the Cr conditions to those obtained through traditional Diels-Alder conditions (i.e., **14** vs. **34**); the remaining cyclohexene products were assigned by analogy.

In the cases of the 1-substituted diene products, the minor isomer was assigned based on the splitting pattern and coupling constants of the peaks for the benzylic proton (H_a) and the proton alpha to the ester (H_b) in the ^{1}H NMR spectrum of cyclohexene **22** (Figure S9).

The peaks of the major and minor isomer were assigned with the benzylic proton (H_a) being the farther downfield peak and the proton alpha to the ester (H_b) being the more upfield peak. This trend is observed in all of the ester cyclohexene products. These two protons are presumed anti to each other because of their large coupling constant (J = 10.4 Hz). Throughout this research, we have only ever observed an anti relationship in the product between the two substituents of the starting alkene of the dienophile.

The peak of the benzylic proton (H_a) of the minor isomer is a triplet of doublets, indicating that H_a is being split by 3 other protons; the peak of the proton alpha to the ester (H_b) of the minor isomer is a triplet, indicating that H_b is being split by 2 other protons. This assessment is aligned with the minor product being the constitutional isomer (**S44**). This minor isomer was formed in too small of an amount for its stereochemistry to be determined.

Major isomer ***Minor isomer***

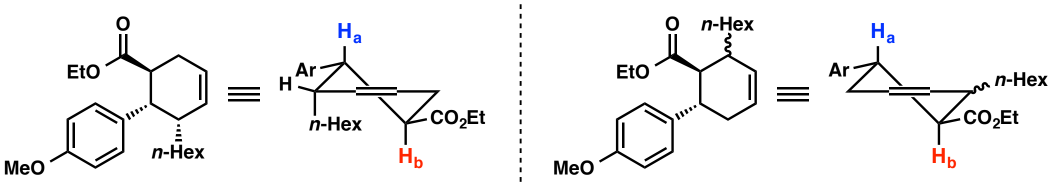

22

H	shift (ppm)	splitting	*J* (Hz)
H_a	3.29	dd	10.4, 5.4
H_b	2.99	dt	10.4, 7.6

S44

H	shift (ppm)	splitting	*J* (Hz)
H_a	2.80	td	11.2, 5.1
H_b	2.60	t	11.2

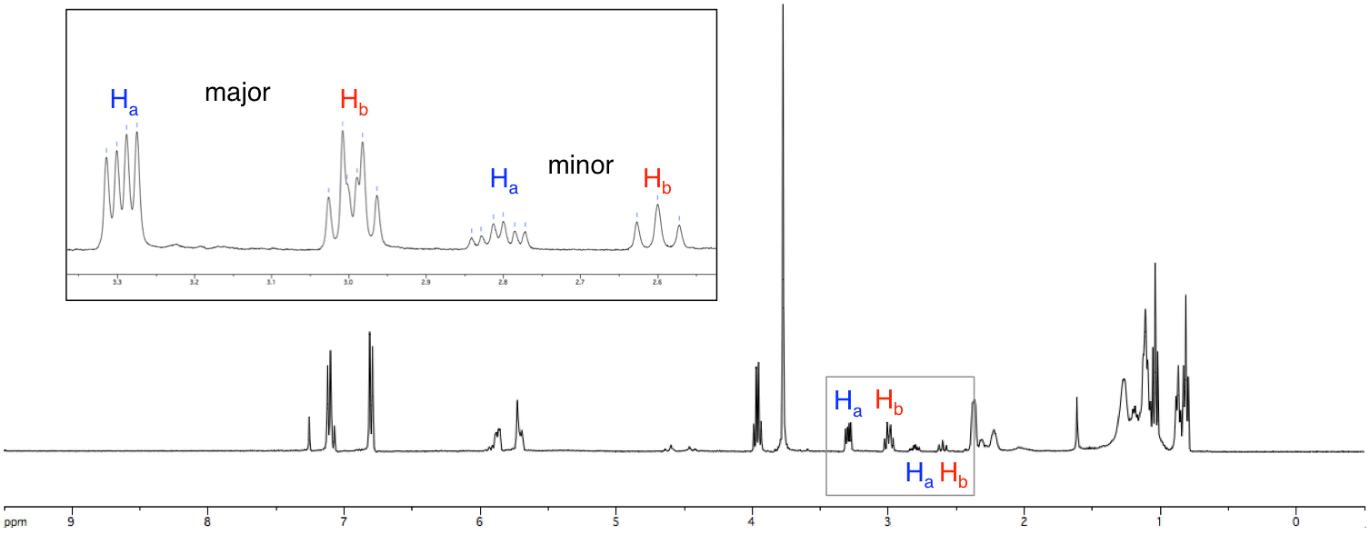

Figure S9. Identification of minor isomer **S44**.

The other possible minor product, if not a constitutional isomer, would be the diastereomer, cyclohexene **S45** (Figure S10). This diastereomer is likely not the minor product, since the coupling constant between H_a and H_c is 5.1 Hz, which is small and not indicative of an anti relationship, as would be required in the exo diastereomer.

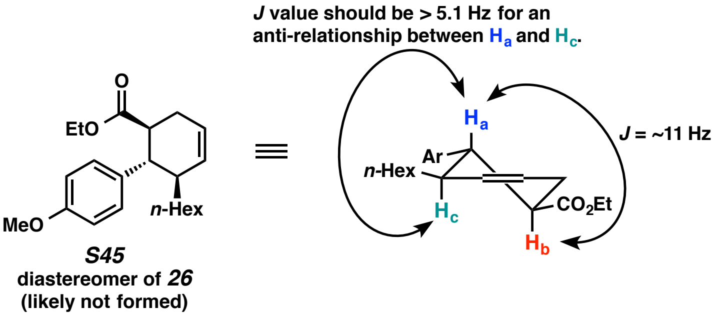

Figure S10. Evaluation of the diastereomer **S45** as possible minor isomer.

Oxidative Cleavage of PMP Group

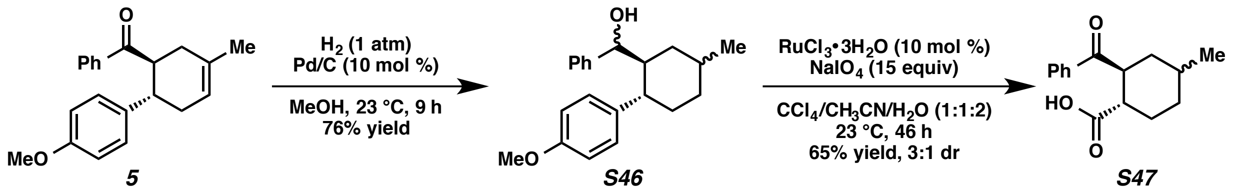

Cyclohexane S46. H_2 gas was bubbled through a mixture of cyclohexene **5** (60.0 mg, 0.200 mmol) and Pd/C (10% w/w, 21.3 mg, 0.0200 mmol) in MeOH (2.00 mL) for 30 s using a balloon of H_2 and needle outlet. The needle outlet was removed and the reaction mixture was left under positive pressure of H_2 and stirred for 9 h. The reaction mixture was then passed through a short plug of celite (2.0-2.5 x 1 cm, EtOAc eluent). The volatile materials were removed by rotary evaporation, and the crude product was purified by flash chromatography (100% hexanes → 15:1 hexanes/EtOAc eluent) to afford cyclohexane **S46** (47.0 mg, 76% yield) as a colorless oil. The NMR spectrum of the major diastereomer is reported.

TLC: R_f = 0.48 in 3:1 hexanes/EtOAc, stained blue with *p*-anisaldehyde.

^{1}H NMR (400 MHz; $CDCl_3$): δ 7.31-7.15 (m, 7H), 6.92 (d, *J* = 8.7 Hz, 2H), 4.61 (d, *J* = 4.1 Hz, 1H), 3.82 (s, 3H), 2.24 (tt, *J* = 11.7, 3.8 Hz, 1H), 2.03 (td, *J* = 11.7, 3.8 Hz, 1H), 1.94 (dq, *J* = 12.9, 2.5 Hz, 1H), 1.78-1.65 (comp. m, 2H), 1.56-1.24 (comp. m, 3H), 1.06 (d, *J* = 7.1 Hz, 1H), 0.88 (d, *J* = 6.5 Hz, 3H), 0.86-0.81 (m, 1H).

^{13}C NMR (100 MHz; $CDCl_3$): δ 157.9, 138.0, 128.5, 127.71, 127.65, 127.20, 127.17, 114.2, 75.3, 55.2, 48.3, 46.1, 37.7, 35.2, 35.0, 32.2, 22.7.

IR (film): 3386, 3031, 2920, 1611, 1512, 1249, 1036, 704 cm^{-1}.

HRMS (ESI+): *m/z* calc'd for $(M + Na)^+$ $[C_{21}H_{26}O_2 + Na]^+$: 333.1825, found 333.1824.

Carboxylic acid S47. To cyclohexane **S46** (10.4 mg, 0.0335 mmol) in CCl_4 (0.170 mL), acetonitrile (0.170 mL), and H_2O (0.340 mL) under argon was added $RuCl_3$•H_2O (0.9 mg, 0.00337 mmol), then $NaIO_4$ (108.2 mg, 0.506 mmol). The reaction mixture was stirred for 46 h, then was diluted with H_2O (2 mL) and CH_2Cl_2 (2 mL). The layers were

separated, and the aqueous layer was extracted with CH_2Cl_2 (2 x 5 mL). The combined CH_2Cl_2 layers were passed through a short plug of silica (EtOAc eluent). The volatile materials were removed by rotary evaporation, and the crude product was purified by flash chromatography (4:1 → 2:1 hexanes/EtOAc eluent) to afford carboxylic acid **S47** (5.4 mg, 65% yield) as a colorless oil. The NMR spectrum of the major diastereomer is reported.

TLC: R_f = 0.23 in 3:1 hexanes/EtOAc, visualized by UV.

^{1}H NMR (400 MHz; $CDCl_3$): δ 7.96-7.91 (m, 2H), 7.57-7.54 (m, 1H), 7.46 (t, *J* = 7.6 Hz, 2H), 3.63-3.56 (m, 1H), 2.92-2.85 (m, 1H), 2.22 (dd, *J* = 13.2, 3.5 Hz, 1H), 1.98-1.92 (m, 1H), 1.86-1.72 (comp. m, 2H), 1.62-1.46 (comp. m, 3H), 0.90 (d, *J* = 6.5 Hz, 3H).

^{13}C NMR (100 MHz; $CDCl_3$): δ 211.8, 202.6, 136.0, 132.9, 128.6, 128.4, 46.8, 43.8, 37.9, 34.1, 32.3, 29.1, 22.0.

IR (film): 3060, 2255, 1703, 1448, 1286, 700 cm^{-1}.

HRMS (ESI+): *m/z* calc'd for $(M + Na)^+$ $[C_{15}H_{18}O_3 + Na]^+$: 269.1148, found 269.1149.

Supporting Information References

[1] McDaniel, A. M.; Tseng, H.; Damrauer, N. H.; Shores, M. P. *Inorg. Chem*. **2010**, *49*, 7981-7991.

[2] Higgins, R. F.; Fatur, S. M.; Shepard, S. G.; Stevenson, S. M.; Boston, D. J.; Ferreira, E. M.; Damrauer, N. H.; Rappé, A. K.; Shores, M. P. *J. Am. Chem. Soc*. **2016**, *138*, 5451-5464.

[3] Cismesia, M. A.; Yoon, T. P. *Chem. Sci*. **2015**, *6*, 5426-5434.

[4] Stevenson, S. M.; Shores, M. P.; Ferreira, E. M. *Angew. Chem., Int. Ed*. **2015**, *54*, 6506-6510.

[5] Connelly, N. G.; Geiger, W. E. *Chem. Rev.* **1996**, *96*, 877-910.

[6] Vinylcyclobutane **S21** was prepared analogously to vinylcyclobutane **6**.

[7] Toda, F.; Tanaka, K.; Kato, M. *J. Chem. Soc., Perkin Trans. 1* **1998**, 1315-1318.

[8] Vaidya, S. S.; Vinaya, H.; Mahajan, S. S. *Med. Chem. Res.* **2012**, *21*, 4311-4323.

[9] Gao, F.; Feng, H.; Sun, Z. *Tetrahedron Lett*. **2014**, *55*, 6451-6454.

[10] Chen, X.; Zhou, H.; Zhang, K.; Li, J.; Huang, H. *Org. Lett.* **2014**, *16*, 3912-3915.

[11] Kelly, C. B.; Ovian, J. M.; Cywar, R. M.; Gosselin, T. R.; Wiles, R. J.; Leadbeater, N. E. *Org. Biomol. Chem*. **2015**, *13*, 4255-4259.

[12] Salabert, J.; Sebastian, R. M.; Vallribera, A.; Cívicos, J. F.; Najera, C. *Tetrahedron* **2013**, *69*, 2655-2659.

[13] Morales-Serna, J. A.; García-Ríos, E.; Bernal, J.; Paleo, E.; Gaviño, R.; Cárdenas, J. *Synthesis* **2011**, *9*, 1375-1382.

[14] Yang, H.; Fu, L.; Wei, L.; Liang, J.; Binks, B. P. *J. Am. Chem. Soc.* **2015**, *137*, 1362-1371.

[15] Patil, C. B.; Mahajan, S. K.; Katti, S. A. *J. Pharm. Sci. & Res*. **2009**, *1*, 11-22.

[16] Wu, J. Y.; Stanzl, B. N.; Ritter, T. *J. Am. Chem. Soc.* **2010**, *132*, 13214-13216.

[17] Lautens, M.; Tam, W.; Sood, C. *J. Org. Chem*. **1993**, 58, 4513-4515.

[18] Wu, J. Y.; Moreau, B.; Ritter, T. *J. Am. Chem. Soc.* **2009**, *131*, 12915-12917.

[19] Hioki, H.; Ooi, H.; Hamano, M.; Mimura, Y.; Yoshio, S.; Kodama, M.; Ohta, S.; Yanai, M.; Ikegami, S. *Tetrahedron* **2001**, *57*, 1235-1246.

[20] Llaveria, J.; Beltrán, Á.; Díaz-Requejo, M. M.; Matheu, M. I.; Castillón, S.; Pérez, P. J. *Angew. Chem., Int. Ed.* **2010**, *49*, 7092-7095.

[21] Pullin, R. D. C.; Sellars, J. D.; Steel, P. G. *Org. Biomol. Chem.* **2007**, *5*, 3201-3206.

[22] Hirner, J. J.; Blum, S. A. *Organometallics* **2011**, *30*, 1299-1302.

[23] Laird, T.; Ollis, W. D.; Sutherland, I. O. *J. Chem. Soc., Perkin Trans. 1* **1980**, 2033-2048.

Supporting Information - Spectra Compilation

Chromium Photocatalysis: Accessing Structural Complements to Diels-Alder Adducts with Electron-Deficient Dienophiles

Susan M. Stevenson,[a] Robert F. Higgins,[b] Matthew P. Shores,[b] and Eric M. Ferreira*[a]

[a]*Department of Chemistry, University of Georgia, Athens, GA 30602, United States*
[b]*Department of Chemistry, Colorado State University, Fort Collins, CO 80523, United States*

emferr@uga.edu

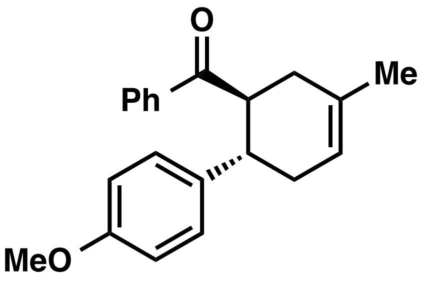

5
^{1}H NMR
(400 MHz, $CDCl_3$)

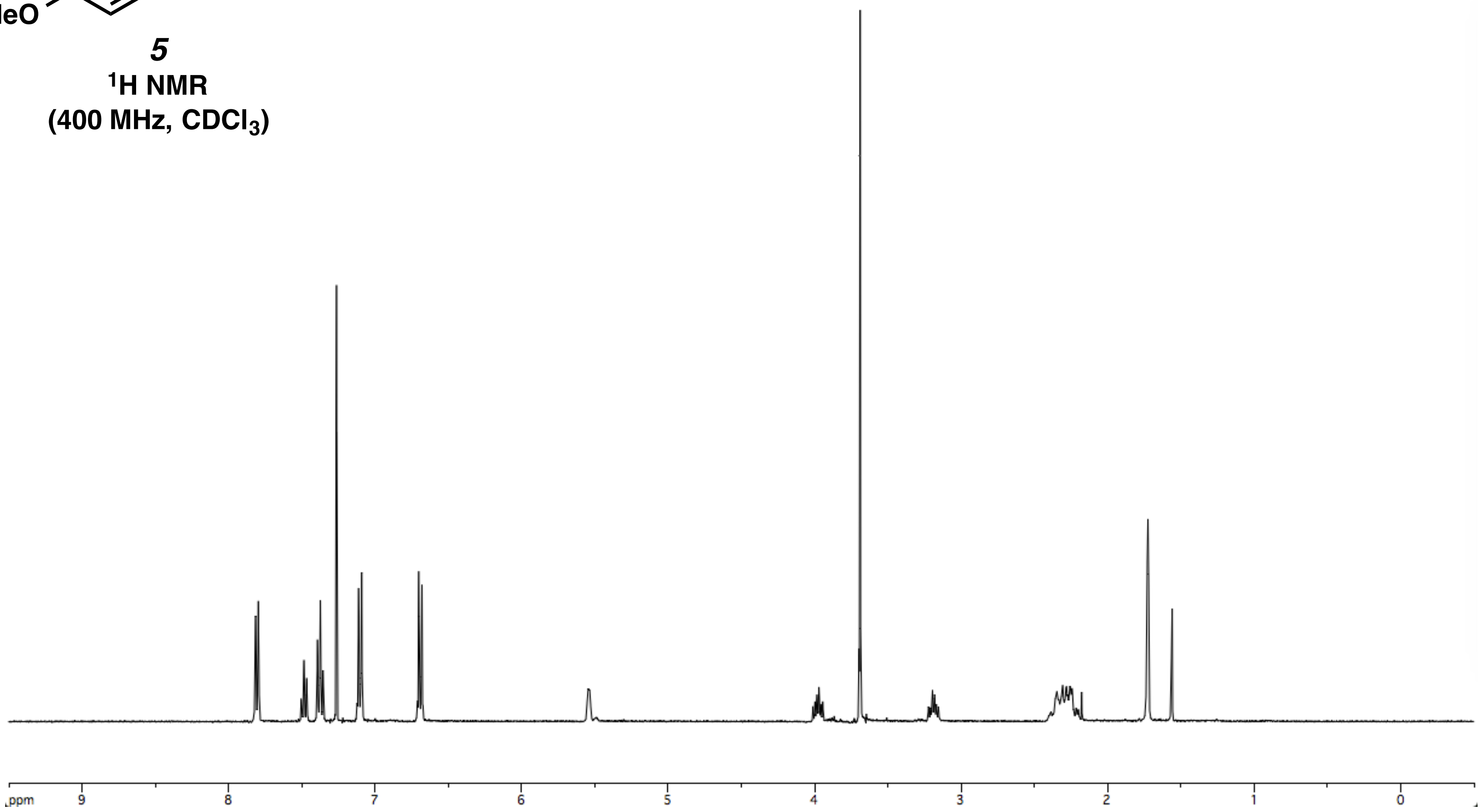

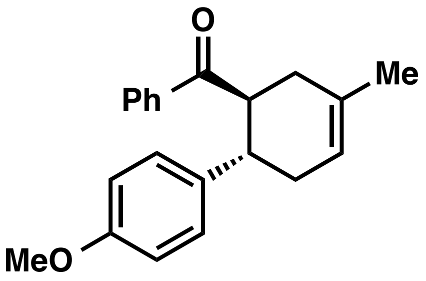

5

^{13}C NMR

(100 MHz, $CDCl_3$)

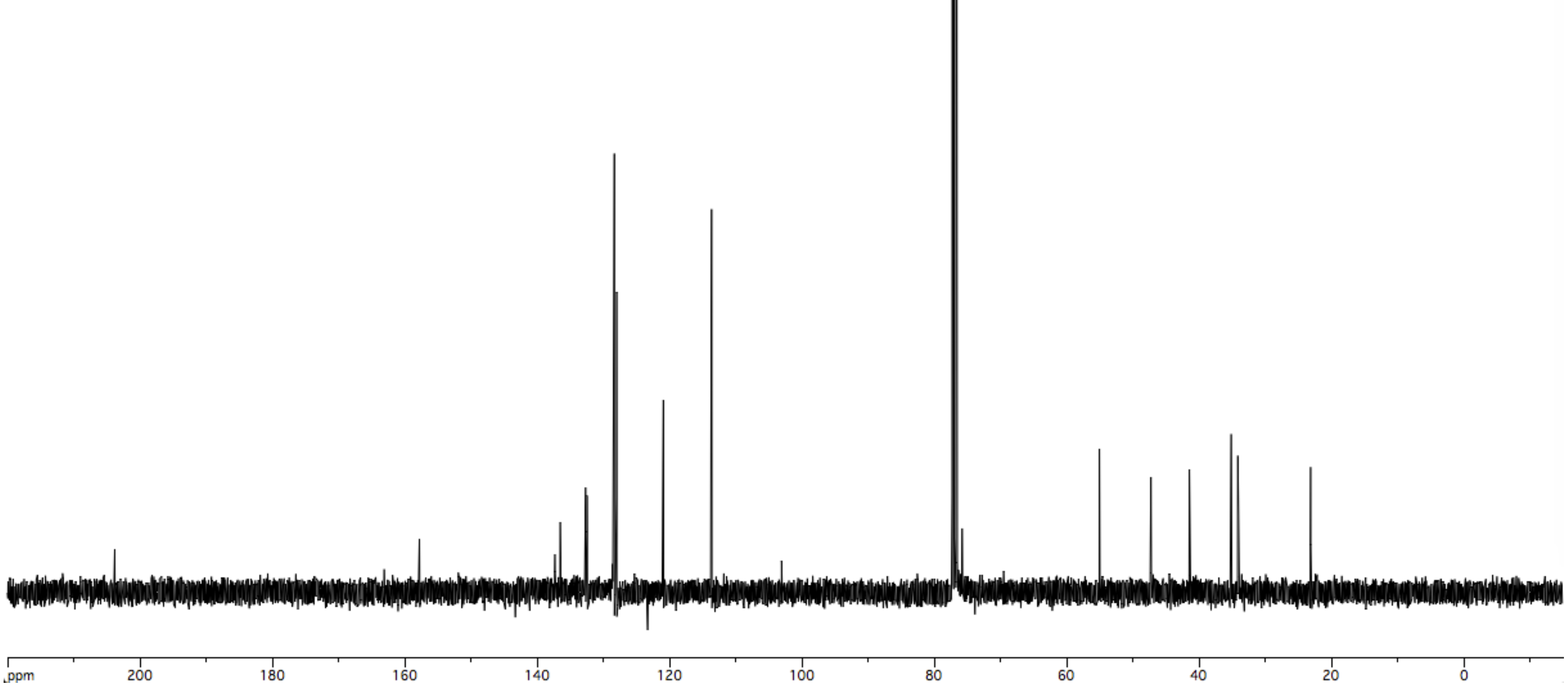

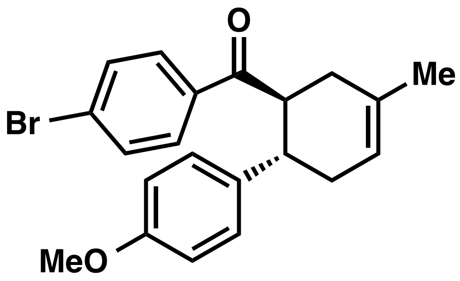

7

^{1}H NMR

(400 MHz, $CDCl_3$)

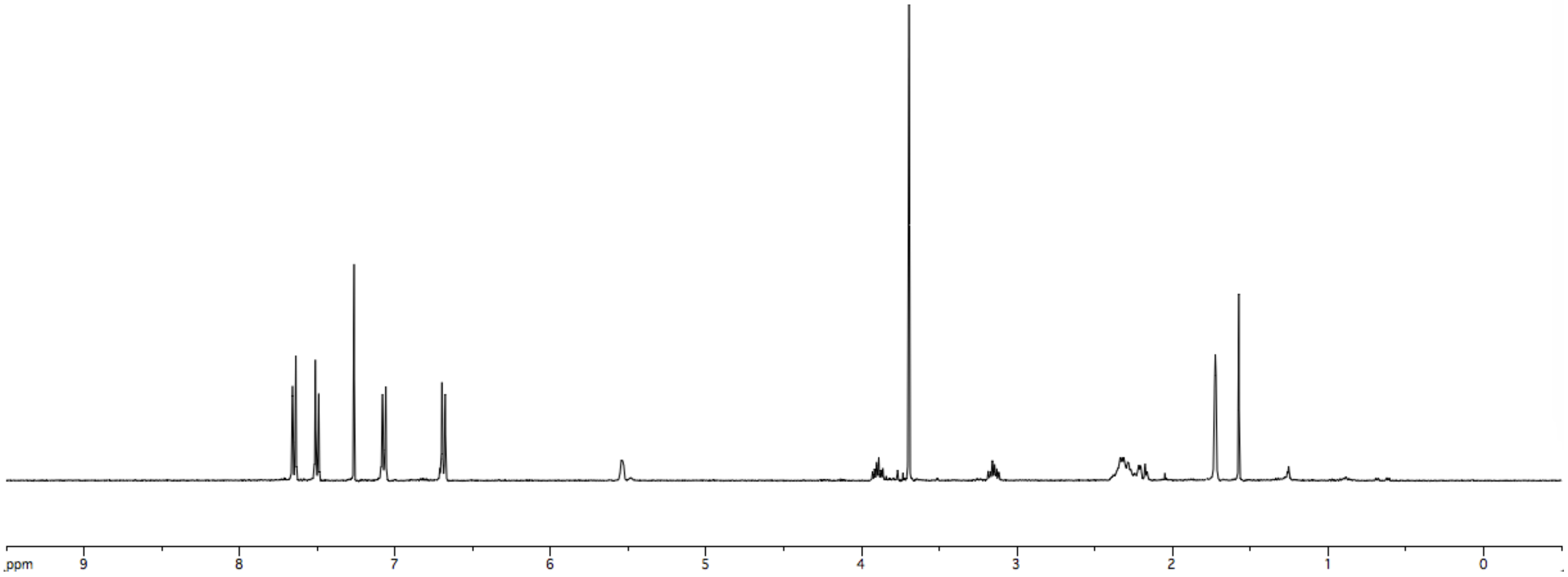

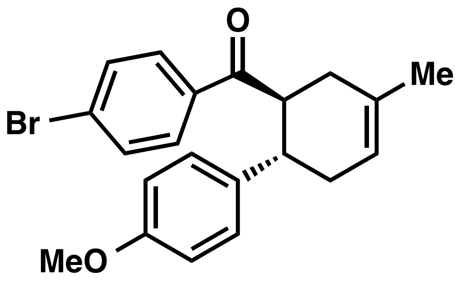

7

^{13}C NMR

(100 MHz, $CDCl_3$)

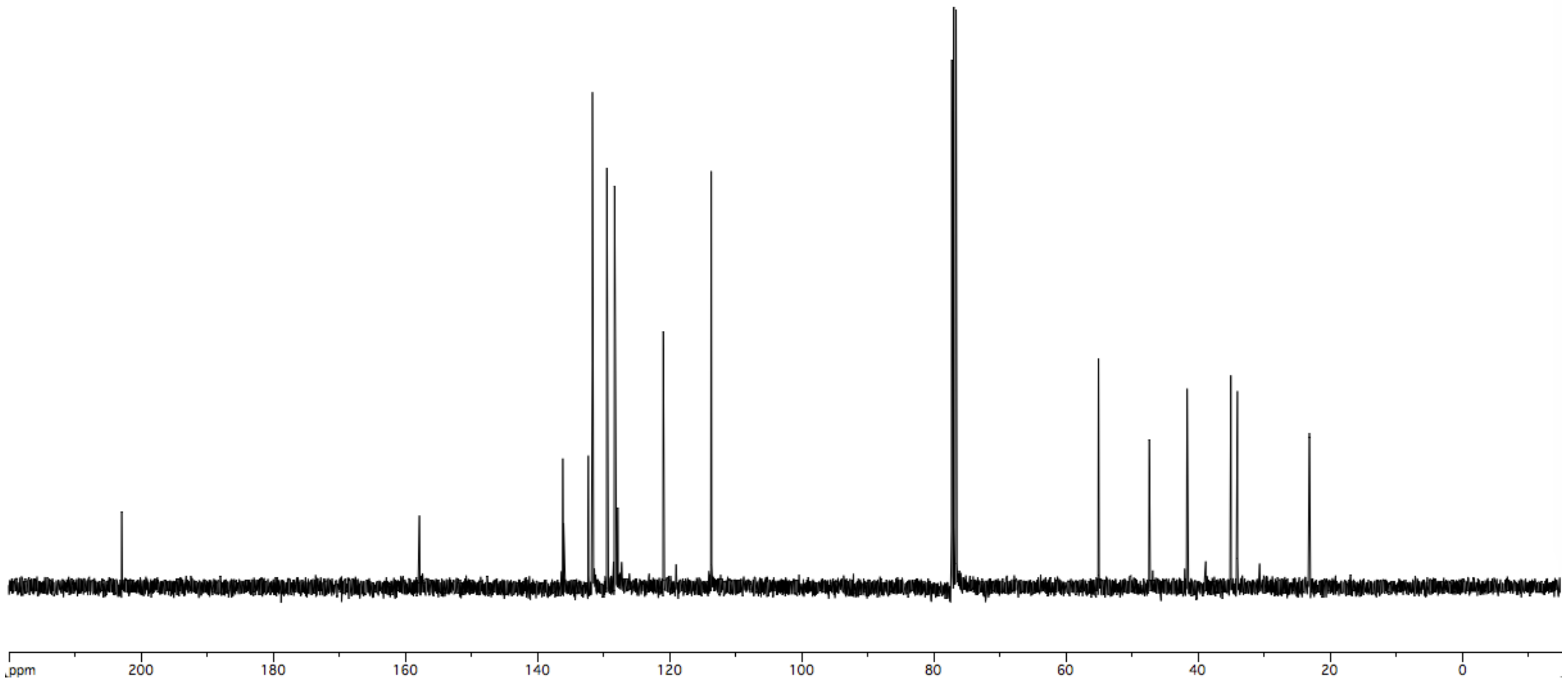

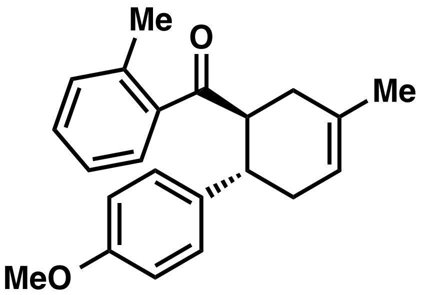

8

^{1}H NMR

(400 MHz, $CDCl_3$)

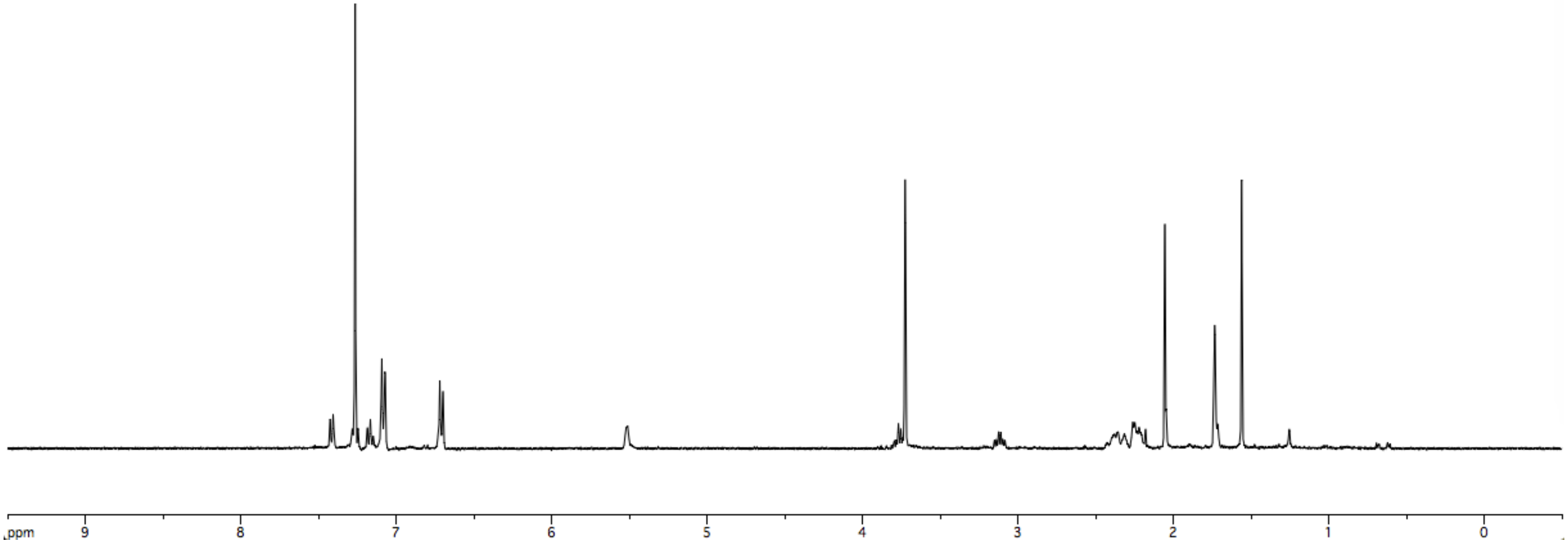

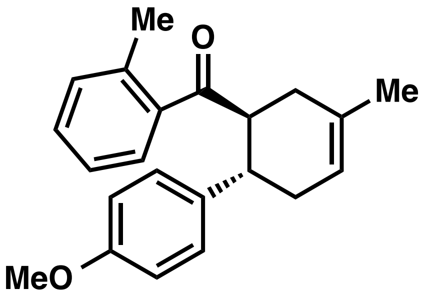

8

^{13}C NMR

(100 MHz, $CDCl_3$)

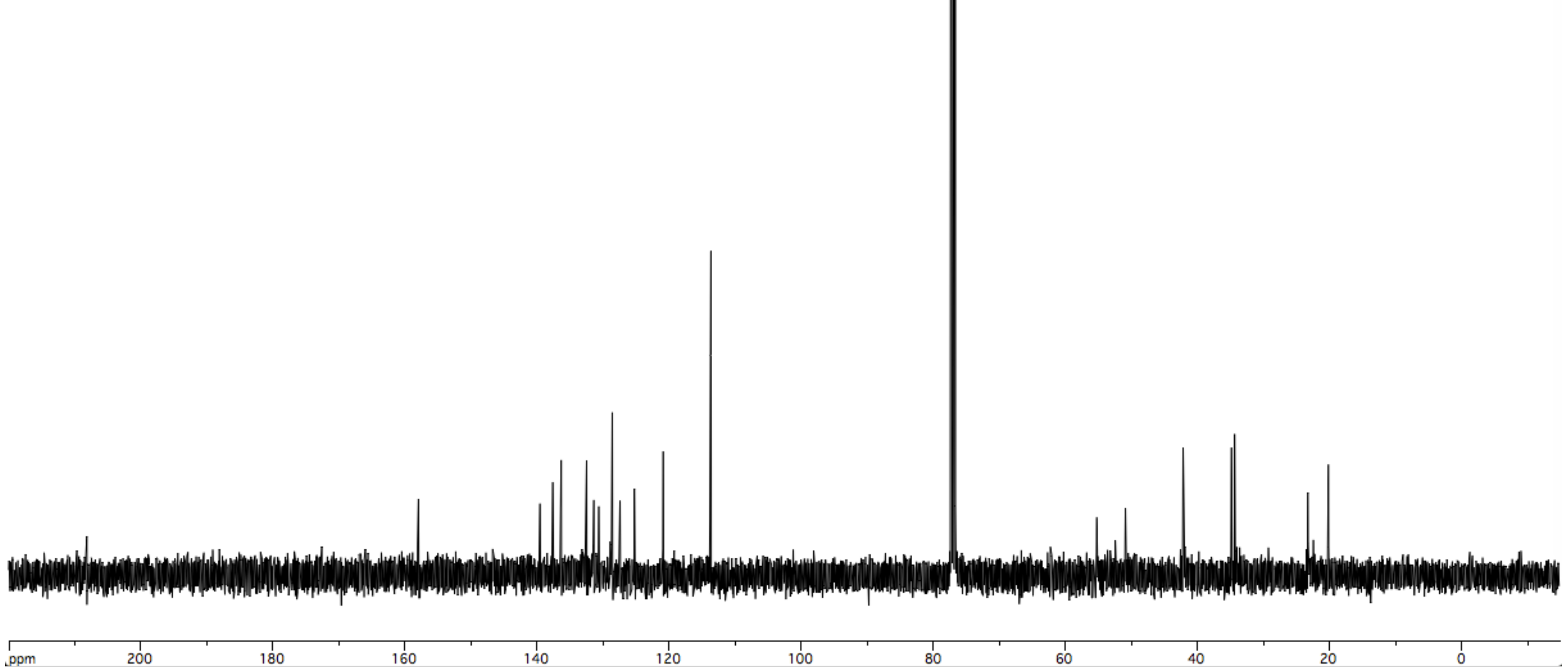

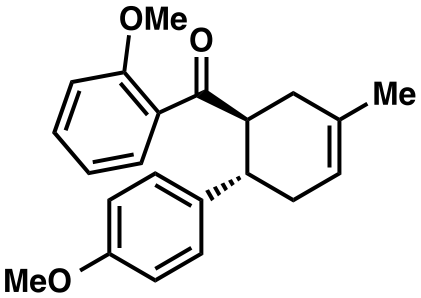

9

^{1}H NMR

(400 MHz, $CDCl_3$)

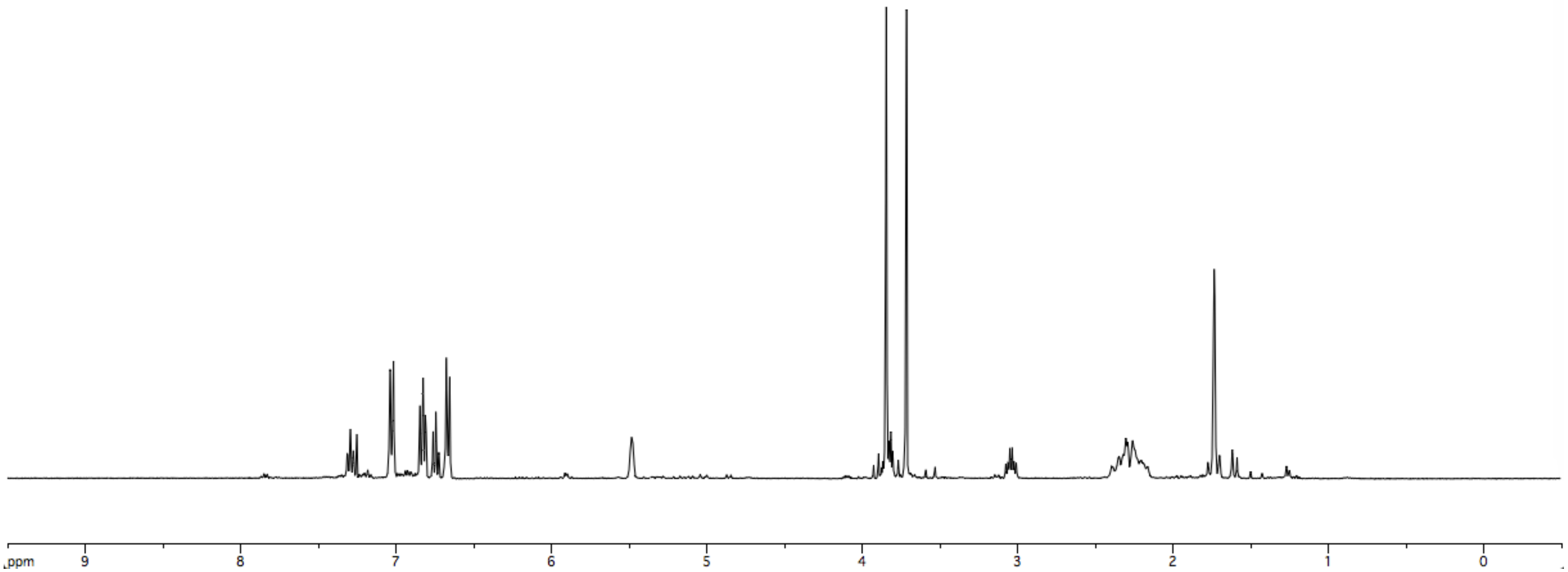

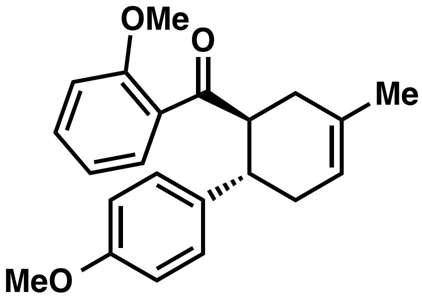

9
^{13}C NMR
(100 MHz, $CDCl_3$)

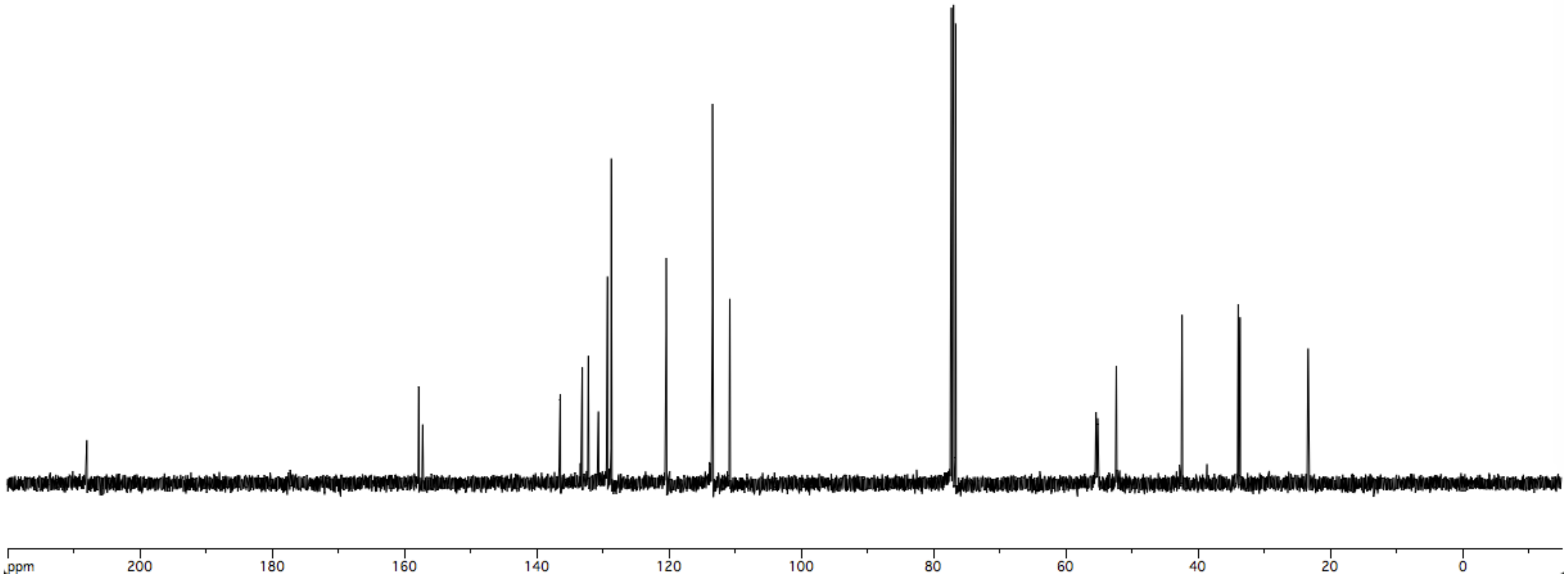

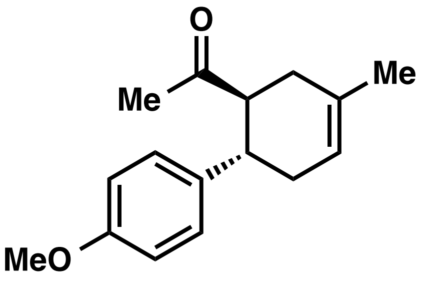

10

^{1}H NMR

(400 MHz, $CDCl_3$)

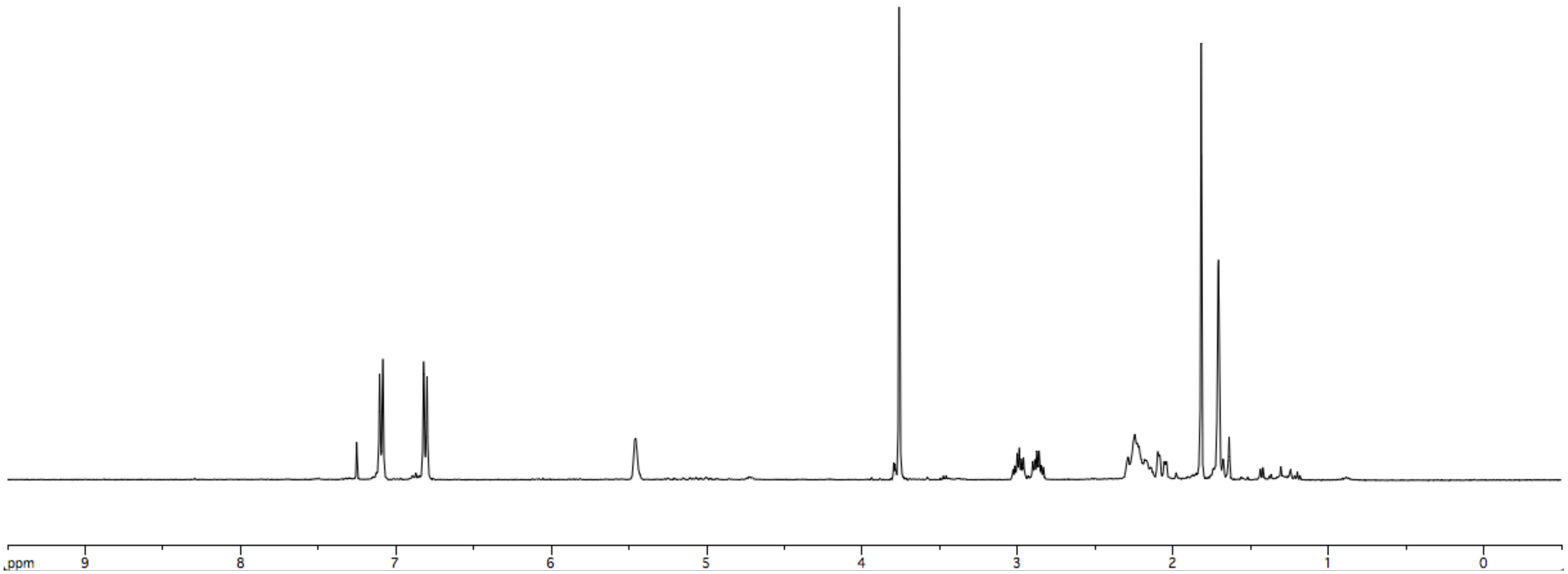

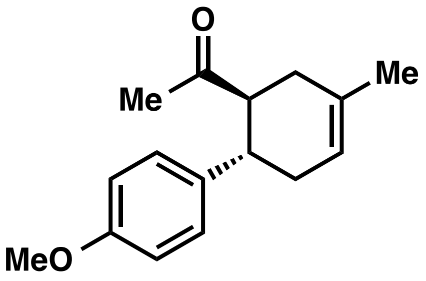

10

^{13}C NMR

(100 MHz, $CDCl_3$)

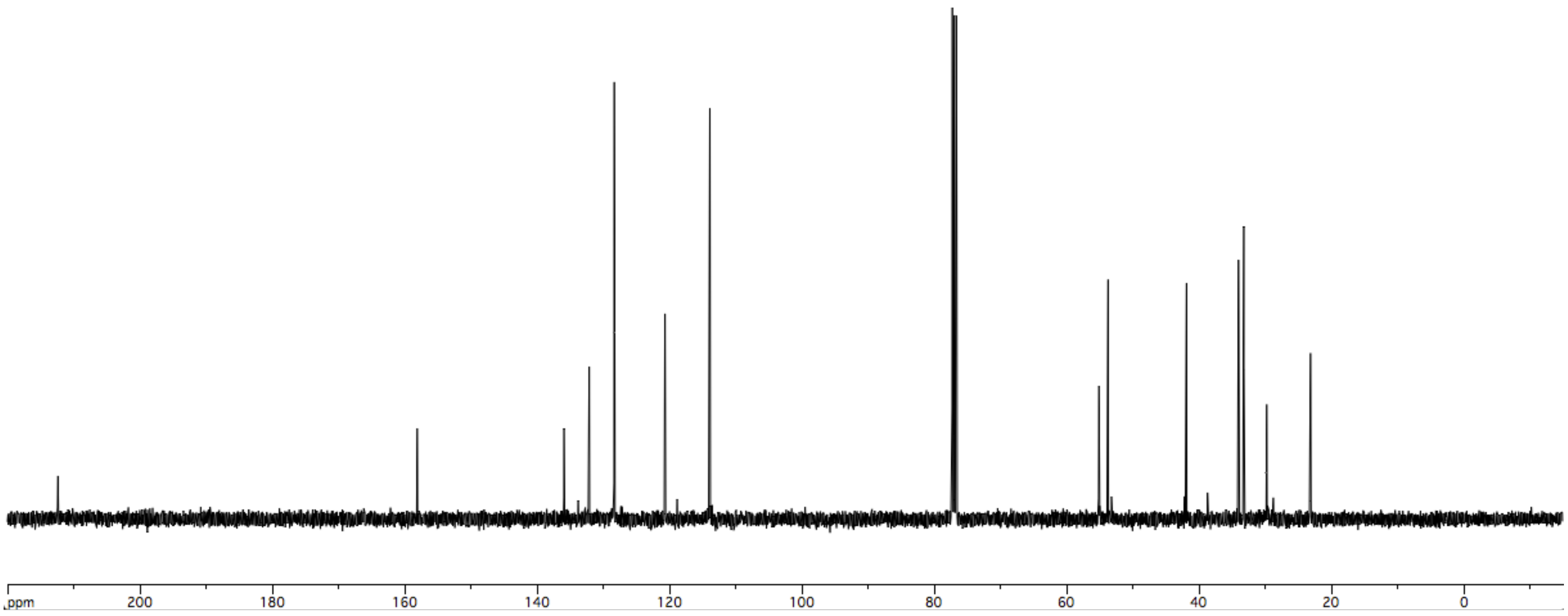

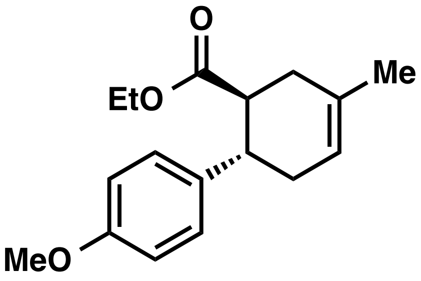

11

^{1}H NMR

(400 MHz, $CDCl_3$)

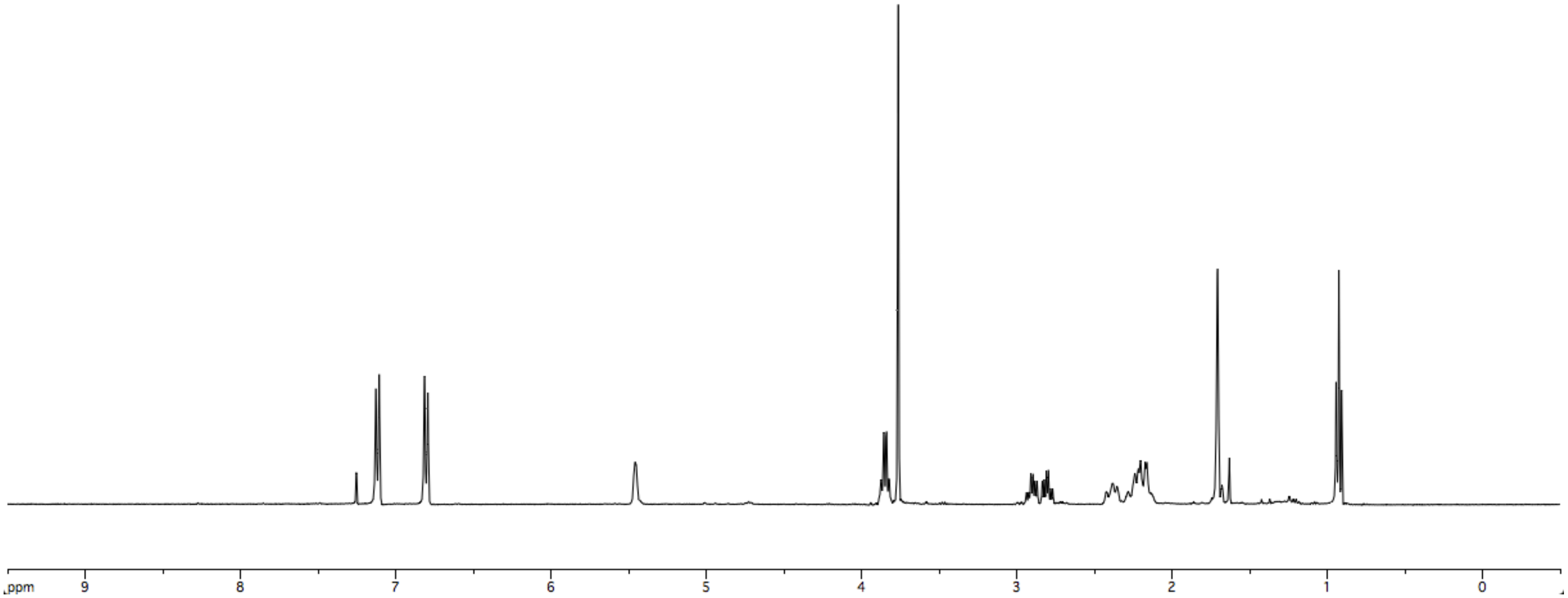

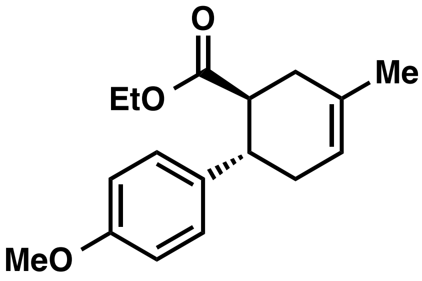

11

^{13}C NMR
(100 MHz, $CDCl_3$)

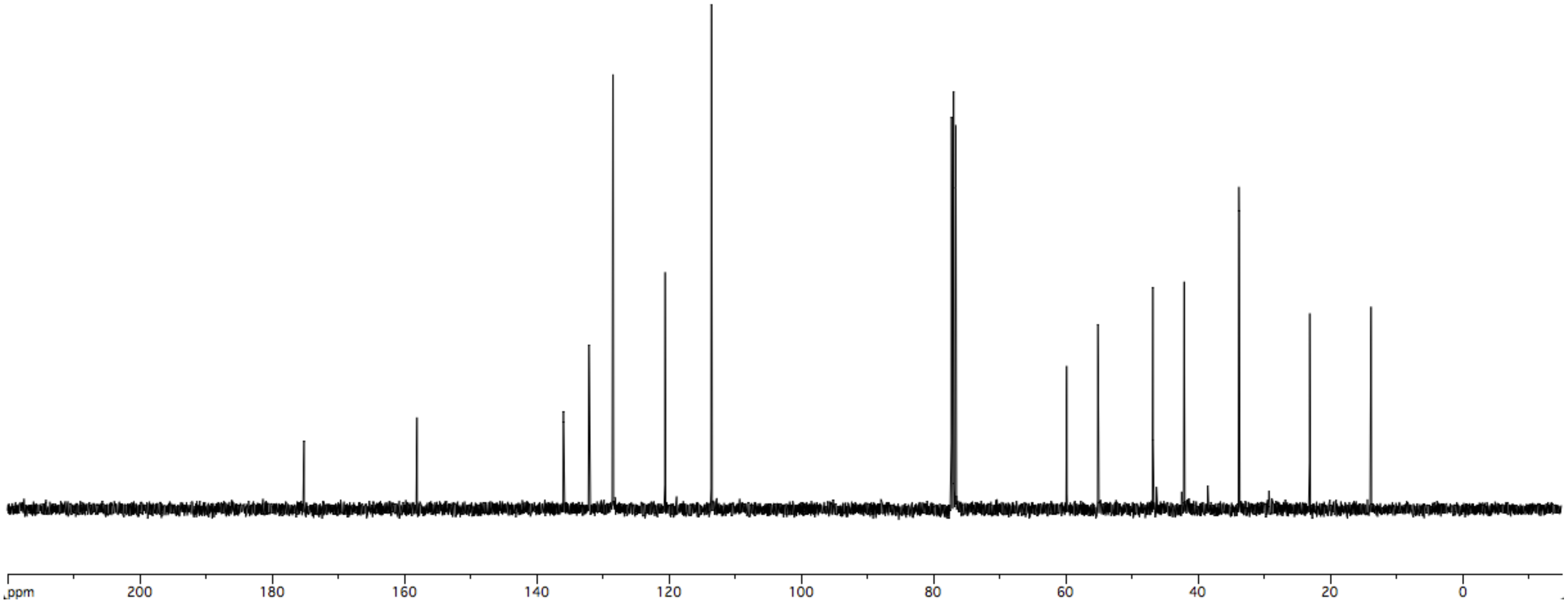

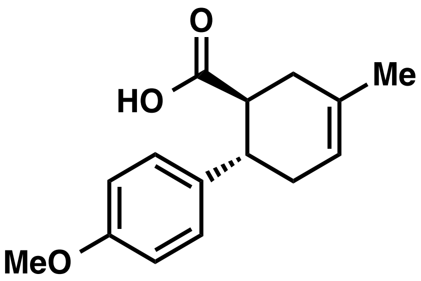

12
^{1}H NMR
(400 MHz, $CDCl_3$)

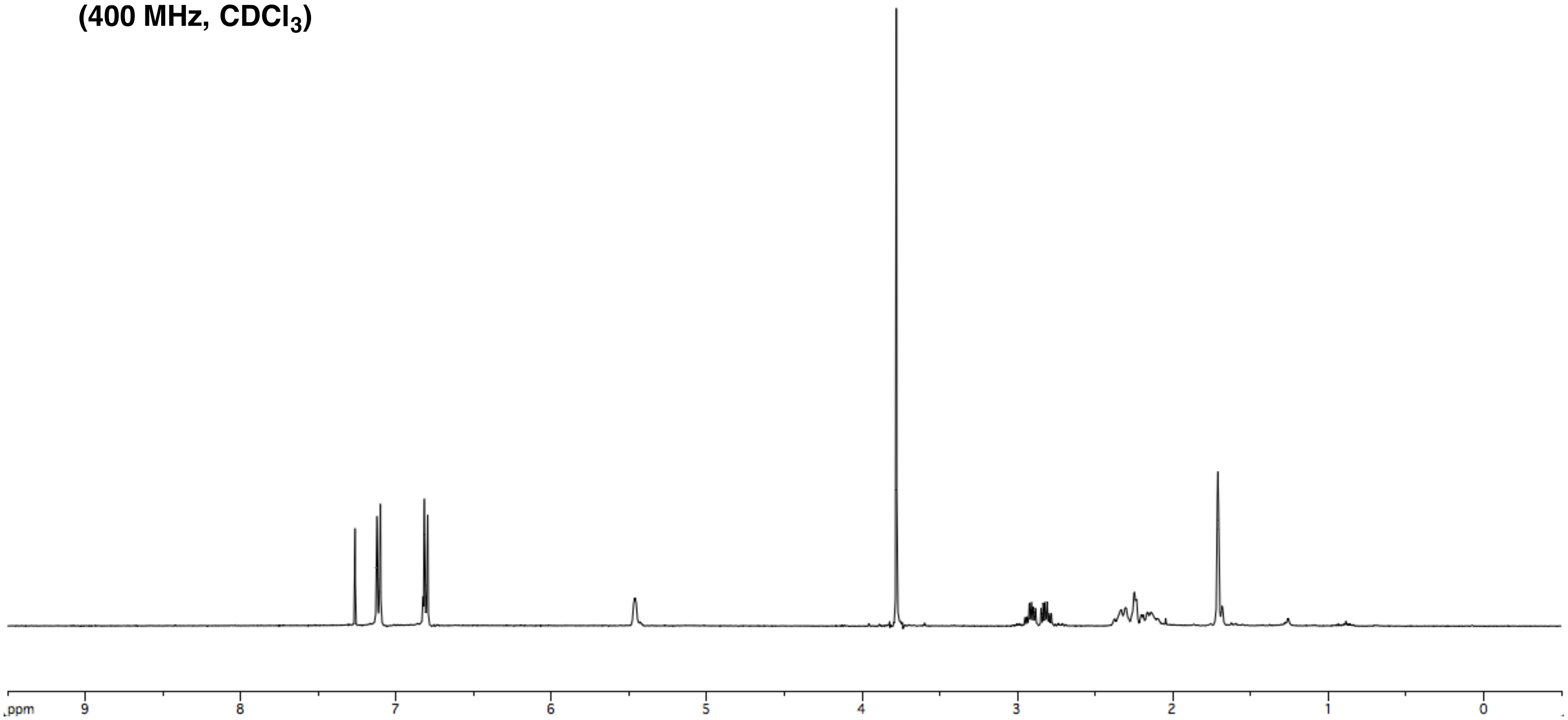

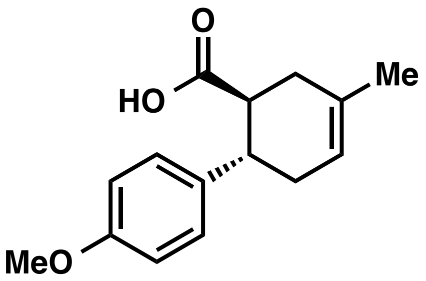

12

^{13}C NMR

(100 MHz, $CDCl_3$)

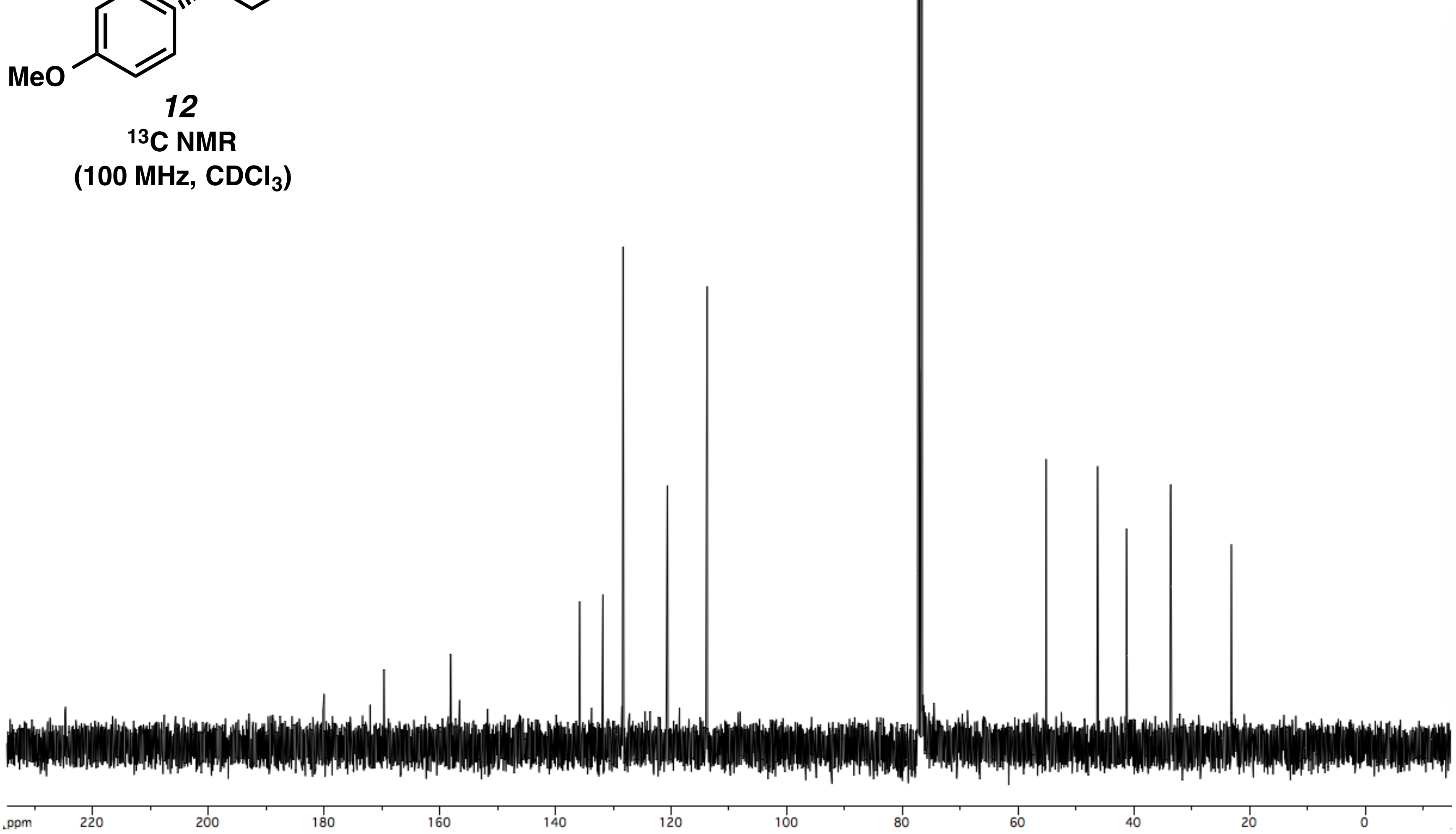

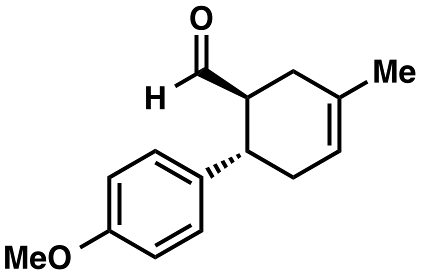

13
^{1}H NMR
(400 MHz, $CDCl_3$)

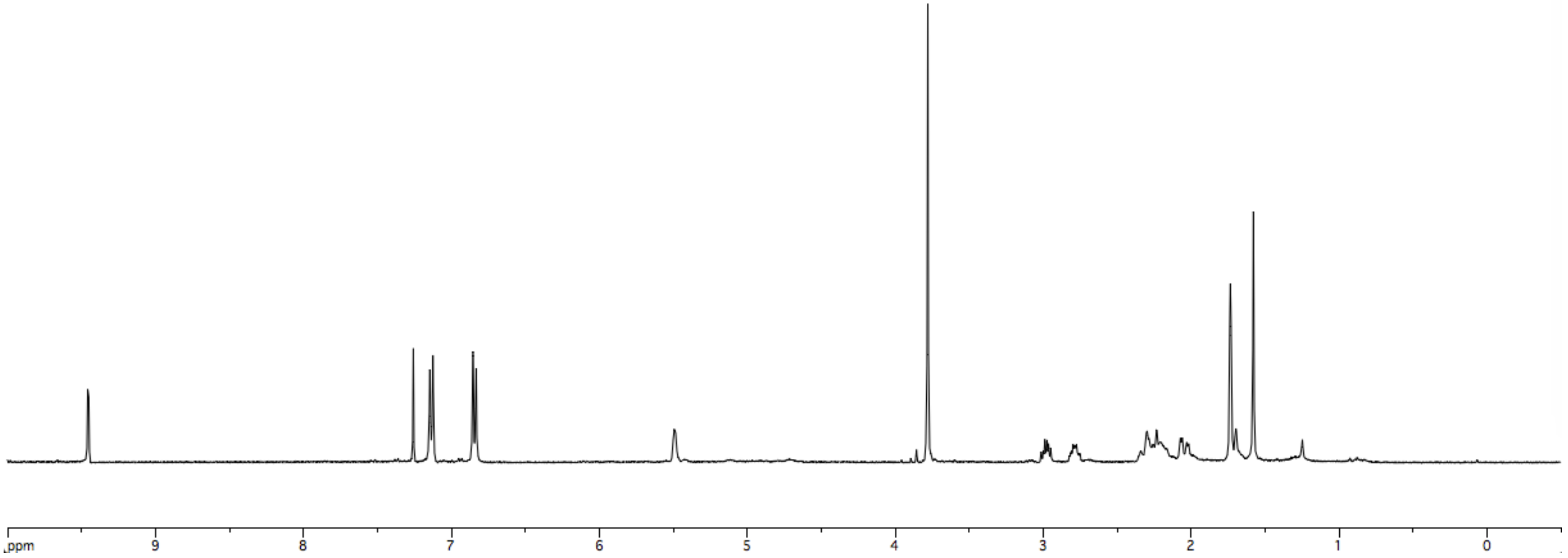

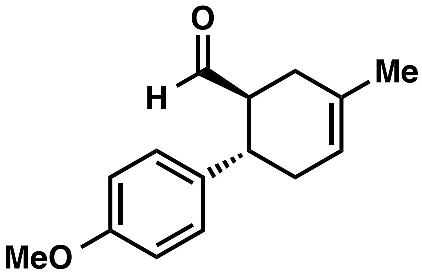

13
^{13}C NMR
(100 MHz, $CDCl_3$)

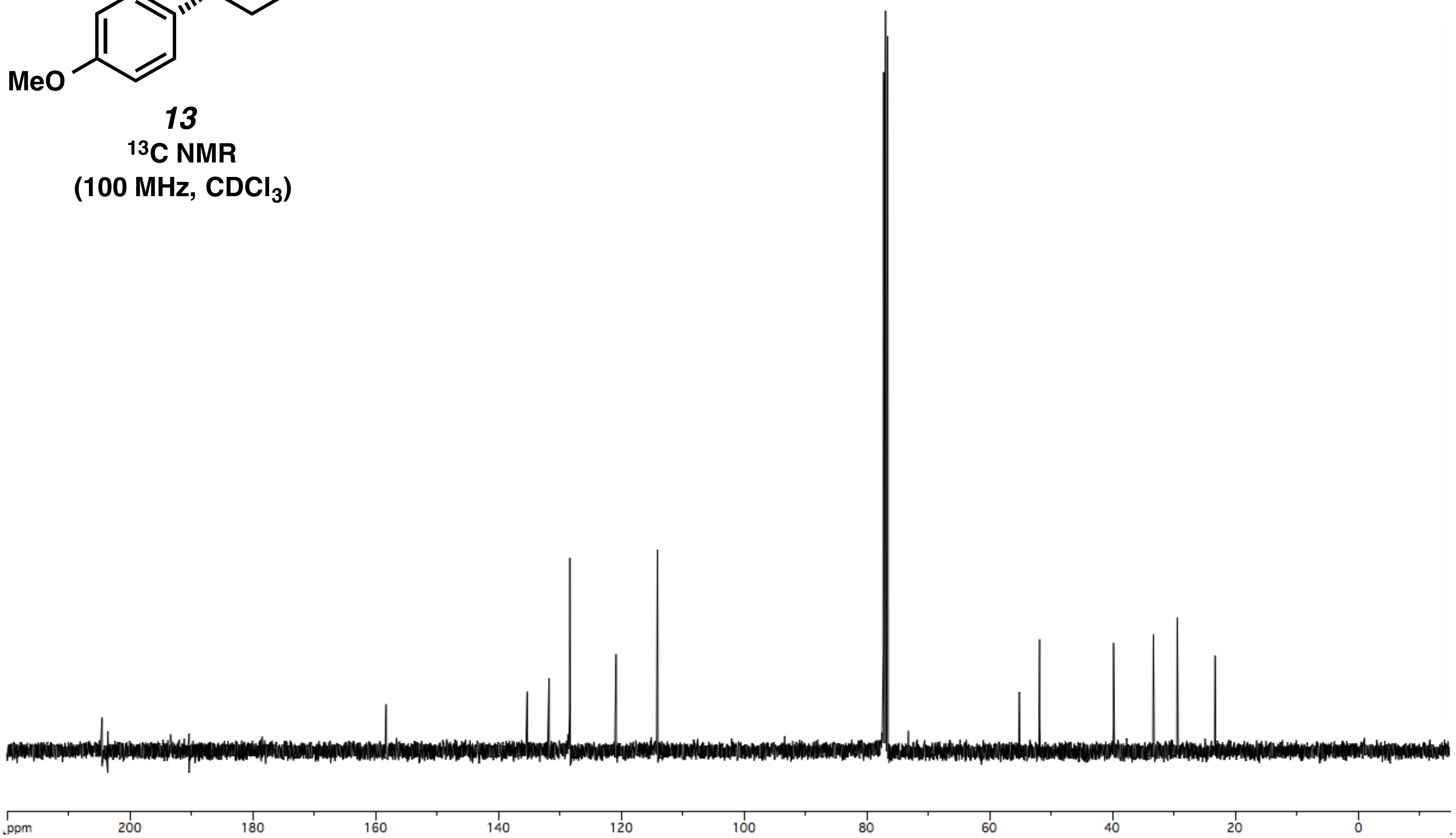

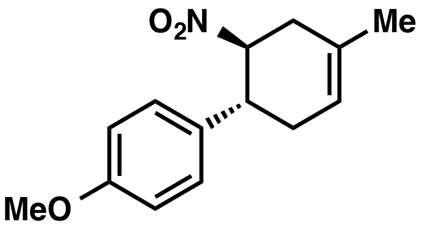

14
^{1}H NMR
(400 MHz, $CDCl_3$)

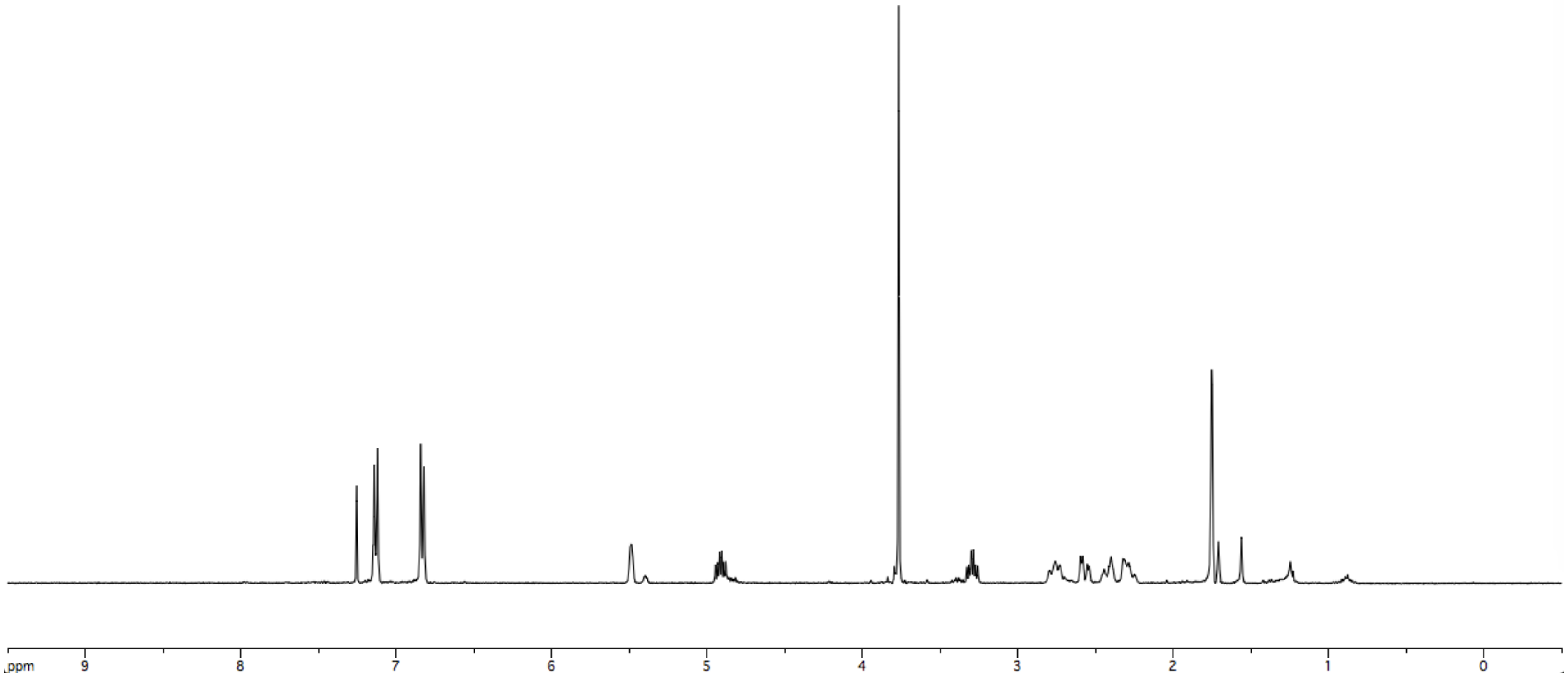

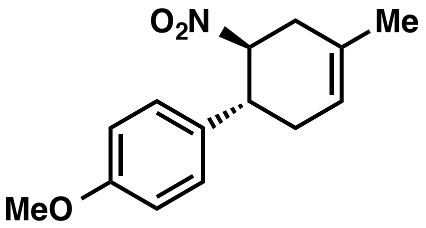

14

^{13}C NMR

(100 MHz, $CDCl_3$)

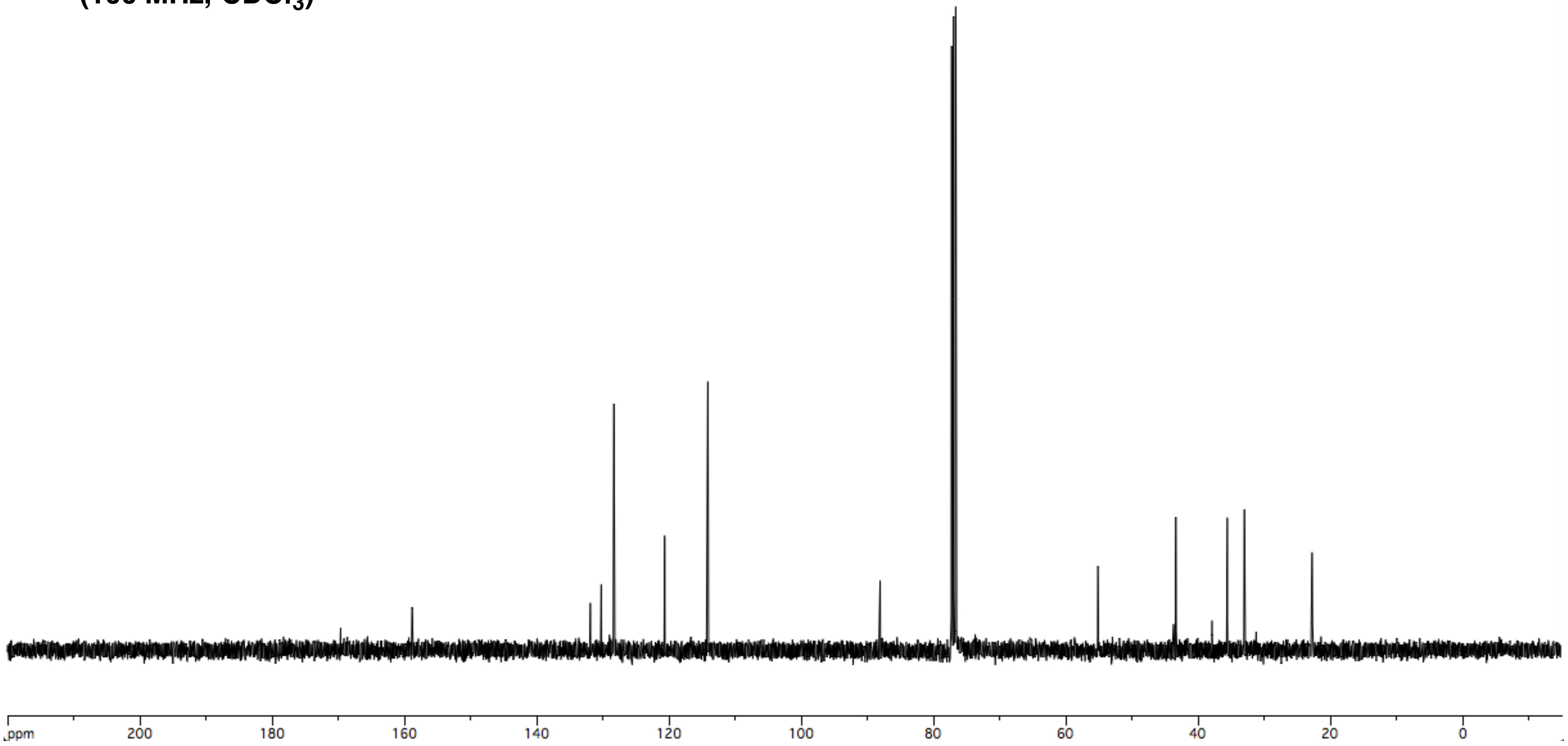

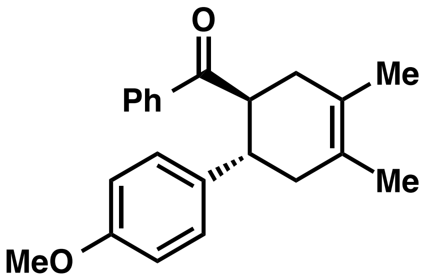

15

^{1}H NMR

(400 MHz, $CDCl_3$)

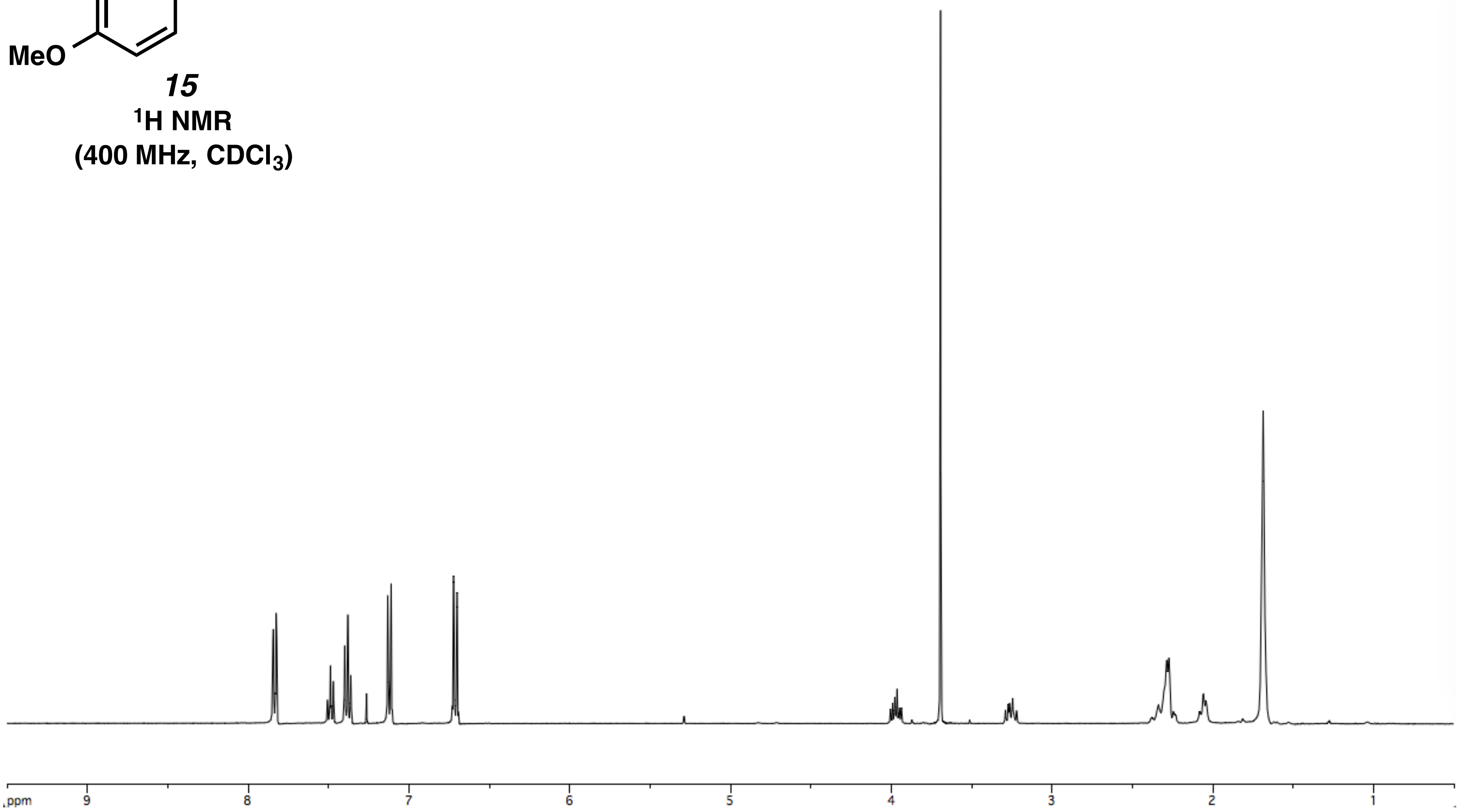

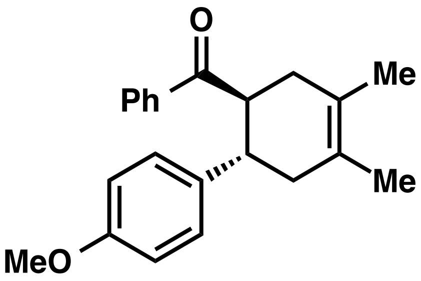

15
^{13}C NMR
(100 MHz, $CDCl_3$)

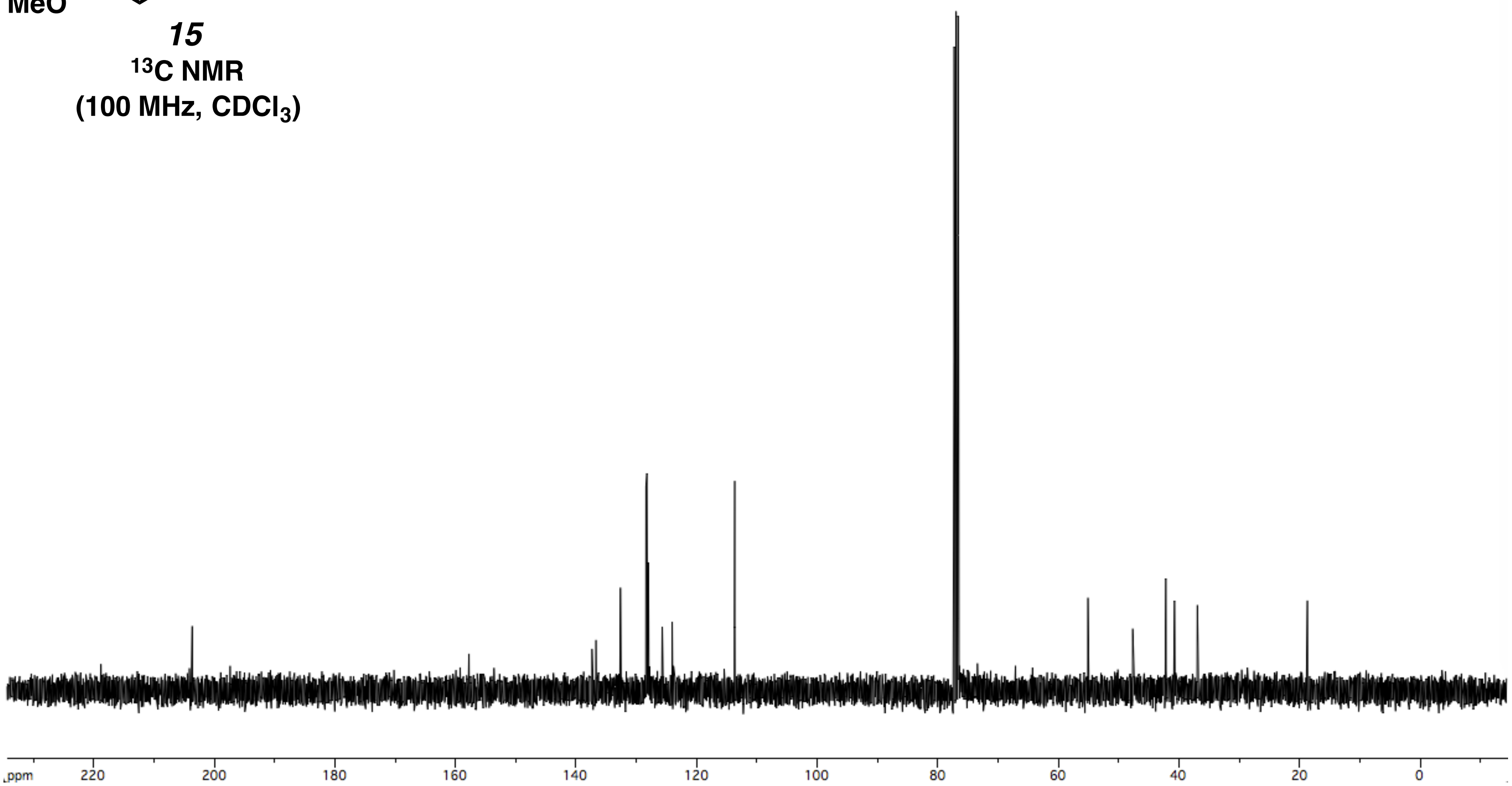

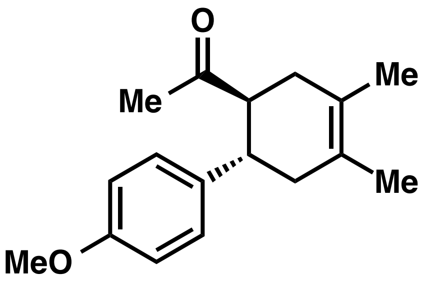

16

^{1}H NMR

(400 MHz, $CDCl_3$)

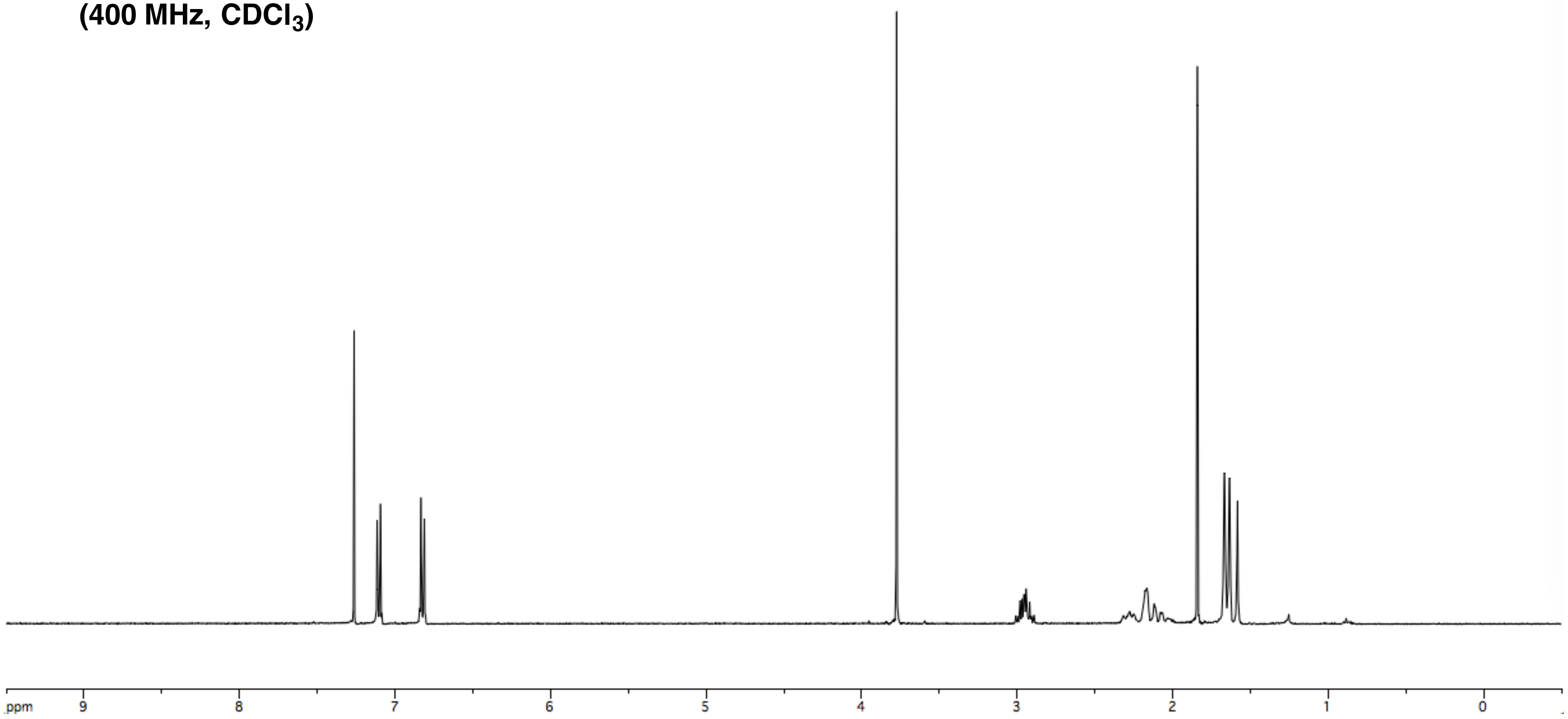

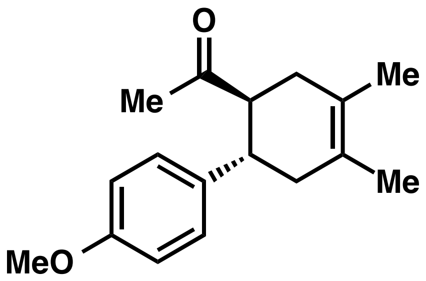

16

^{13}C NMR

(100 MHz, $CDCl_3$)

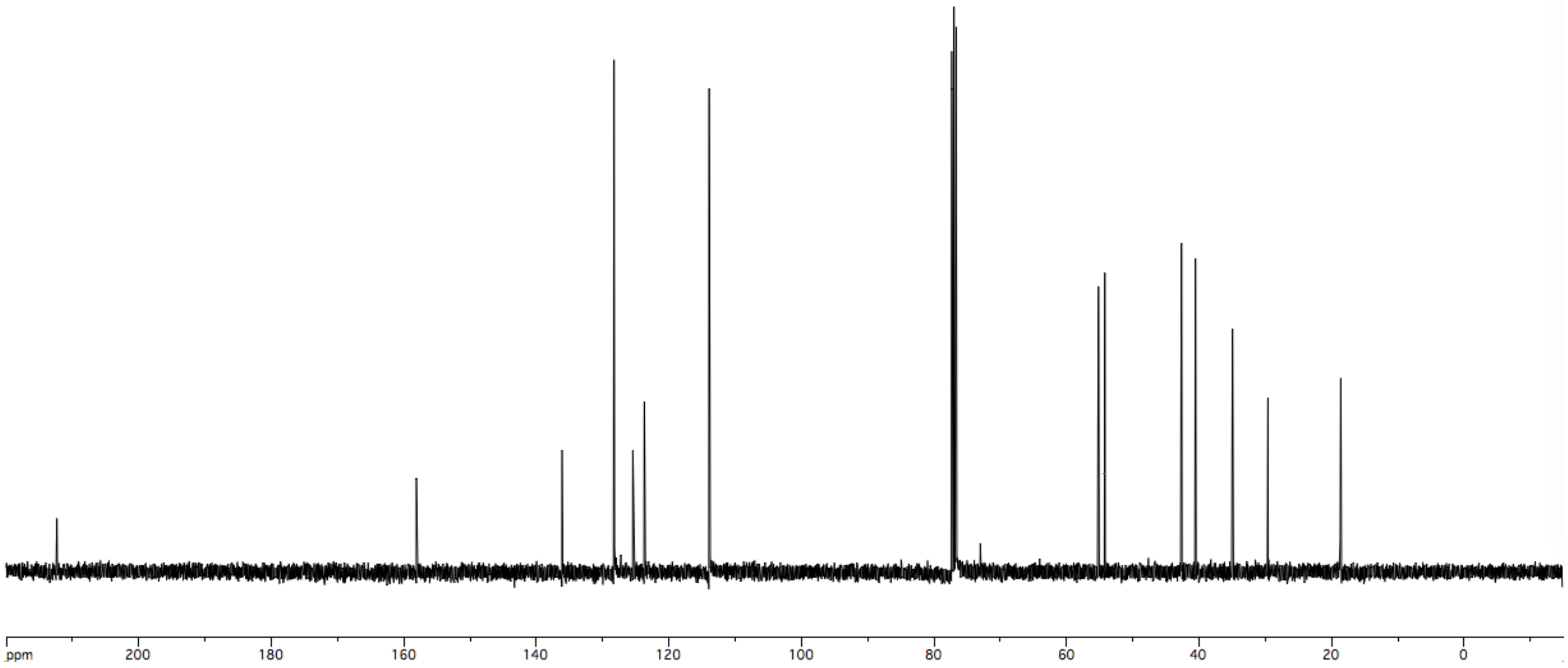

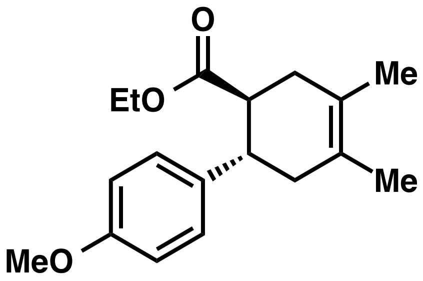

17
^{1}H NMR
(400 MHz, $CDCl_3$)

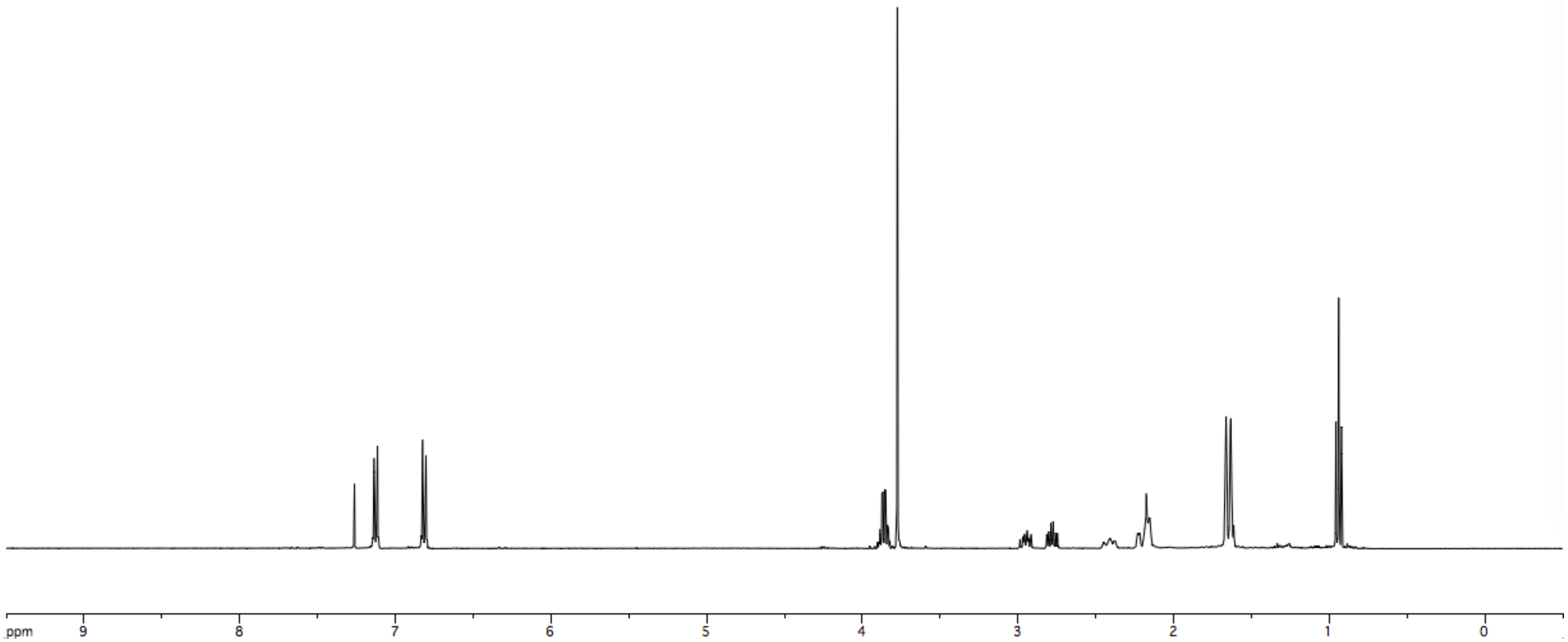

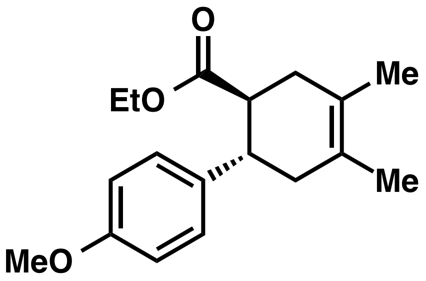

17

^{13}C NMR

(100 MHz, $CDCl_3$)

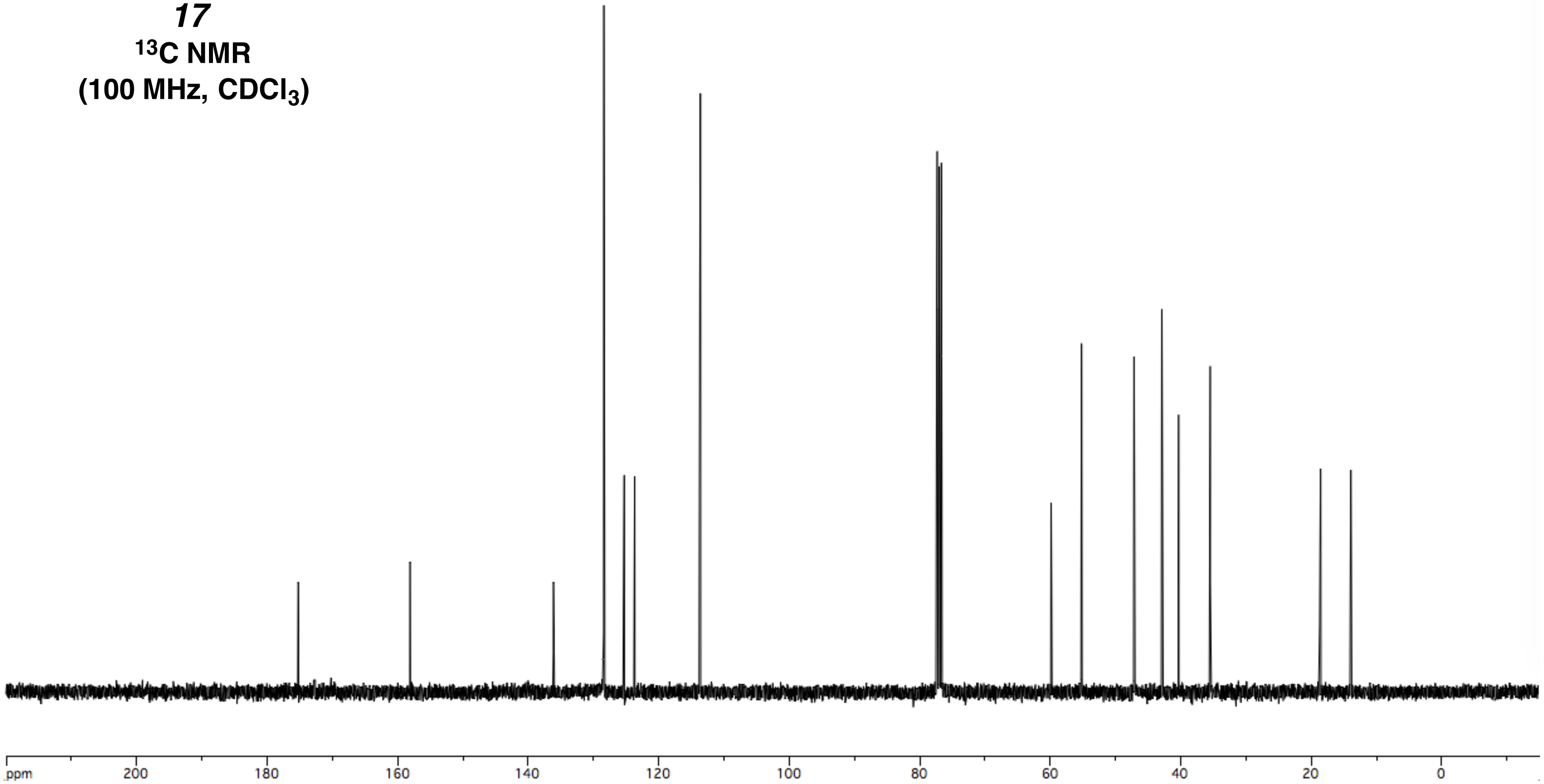

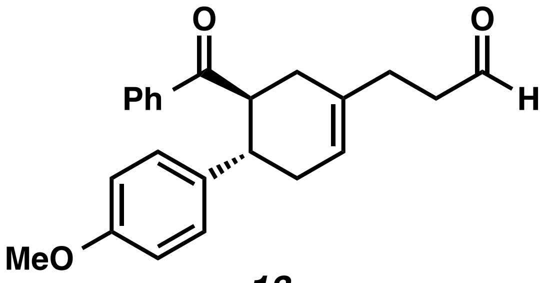

18
^{1}H NMR
(400 MHz, $CDCl_3$)

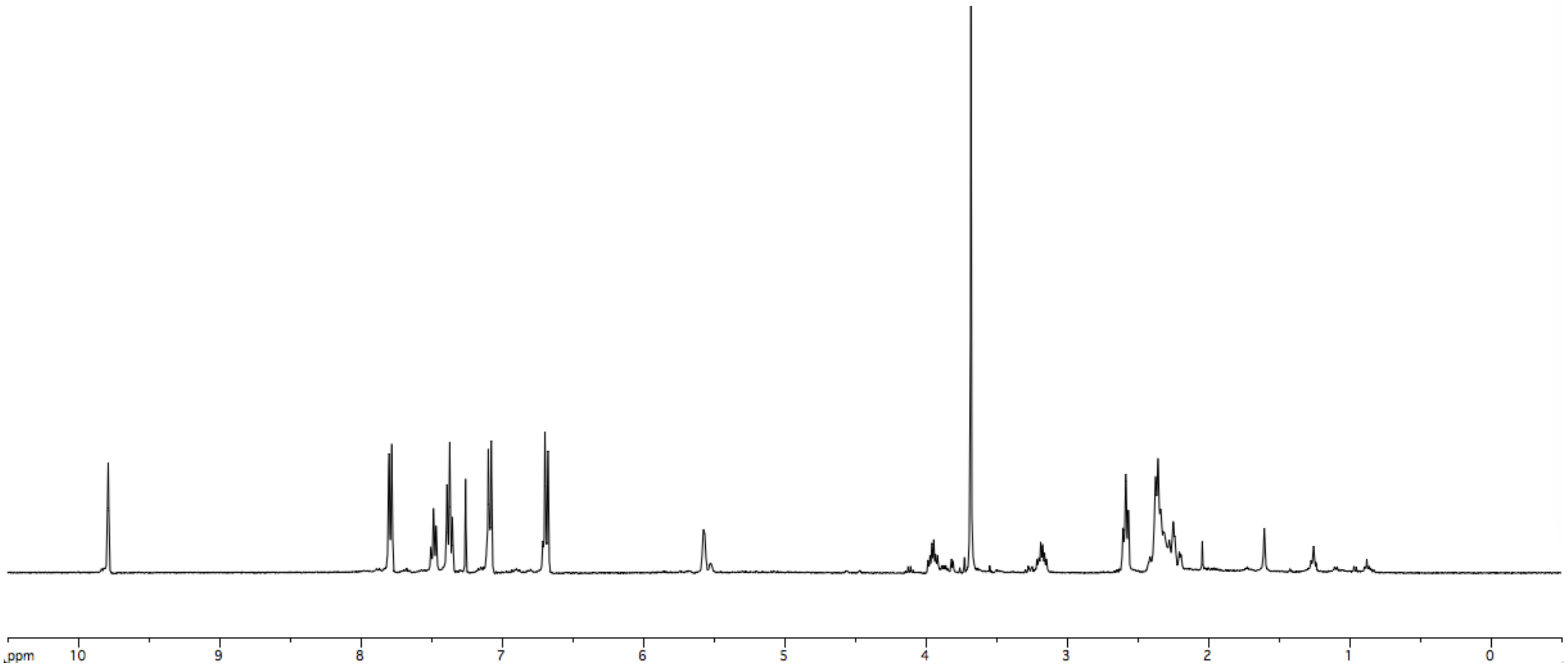

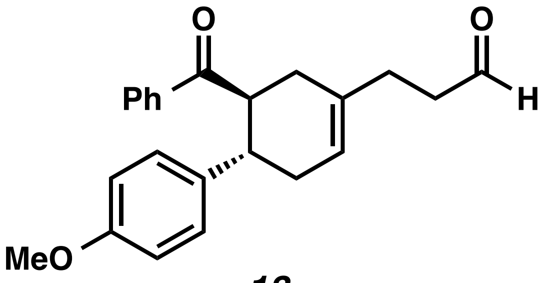

18

^{13}C NMR

(100 MHz, $CDCl_3$)

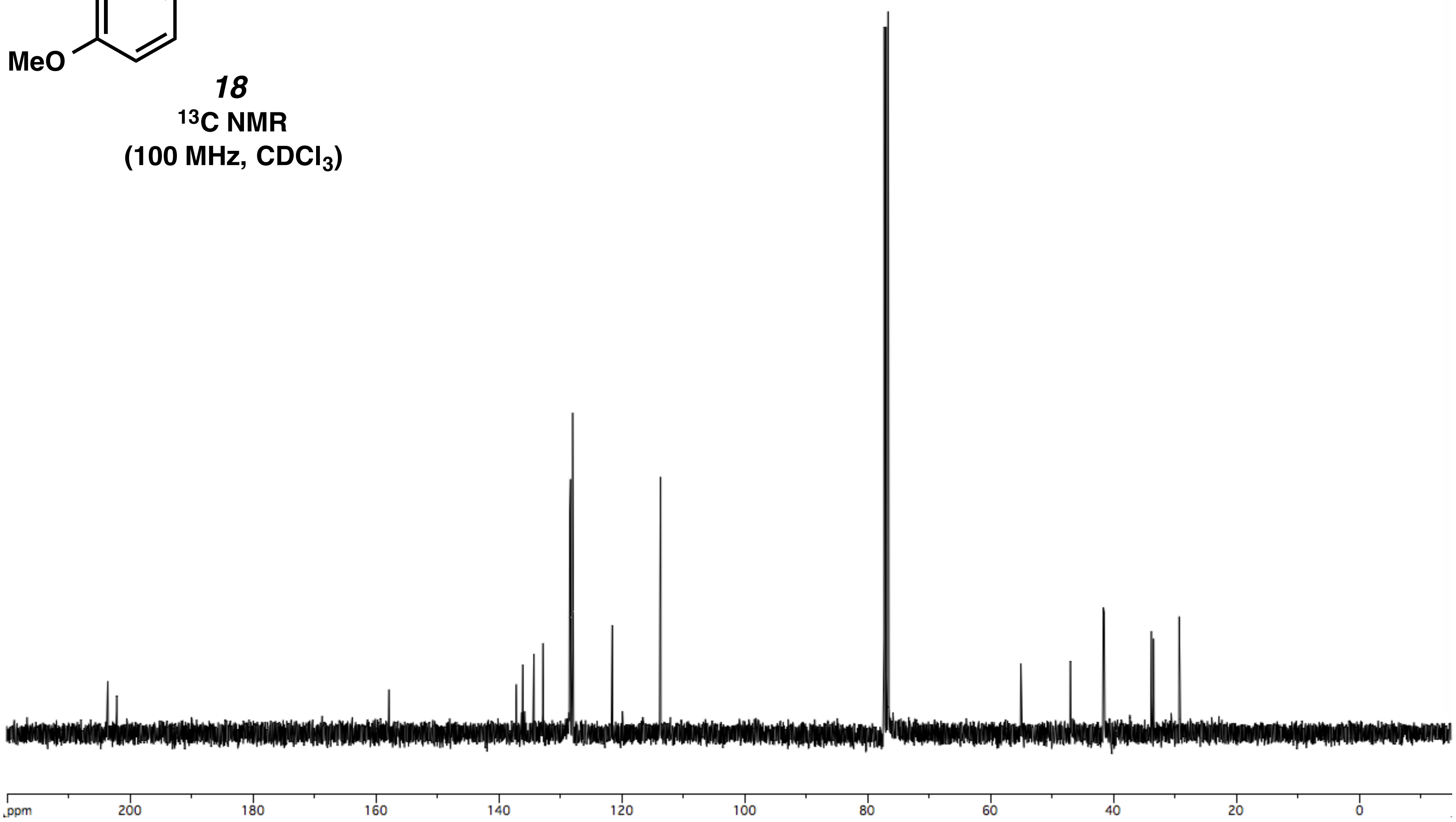

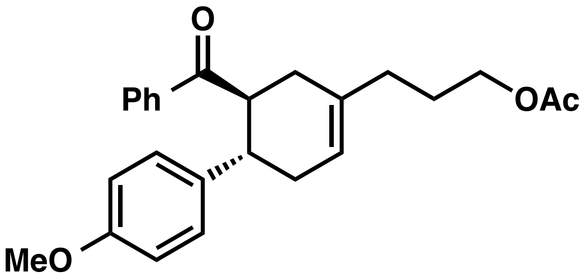

19
^{1}H NMR
(400 MHz, $CDCl_3$)

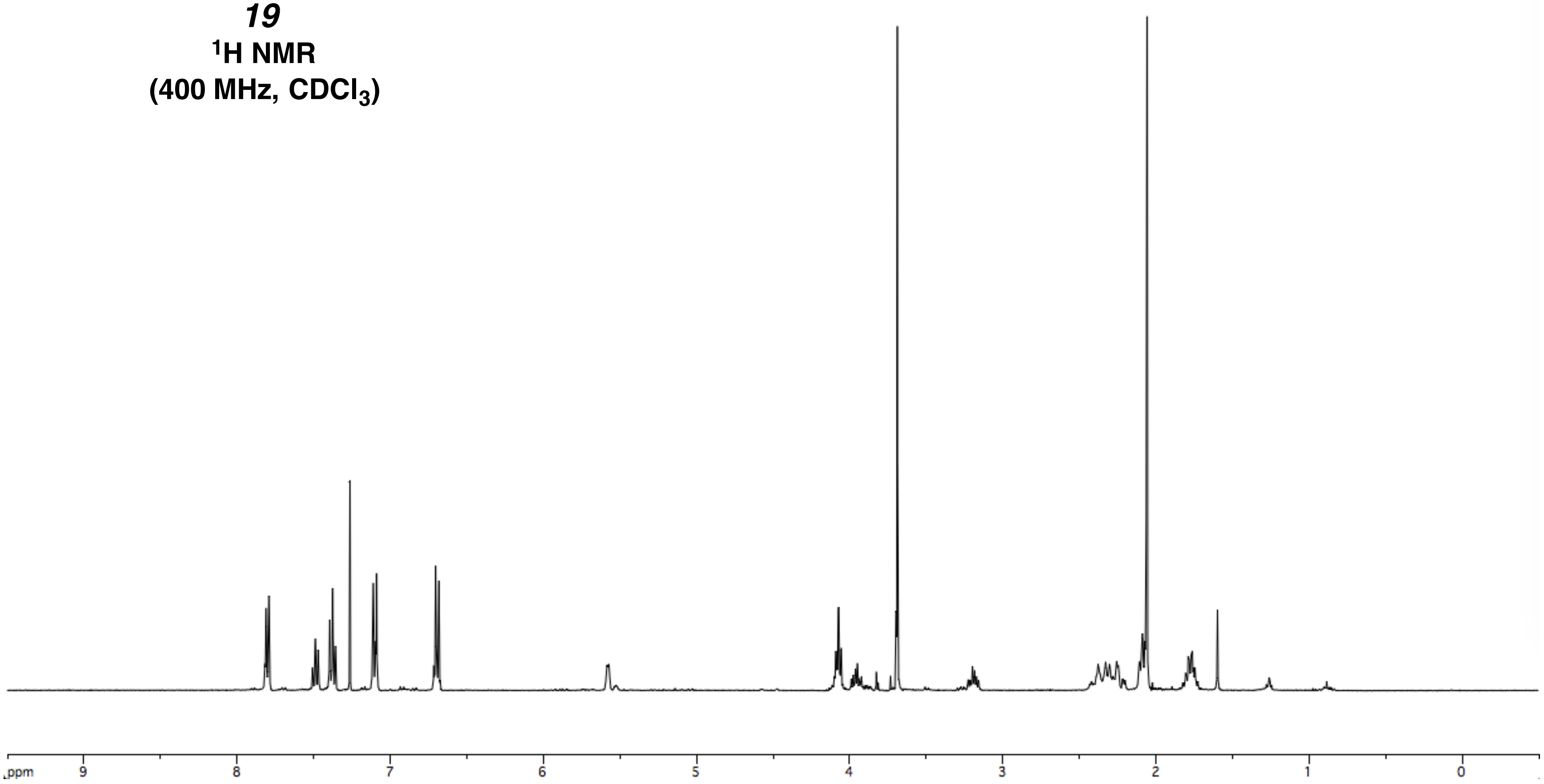

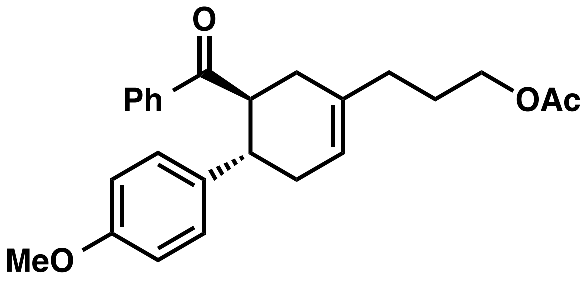

19

^{13}C NMR

(100 MHz, $CDCl_3$)

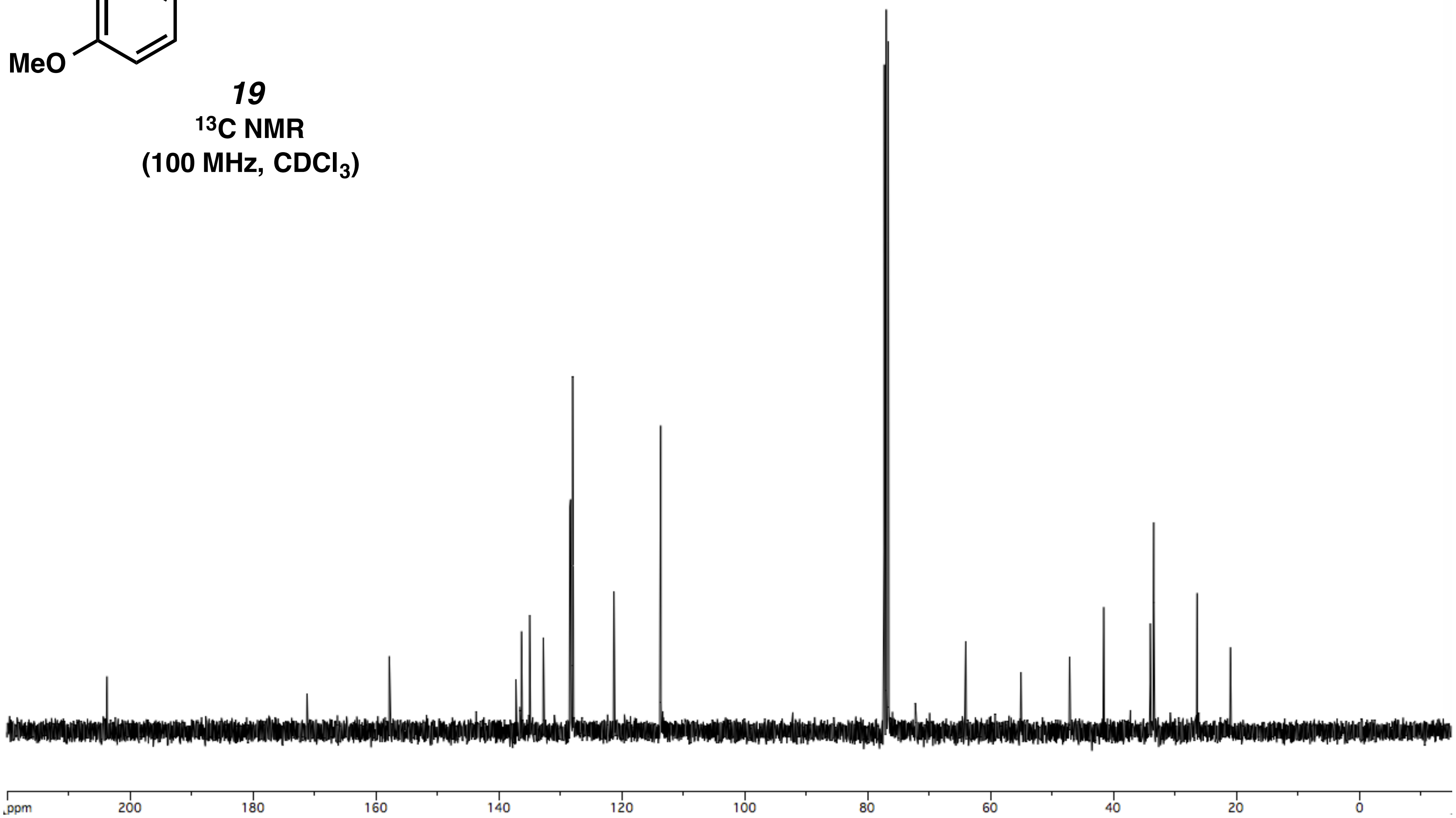

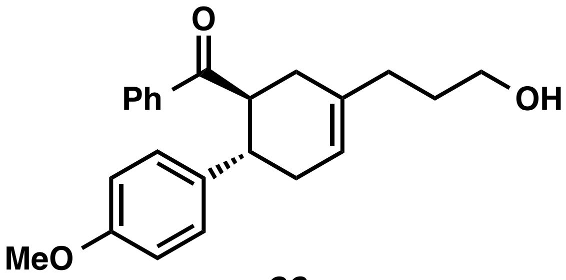

20
^{1}H NMR
(400 MHz, $CDCl_3$)

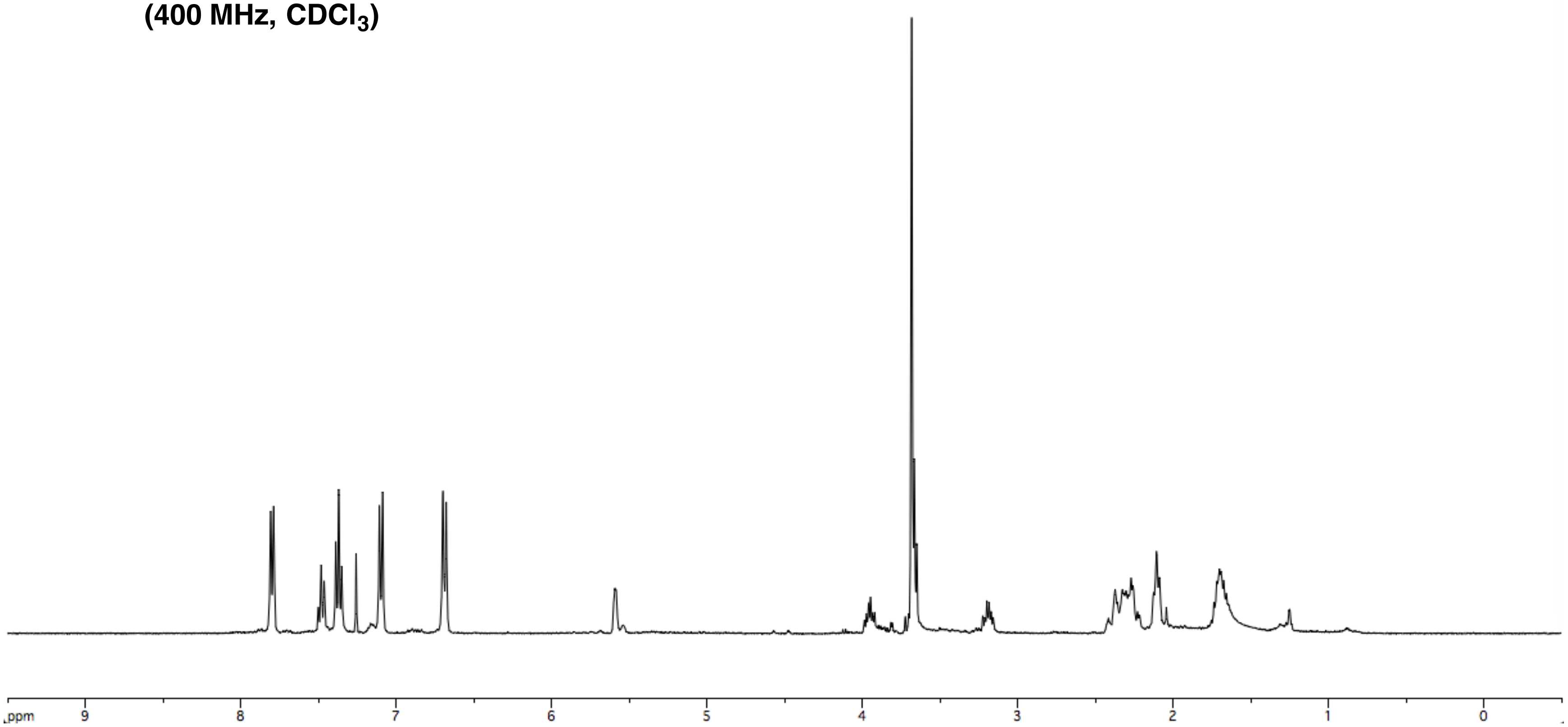

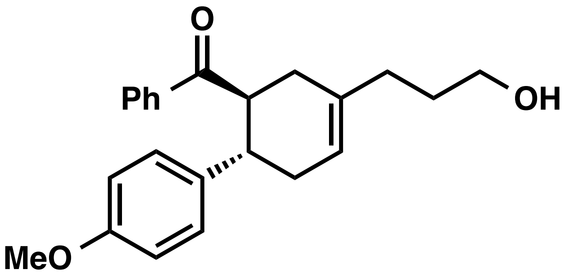

20

^{13}C NMR

(100 MHz, $CDCl_3$)

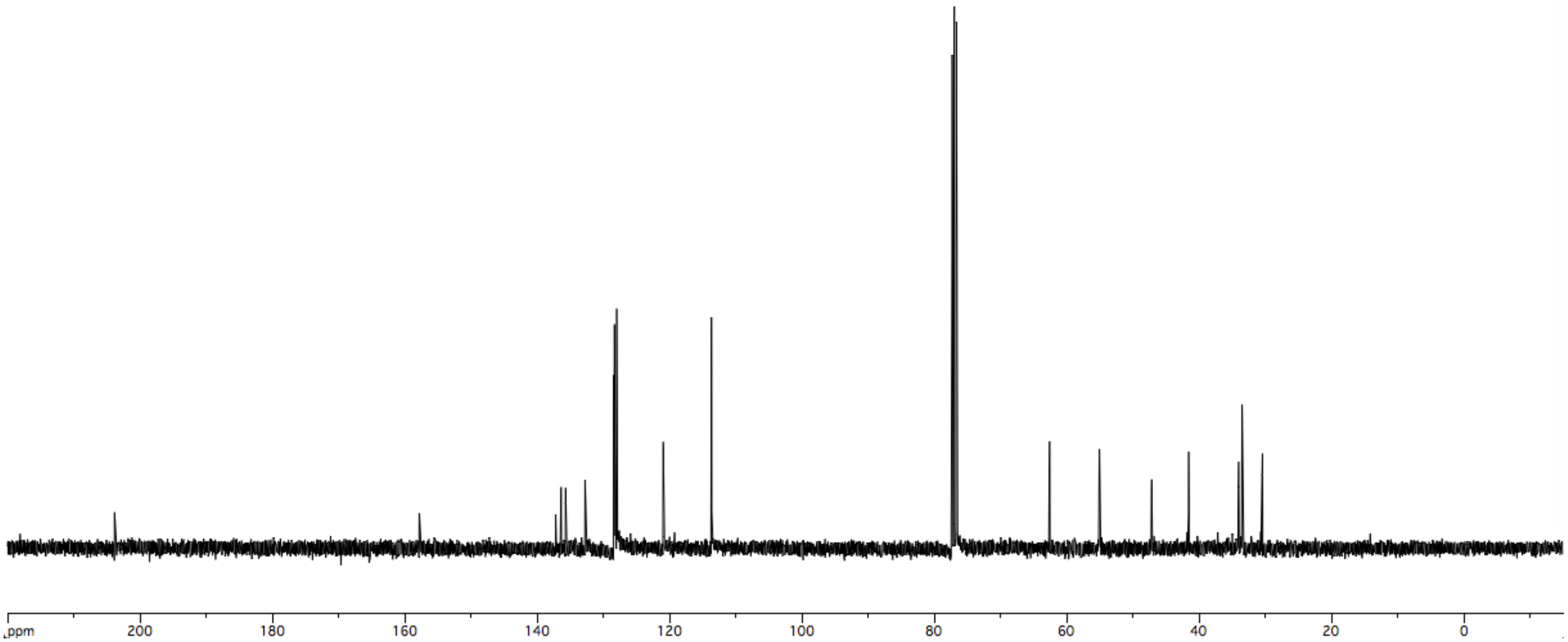

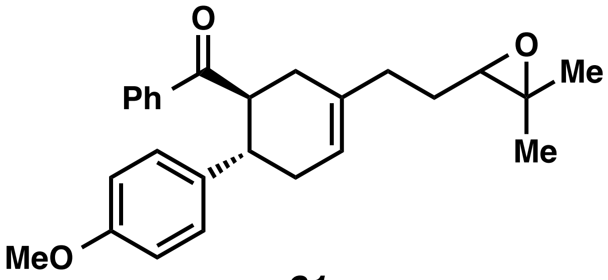

21

^{1}H NMR

(400 MHz, $CDCl_3$)

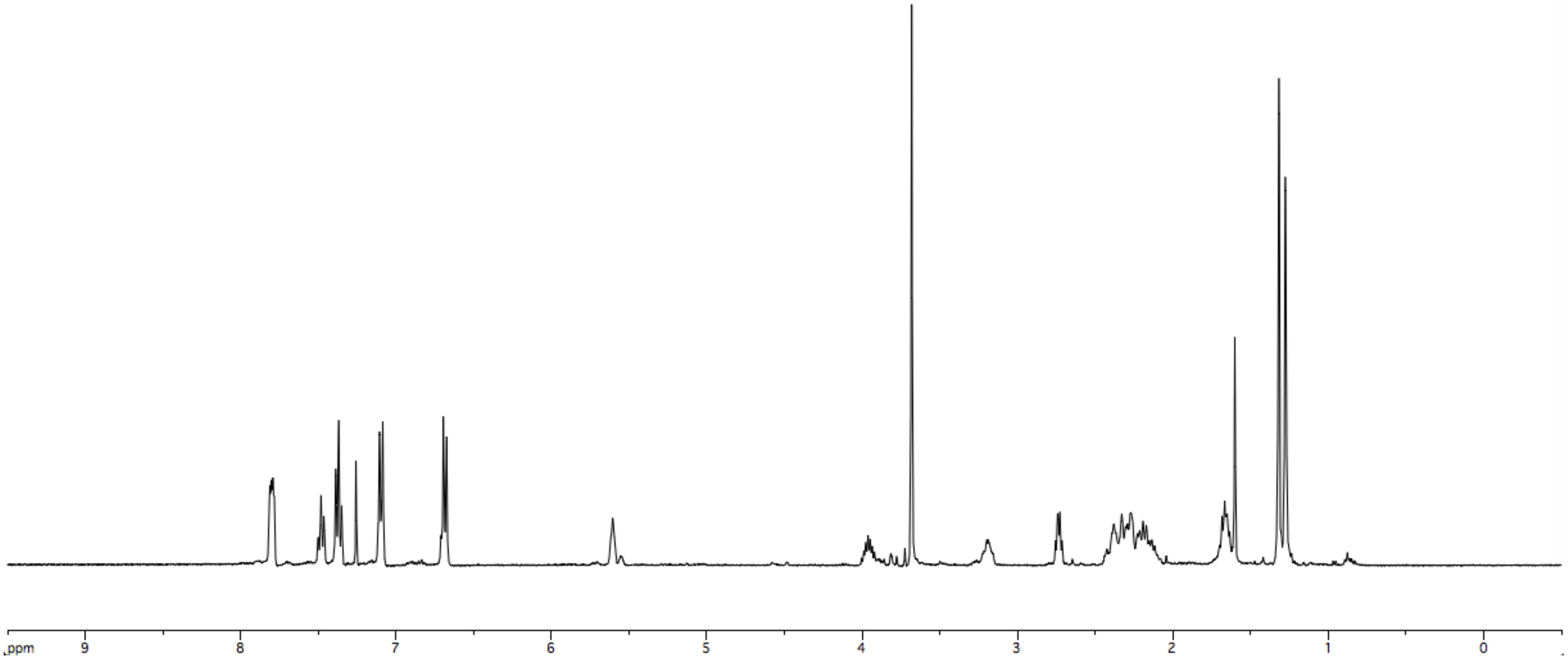

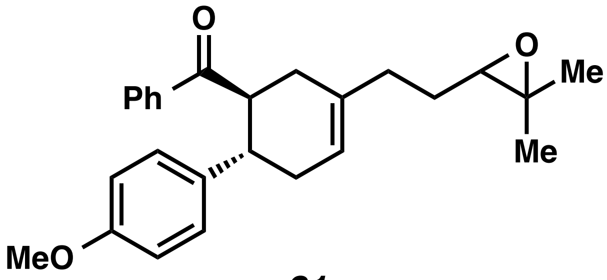

21

^{13}C NMR

(100 MHz, $CDCl_3$)

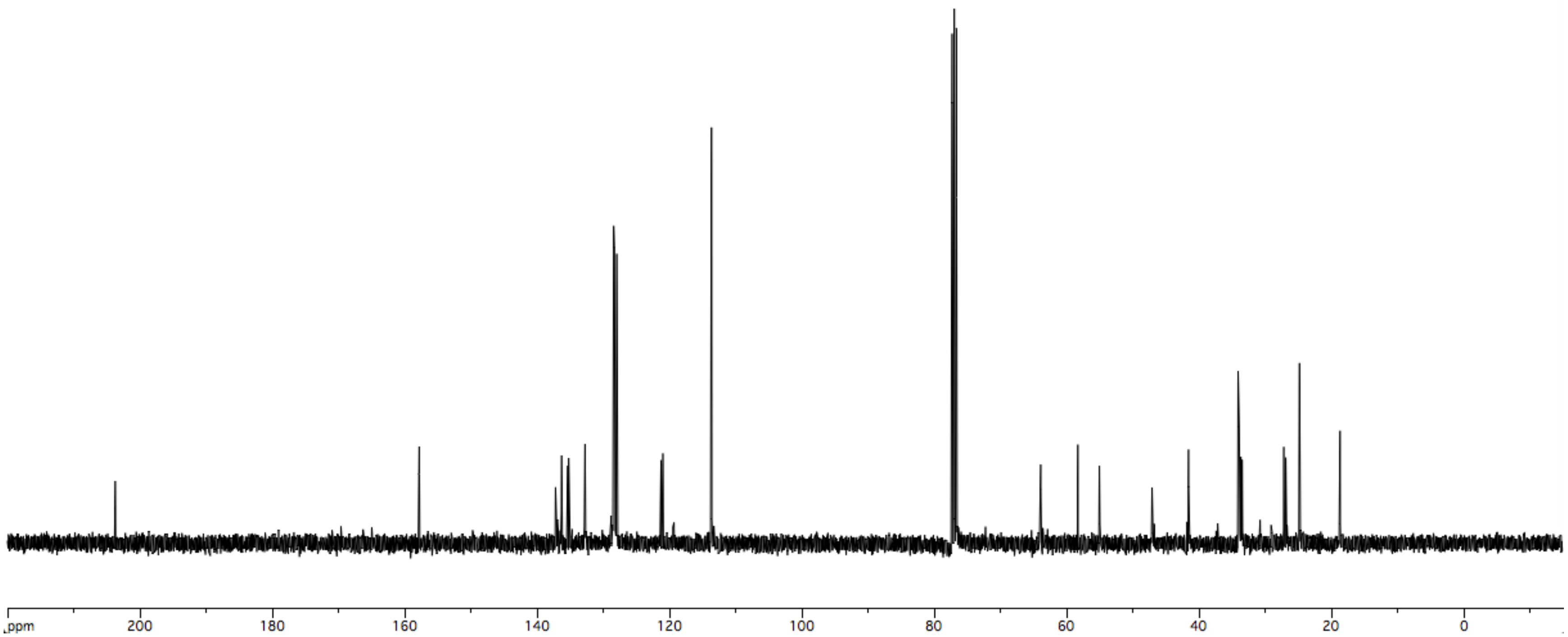

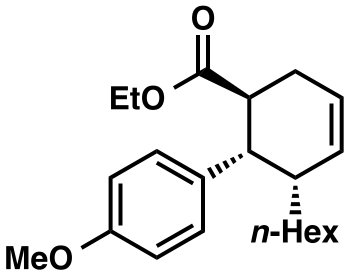

22
^{1}H NMR
(400 MHz, $CDCl_3$)

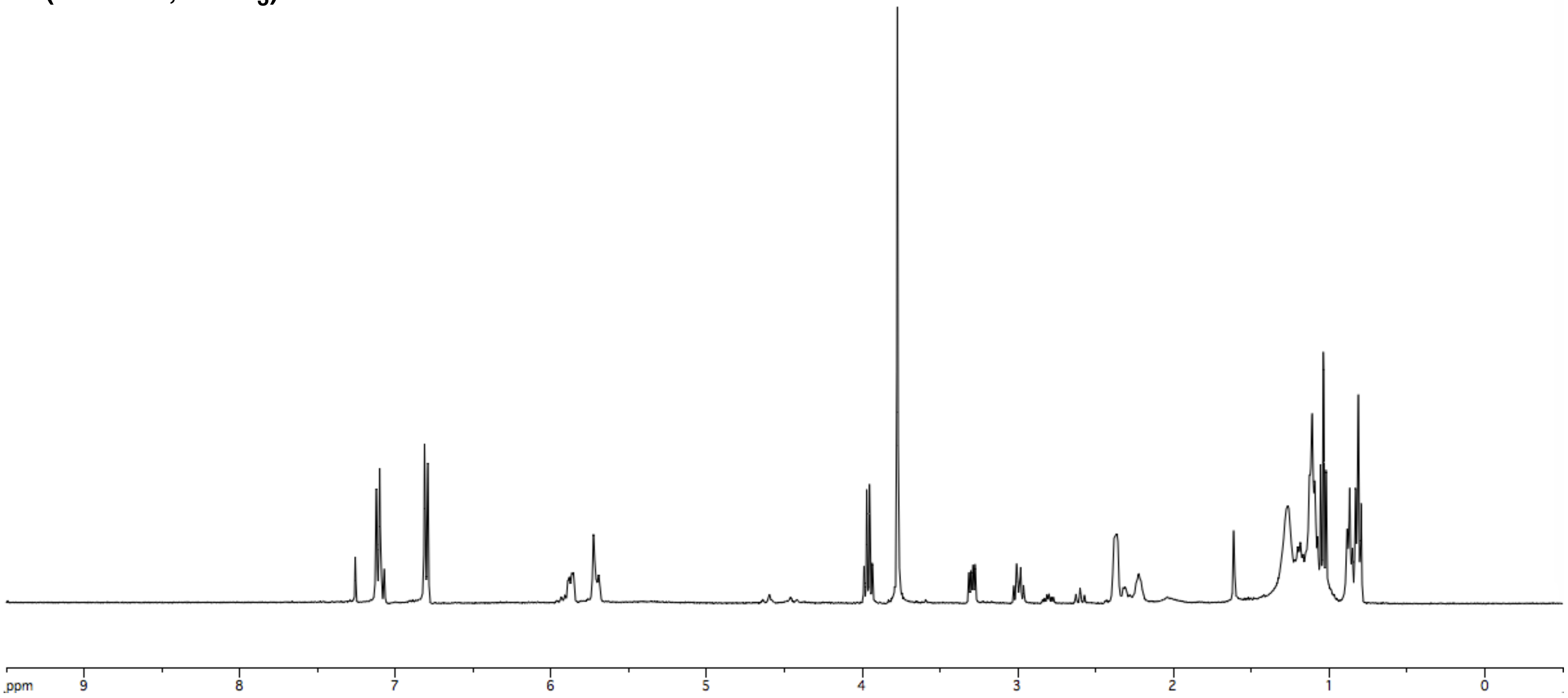

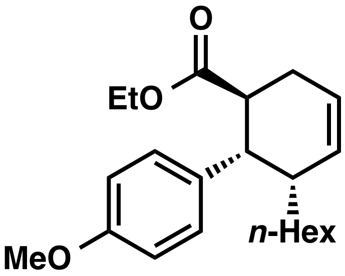

22

^{13}C NMR
(100 MHz, $CDCl_3$)

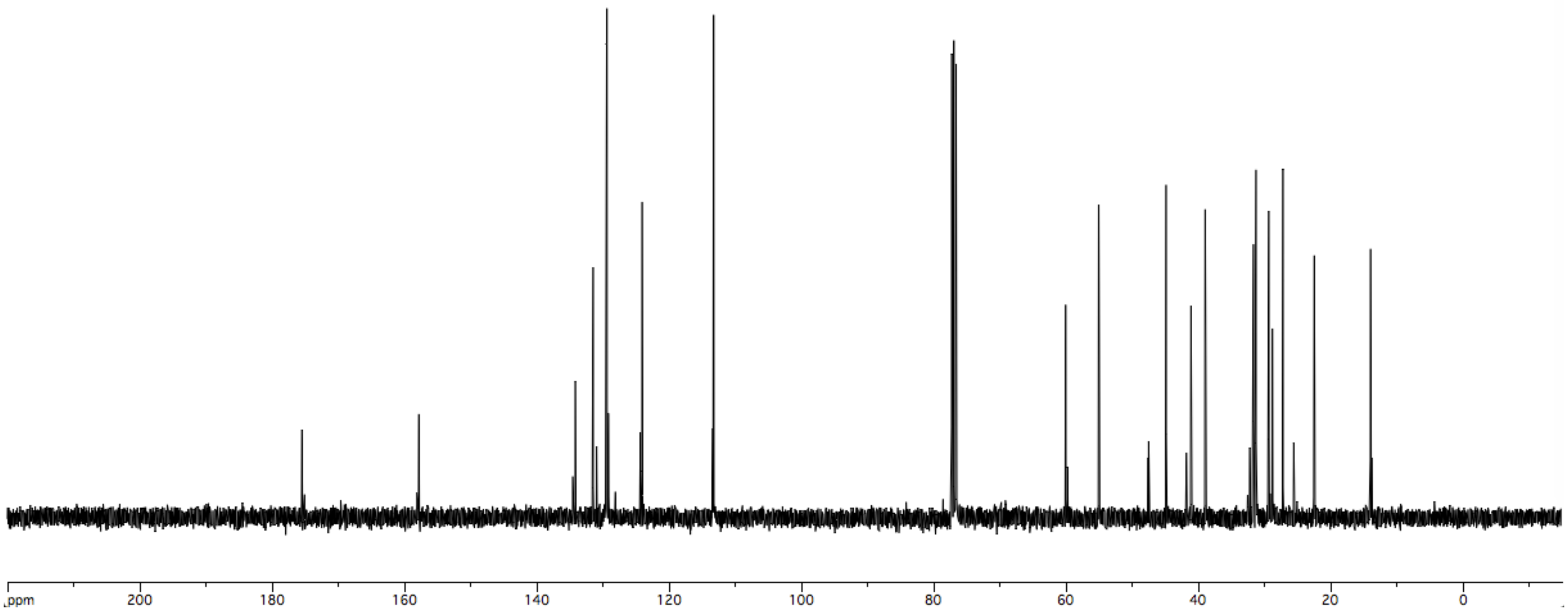

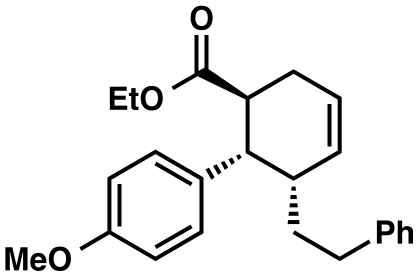

23
^{1}H NMR
(400 MHz, $CDCl_3$)

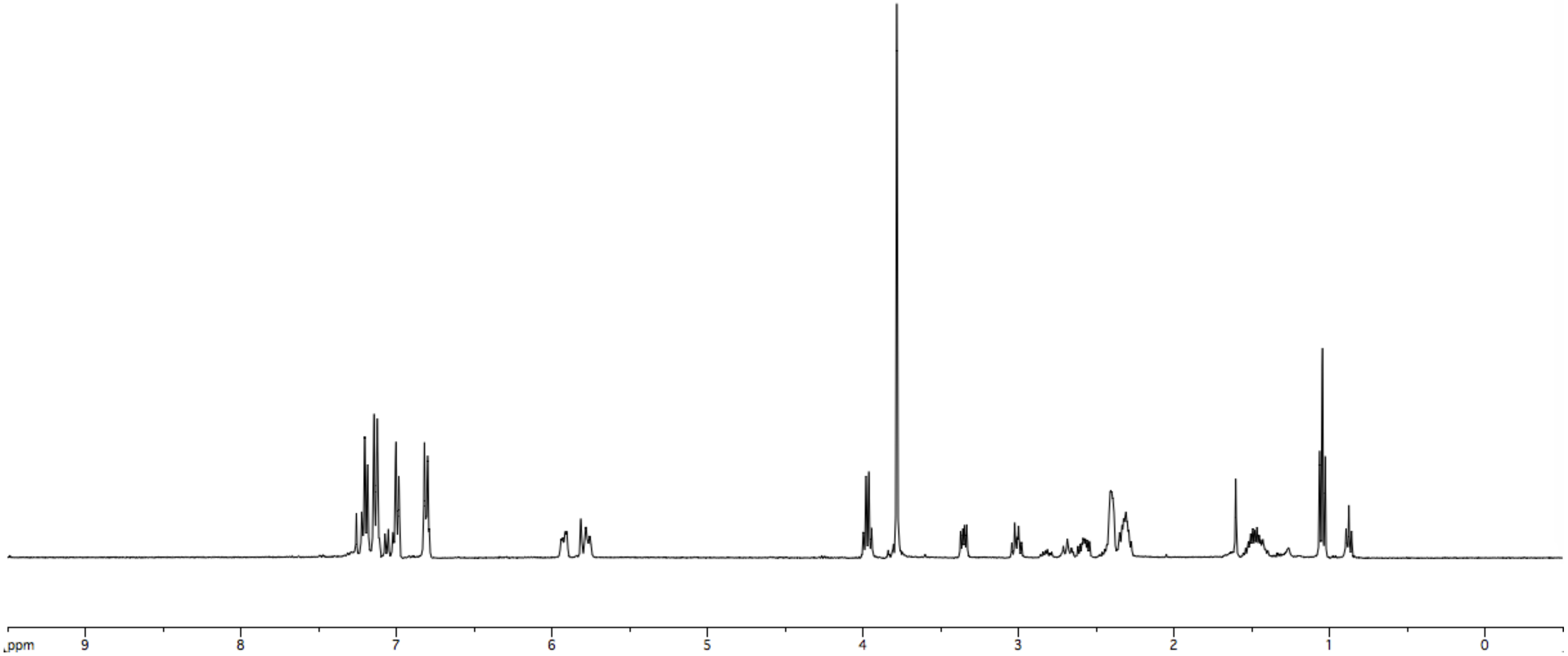

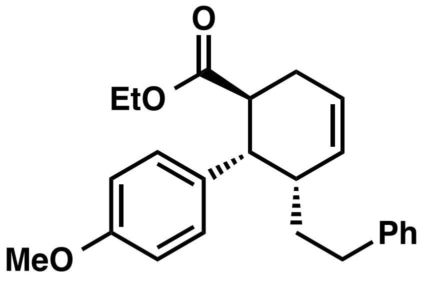

23
^{13}C NMR
(100 MHz, $CDCl_3$)

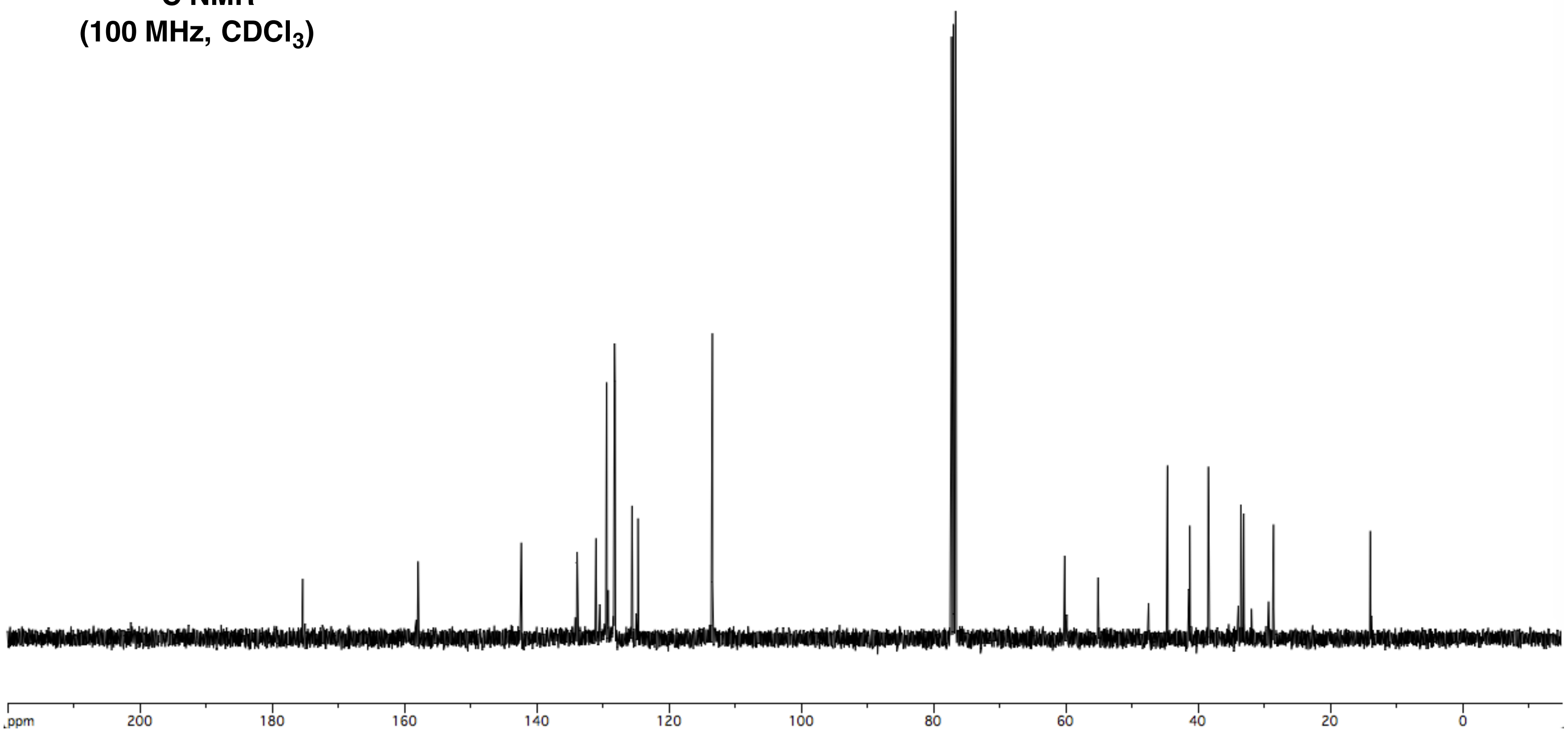

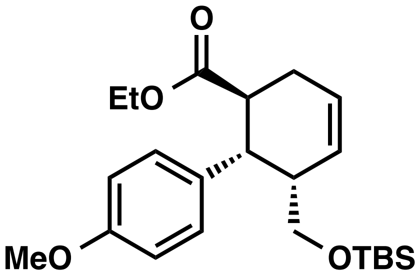

24
^{1}H NMR
(400 MHz, $CDCl_3$)

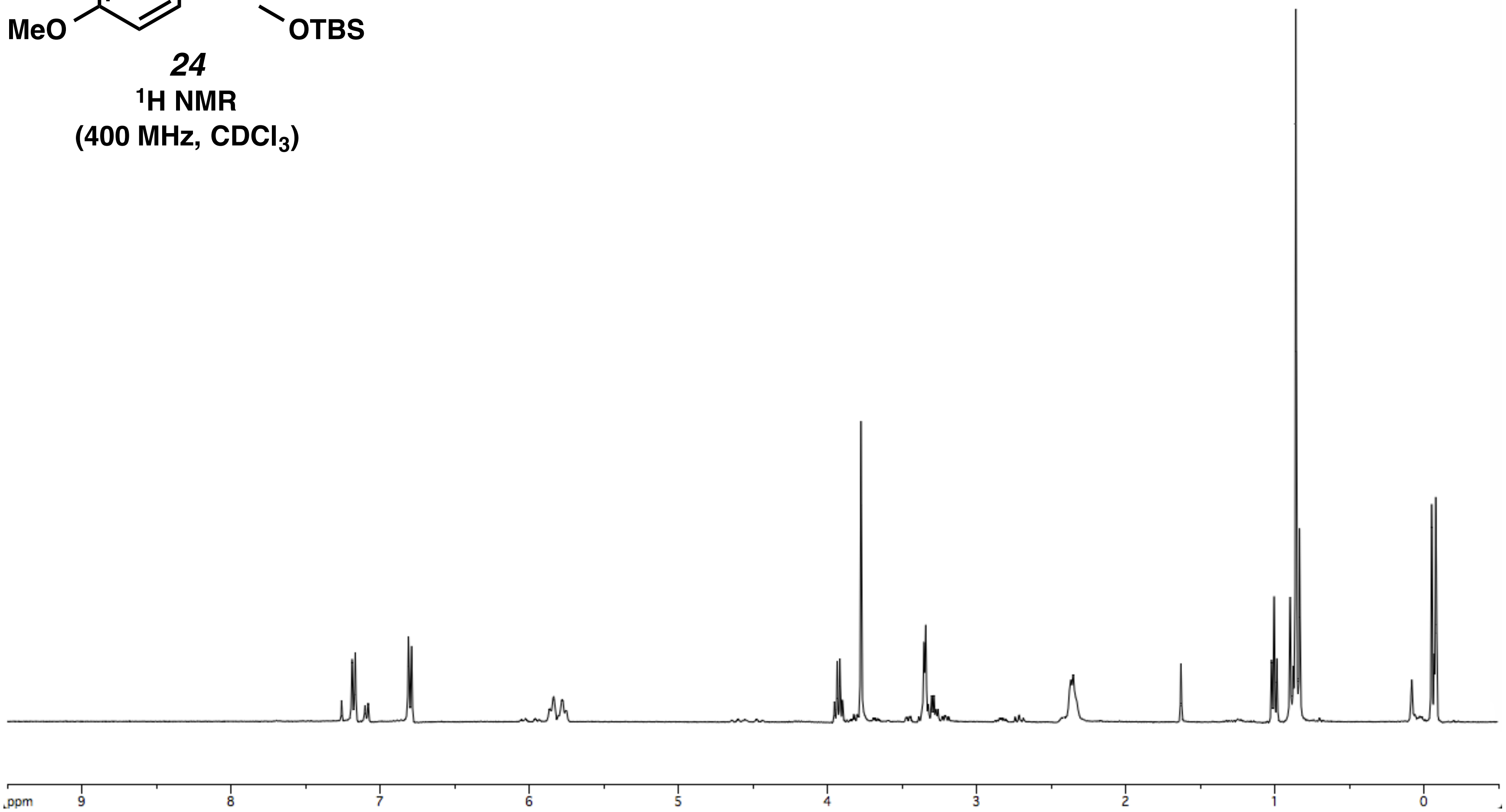

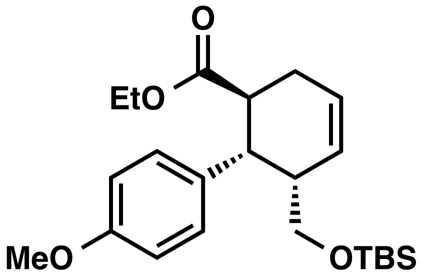

24

^{13}C NMR
(100 MHz, $CDCl_3$)

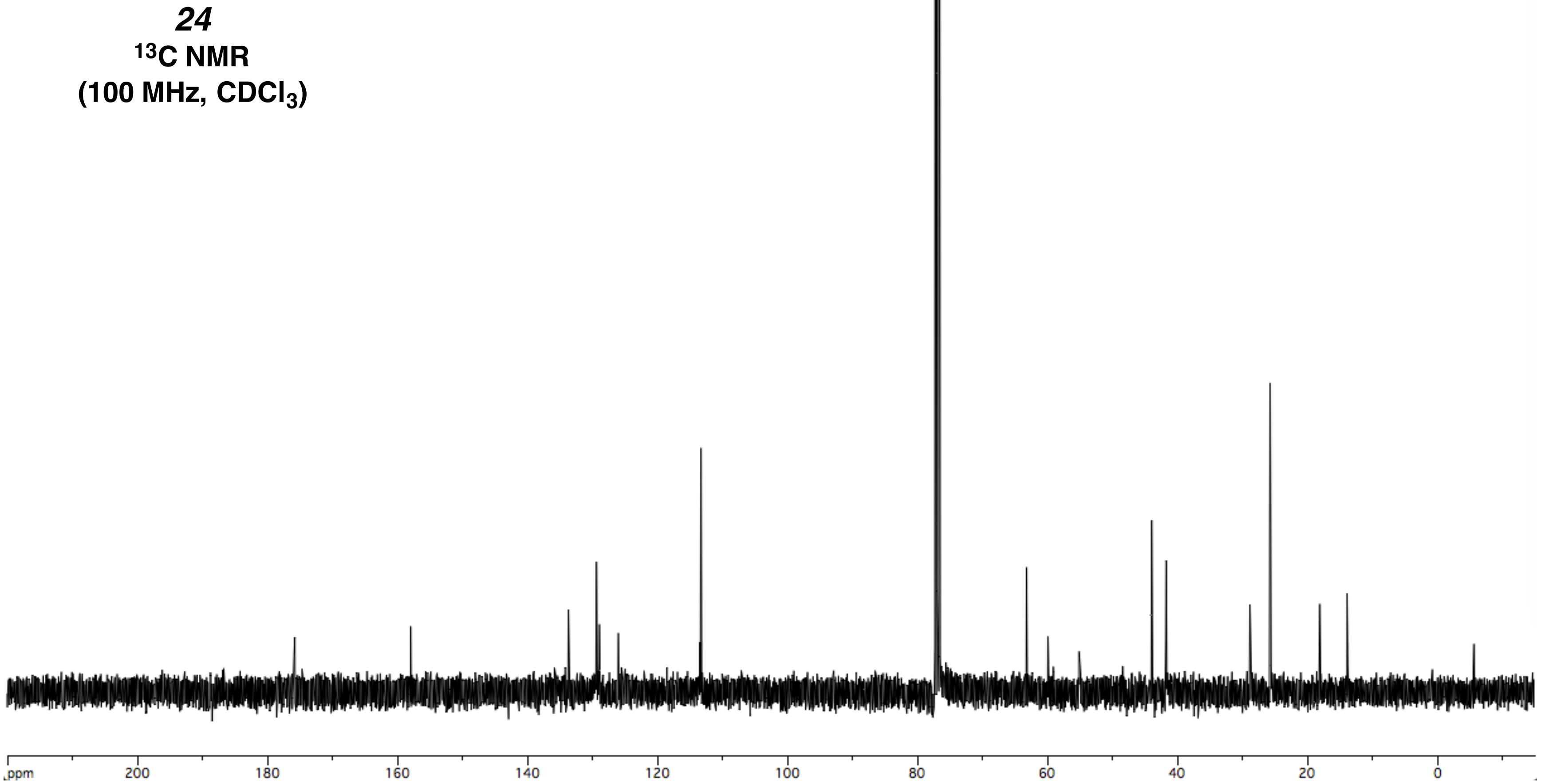

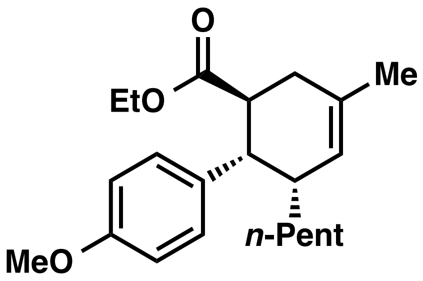

25
^{1}H NMR
(400 MHz, $CDCl_3$)

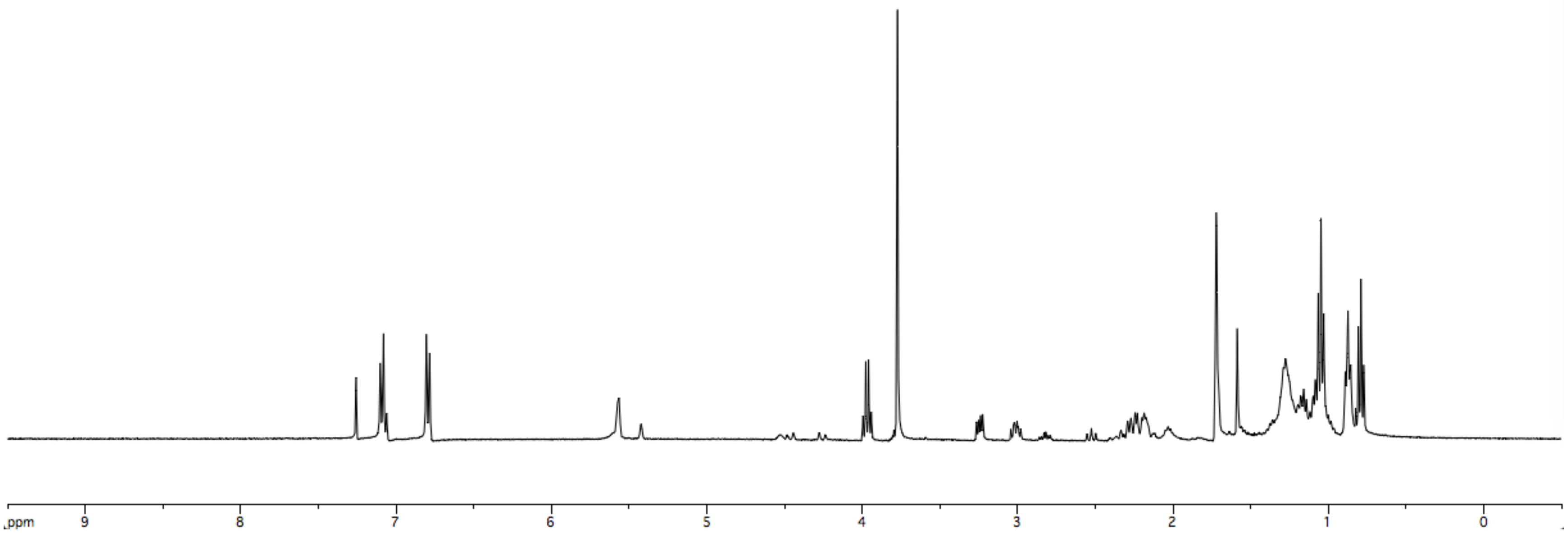

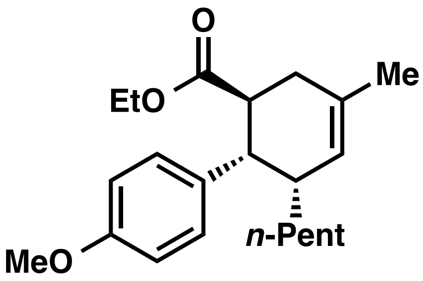

25

^{13}C NMR

(100 MHz, $CDCl_3$)

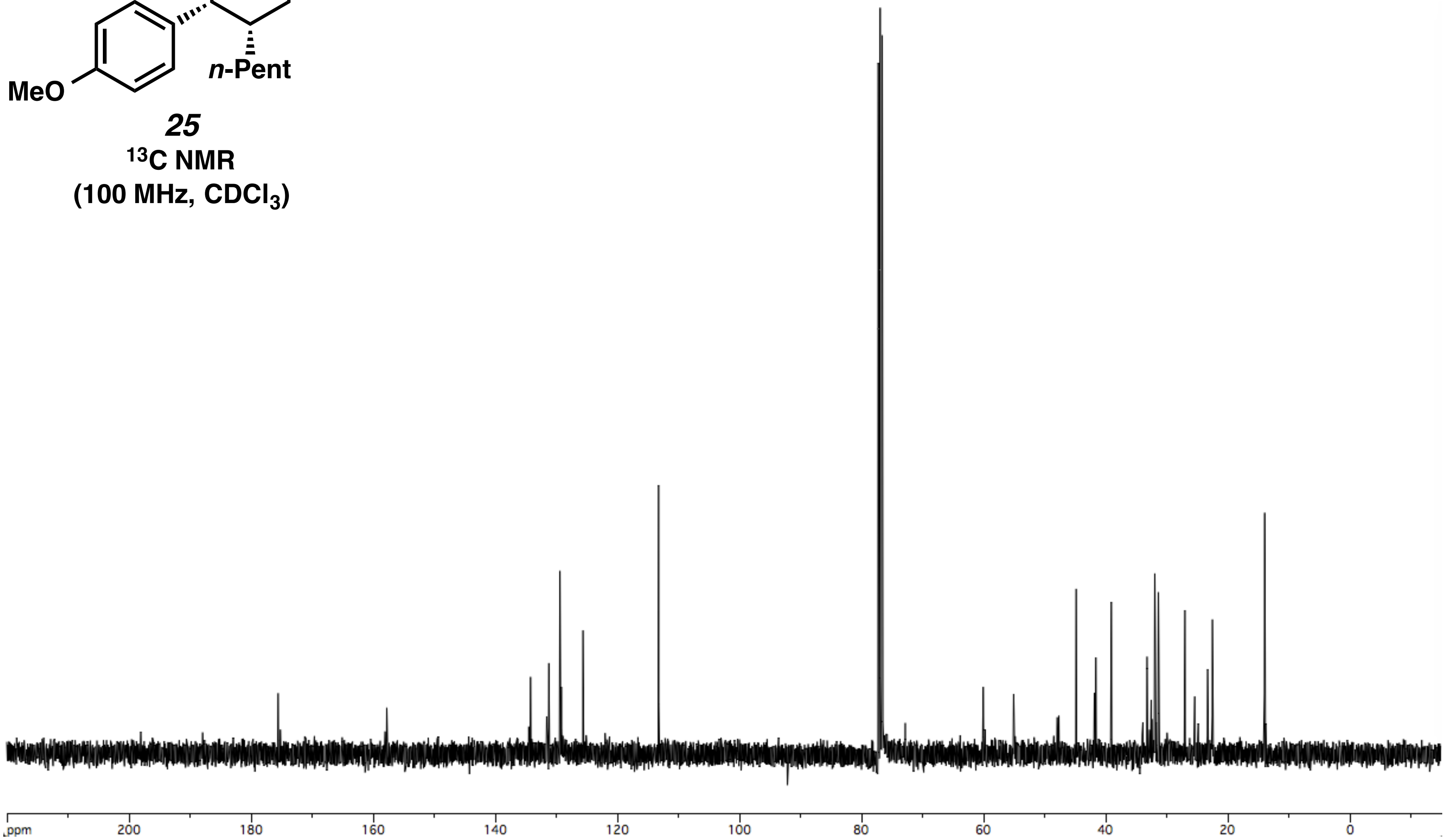

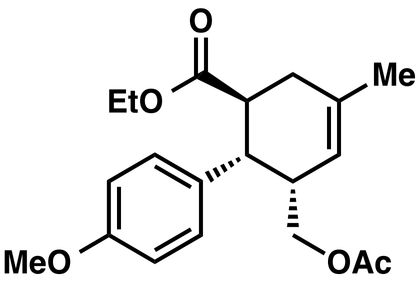

26

^{1}H NMR

(400 MHz, $CDCl_3$)

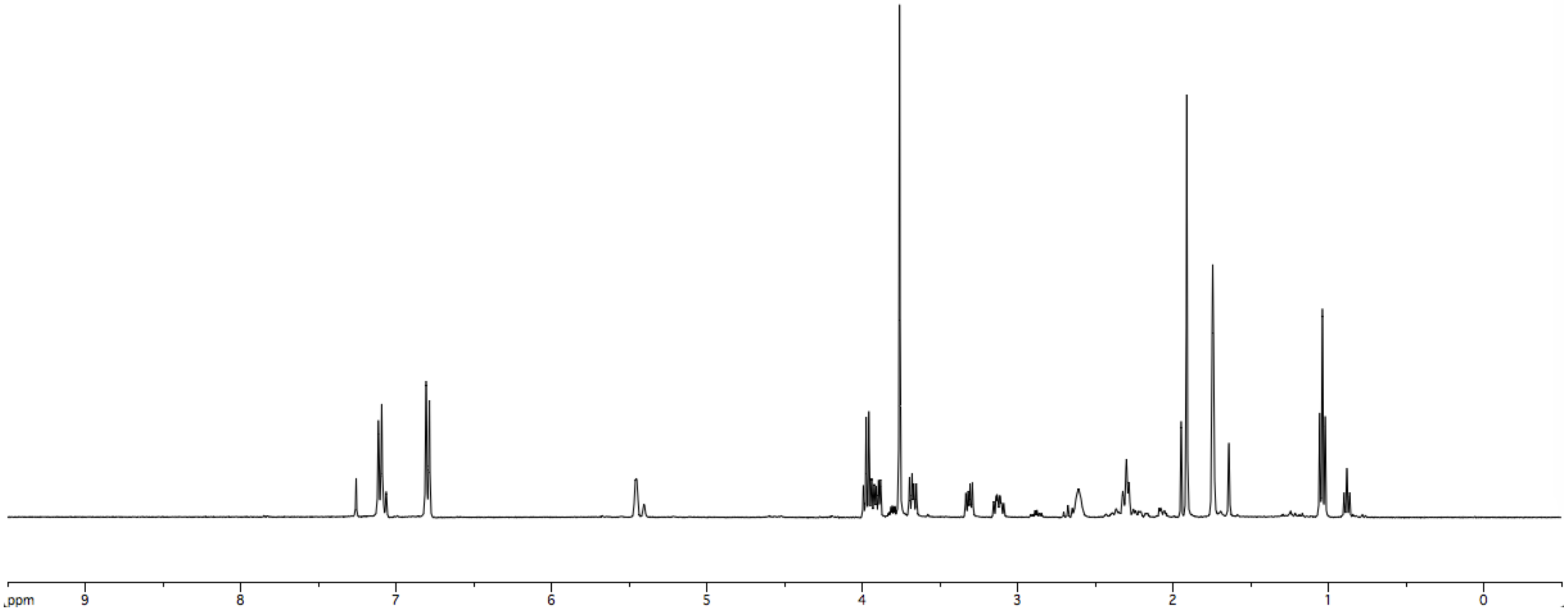

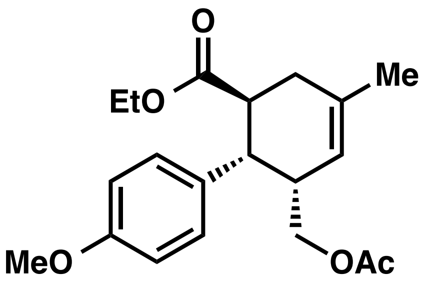

26
^{13}C NMR
(100 MHz, $CDCl_3$)

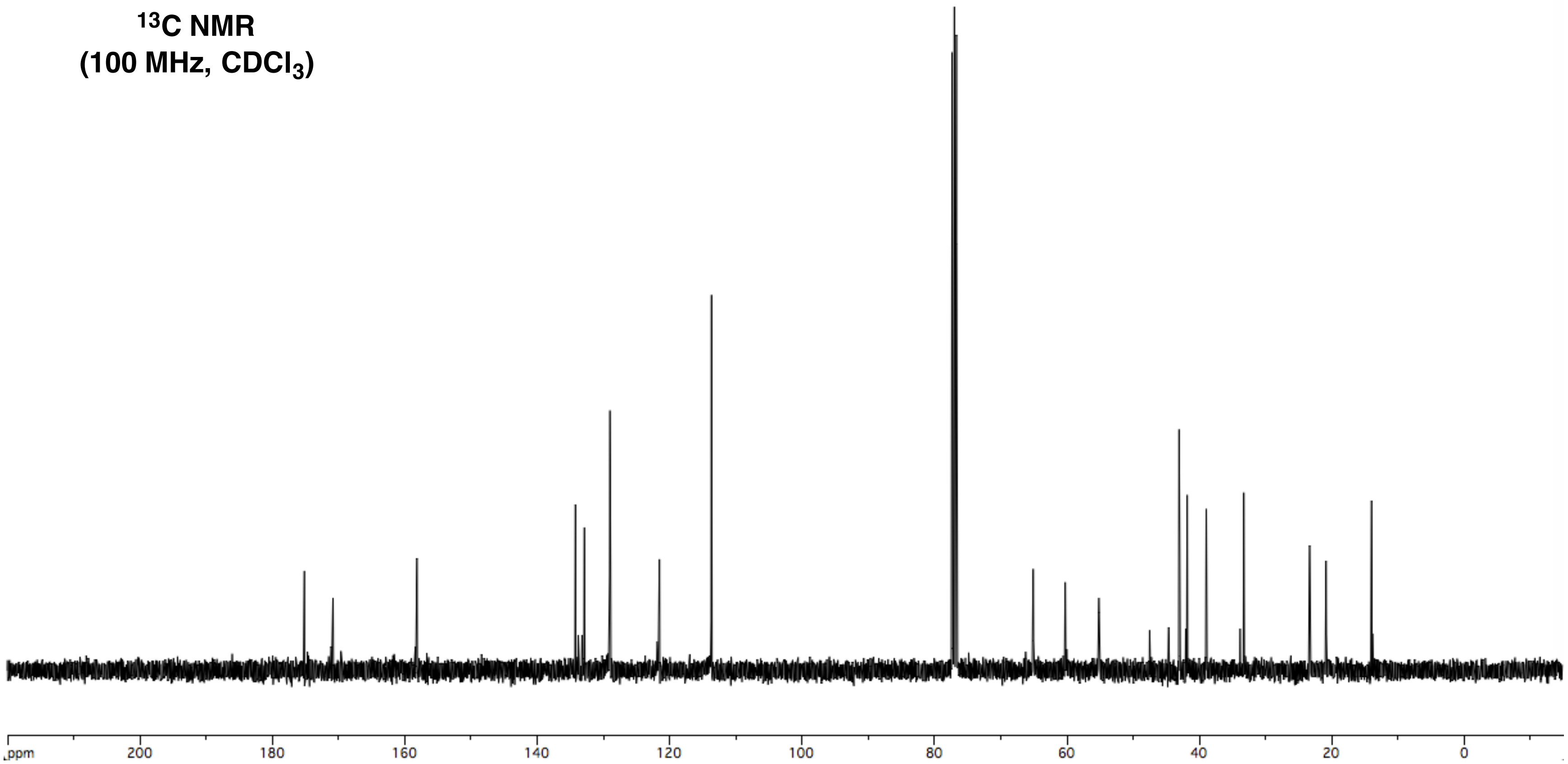

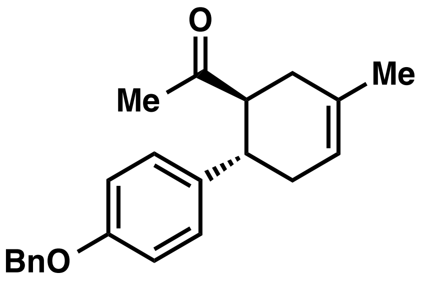

27

^{1}H NMR

(400 MHz, $CDCl_3$)

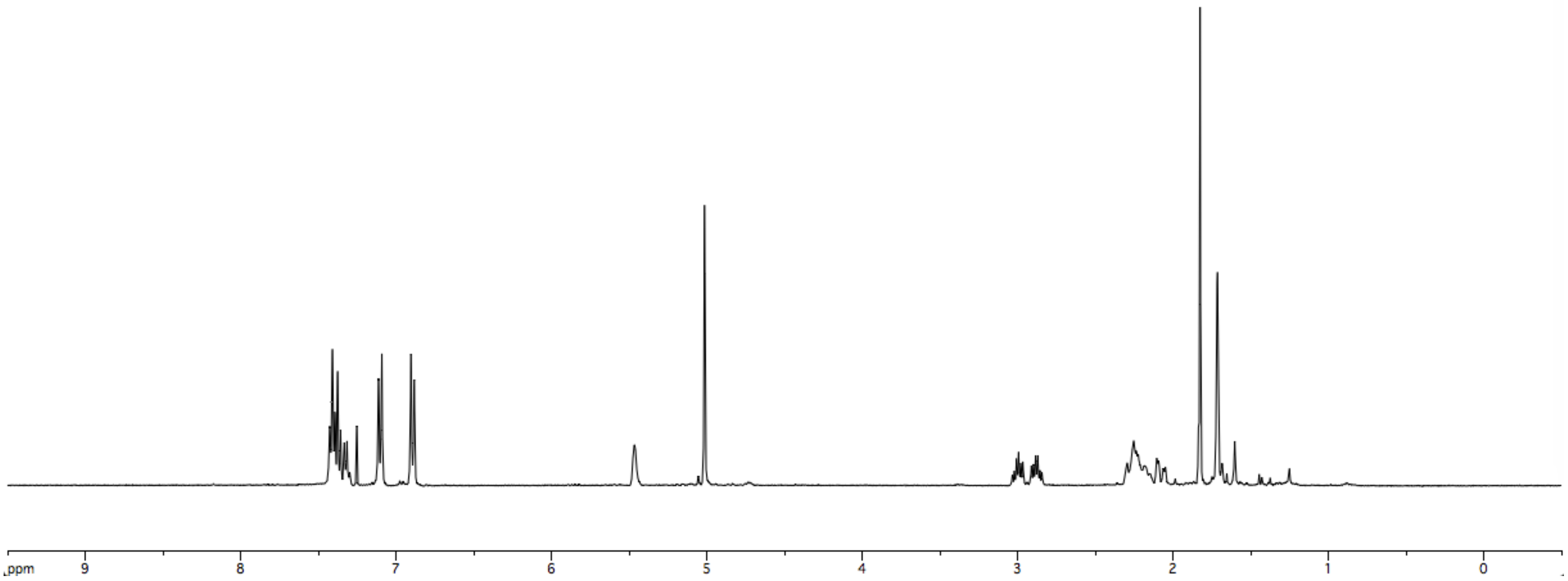

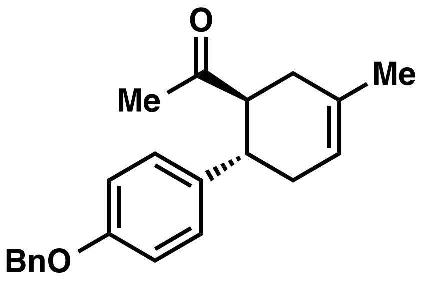

27

^{13}C NMR

(100 MHz, $CDCl_3$)

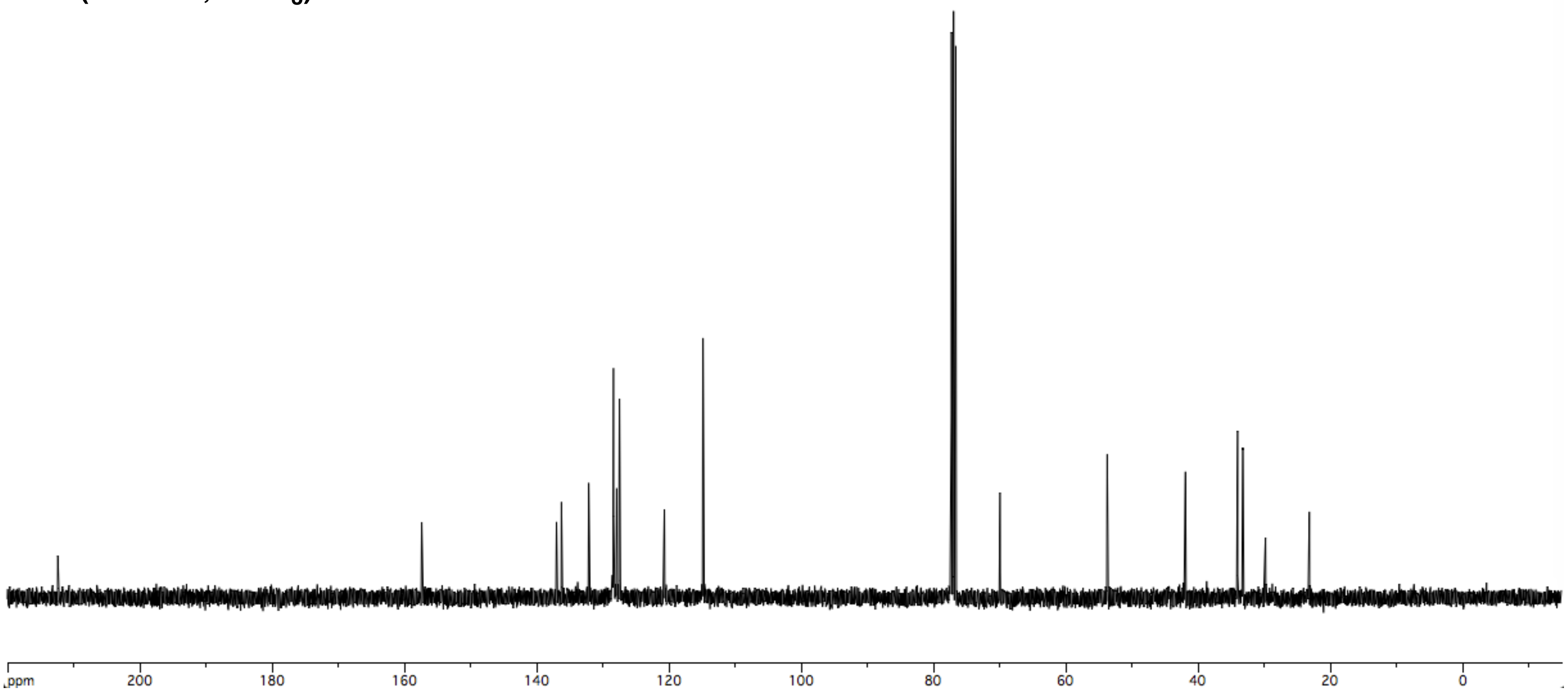

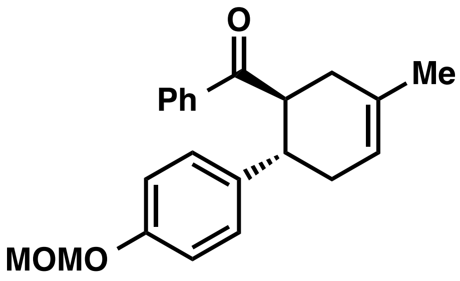

28

^{1}H NMR

(400 MHz, $CDCl_3$)

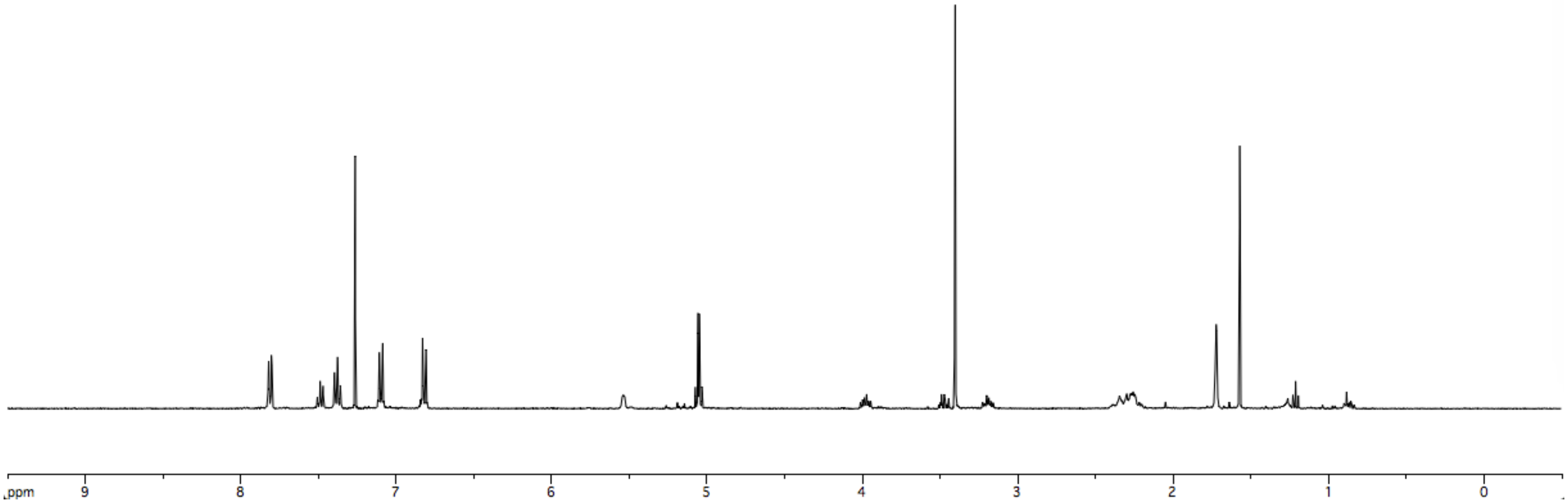

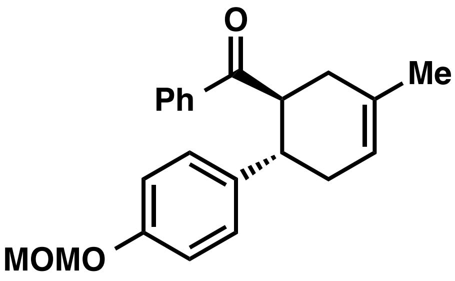

28

^{13}C NMR

(100 MHz, $CDCl_3$)

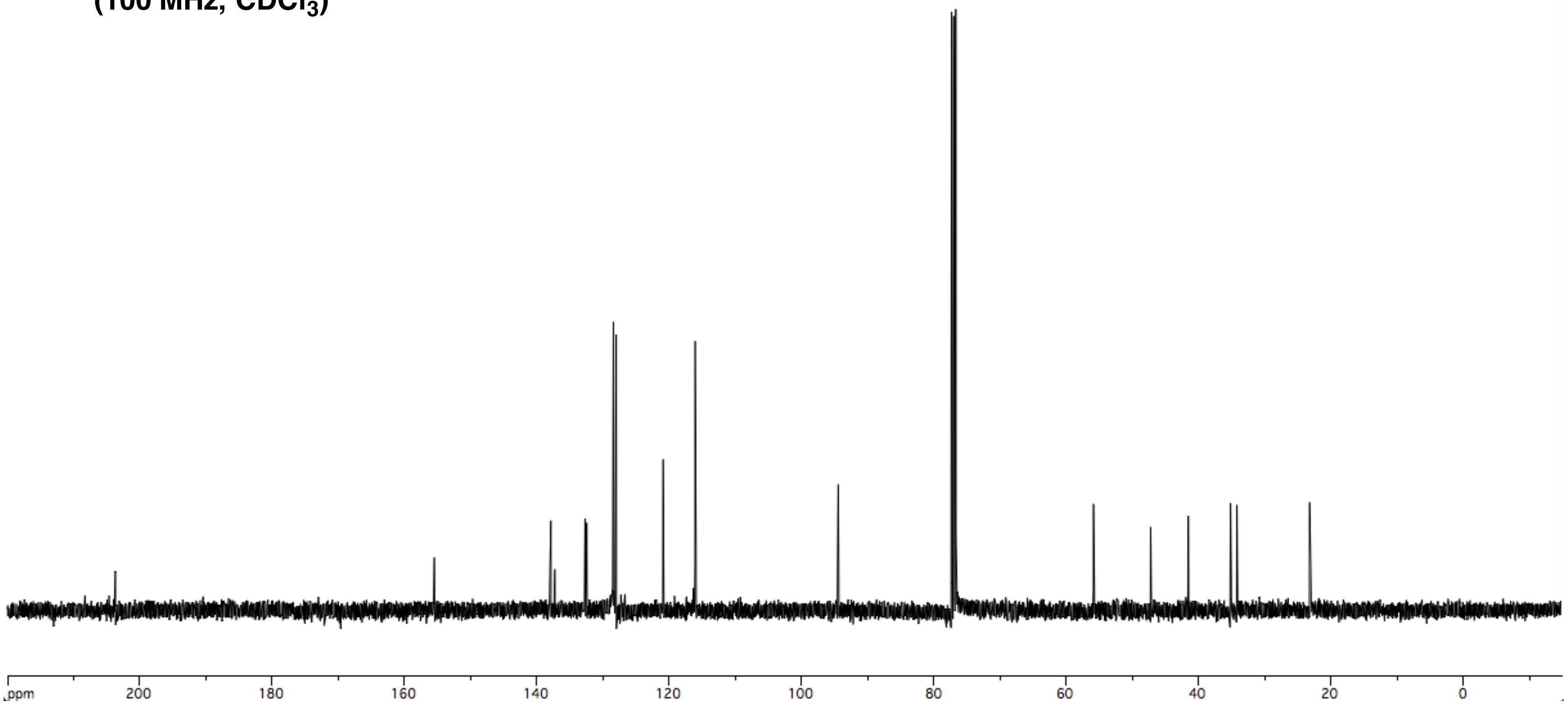

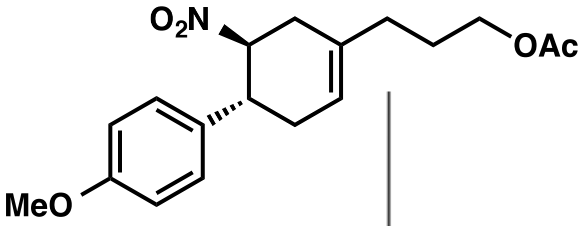

36

^{1}H NMR

(400 MHz, $CDCl_3$)

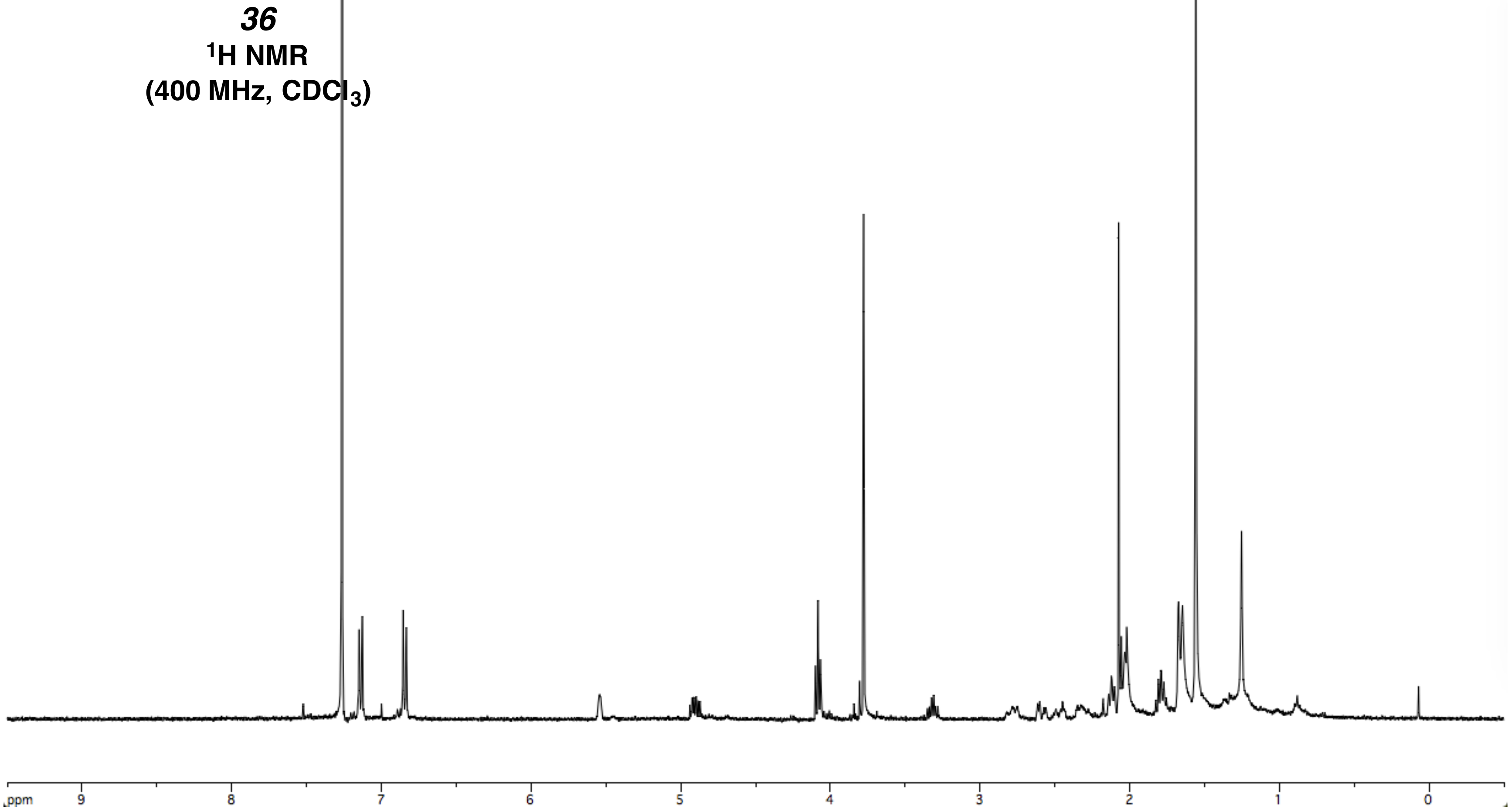

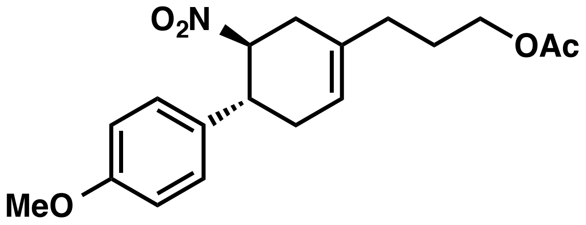

36

^{13}C NMR

(100 MHz, $CDCl_3$)

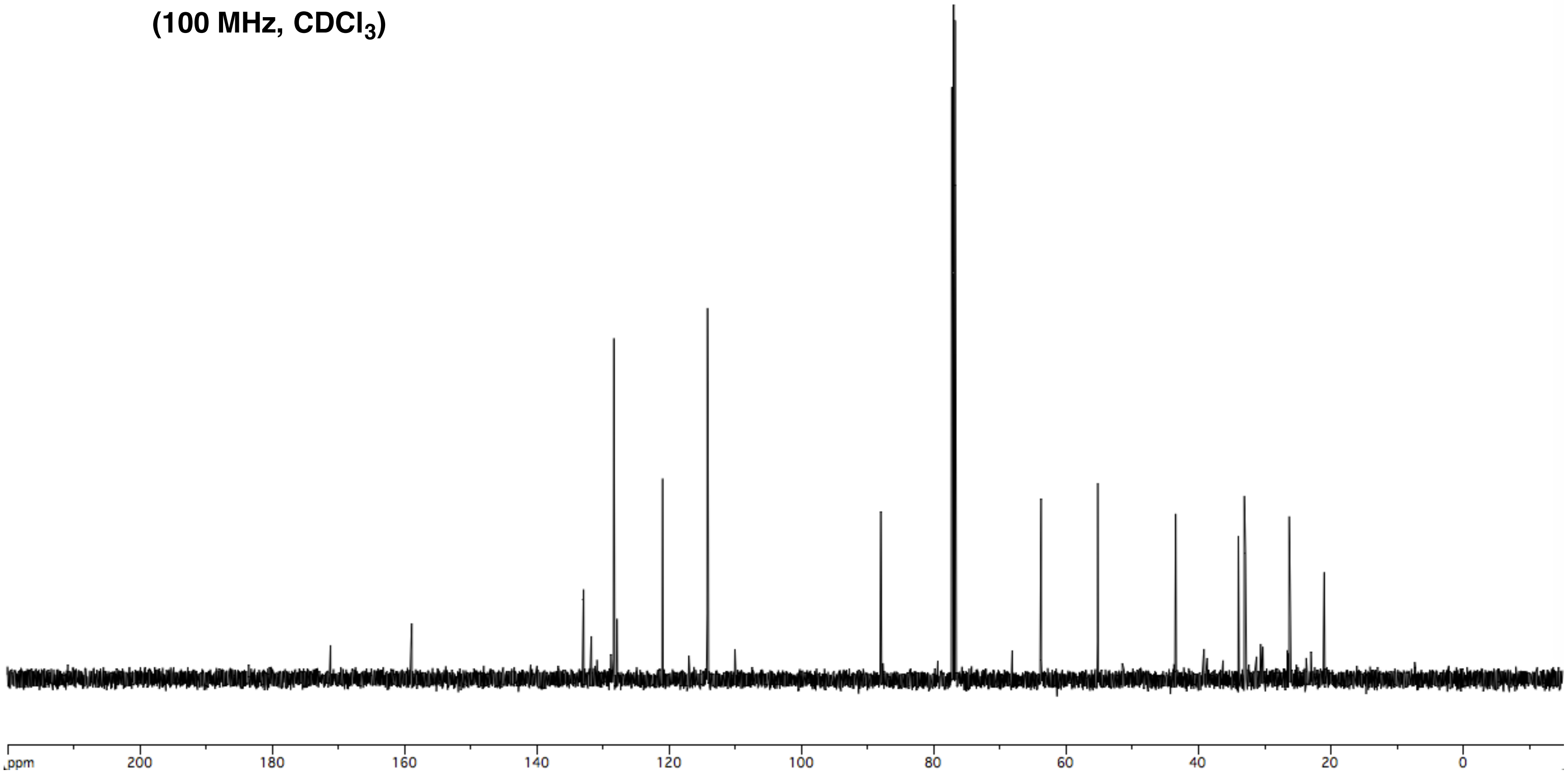

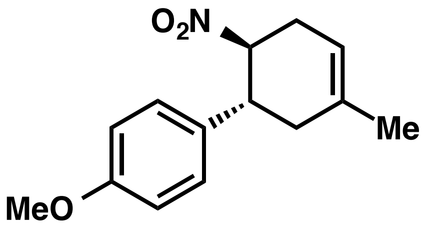

34

^{1}H NMR

(400 MHz, $CDCl_3$)

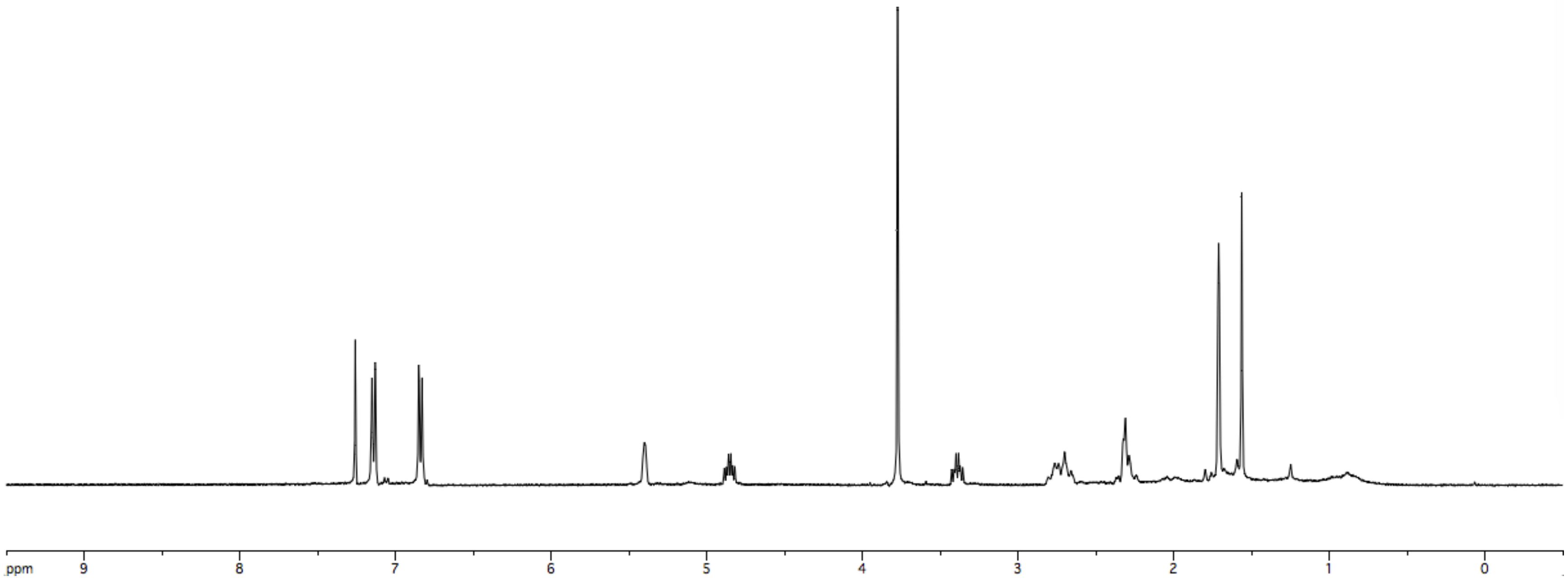

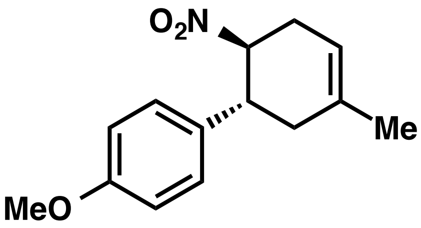

34

^{13}C NMR

(100 MHz, $CDCl_3$)

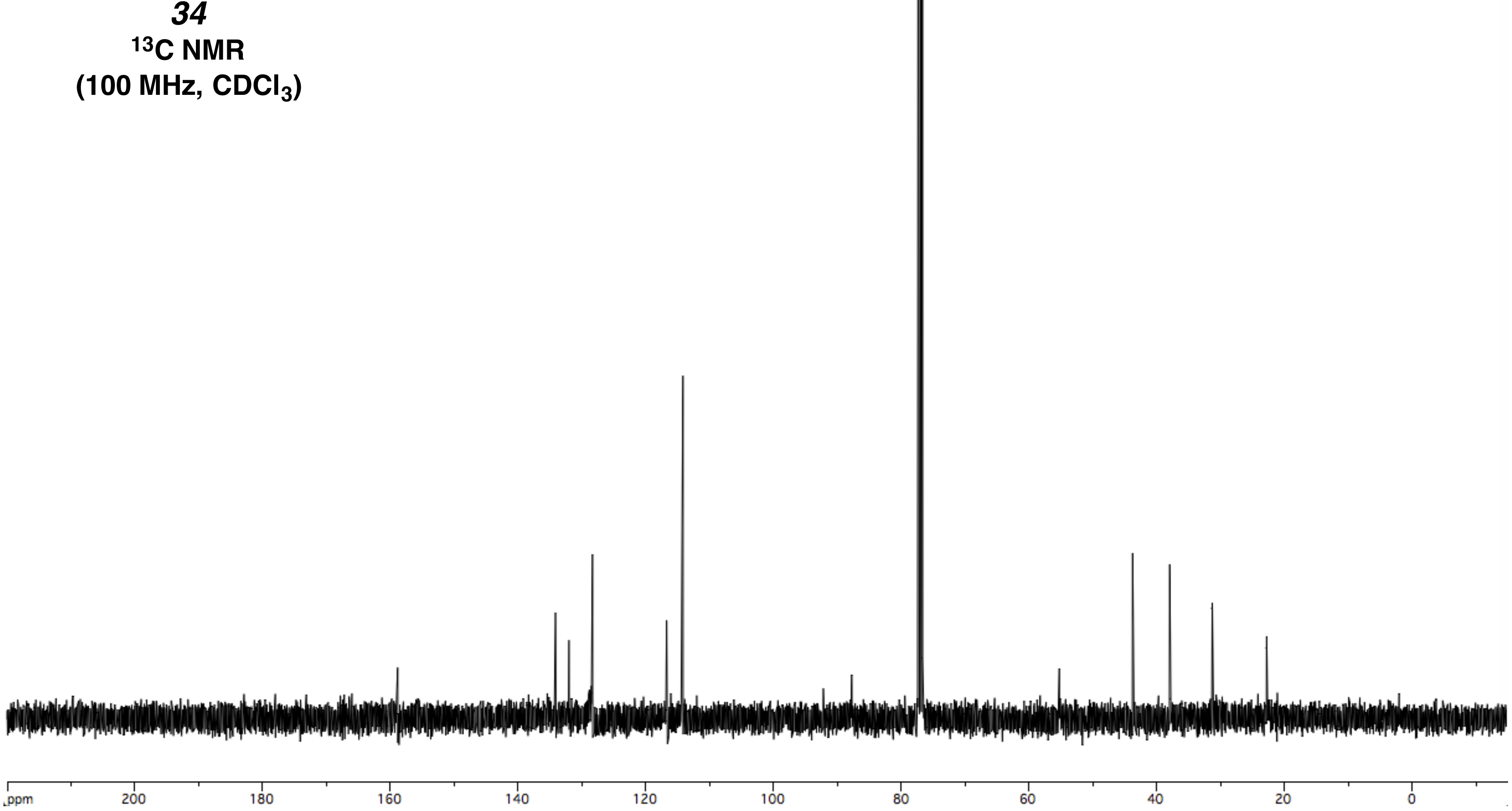

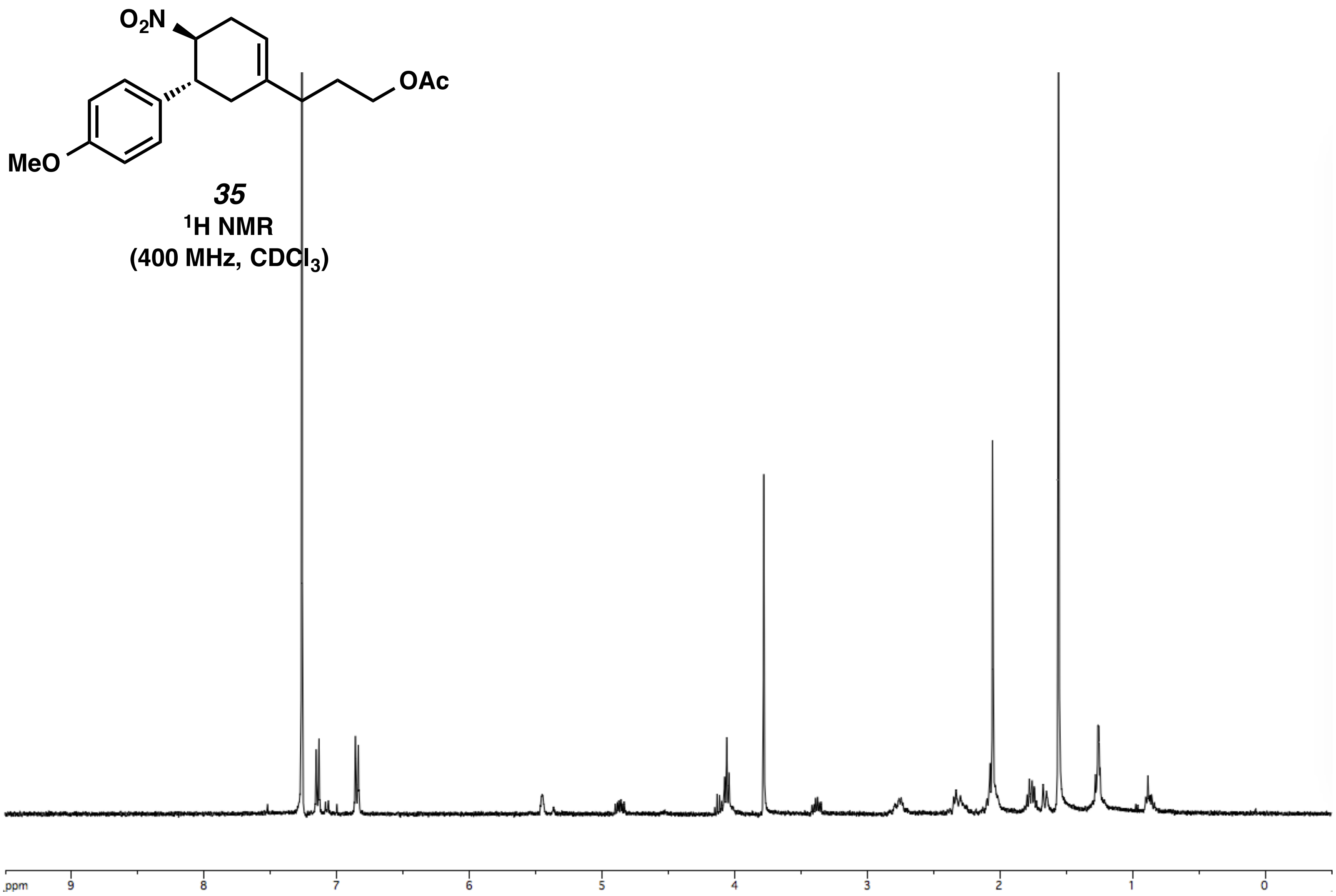
O2N
OAc
MeO
35
1H NMR
(400 MHz, CDCl3)
ppm 9 8 7 6 5 4 3 2 1 0

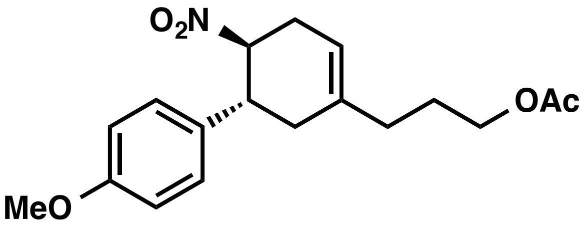

35

^{13}C NMR

(100 MHz, $CDCl_3$)

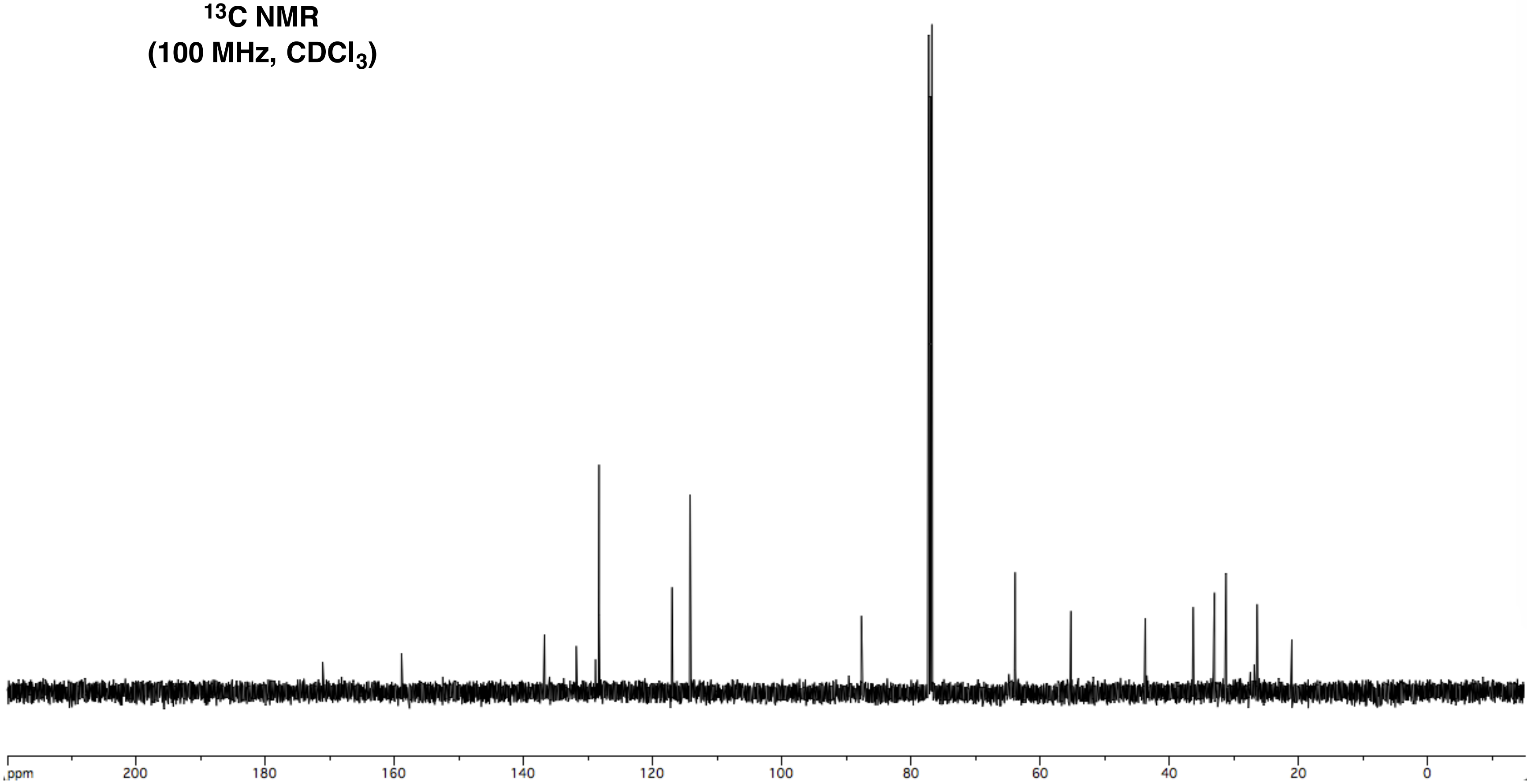

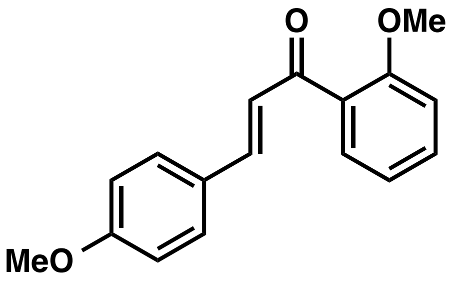

S3

^{1}H NMR

(400 MHz, $CDCl_3$)

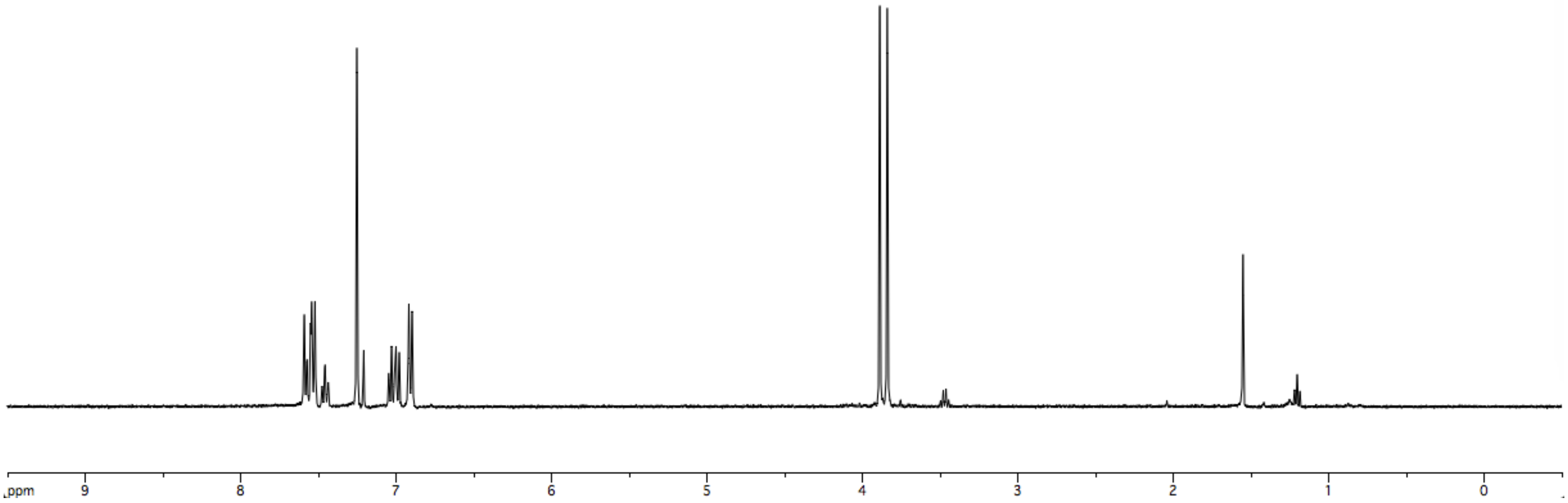

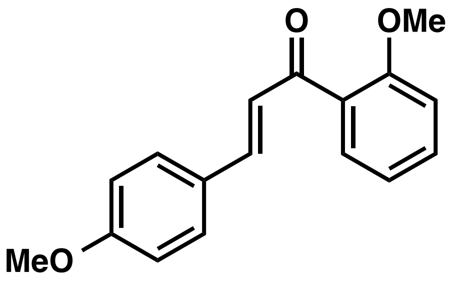

S3

^{13}C NMR

(100 MHz, $CDCl_3$)

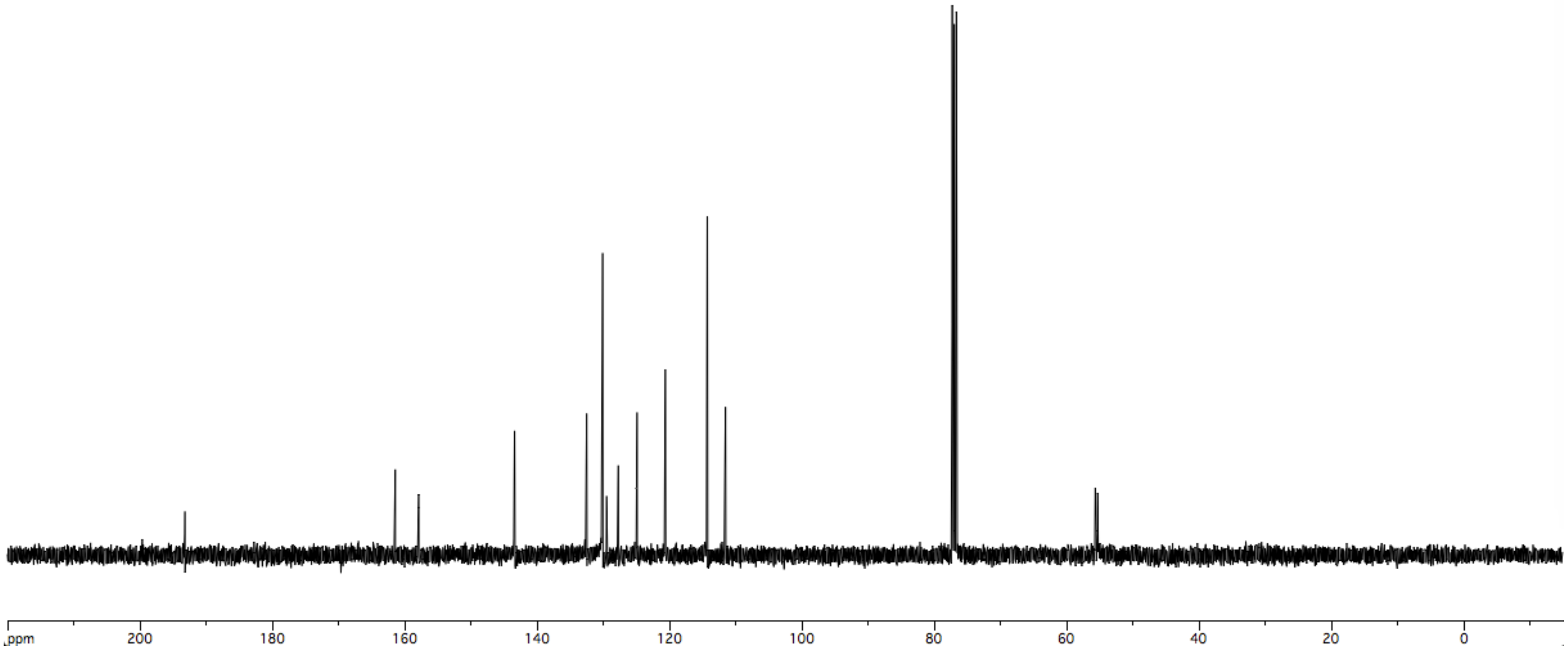

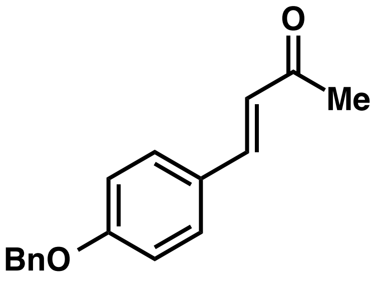

S18
^{1}H NMR
(400 MHz, $CDCl_3$)

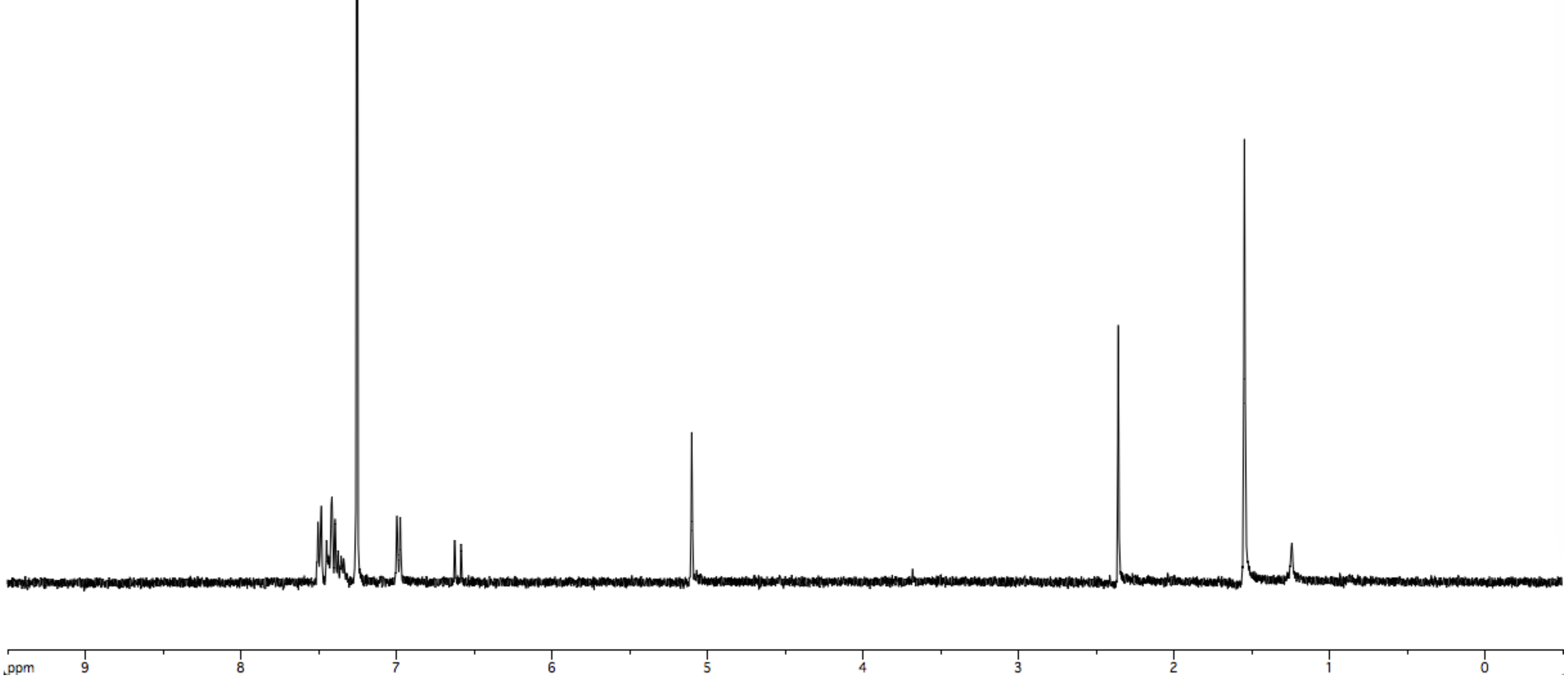

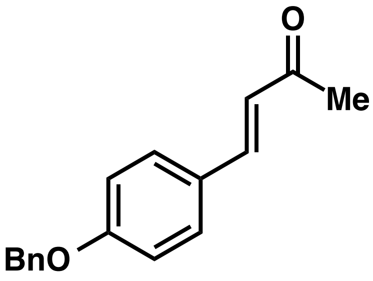

S18

^{13}C NMR

(100 MHz, $CDCl_3$)

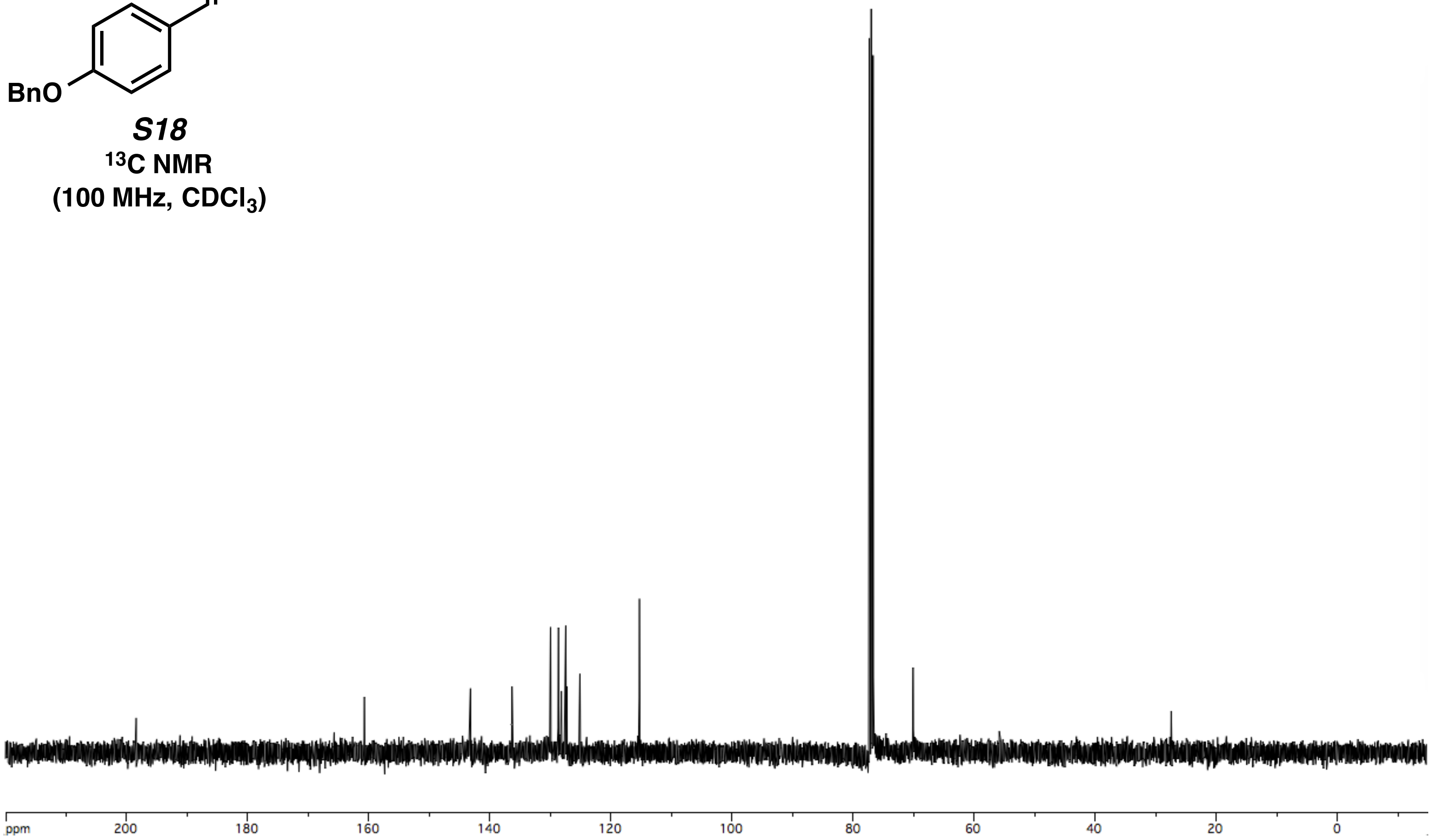

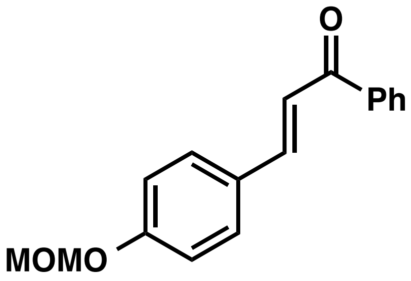

S19

¹H NMR

(400 MHz, $CDCl_3$)

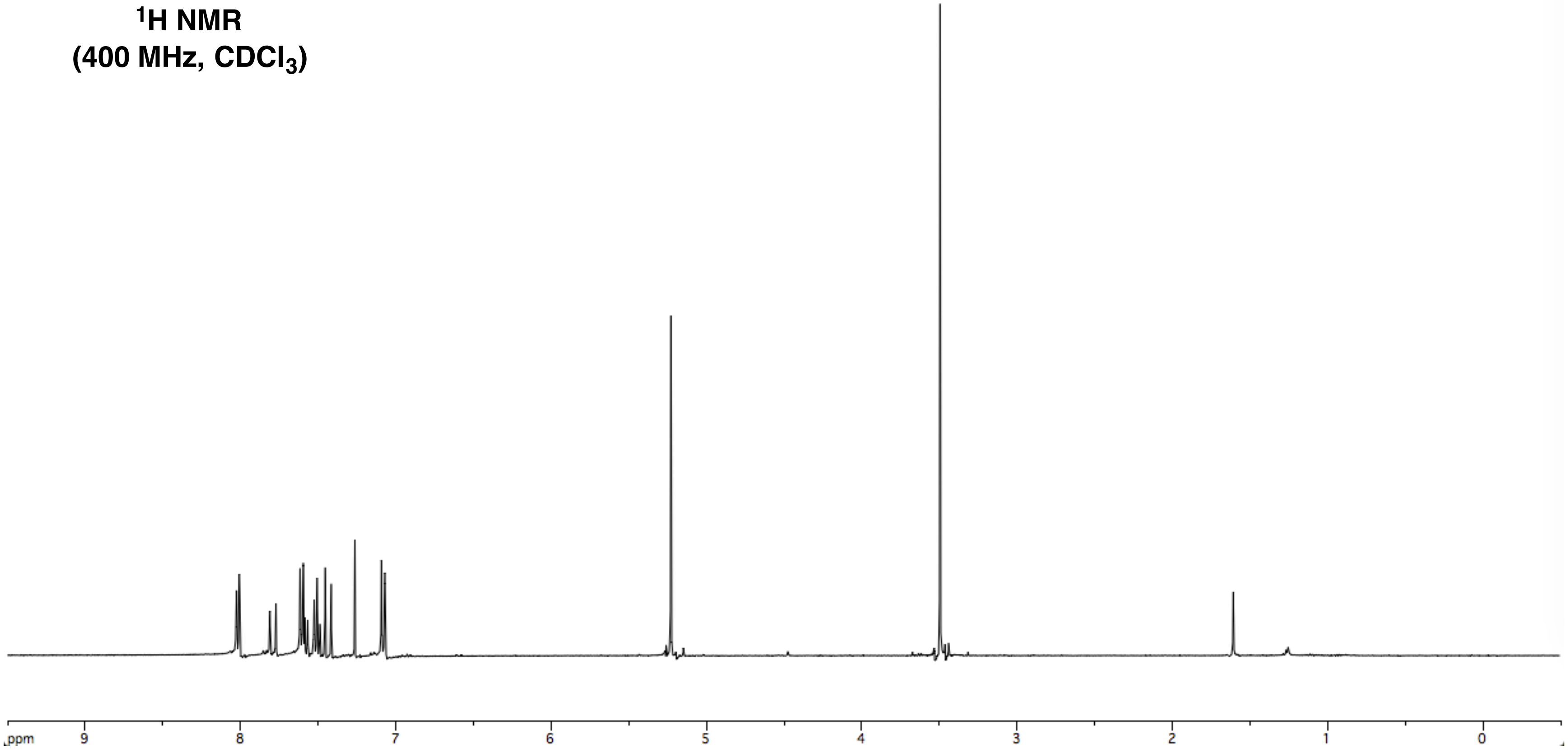

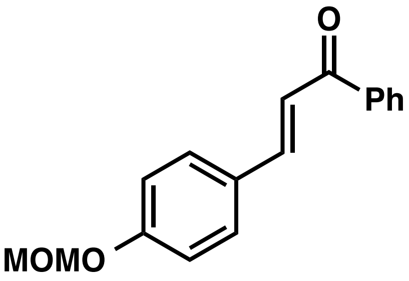

S19
^{13}C NMR
(100 MHz, $CDCl_3$)

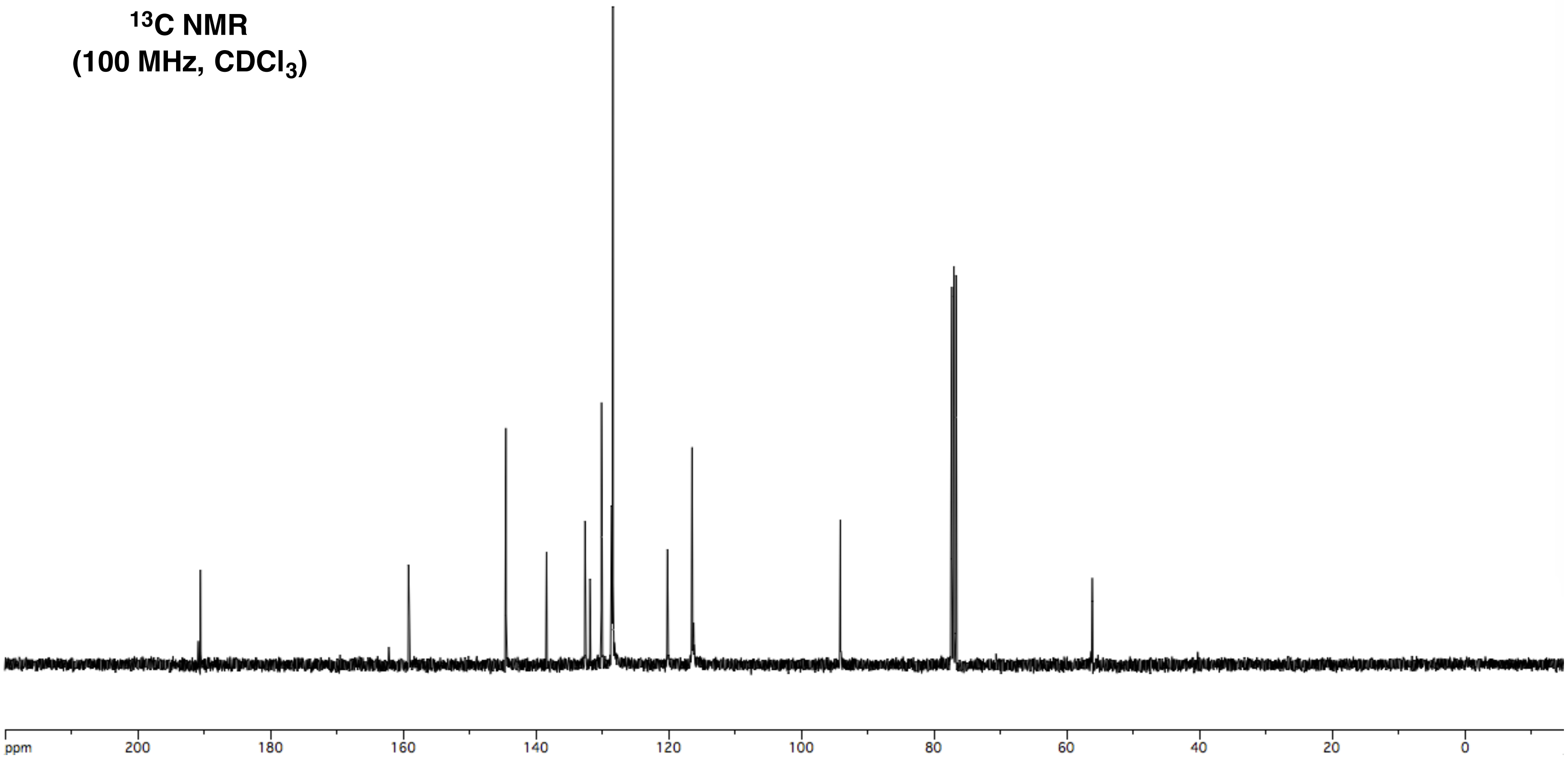

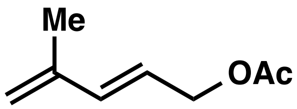

S17

^{1}H NMR

(400 MHz, $CDCl_3$)

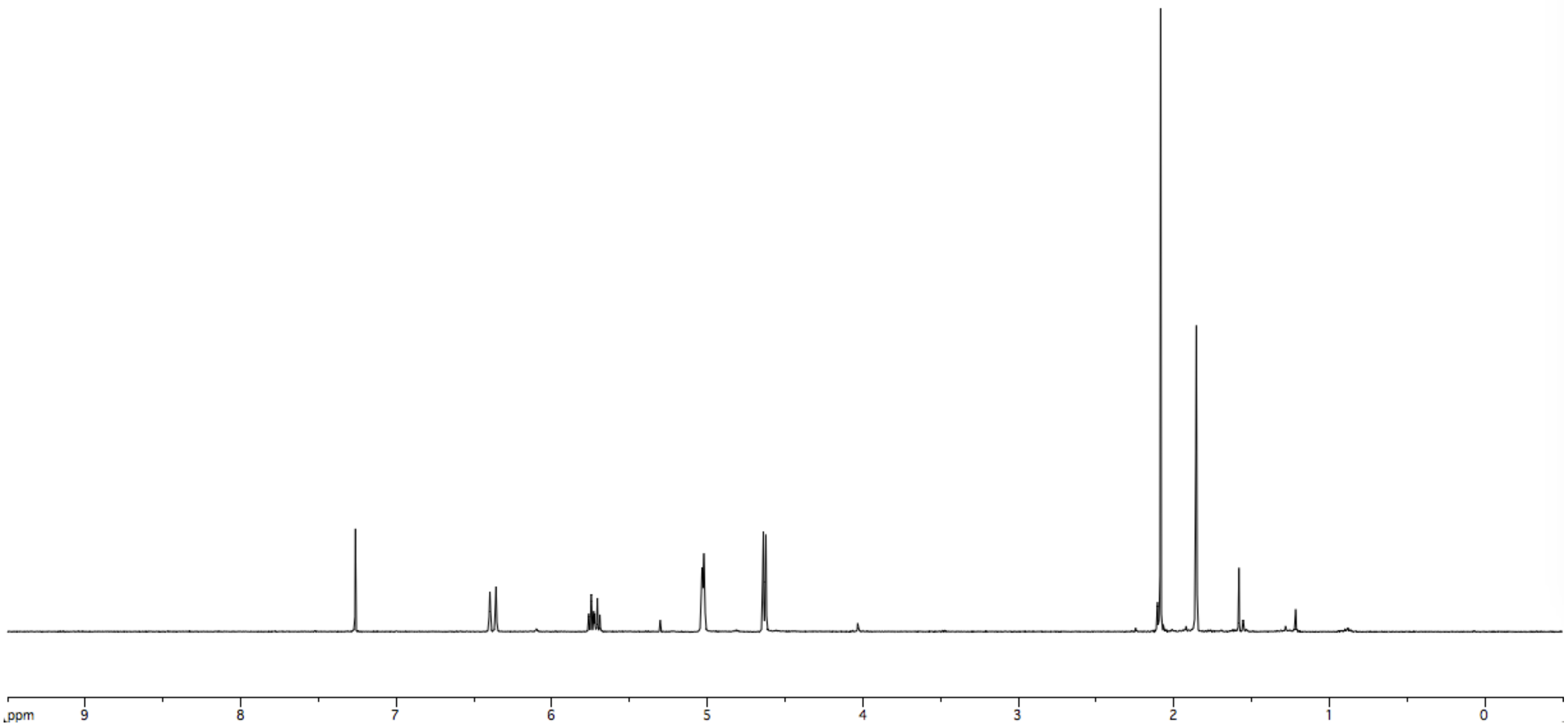

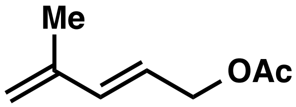

S17

^{13}C NMR

(100 MHz, $CDCl_3$)

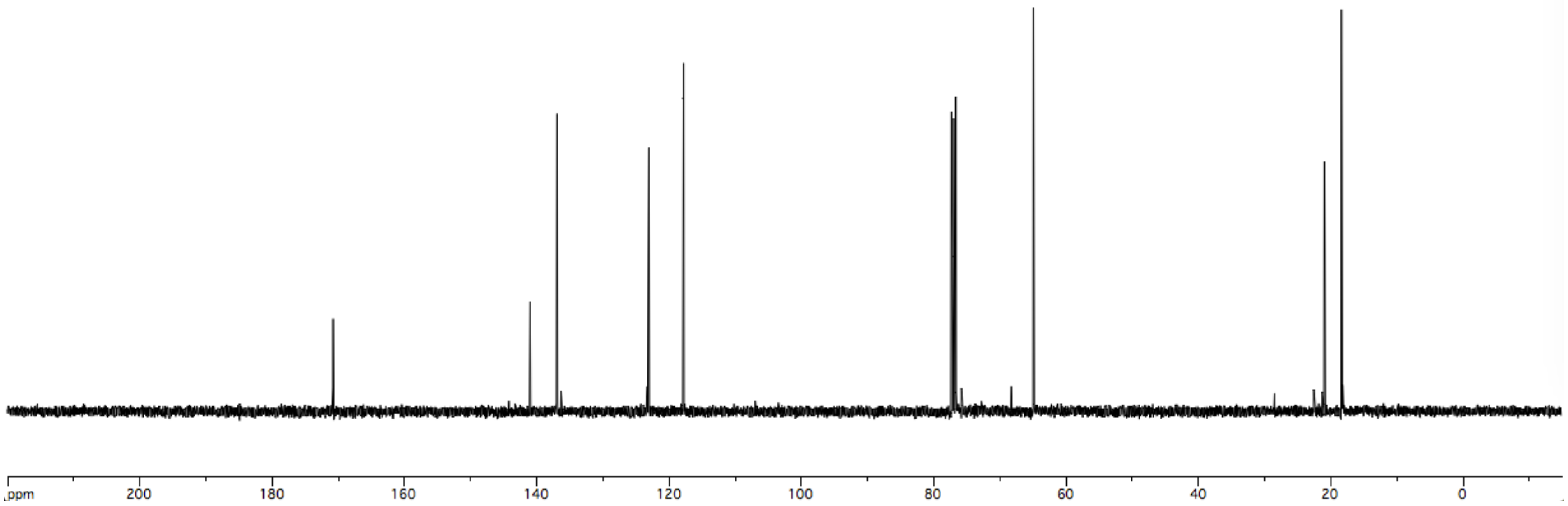

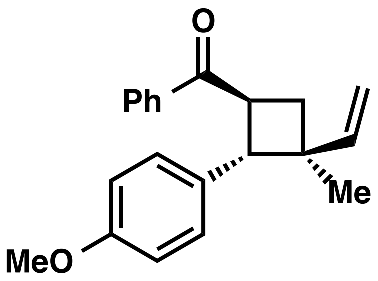

6
^{1}H NMR
(400 MHz, $CDCl_3$)

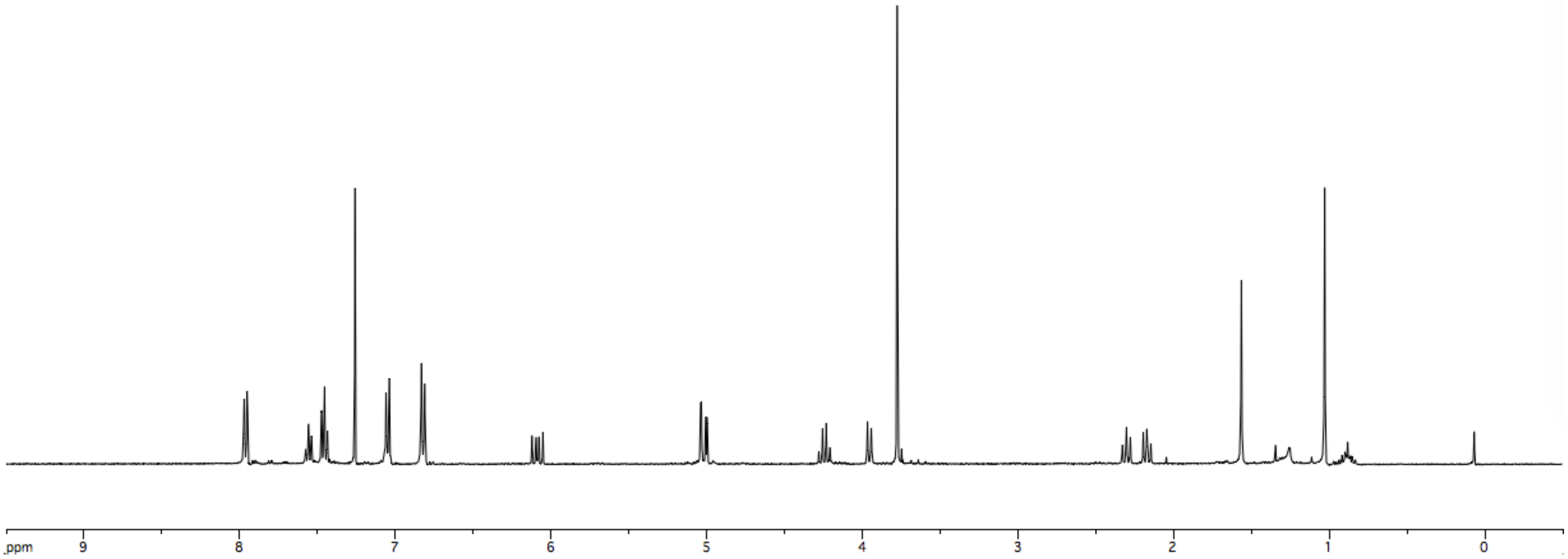

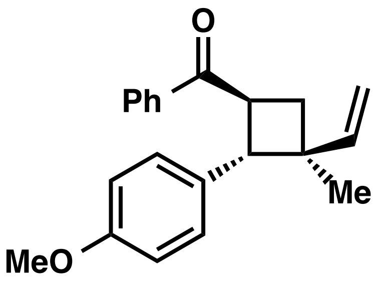

6

^{13}C NMR

(100 MHz, $CDCl_3$)

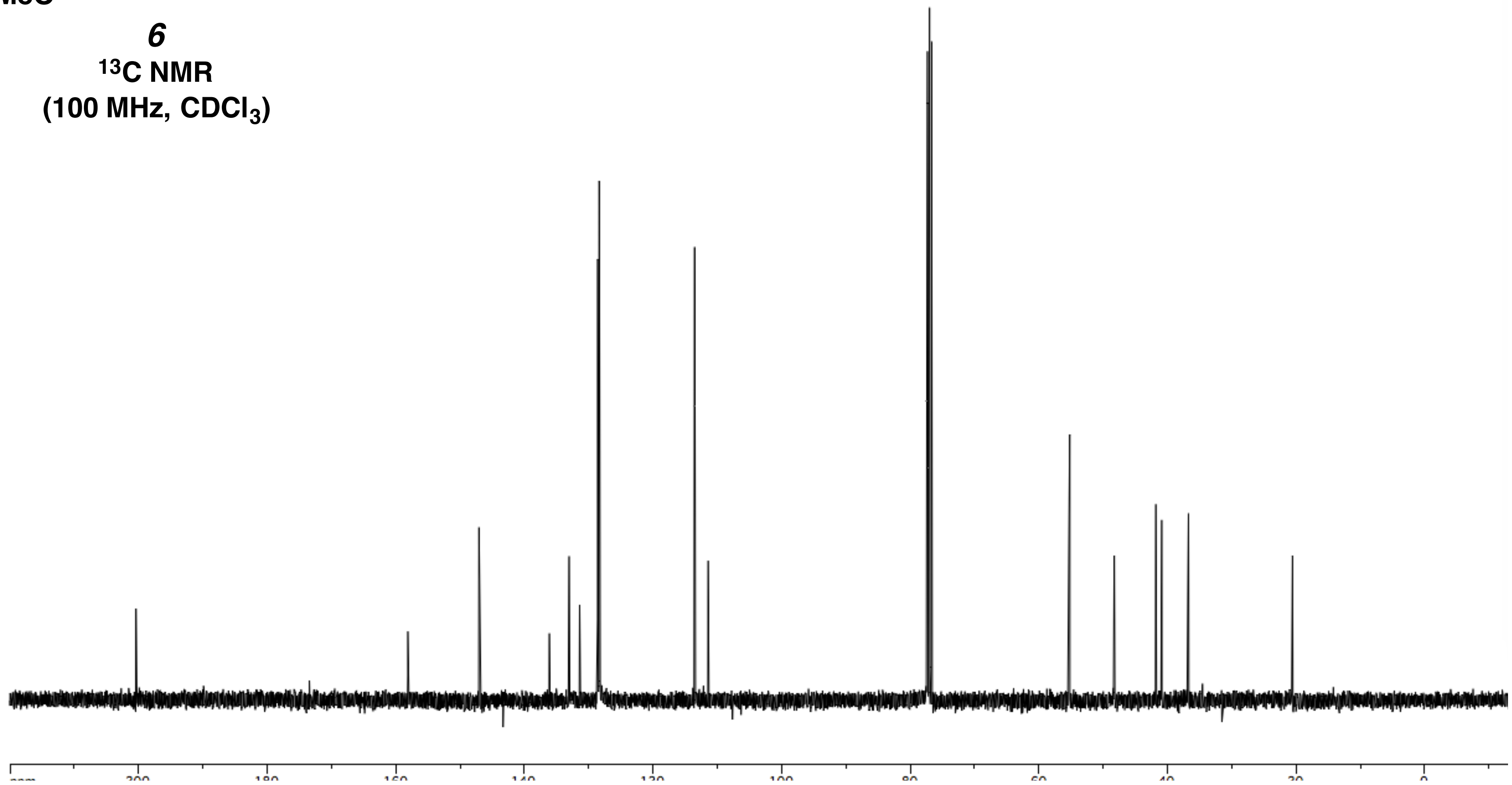

Supplement: Supplementary file 1 [file SC-008-C6SC03303B-s001.pdf]
